# Total synthesis of periploside A, a unique pregnane hexasaccharide with potent immunosuppressive effects

**DOI:** 10.1038/ncomms6879

**Published:** 2015-01-20

**Authors:** Xiaheng Zhang, Yu Zhou, Jianping Zuo, Biao Yu

**Affiliations:** 1 State Key Laboratory of Bioorganic and Natural Products Chemistry, Shanghai Institute of Organic Chemistry, Chinese Academy of Sciences, 345 Lingling Road, Shanghai 200032, China; 2 State Key Laboratory of New Drug Research, Shanghai Institute of Materia Medica, Chinese Academy of Sciences, Shanghai 201203, China

## Abstract

Periploside A is a pregnane hexasaccharide identified from the Chinese medicinal plant *Periploca sepium*, which features a unique seven-membered formyl acetal bridged orthoester (FABO) motif and potent immunosuppressive activities. Here, we show the synthesis of this molecule in a total of 76 steps with the longest linear sequence of 29 steps and 9.2% overall yield. The FABO motif is constructed via a combination of Sinaÿ’s and Crich’s protocol for the formation of orthoester and acetal glycosides, respectively. The 2-deoxy-β-glycosidic linkages are assembled stereoselectively with judicious choice of the glycosylation methods. The epimer at the spiro-quaternary carbon in the FABO motif has also been elaborated in a stereo-controlled manner. This epimer, as well as the synthetic analogues bearing the FABO motif, retain largely the inhibitory activities of periploside A against the proliferation of T-lymphocyte, indicating the importance of the chemical connection of the FABO motif to their immunosuppressive activity.

## Abstract (web summary)

Periploside A is a natural pregnane glycoside with promising immunosuppressive properties, but the presence of a seven-membered formyl acetal bridged orthoester makes it difficult to chemically synthesize. Here, the authors present a 76-step total synthesis of periploside A.

## Graphical abstract

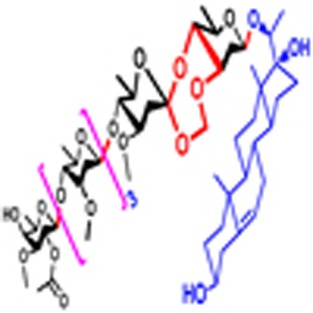

Periploside A (or Periplocoside E) is the prototypical member of a group of pregnane glycosides isolated from *Periploca sepium* and *P. forrestii* (Asclepiadaceae), which features a unique seven-membered formyl acetal bridged orthoester (FABO) linkage between two sugar units^1^,^2^,^3^,^4^,^5^,^6^,^7^. This natural product shows immunosuppressive activities, that is, it inhibits the ConA-induced T cell proliferation as potently as rapamycin and cyclosporin A while showing reasonably low toxicity (IC_50_=0.64 μM and CC_50_=10.1 μM)^5^,^6^. The immunosuppressive effects of periploside A has also been found significant in mice models^8^,^9^,^10^, that validates the folkloric reputation of the plant as a traditional Chinese medicine for rheumatoid arthritis^11^. Such autoimmune diseases as rheumatoid arthritis, multiple sclerosis, systemic lupus erythematosus and Crohn’s disease, are notoriously malignant and refractory, thus efficacious new immunosuppressant drugs have long been a quest^12^.

The FABO motif in periplosides, which has been found critical to their activities^6^, remains ambiguous in both the chemical connection and stereochemistry for over two decades. In 1987, Hikino *et al.* reported the first structural assignment of periploside A, in that the connection of the orthoester was proposed on the basis of extensive chemical degradation and spectroscopic analysis^1^. However, the stereochemistry of the quaternary anomeric carbon was overlooked but drawn in a configuration comprising an unusual α-(1→4)-glycosidic linkage (**1a**). Shortly afterwards, Itokawa *et al.*^2^,^3^,^4^ suggested a novel peroxy linkage (**1b**) for periplosides (and named as periplocosides), as misled by a conventional colour reaction of peroxides, although all the analytical data of periplocoside E were found identical to those of the previous periploside A (ref. 3). This peroxide structure was then accepted till 2011 (refs 5, 6), Zhao and coworkers acquired an X-ray diffraction of a single crystal of a periploside congener, confirming the originally assigned FABO connection^6^. Unfortunately, the stereochemistry of the spiro-quaternary carbon remained incorrect until we examined it carefully during our journey toward the synthesis of periploside A (ref. 7).

Herein, we report the first total synthesis of periploside A (**1**), employing total of 76 steps of transformations from glucal, methyl α-D-glucopyranoside and dehydroepiandrosterone, with the longest linear sequence of 29 steps and in 9.2% overall yield. The stereoselective construction of the FABO motif with either the natural or unnatural configuration at the anomeric spiro-quaternary center is achieved via alternative combination of Crich’s protocol for acetal glycoside synthesis and Sinaÿ’s protocol for glycosyl orthoester formation. The 2-deoxy-β-glycosidic linkages are synthesized by glycosylation with trifluoroacetimidate donors (**6**/**27**) equipped with 2-iodide, with digitoxosyl *ortho*-alkynylbenzoate donor (**8**) installed with bulky TBDPS group on the remote 3,4-OH, and with cymarosyl *ortho*-alkynylbenzoate donor (**2**) under the promotion of Ph_3_PAuOTf/TTBP. In addition, a preliminary structure–activity relationship of the synthetic periplosides against the proliferation of T-lymphocyte is provided, indicating the importance of the chemical connection of the FABO motif to the activities.

## Results

### Retrosynthetic analysis

**Figure 1:**
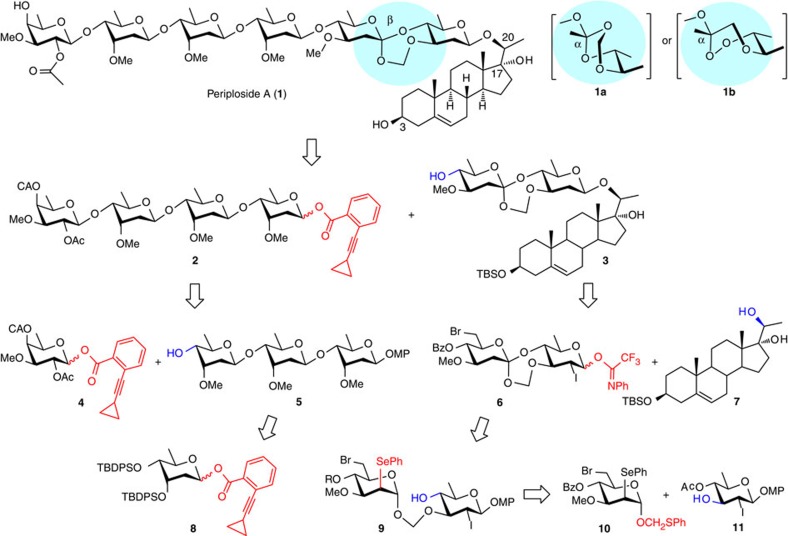
Periploside A 1 and the retrosynthetic analysis. Shadowed in pale blue are the FABO motifs that have been previously assigned; highlighted in red are leaving groups in donors and in blue are hydroxyl groups in acceptors. Ac, acetyl; CA, chloroacetyl; MP, 4-methoxyphenyl; TBDPS, *tert*-butyldiphenylsilyl; TBS, *tert*-butyldimethylsilyl.

Given the big size and linear structure of the target molecule, a convergent synthesis was desired^13^. Thus, periploside A (**1**) was disconnected into two fragments of similar complexity, that is, tetrasaccharide donor **2** and pregnane FABO disaccharide **3** (Fig. 1). The (1→4)-cymarosyl linkage between the tetrasaccharide unit and **3** would be difficult to construct in favour of formation of the thermodynamically unfavored β-anomer^14^,^15^,^16^,^17^; the glycosylation protocol with *ortho*-alkynylbenzoates as leaving groups (as in **2**) under the catalysis of a gold(I) complex could address this problem, in that substitution via an α-glycosyloxypyrylium or α-triflate intermediate might get invoked^18^. The β-(1→4)-cymarosyl linkages in tetrasaccharide **2**, however, could be built stereoselectively under the influence of a substituent at the axial 3-OH of a digitoxosyl donor, which could subsequently be converted into the required methyl group. In this respect, we had screened carefully during the synthesis of digitoxin and gordonoside F and found gold(I)-catalysed glycosylation with digitoxosyl *ortho*-cyclopropylethynylbenzoate equipped with two bulky TBDPS groups at 3,4-OH (that is, **8**) to be an optimal choice^15^,^17^. After furnishing the cymarose trisaccharide **5**, its coupling with the terminal digitalose unit could be realized with donor **4**, in that the formation of the β-linkage should be secured by participation of the 2-*O*-acetyl group; and the 4-*O*-chloroacetyl group was selectively removable afterwards. The *p*-methoxyphenyl (MP) group was employed throughout (as in **5**, **9** and **11**) as the anomeric protecting group, which could remain intact before cleavage and subsequently converting the saccharides into donors (for example, **2** and **6**).

Uncertainty lies in the synthesis of FABO disaccharide **3**. Condensation of 3β-silyloxy-pregnene-17α,20α-diol **7** with disaccharide trifluoroacetimidate **6**, one of the most reliable type of glycosylation donors^19^, which was equipped with an equatorial iodide at C2 (ref. 20), would lead to the desired C20-*O*-β-glycoside **3** in a regio- and stereo- selective manner. However, few clues occur in the literatures, which could lead to the construction of a disaccharide precursor consisting of the FABO linkage. The most promising approach turned out to be a combination of Crich’s protocol to form the formyl acetal glycosidic linkage (from a thiomethyl glycoside donor such as **10**; ref. 21) and Sinaÿ’s protocol to synthesize the anomeric orthoester via a ketene acetal intermediate (derived from a 2-selenoglycoside such as **9**; refs 22, 23, 24). However, the tolerance of the formyl acetal (in **9**) towards the strong conditions required for Sinaÿ’s oxidation–elimination–cyclization sequence and the stereochemistry in the formation of the seven-membered spiro-orthoester were beyond estimation.

### Synthesis of tetrasaccharide donor 2

**Figure 2:**
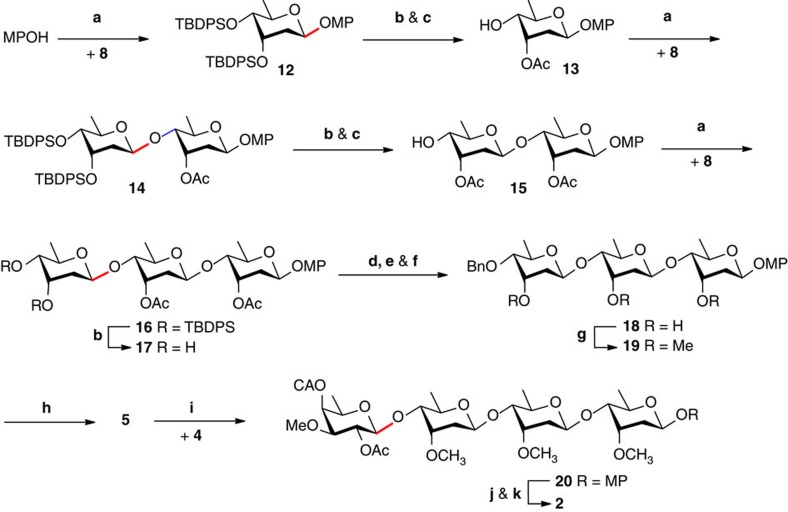
Synthesis of tetrasaccharide donor 2. Highlighted in red are the nascent glycosidic bonds. (**a**) Ph_3_PAuNTf_2_ (0.1 equiv), toluene, 4 Å MS, −40 °C; 95% and β only (for **12**); 99% and β only (for **14** and **16**); (**b**) TBAF, THF, 0 °C to RT; 99% (from **12**); 94% (from **14**); 96% (from **16**); (**c**) CH_3_(OMe)_3_, *p*-TsOH, RT, 93% for (**13**); 91% (for **15**); (**d**) ^*n*^Bu_2_SnO, MeOH, reflux; (**e**) BnBr, DMF, CsF, RT; (**f**) LiOH, THF, H_2_O, RT; 87% (for three steps); (**g**) MeI, NaH, DMF, 0 °C to RT, 99%; (**h**) Pd(OH)_2_/C, H_2_ (1 atm), Et_3_N, EtOAc, MeOH, 50 °C, 93%; (**i**) Ph_3_PAuNTf_2_ (0.2 equiv), toluene, 5 Å MS, −50 °C to RT, 93%, β/α=4/1; (**j**) Ag(DPAH)_2_, CH_3_CN, H_2_O, 0 °C to RT, 95%; (**k**) *o*-cyclopropylethynylbenzoic acid, EDCI, DMAP, 4 Å MS, CH_2_Cl_2_, RT, 99%. DMAP, 4,4-dimethylaminopyridine; DPAH, bis(hydrogen dipicolinate); EDCI, *N*-ethyl-*N*′-(3-dimethylaminopropyl)carbodiimide hydrochloride; MS, molecular sieves; TBAF, tetrabutylammonium fluoride.

The desired digitalosyl and digtoxosyl *ortho*-cyclopropylethynylbenzoates **4** and **8** were prepared in a robust manner from D-fucose (13 steps and 37% overall yield) (see Supplementary Methods) and methyl α-D-glucopyranoside (10 steps and 47% overall yield)^15^, respectively. Subjection of **8** to glycosidation with *p*-methoxyphenol under optimized conditions (0.1 equivalent (equiv) Ph_3_PAuNTf_2_, toluene, 4 Å molecular sieves (MS), −40 °C) led to β-glycoside **12** as the sole anomer in an excellent 95% yield (Fig. 2). Removal of the 3,4-di-*O*-TBDPS group followed by selective acetylation of the axial 3-OH via orthoester formation/hydrolysis provided glycoside **13** bearing a free 4-OH (92%, two steps)^25^. Similar transformations were then applied to extend monosaccharide **13** to trisaccharide **17**, in that the glycosidation of **8** with monosaccharide acceptor **13** and disaccharide acceptor **15** led to the corresponding coupled 2-deoxy-β-saccharides nearly quantitatively. Trisaccharide 3′′,4′′-diol **17** was then subjected to a tin-mediated selective benzylation on the equatorial 4′′-OH (ref. 26); cleavage and scramble of the acetyl groups on the axial hydroxyl groups were detected, nevertheless, exposure of the resultant mixture to LiOH afforded **18** in excellent yield (87%, three steps). The three axial hydroxyl groups in **18** were then methylated (99%). Unexpectedly, hydrogenolysis of the 4′′-*O*-benzyl group in trisaccharide **19** under conventional conditions (Pd(OH)_2_/C, H_2_, room temperature (RT)) led to cleavage of the glycosidic linkages, testifying the vulnerability of the cymarosyl-β-(1→4)-linkage. Thus, Et_3_N was added to sequester any nascent proton; the hydrogenolysis proceeded sluggishly, nevertheless, leading to the desired trisaccharide **5** in a satisfactory 93% yield at 50 °C for 2 days.

Given the acid lability of the cymarosyl-β-(1→4)-linkage, it was not fully surprising to find that glycosylation of trisaccharide acceptor **5** with digitalosyl donor **4** in the presence of Ph_3_PAuOTf led to considerable cleavage of the trisaccharide. Ph_3_PAuNTf_2_ was found to be more stable towards moisture (thus less acidic)^27^, therefore, it catalysed the condensation of **5** and **4** smoothly. However, in spite of the presence of 2-*O*-acetyl group in donor **4**, the glycosylation led to anomeric mixtures of the coupled tetrasaccharide. Under optimized conditions (0.2 equiv Ph_3_PAuNTf_2_, toluene, 5Å MS, −50 °C to RT), tetrasaccharide **20** was obtained in 93% yield with β/α ratio of 4:1, which was separable on silica gel column chromatography. Removal of the anomeric MP group in tetrasaccharide **20** with CAN was not successful under a variety of conditions, resulting unavoidably degradation of the cymarosyl-β-(1→4)-linkages. Fortunately, a mild oxidizing agent, Ag(DPAH)_2_, could achieve this task (CH_3_CN/H_2_O, 0 °C to RT) to afford smoothly the corresponding hemiacetal (95%) (ref. 28), which was then subjected to the formation of *ortho*-cyclopropylethynylbenzoate **2** (99%) (see Supplementary Methods).

### Attempts at construction of the FABO motif

**Figure 3:**
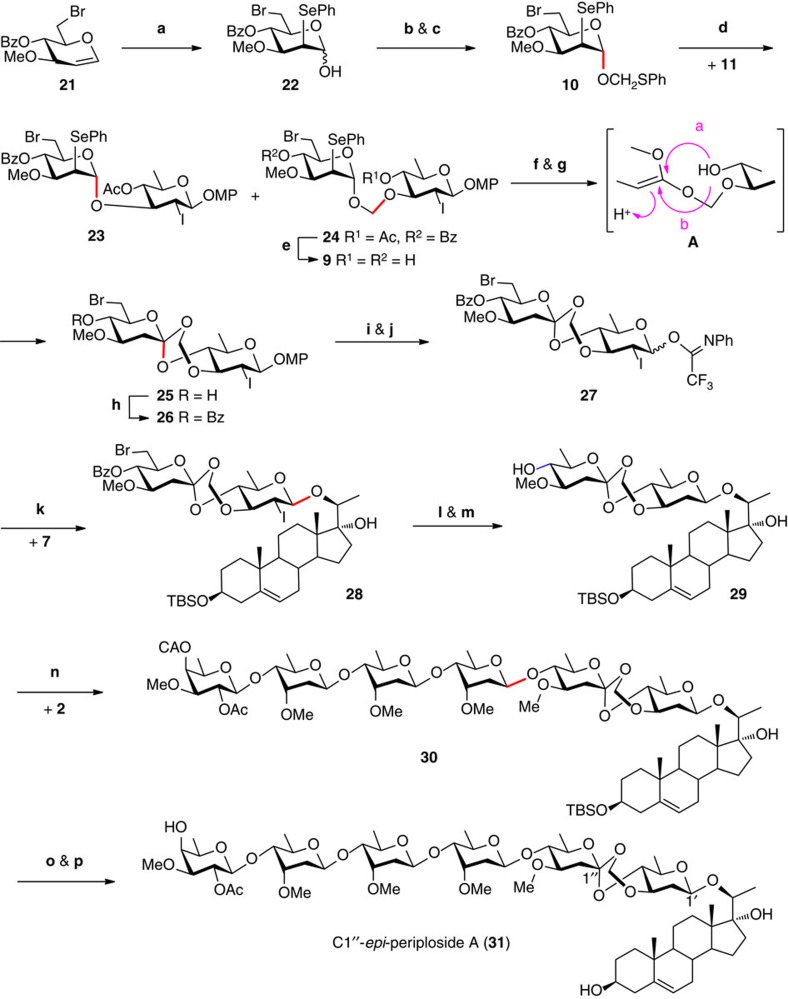
Construction of the FABO motif and synthesis of C1′′-*epi*-periploside A (31). Highlighted in red are the nascent glycosidic bonds. (**a**) PhSeCl, CH_3_CN, −40 °C, 78%; (**b**) DAST, THF, −30 °C to RT; (**c**) PhSCH_2_OH, SnCl_2_, CH_2_Cl_2_, 4 Å MS, −40 °C to RT; 58% (for two steps); (**d**) NIS, TfOH, CH_2_Cl_2_, −30 °C, 75% (for **24**), 20% (for **23**); (**e**) MeONa, MeOH, CH_2_Cl_2_, RT, 93%; (**f**) NaIO_4_, NaHCO_3_, MeOH, CH_2_Cl_2_, H_2_O, RT, 99%; (**g**) vinyl acetate, toluene, DIPA, Mw, 145 °C, 20 min, 85%; (**h**) BzCl, Et_3_N, DMAP, CH_2_Cl_2_, 0 °C to RT, 99%; (**i**) Ag(DPAH)_2_, CH_3_CN, H_2_O, 0 °C to RT, 90%; (**j**) *N*-phenyl-2,2,2-trifluoroacetimidoyl chloride, Cs_2_CO_3_, CH_2_Cl_2_, RT, 95%; (**k**) TBSOTf, CH_2_Cl_2_, 5 Å MS, −78 °C, 85%, β/α=3/1; (**l**) Et_3_B, Bu_3_SnH, toluene, RT, 97%; (**m**) MeONa, MeOH, CH_2_Cl_2_, RT, 99%; (**n**) Ph_3_PAuOTf (0.8 equiv), TTBP, CH_2_Cl_2_, 4 Å MS, −20 °C to −10 °C, 64% (87% b.r.s.m.), β/α=2.1/1; (**o**) thiourea, pyridine, EtOH, 80 °C; (**p**) HF·py, pyridine; THF, 0 °C to RT; 91% (for two steps). DAST, (diethylamino)sulfur trifluoride; DIPA, diisopropylamine; TBSOTf, *tert*-butyldimethylsilyl trifluoromethane sulfonate; Mw, microwave; TTBP, 2,4,6-tri-*tert*-butylpyrimidine.

We have tried a number of approaches to the construction of the FABO disaccharide motif, those include condensation of sugar lactones with vicinal diols in the presence of formaldehyde or its surrogates^29^,^30^, transacetalation of sugar lactones with sugar 4-hydroxyl-3-methyoxymethyl ether derivatives^31^, and intramolecular hydrogen atom transfer reactions of formyl acetal linked disaccharides^21^,^32^,^33^. However, no coupled FABO products have ever been detected, that prompted us to focus our attention on the planned Crich–Sinaÿ approach (Fig. 3). Thus, starting from D-glucal triacetate, 3-*O*-methyl-6-bromo-glucal **21** and *p*-methoxyphenyl 2,6-dideoxy-2-iodo-glucoside **11** were prepared in seven steps (66% overall yield) and 13 steps (18% overall yield), respectively (see Supplementary Methods). Glucal **21** was converted into the desired phenylthiomethyl 2-phenylseleno-α-D-mannopyranoside **10** in three steps (45%), that is, addition with PhSeCl to provide 2-phenylseleno-D-mannopyranose **22**, conversion of the lactol into fluoride with DAST, and subsequent glycosidation with PhSCH_2_OH in the presence of SnCl_2_ (refs 23, 34). Condensation of **10** with sugar acceptor **11** under Crich’s conditions (NIS, TfOH, CH_2_Cl_2_, −30 °C) did lead to the desired acetal glycoside **24**, albeit in only a moderate yield of ~30%; the major product turned out to be the α-glycoside **23** (~65%). A wide variety of the promoters for glycosidation of thioglycosides^35^ were screened in the present coupling (see Supplementary Table 1), including NBS/TfOH^36^, Tf_2_O/BSP/TTBP^37^, DMTST/TTBP^38^, MeOTf^39^ and NIS/AgOTf^40^. However, the yield of acetal glycoside **24** was not improved. These results indicate that the transient glycosyloxymethyl cation (generated from **10**) decomposes favourably to the glycosyl oxocarbenium species (and a molecule of formaldehyde); capture of the oxocarbenium intermediate by the incoming hydroxyl group (in **11**) leads to disaccharide **23**. Adjustment of the substituting groups in donor **10**, that is, removal of the 6-bromide and/or replacement of the 4-*O*-benzoyl group with benzyl group also failed to improve the yields of the corresponding acetal glycosides in condensation with **11**. Gratifyingly, when we applied the inverse procedure^41^ (addition of donor **10** to a mixture of the acceptor **11** and promoter NIS/TfOH at −30 °C) and increased the equivalent of acceptor **11** (to 5.0 equiv), acetal glycoside **24** was obtained in a satisfactory 75% yield. The 4-*O*-acetyl and 4′-*O*-benzoyl group in **24** were removed simultaneously in the presence of MeONa in MeOH, providing diol **9** (93%), which was ready for Sinaÿ’s orthoester formation.

Thus, 2′-seleno-disaccharide **9** was subjected to oxidation (NaIO_4_, NaHCO_3_, MeOH/CH_2_Cl_2_/H_2_O, RT) to provide the selenoxide cleanly, subsequent *syn*-elimination and intramolecular cyclization of the resultant ketene acetal (**A**) took place sluggishly under forced conditions (vinyl acetate/toluene/DIPA, 140 °C), leading to an orthoester product (**25**) in a moderate 20% yield for 12 h. By carefully screening the reaction conditions, we finally managed to obtain **25** in an excellent 85% yield under microwave irradiation (145 °C, 20 min). The addition of the 4-hydroxyl group onto C1′ of the ketene acetal in **A** could proceed from both the top and the bottom faces, thus two diastereoisomers should be provided. Surprisingly, only one isomer (**25**) was isolated under various conditions. We had expected that the diastereoselectivity bestowed by the two sugar units could lead to formation of the native configuration in the natural product (opposite to that drawn in **25**). However, in the previous synthesis of the Sinaÿ-type five- and six-membered orthoesters, cyclization of the incipient ketene acetals was found to favour formation of the axial α-linkage, so as to maximize the anomeric effect^22^,^23^,^24^ or to follow the trajectory of addition onto Woerpel’s low-energy conformer of the dioxocarbenium intermediate^42^,^43^. Determination of the configuration of the quaternary C1′ in **25** was not possible by spectroscopic methods, especially without comparison to its epimer, and attempts at acquisition of a single crystal of **25** or its derivatives for X-ray diffraction analysis were unsuccessful. Therefore, this problem was not solved until completion of the total synthesis.

### Synthesis of C1′′-*epi*-periploside A (31)

Temporary protection of the 4′-OH in **25** with benzoyl group led to **26** (99%), which was subjected to selective cleavage of the anomeric MP group (Ag(DPAH)_2_, CH_3_CN, H_2_O, 90%) and subsequent formation of trifluoroacetimidate (95%) to afford the desired donor **27** (Fig. 3). Glycosylation of pregnane diol **7**, which was prepared from hydroxyandrost-5-en-17-one in three steps (see Supplementary Methods and Supplementary Fig. 59), with disaccharide imidate **27** proceeded smoothly under the catalysis of TBSOTf (0.1 equiv) in the presence of 5 Å MS at −78 °C in CH_2_Cl_2_, affording pregnane 20-*O*-disaccharide **28** in 85% (96%, b.r.s.m.) yield and β/α ratio of 3:1. The erosion of 1,2-*trans*-selectivity in the glycosylation with a donor (that is, **27**) equipped with an iodide at C2 could be contributed to the presence of the FABO motif, which might cause conformational restraint for the neighbouring participation. Notably, the present reaction must be quenched at −78 °C (with Et_3_N), so as to avoid the decomposition of the acid-labile FABO motif. Reductive removal of the bromide and iodide in **28** under the conventional radical conditions (AIBN/Bu_3_SnH, toluene, 80 °C) led to decomposition of the substrate. Et_3_B could initiate the radical reaction at RT^44^, which thus enabled the reduction of **28** with Bu_3_SnH to proceed smoothly to afford the corresponding deoxydisaccharide (99%). Subsequent removal of the 4′-*O*-benzoyl group provided the desired pregnane 20-*O*-disaccharide **29** (99%).

The expected challenge in stereoselective construction of the 2-deoxy-β-cymarosyl-(1→4)-linkage in the union of tetrasaccharide **2** and pregnane disaccharide **29** was further manifested by the fact that both the 2,6-dideoxyglycosidic linkages and the FABO motif were shown to be extremely labile toward acid. Thus, we screened carefully the coupling conditions, including the gold(I) catalyst, solvent and temperature. When the coupling of **2** and **29** was conducted in the presence of Ph_3_PAuNTf_2_ (0.2 equiv), the coupled hexasaccharide was obtained in good yield (50%) but in favour of the thermodynamically more stable α-anomer (β/α~1:3). With Ph_3_PAuOTf as the catalyst, however, the reaction of **2** and **29** provided a messy mixture. Careful isolation led to identification of a pentasaccharide lactone, which was derived from cleavage of the FABO linkage in the coupled hexasaccharide (**30**). Encouragingly, the originally formed cymarosyl-(1→4)-linkage in the resultant pentasaccharide lactone was found in the required β configuration, implying that the glycosylation reaction took place via a glycosyl α-triflate intermediate^18^. Nevertheless, the HOTf generated after glycosylation, before being quenched by the isochromen-4-yl gold(I) complex^45^, degraded the FABO linkage. On the basis of this rational, we introduced a hindered base, that is, 2,4,6-tri-*tert*-butylpyrimidine (TTBP), into the present Ph_3_PAuOTf-catalysed glycosylation to rest the incipient HOTf, so as to avoid the cleavage of the FABO linkage. The sequester of the HOTf also retarded the gold(I) catalytic cycle, therefore, requiring almost equivalent of the gold complex to drive the reaction to completion^45^,^46^. In fact, the condensation of **2** and **29** proceeded smoothly in the presence of Ph_3_PAuOTf (0.8 equiv) and TTBP (1.5 equiv) in CH_2_Cl_2_ at −10 °C, leading to the coupled hexasaccharide **30** in a satisfactory yield of 64% (87%, b.r.s.m.) and β/α ratio of 2.1:1. Finally, removal of the terminal CA group and TBS group was achieved with thiourea^47^ and pyridine-buffered HF·pyridine, respectively, furnishing the target pregnane hexasaccharide **31** cleanly (91%, two steps).

The ^1^H and ^13^C NMR spectra of **31** are similar to those of the authentic periploside A (**1**) (see Supplementary Figs 60 and 61). However, discrepancies occur for the signals from the formyl acetyl CH_2_ residue (88.4 p.p.m. for the ^13^C signal in **31** versus 86.4 p.p.m. in **1**; 4.93 and 4.82 p.p.m. for the ^1^H signals in **31** versus 5.13 and 4.74 p.p.m. in **1**) (see Supplementary Table 3). In addition, the adjacent C4′, C2′′ and C3′′ are also in disparate chemical shifts (C4′: 81.5 versus 79.2 p.p.m.; C2′′: 37.9 versus 36.7 p.p.m.; C3′′: 77.4 versus 78.3 p.p.m., in **31** and **1**, respectively). These discrepancies indicate that the synthetic **31** is indeed the epimer of periploside A (**1**) with the opposite configuration at the quaternary C1′′. The problem of diastereoselectivity in the previous formation of the FABO motif is thus addressed.

### Construction of the FABO motif of the natural configuration

The addition of the hydroxyl group onto the ketene acetal in **A** proceeded exclusively from the α face, leading to the FABO disaccharide with the unnatural configuration (**9**→**25**). The presence of a nucleophile capable of interception of the oxocarbenium species developed from the ketene acetal might lead to a thermodynamically favored α-intermediate; substitution of the intermediate *in situ* by the 4-hydroxyl group would then proceed from the β face to provide the FABO disaccharide with the natural configuration. Therefore, we tried the elimination/cyclization (from **9**) in various nucleophilic solvents, including CH_3_CN, 1,4-dioxane, THF, DME, DMF and MeOH, under varied temperatures; however, the only FABO disaccharide identified was **25**. Addition of nucleophilic additives, such as 4-dimethylaminopyridine (DMAP), LiBr, NaBr and NaI, was also found futile. The presence of an electrophilic reagent, such as NIS, NBS and I_2_, might convert the ketene acetal into a 1,2-halonium intermediate, which could then be attacked by the 4-hydroxyl group to give the FABO disaccharide. These attempts were again unsuccessful.

**Figure 4:**
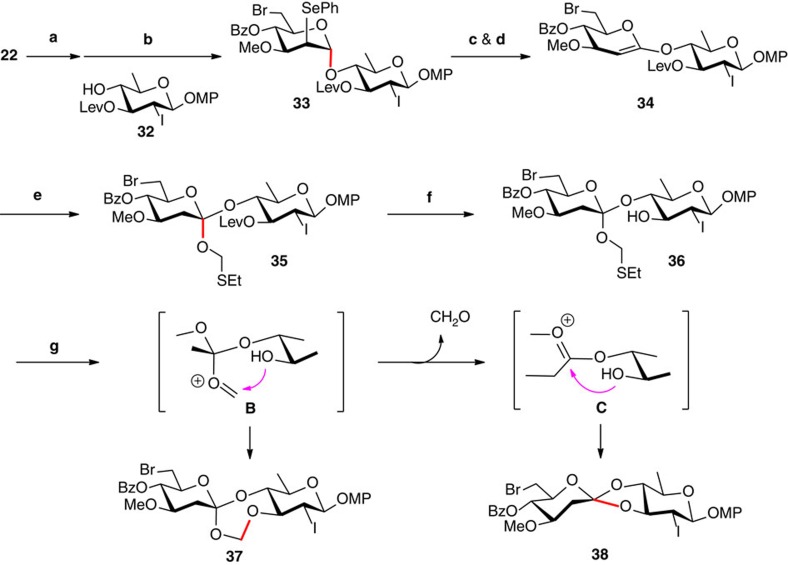
Construction of the FABO motif with the natural configuration. Highlighted in red are the nascent glycosidic bonds. (**a**) DAST, THF, −30 °C to RT; (**b**) SnCl_2_, Et_2_O, 4 Å MS, 0 °C to RT; 85% (for two steps); (**c**) NaIO_4_, NaHCO_3_, MeOH, CH_2_Cl_2_, H_2_O, RT, 99%; (**d**) vinyl acetate, toluene, DIPA, Mw, 140 °C, 40 min, 92%; (**e**) EtSCH_2_OH, DCCl_3_, Mw, 10 min, 110 °C, 81%; (**f**) H_2_NNH_2_·H_2_O, pyridine, HOAc, 0 °C to RT, 92%; (**g**) BSP, Tf_2_O, DTBP, 5 Å MS, Et_2_O, −114 °C, 64% (for **37**), 18% (for **38**). BSP, 1-benzenesulfinyl piperidine; DTBP, 2,6-di-*tert*-butylpyridine; Lev, levuloyl; Mw, microwave.

The failure in formation of the seven-membered orthoester in the desired stereochemistry forced us to construct the orthoester before cyclization of the formyl acetal (Fig. 4). Thus, 2-phenylseleno-D-mannopyranoside **22** was converted into the fluoride (with DAST), which was then coupled with sugar acceptor **32** (see Supplementary Methods) under the action of SnCl_2_ to give α-disaccharide **33** (85%). Gratifyingly, after intensive attempts, we managed to obtain the ketene acetal **34** in excellent yield (91%) from disaccharide **33** via selenoxide formation and *syn*-elimination under the modified Sinaÿ conditions (NaIO_4_, NaHCO_3_, MeOH/CH_2_Cl_2_/H_2_O, RT; then vinyl acetate/toluene/DIPA, microwave 140 °C, 40 min). Although purification of **34** on silica gel required the addition of 1% Et_3_N in the eluent to prevent hydrolysis, the purified ketene acetal **34** remained stable at −20 °C for several days. To the best of our knowledge, this is the first time a ketene acetal linked disaccharide (that is, **34**) being isolated. Noteworthy is the decisive role played by the 3-*O*-Lev group (in **33**–**35**), which facilitated the procurement of the ketene acetal disaccharide as well as the selective removal afterwards; in contrast, analogues of **33** bearing an electron-donating group (for example, TBS) at the 3-OH led to decomposed monosaccharide derivatives under identical conditions.

Addition of PhSCH_2_OH onto ketene acetal **34** was first attempted in CDCl_3_ at 50 °C (ref. 48), the starting ketene acetal decomposed largely in 12 h, with the desired orthoester being isolated in low yield (20%) as a single α-isomer. By raising the temperature and shortening the reaction duration (CDCl_3_, microwave 100 °C, 1 h), the addition of **34** and PhSCH_2_OH led to the desired orthoester in a good 70% yield but in a moderate diastereoselectivity of 1.4/1. Modification of the reaction conditions by the addition of acidic promoters (*p*-TsOH or PPTS) or variation of solvent (toluene, CH_2_Cl_2_ or CHCl_3_) failed to improve the yield or the diastereoselectivity. Fortunately, replacement of PhSCH_2_OH with the more nucleophilic EtSCH_2_OH, the addition with ketene acetal **34** under the optimal conditions (CDCl_3_, microwave 110 °C, 10 min) furnished the desired orthoester **35** in 81% yield as a single α-isomer. It should be noted that the replacement of CDCl_3_ with CHCl_3_ or toluene led to lower yields of **35** (<65%). Selective removal of the 3-*O*-Lev group in **35** was achieved with H_2_NNH_2_·H_2_O to provide orthoester **36** (92%).

Treatment of ethylthiomethyl glycoside **36** with the mild promoters for glycosidation of thioglycosides (see Supplementary Table 2), such as MeOTf/TTBP, DMTST/TTBP, and CuBr_2_/Bu_4_NBr^49^, failed to afford the seven-membered FABO derivative **37**; instead, the five-membered orthoester **38** was isolated in high yield (>80%) as a mixture of the two diastereoisomers. These results indicate again (*cf.*, **10**→**23**/**24**) that the glycosyloxymethyl cation (**B**) generated via activation of the thioacetal could readily undergo decomposition to the glycosyl oxocarbenium (**C**) and formaldehyde; intramolecular addition of the proximal hydroxyl group to **C** led to the five-membered orthoester. Thus, a strong promoter which can activate the thioacetal under milder conditions might be able to allow the transient glycosyloxymethyl cation **B** to be captured by the hydroxyl group before decomposition. Using Crich’s conditions (Tf_2_O/BSP/TTBP, −60 °C, CH_2_Cl_2_)^37^, however, the five-membered orthoester **38** was again obtained nearly quantitatively as a mixture of the diastereoisomers. Encouragingly, when lowering the reaction temperature to −78 °C, a trace amount of the desired seven-membered FABO product **37** was detected. Optimizing along this direction, we finally managed to obtain the desired **37** in a satisfactory 64% yield in the presence of Tf_2_O/BSP/DTBP in Et_2_O at −114 °C, whereas the five-membered orthoester **38** was isolated in 18% yield as a single diastereoisomer. Comparison of the NMR data of the present FABO disaccharide **37** with those of the previous **26** supported that we had fixed the desired configuration by the present approach.

### Completion of the synthesis of periploside A (1)

**Figure 5:**
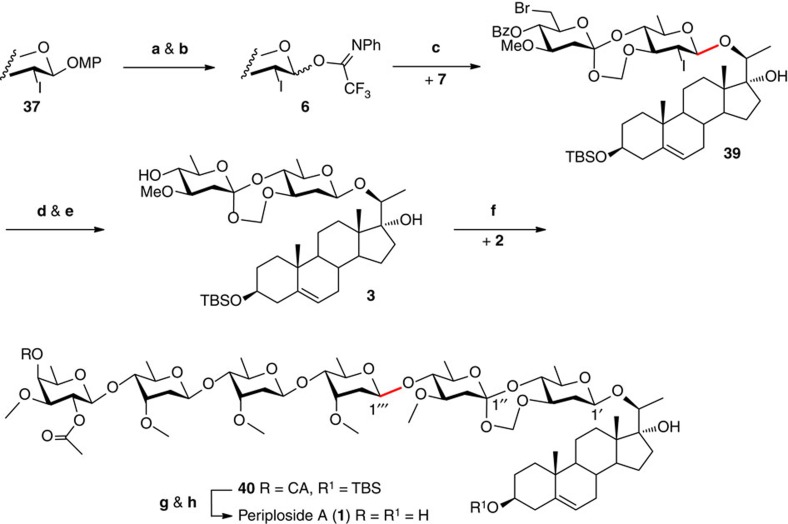
Completion of the total synthesis of periploside A (1). Highlighted in red are the nascent glycosidic bonds. (**a**) Ag(DPAH)_2_, CH_3_CN, H_2_O, 0 °C, 91%; (**b**) *N*-phenyl-trifluoroacetimidoyl chloride, Cs_2_CO_3_, CH_2_Cl_2_, RT, 90%; (**c**) TBSOTf, CH_2_Cl_2_, 5 Å MS, −78 °C, 87%, β/α=6.1/1; (**d**) Et_3_B, Bu_3_SnH, toluene, RT, 95%; (**e**) MeONa, MeOH, CH_2_Cl_2_, RT, 96%; (**f**) Ph_3_PAuOTf (0.8 equiv), TTBP, CH_2_Cl_2_, 4 Å MS, −10 °C, 80%, β/α=2/1; (**g**) thiourea, pyridine, EtOH, 80 °C; (**h**) HF·py, pyridine, THF, 0 °C to RT, 93% (for two steps).

The transformations previously developed for the synthesis of C1′′-*epi*-periploside A (**26**→**31**, eight steps, 28% overall yield) were applied to the synthesis of periploside A (Fig. 5). Thus, FABO disaccharide **37** was subjected to selective cleavage of the anomeric MP group (with Ag(DPAH)_2_, 91%) and conversion into the trifluoroacetimidate donor **6** (90%). Coupling of pregnane diol **7** with donor **6** under the similar conditions as that for **7**+**27**→**28** (85%, β/α=3:1) led to the corresponding disaccharide **39** in a similar yield (87%) but much higher β selectivity (β/α=6.1:1). This result supports the previous assumption that the conformational restraint provided by the FABO motif affects the glycosidation transition state of the disaccharide. The bromide and iodide in **39** were removed cleanly with Et_3_B/Bu_3_SnH (95%); subsequent cleavage of the 4′-*O*-benzoyl group (NaOMe, HOMe, 96%) provided the desired disaccharide acceptor **3**. Condensation of **3** with tetrasaccharide donor **2** under the optimized conditions for **29**+**2**→**30** (64%, β/α=2.1:1) afforded hexasaccharide **40** in a higher 80% (92%, b.r.s.m.) yield and similar β/α ratio of 2:1. Finally, the terminal CA and TBS groups were removed successfully with thiourea and pyridine-buffered HF-pyridine, respectively, furnishing the target periploside A (**1**) (93%, two steps). Gratifyingly, all the analytical data of **1** are in full agreement with those obtained for the natural product^1^ (see Supplementary Table 3).

### The immunosuppressive activities of the synthetic compounds

**Figure 6:**
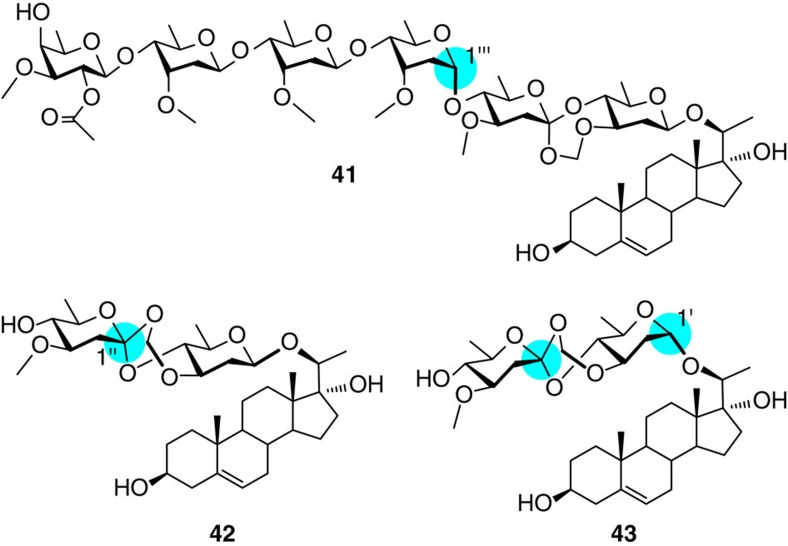
Synthetic analogues of periplosides. Shadowed in pale blue are the carbon centres with opposite configurations to those in the nature product.

The synthetic periploside A (**1**) showed similar toxicity and activity as the natural product against T-lymphocyte and their proliferation induced by ConA^5^,^6^. The C1′′-epimer (**31**) which has the opposite configuration at the FABO motif as in periploside A was also found active (IC_50_=0.41 μM), although being ~2.4-fold less potent than the natural product (Table 1). The C1′′′-epimer of periploside A (**41**) was also active (IC_50_=1.96 μM) (Fig. 6). Interestingly, disaccharide **42**, with the tetrasaccharide in **31** being truncated, still had certain activity (IC_50_=6.20 μM); and its α-anomer **43** even showed a higher activity (IC_50_=3.70 μM). Previous assays have demonstrated that reductive cleavage of the formyl acetal in the FABO motif in periploside A abolished the activity completely^6^. Taken together, it demonstrates that the chemical connection of the FABO motif, rather than the stereochemistry, has a critical role in the immunosuppressive activities of periplosides. The capability of releasing a formylaldehyde might be taken into account in the future researches into the mechanism of action of this unique type of compounds.

## Methods

### General methods

All the reactions were carried out under nitrogen or argon with anhydrous solvents in flame-dried glassware, unless otherwise noted. All the glycosylation reactions were performed in the presence of 4 or 5 Å MS, which were flame-dried immediately before use in the reaction under high vacuum. Glycosylation solvents were dried using a solvent purification system and used directly without further drying. The chemicals used were reagent grade as supplied, except where noted. For details of the synthetic procedures and the characterization data of compounds, see Supplementary Methods. For ^1^H and ^13^C NMR spectra of the compounds prepared in this study, see Supplementary Figs 1–58.

### Synthesis of acetal glycoside **24**

To a solution of sugar alcohol **11** (620 mg, 1.47 mmol) in CH_2_Cl_2_ (5 ml) was added acid-washed 3 Å MS at RT. After stirring for 30 min at RT, NIS (142 mg, 0.63 mmol) and TfOH (2.6 μl, 0.029 mmol) were added to the mixture at −30 °C, followed by the addition of a solution of phenylthiomethyl glycoside **10** (183 mg, 0.29 mmol) in CH_2_Cl_2_ (3 ml) via a syringe pump. The reaction mixture was stirred at −30 °C for 1 h. When TLC showed the donor **10** had been consumed, saturated aqueous NaHCO_3_ was added at 0 °C. The mixture was filtered. The filtrate was washed with a solution of NaS_2_O_3_ and brine, respectively, and was then dried over Na_2_SO_4_ and concentrated. The residue was purified by flash chromatography (petroleum ether/CH_2_Cl_2_/EtOAc=8:1:1) to afford **24** (206 mg, 75%) and glycoside **23** (53 mg, 20%) as syrups.

### Synthesis of FABO disaccharide **25**

To a solution of acetal glycoside **9** (187 mg, 0.24 mmol) in MeOH/CH_2_Cl_2_/H_2_O (3 ml/2 ml/1 ml) were added NaIO_4_ (507 mg, 2.37 mmol) and NaHCO_3_ (159 mg, 1.90 mmol) at RT. After stirring for 3 h, the mixture was diluted with CH_2_Cl_2_, washed with saturated NH_4_Cl solution and brine, respectively, and was then dried over Na_2_SO_4_ and concentrated. The resultant selenoxide was azeotroped with toluene (3 × 5 ml) and dried under high vacuum for 2 h to afford a white solid (190 mg, 99%). The above selenoxide (125 mg, 0.155 mmol) was dissolved in toluene (6 ml). Diisopropylamine (3 ml) and vinyl acetate (6 ml) were added, and the reaction was conducted under microwave at 145 °C for 20 min. The mixture was cooled to RT and concentrated. The residue was purified by flash chromatography on silica gel (petroleum ether/EtOAc=1.5:1) to afford **25** (83 mg, 85%) as a colourless syrup.

### Synthesis of α-disaccharide **33**

To a solution of lactol **22** (603 mg, 1.21 mmol) in THF (10 ml) was added DAST (0.44 ml, 3.63 mmol) at −30 °C. After stirring for 1.5 h while warming to RT, a saturated NaHCO_3_ solution was added slowly to the mixture. The resulting mixture was extracted with CH_2_Cl_2_. The combined organic layer was washed with brine, dried over Na_2_SO_4_ and concentrated. The crude glycosyl fluoride was azeotroped with toluene (3 × 5 ml). After drying under high vacuum for 2 h, the above product was dissolved in Et_2_O (10 ml), 4 Å MS (1.2 g) and sugar alcohol **32** (330 mg, 0.69 mmol) were added and the reaction mixture was stirred at 0 °C for 30 min. SnCl_2_ (235 mg, 1.24 mmol) was added in one portion and the reaction mixture was allowed to warm to RT and stirred for 4 h. The mixture was quenched with Et_3_N (1 ml) and filtered. The solution was diluted with EtOAc and washed with water. The water layer was extracted with EtOAc twice. The combined organic layer was washed with saturated NaHCO_3_ solution and brine, respectively, and was then dried over Na_2_SO_4_ and concentrated. The residue was purified by flash chromatography (petroleum ether/EtOAc=4:1) to afford **33** (560 mg, 85%) as a colourless syrup.

### Synthesis of ketene acetal **34**

To a solution of **33** (248 mg, 0.26 mmol) in MeOH/CH_2_Cl_2_/H_2_O (3 ml/2 ml/1 ml) were added NaIO_4_ (552 mg, 2.58 mmol) and NaHCO_3_ (173 mg, 2.06 mmol) at RT. After stirring for 12 h at RT, the mixture was diluted with CH_2_Cl_2_, and washed with saturated NH_4_Cl solution and brine, respectively. The organic layer was dried over Na_2_SO_4_ and concentrated. The crude selenoxide was azeotroped with toluene (3 × 5 ml) and dried under high vacuum for 2 h to afford a colourless syrup (251 mg, 99%). The above selenoxide (104 mg, 0.11 mmol) was dissolved in toluene (2 ml). Diisopropylamine (1 ml) and vinyl acetate (2 ml) were added. The reaction was conducted under microwave at 140 °C for 40 min. The mixture was cooled to RT and concentrated. The residue was purified by flash chromatography (petroleum ether/EtOAc=5:1, containing 1% Et_3_N) to afford **34** (85 mg, 92%) as a colourless syrup.

### Synthesis of orthoester **35**

To a solution of **34** (75 mg, 0.093 mmol) in CDCl_3_ (3 ml) was added EtSCH_2_OH (0.05 ml) at RT. The reaction was conducted under microwave at 110 °C for 10 min. The mixture was cooled to RT and concentrated. The residue was purified by flash chromatography (petroleum ether/EtOAc=5:1, containing 1% Et_3_N) to afford **35** (67 mg, 81%) as a colourless syrup.

### Synthesis of orthoester **36**

To a solution of **35** (110 mg, 0.120 mmol) in pyridine/HOAc (3 ml/2 ml) was added H_2_NNH_2_ H_2_O (0.10 ml, 1.60 mmol) at 0 °C. After stirring at RT for 5 h, the mixture was diluted with CH_2_Cl_2_, and washed with ice water and then with a saturated NaHCO_3_ solution and brine, respectively. The organic layer was dried over Na_2_SO_4_ and concentrated. The residue was purified by flash chromatography (petroleum ether/EtOAc=5:1, containing 1% Et_3_N) to afford **36** (90 mg, 92%) as a colourless syrup.

### Synthesis of FABO disaccharide **37**

To a solution of **36** (19.1 mg, 0.024 mmol) in Et_2_O (3 ml) were added BSP (8.2 mg, 0.036 mmol), 2,6-di-*tert*-butylpyridine (16.0 μl, 0.072 mmol) and 5 Å MS at RT. After stirring at −114 °C (liq. N_2_-EtOH) for 20 min, Tf_2_O (6.0 μl, 0.036 mmol) was added to the mixture. The mixture was stirred at −114 °C for 1 h, and was then warmed to RT and filtered. The filtrate was washed with a saturated aqueous NaHCO_3_ solution and brine, respectively, and was then dried over Na_2_SO_4_ and concentrated. The residue was purified by flash chromatography on silica gel (petroleum ether/EtOAc=5:1) to afford **37** (11.3 mg, 64%) and the five-membered orthoester **38** (3.0 mg, 18%) as colourless syrups.

### Synthesis of FABO disaccharide trifluoroacetimidate **6**

To a solution of *p*-methoxyphenyl glycoside **37** (36 mg, 0.049 mmol) in CH_3_CN/H_2_O (3 ml/3 ml) was added Ag(DPAH)_2_ (79 mg, 0.17 mmol) at 0 °C. After stirring for 30 min at this temperature, the mixture was filtered. The filtrate was diluted with CH_2_Cl_2_, washed with saturated NaHCO_3_ solution and brine, respectively, and was then dried over Na_2_SO_4_ and concentrated. The residue was purified by flash chromatography (petroleum ether/EtOAc=3:1) to yield the corresponding hemiacetal (28 mg, 91%) as a colourless syrup. To a solution of the hemiacetal (29 mg, 0.046 mmol) in CH_2_Cl_2_ (3 ml) were added Cs_2_CO_3_ (75 mg, 0.23 mmol) and *N*-phenyl-2,2,2-trifluoroacetimidoyl chloride (14 μl, 0.14 mmol) at RT. After stirring for 3 h, the mixture was filtered. The filtrate was evaporated in vacuo to give a residue, which was subjected to chromatography on Davisil silica (pH=7.0, petroleum ether/EtOAc, 5:1) to give **6** (33 mg, 90%) as a colourless syrup. This compound was used directly without further characterization.

### Synthesis of pregnane β-disaccharide **39**

To a solution of imidate **6** (33.0 mg, 0.041 mmol) and pregnane diol **7** (15.6 mg, 0.035 mmol) in CH_2_Cl_2_ (3 ml) was added 5 Å MS at RT. After stirring at −78 °C for 30 min, TBSOTf (1.2 μl, 0.0052, mmol) was added to the mixture. After stirring for 6 h at this temperature, Et_3_N was added to quench the reaction. The resulting mixture was filtered. The filtrate was evaporated in vacuo to give a residue, which was purified by flash chromatography (petroleum ether/CH_2_Cl_2_/EtOAc=10:5:1) to afford **39** (27.6 mg, 75%) and its α-anomer (4.5 mg, 12%) as white solids.

### Synthesis of pregnane disaccharide 3

To a solution of **39** (21.0 mg, 0.020 mmol) in toluene (2 ml) were added Bu_3_SnH (32 μl, 0.12 mmol) and Et_3_B (12 μl, 0.012 mmol) at 0 °C. After stirring for 1 h at RT, the mixture was concentrated in vacuo. The residue was purified by flash chromatography (petroleum ether/EtOAc=5:1) to afford the corresponding 2′,6′′-deoxy derivative (16.5 mg, 97%) as a white solid (see Supplementary Methods). To a solution of this compound (16.5 mg, 0.019 mmol) in CH_2_Cl_2_/MeOH (1.5 ml/1.5 ml) was added NaOMe (20 mg, 0.37 mmol) at RT. After stirring for 40 h, the mixture was filtered through silica gel. The filtrate was evaporated in vacuo to give a residue, which was purified by flash chromatography (petroleum ether/EtOAc=2:1) to afford **3** (14.0 mg, 97%) as a white solid.

### Synthesis of pregnane hexasaccharide **40**

To a solution of tetrasaccharide *ortho*-cyclopropylethynylbenzoate **2** (22.3 mg, 0.025 mmol), pregnane disaccharide **3** (8.0 mg, 0.011 mmol) and 2,4,6-tri-*tert*-butylpyrimidine (TTBP) (4.0 mg, 0.016 mmol) in CH_2_Cl_2_ (2 ml) was added 4 Å MS at RT. After stirring for 30 min at −20 °C, a solution of PPh_3_AuOTf in CH_2_Cl_2_ (0.05 ml, 0.1 M) was added to the mixture. The mixture was stirred for 2 h while warming to −10 °C, then another portion of PPh_3_AuOTf in CH_2_Cl_2_ (0.05 ml, 0.1 M) was added to the mixture. After stirring for 4 h at −10 °C, Et_3_N was added to quench the reaction. The resulting mixture was filtered and concentrated. The residue was purified by flash chromatography (petroleum ether/CH_2_Cl_2_/EtOAc=1:1:1) to afford **40** (8.3 mg, 53%), its α-anomer (4.2 mg, 27%) as white foams, and recovered **3** (1.0 mg, 13%).

### Synthesis of Periploside A **1**

To a solution of **40** (4.7 mg, 3.2 μmol) in pyridine/EtOH (1.0 ml/1.0 ml) was added thiourea (10 mg, 0.13 mmol) at RT. After stirring for 2 h at 80 °C, the mixture was concentrated in vacuo to give a residue, which was purified by flash chromatography (CHCl_3_/MeOH=30:1) to afford a colourless syrup. The syrup was dissolved in THF/pyridine (1.5 ml/0.75 ml). HF-py (70% HF in pyridine, 0.10 ml) was added dropwise at 0 °C. After stirring for 40 h at RT, a saturated NaHCO_3_ solution was added slowly to the mixture at RT. The resulting mixture was diluted with CH_2_Cl_2_, washed with saturated NaHCO_3_ solution and was then extracted with CH_2_Cl_2_ twice. The combined organic layer was washed with brine, dried over Na_2_SO_4_ and concentrated. The residue was purified by flash chromatography (CHCl_3_/MeOH=30:1) to afford periploside A (**1**) (3.8 mg, 93%) as a white foam.

## Author contributions

X.Z. and B.Y. conceived the synthetic route. X.Z. conducted the synthetic work. Y.Z. and J.Z. conducted bioassay. B.Y. and X.Z. wrote the manuscript.

## Additional information

**How to cite this article:** Zhang, X. *et al.* Total synthesis of periploside A, a unique pregnane hexasaccharide with potent immunosuppressive effects. *Nat. Commun.* 6:5879 doi: 10.1038/ncomms6879 (2015).

## Supplementary Material

Supplementary Information Supplementary Figures 1-63, Supplementary Tables 1-3, Supplementary Methods and Supplementary References

## Figures and Tables

**Table 1:** Inhibitory activity of periploside A and analogues against T-lymphocyte proliferation*.

**Compound**	**CC**_**50**_ **(μM)**	**IC**_**50**_ **(μM)**	**SI**
Periploside A (**1**)	2.10±0.32	0.17±0.04	12
C1′′-*epi*-periploside A (**31**)	1.58±0.19	0.41±0.15	3.9
C1′′′-*epi*-periploside A (**41**)	11.40±3.66	1.96±0.43	5.8
Disaccharide **42**	12.80±0.52	6.20±3.20	2.1
Disaccharide **43**	27.70±6.61	3.70±2.25	7.5

^*^See Supplementary Methods and Supplementary Figs 62 and 63 for details.
