# Supplementary material for: Total synthesis of periploside A, a unique pregnane hexasaccharide with potent immunosuppressive effects
Source: Nat Commun. 2015 Jan 20;6:5879. doi: 10.1038/ncomms6879 (PMC4309423; doi:10.1038/ncomms6879)
Supplement: Supplementary Information — Supplementary Figures 1-63, Supplementary Tables 1-3, Supplementary Methods and Supplementary References [file ncomms6879-s1.pdf]

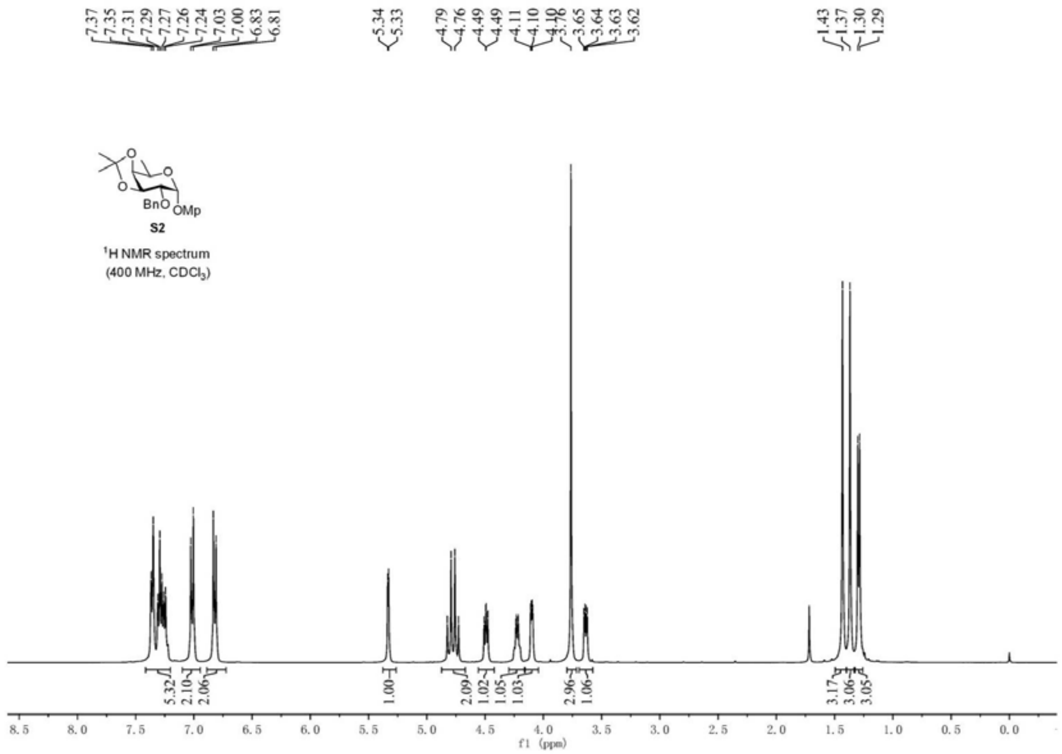

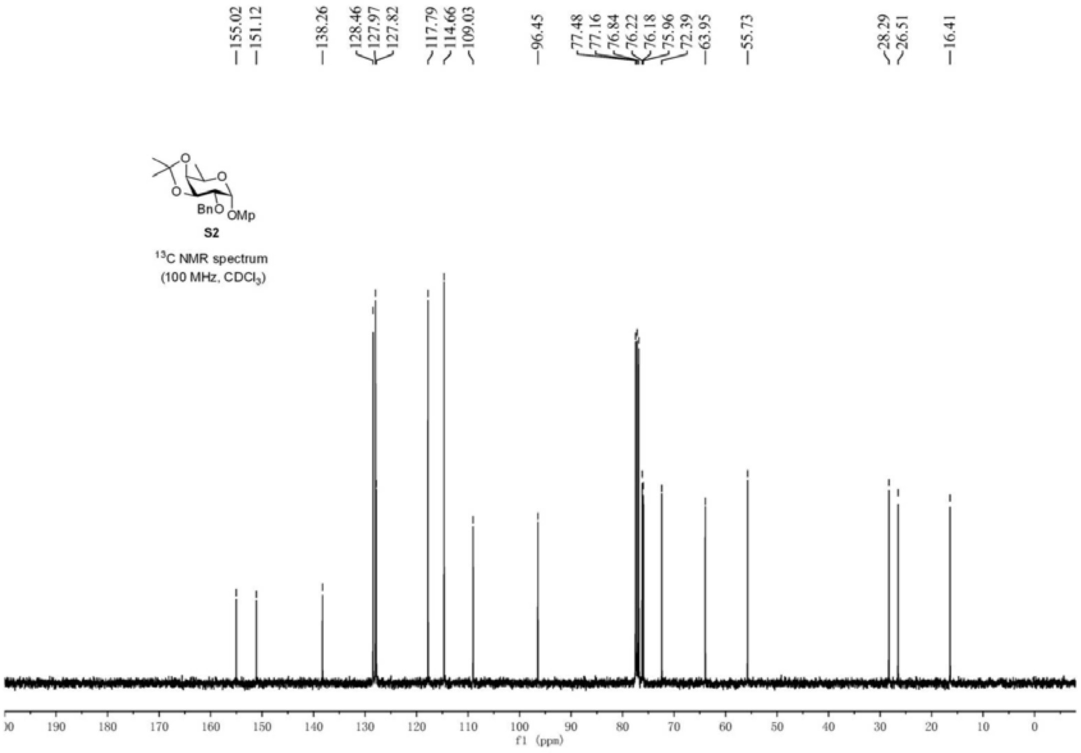

Supplementary Figure 1. ^{1}H and ^{13}C NMR spectra for compound S2.

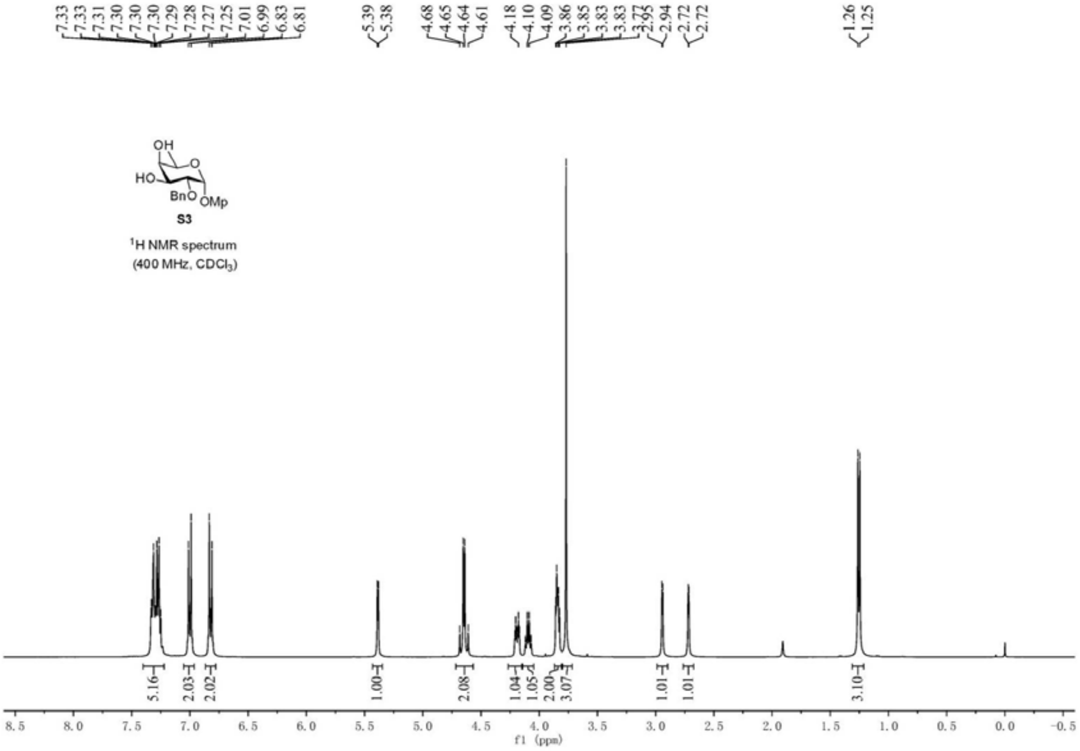

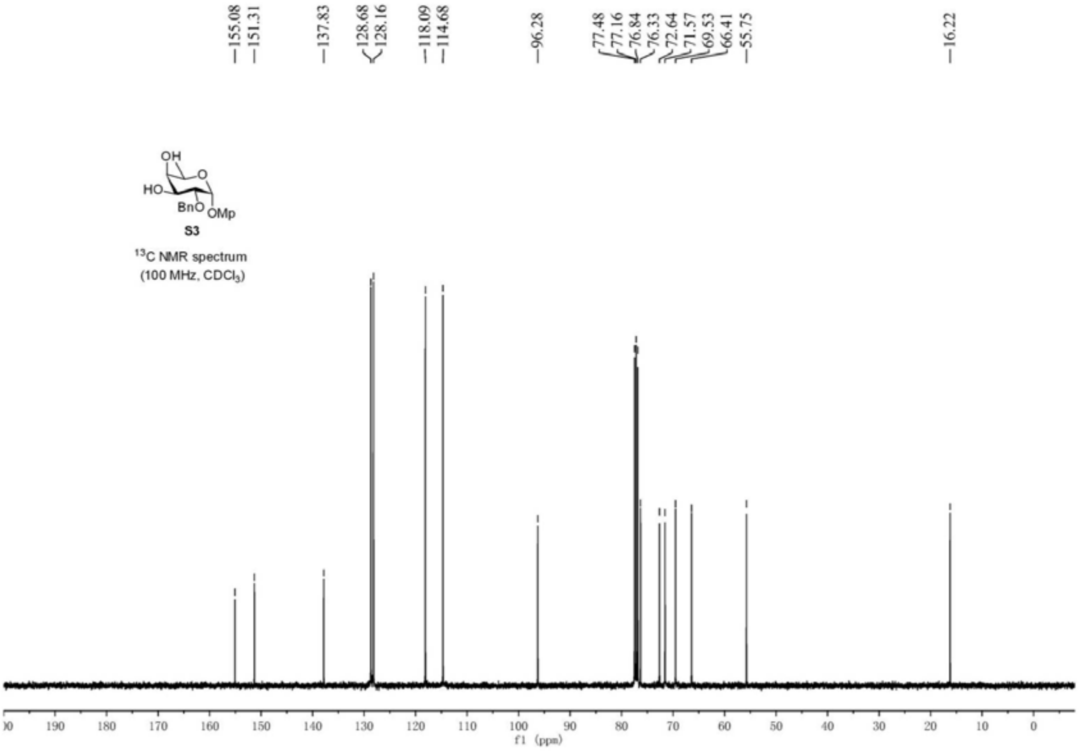

Supplementary Figure 2. ^{1}H and ^{13}C NMR spectra for compound S3.

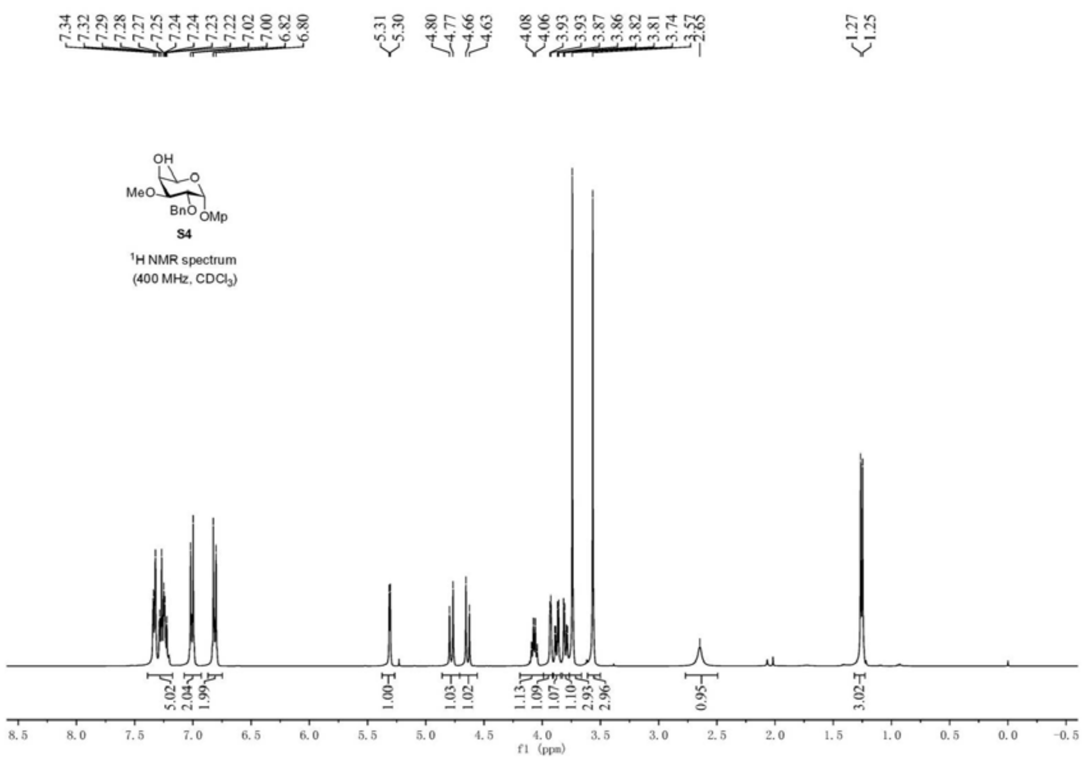

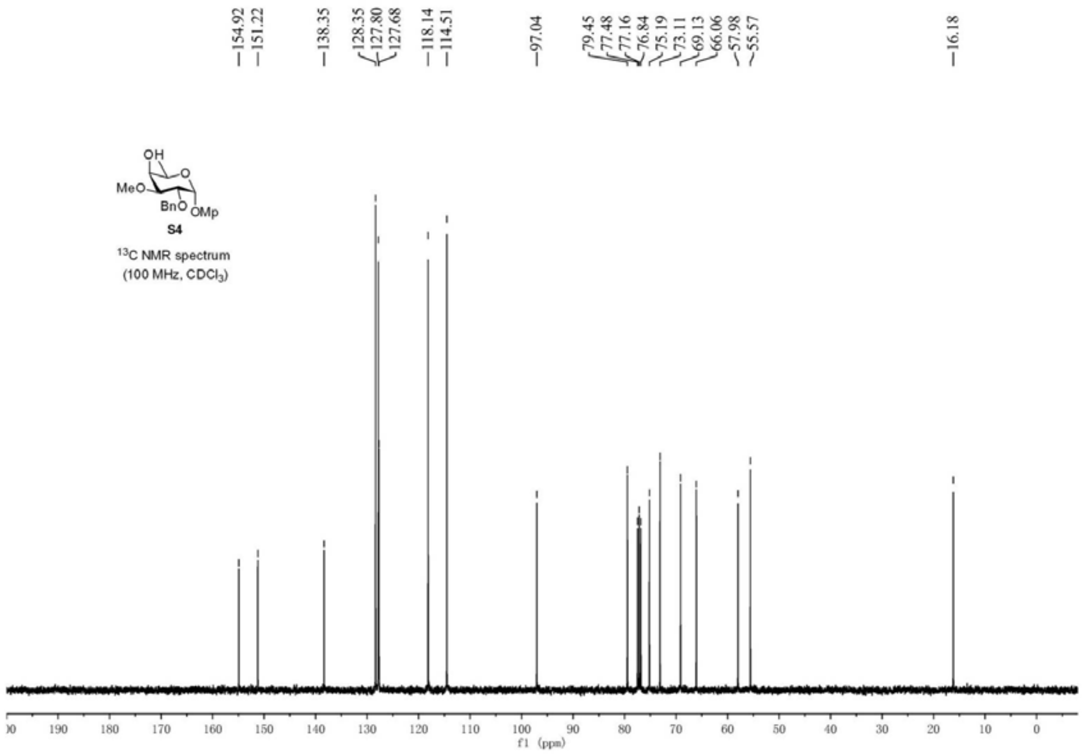

Supplementary Figure 3. ^{1}H and ^{13}C NMR spectra for compound S4.

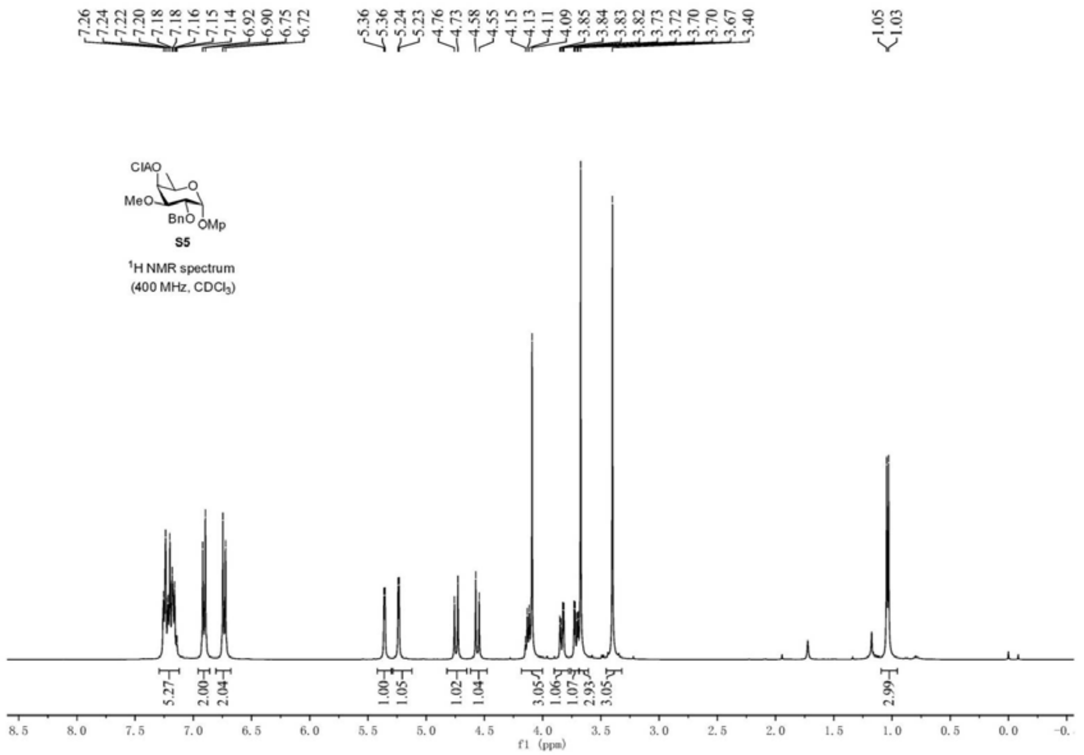

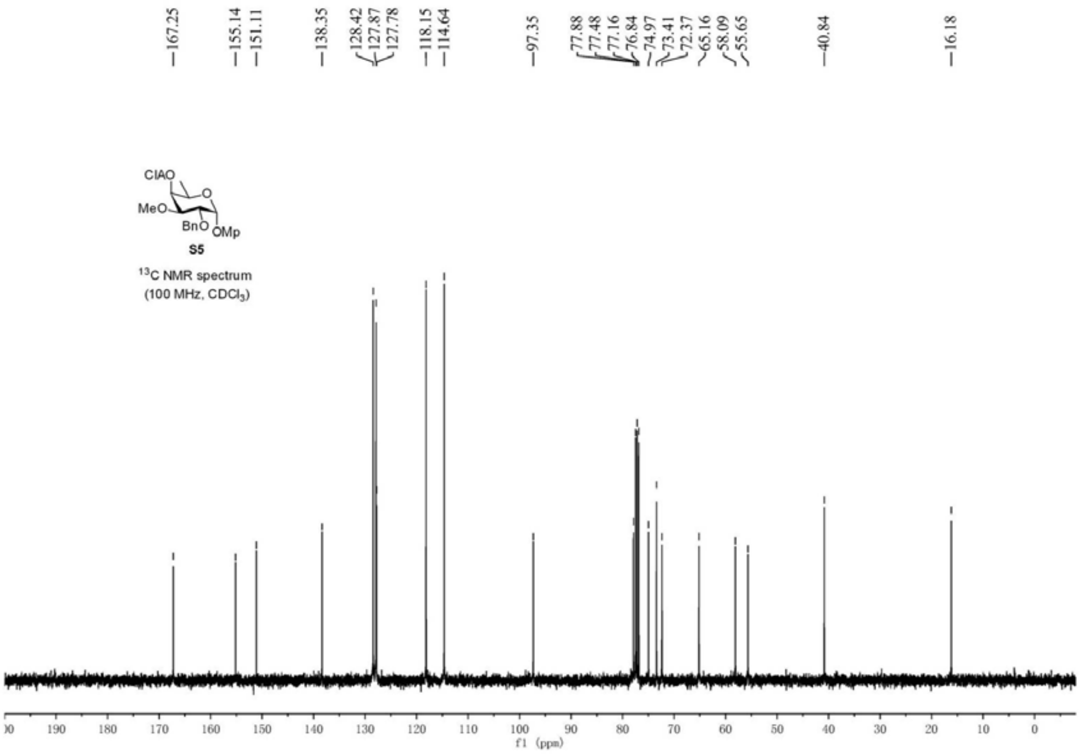

Supplementary Figure 4. ^{1}H and ^{13}C NMR spectra for compound S5.

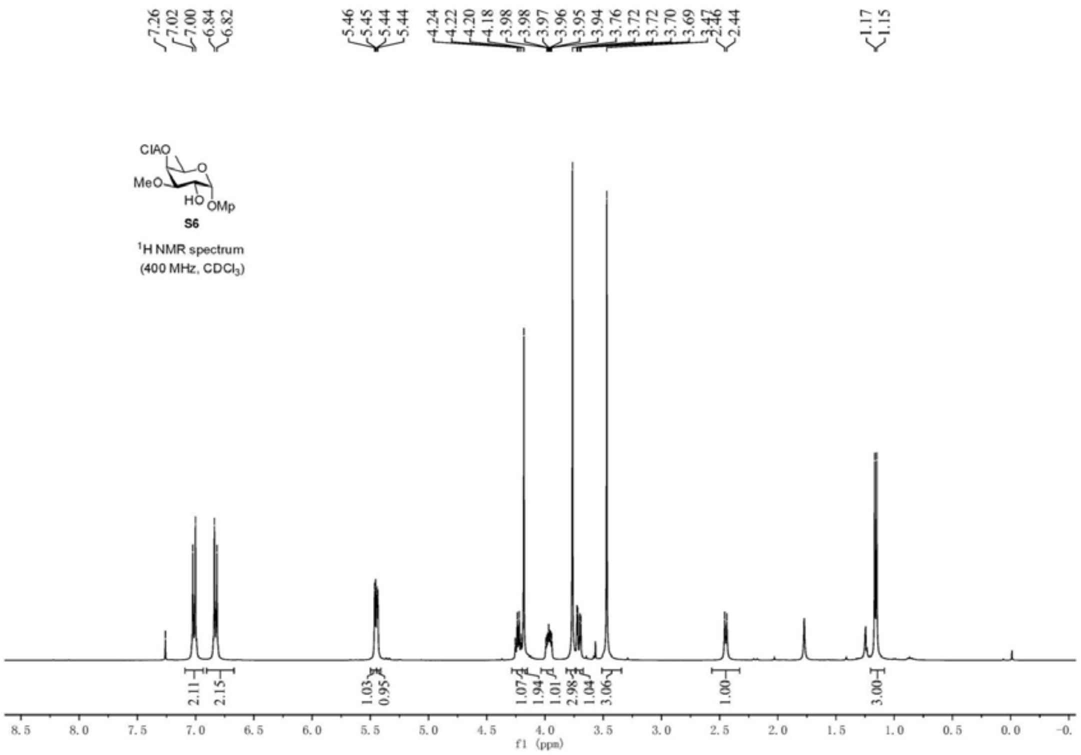

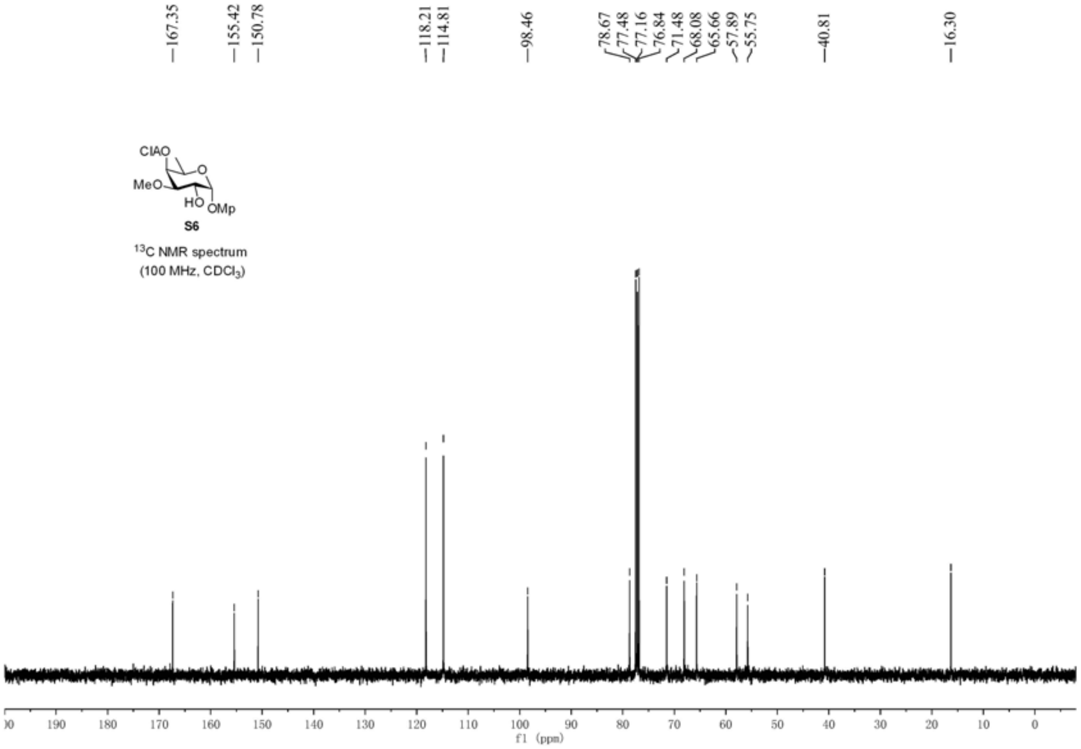

Supplementary Figure 5. ^{1}H and ^{13}C NMR spectra for compound S6.

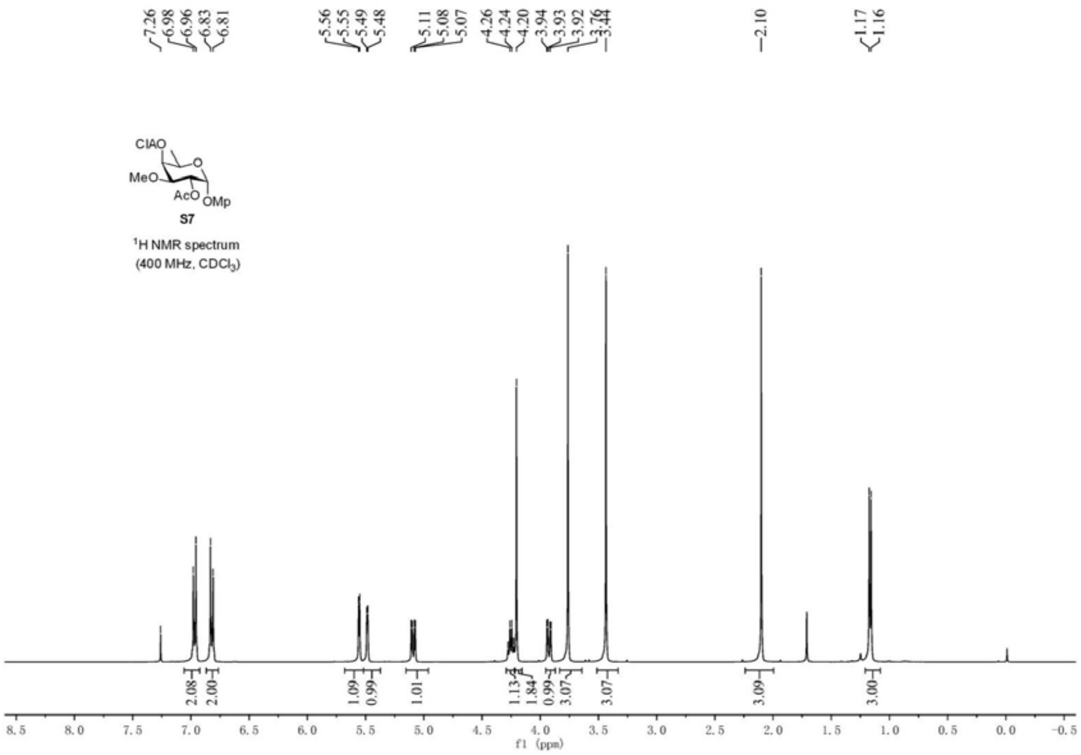

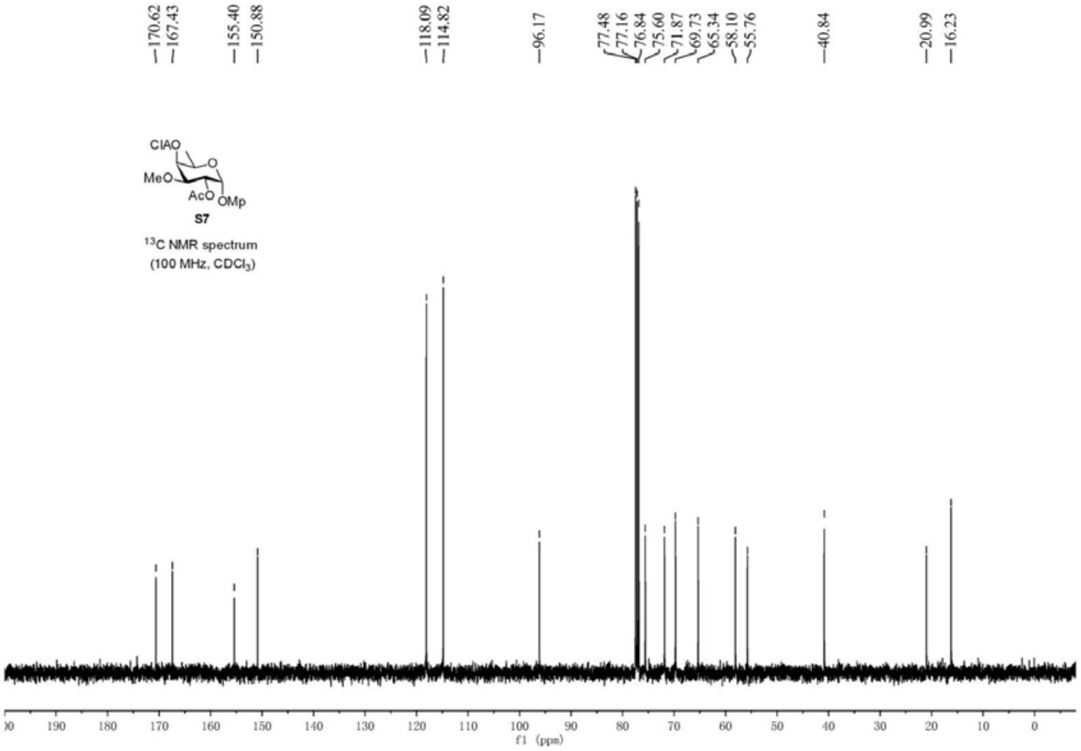

Supplementary Figure 6. ^{1}H and ^{13}C NMR spectra for compound S7.

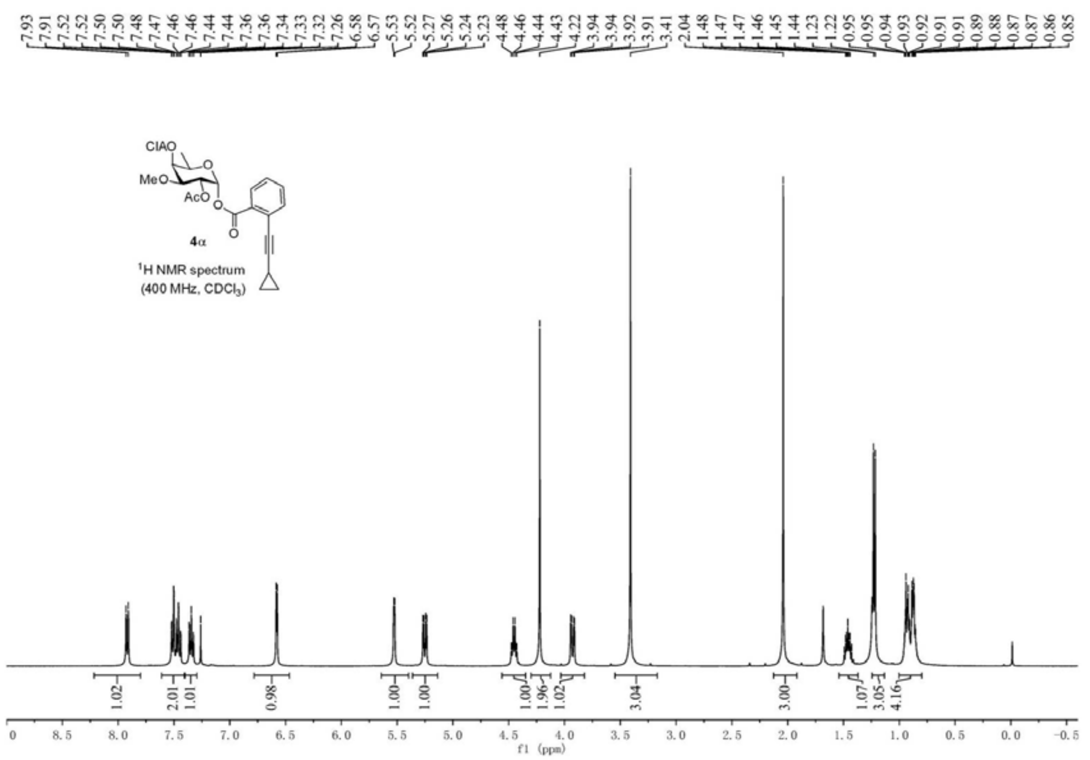

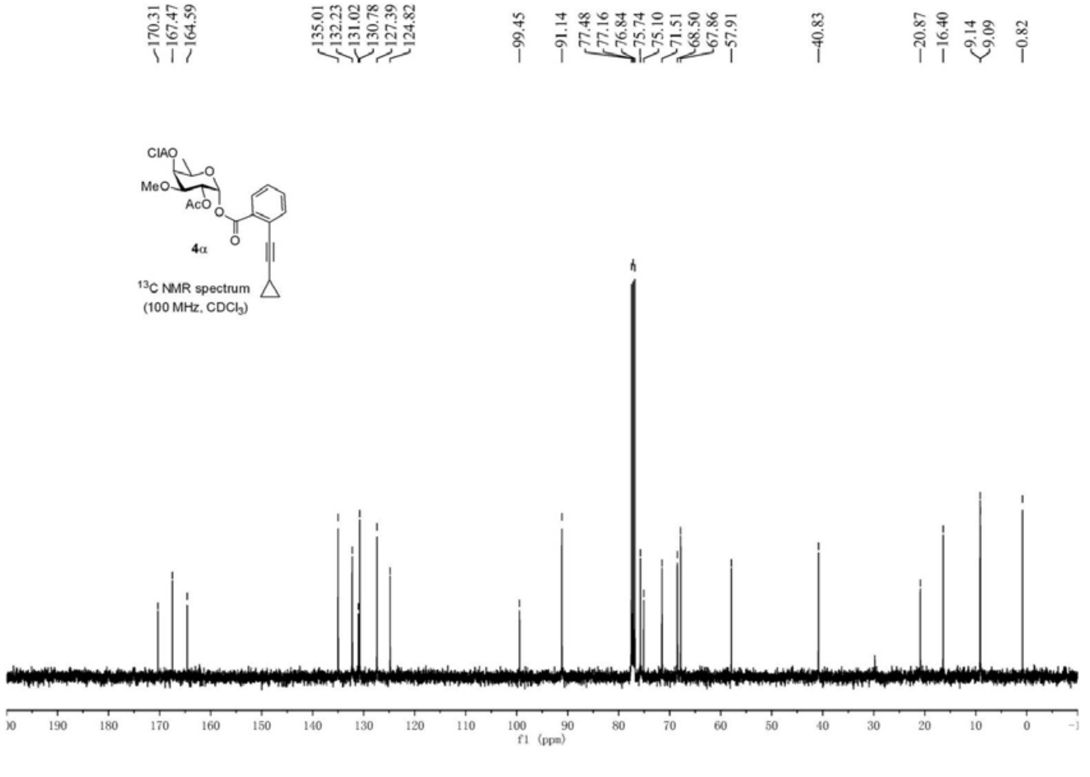

Supplementary Figure 7. ^{1}H and ^{13}C NMR spectra for compound 4α.

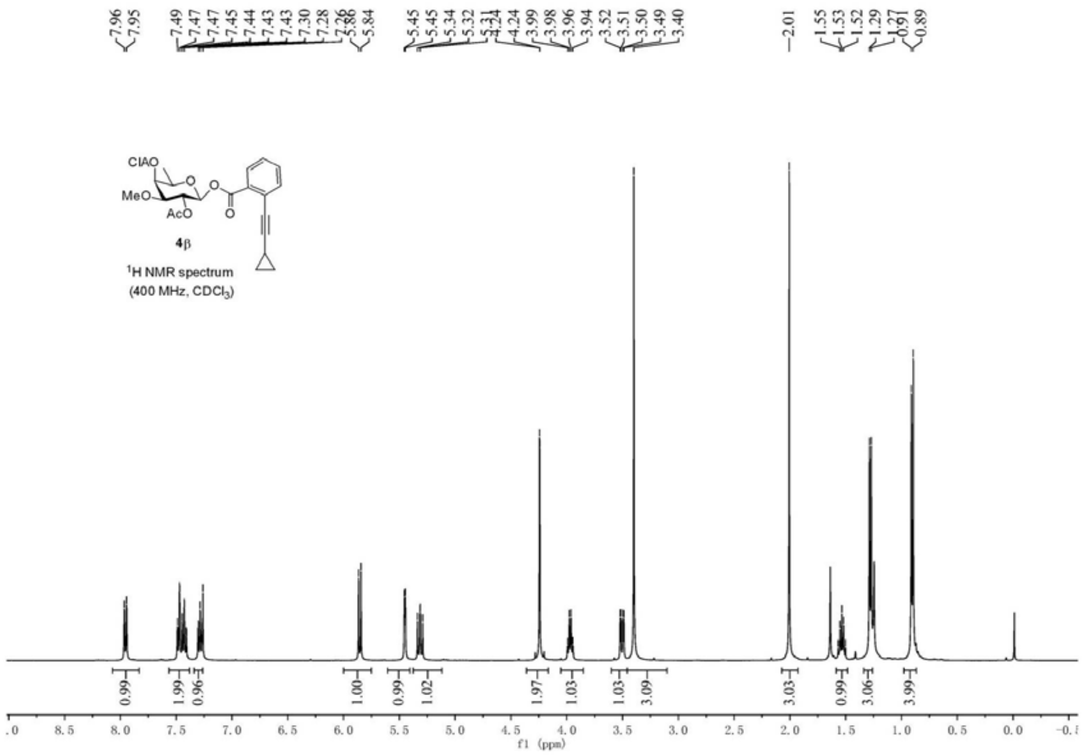

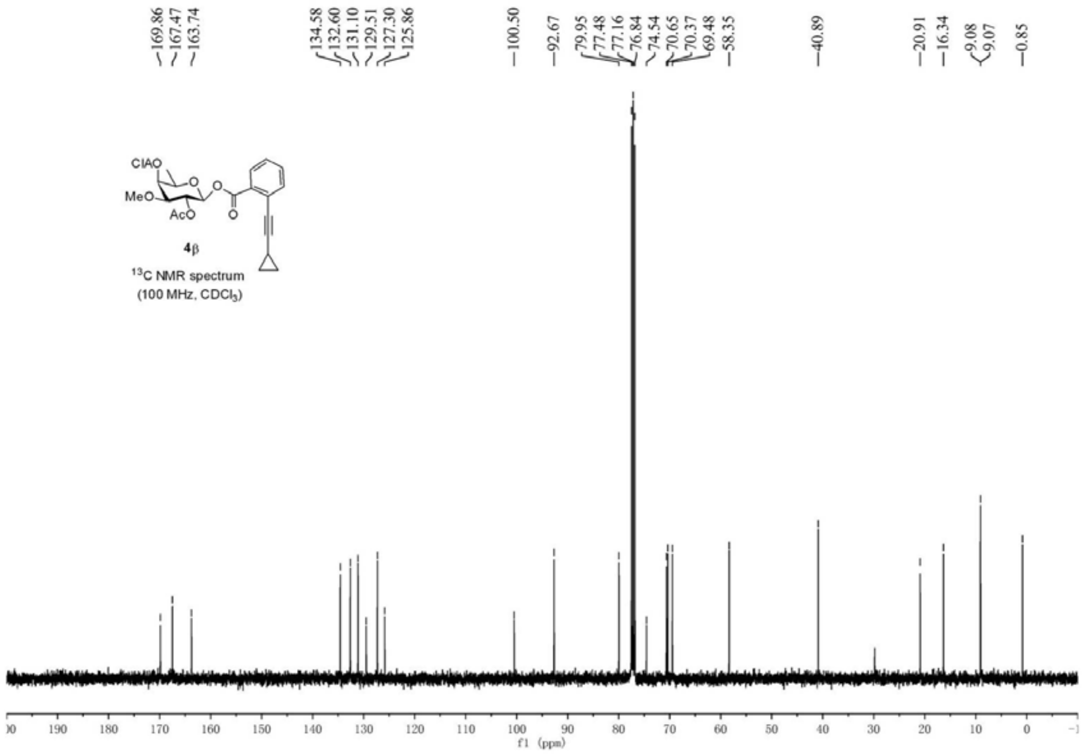

Supplementary Figure 8. ^{1}H and ^{13}C NMR spectra for compound 4β.

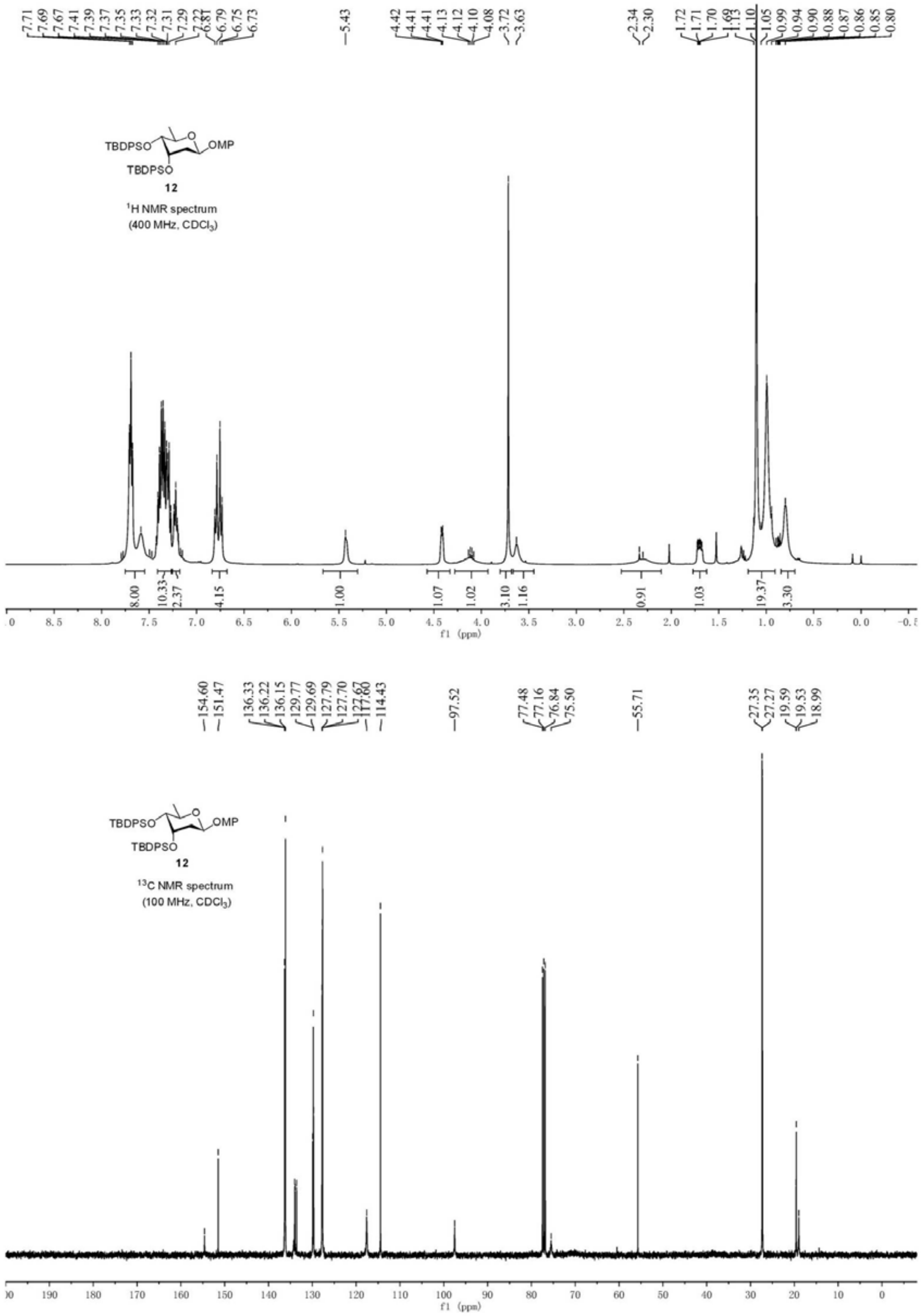

Supplementary Figure 9. ^{1}H and ^{13}C NMR spectra for compound 12.

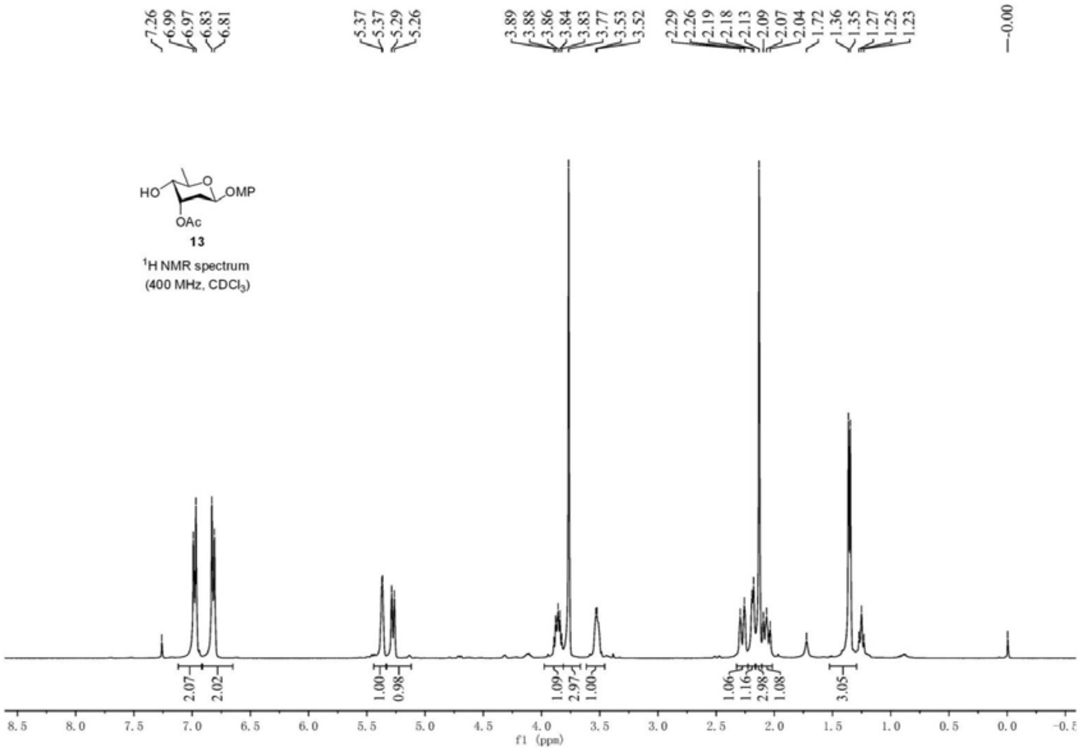

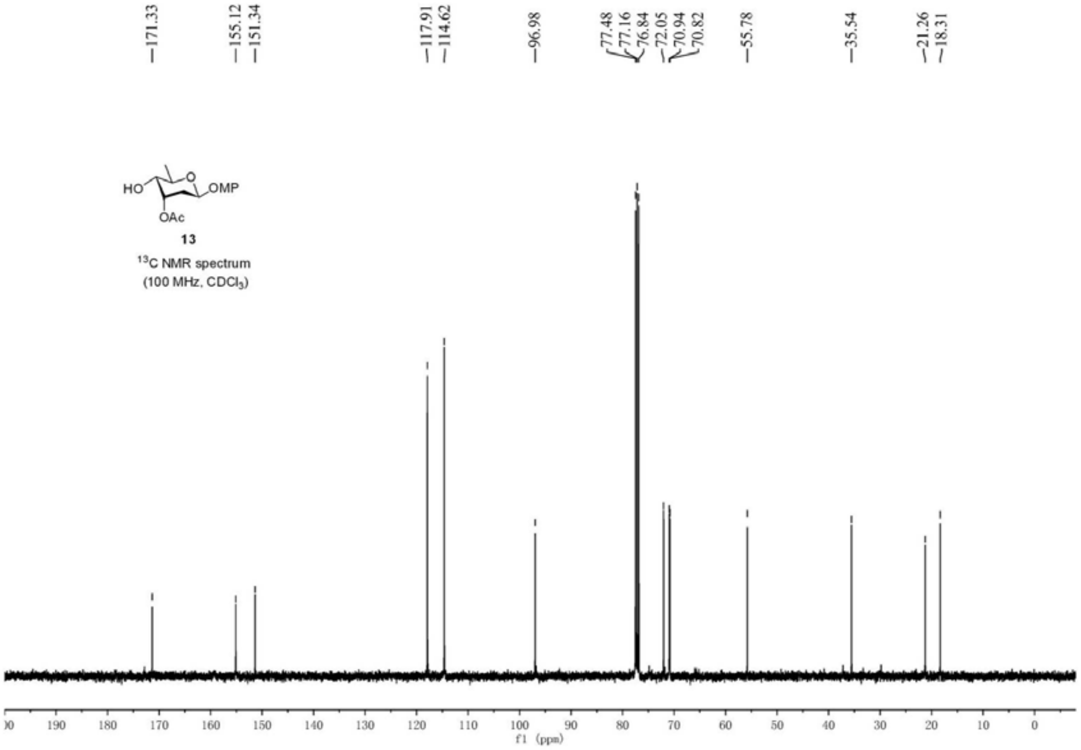

Supplementary Figure 10. ^{1}H and ^{13}C NMR spectra for compound 13.

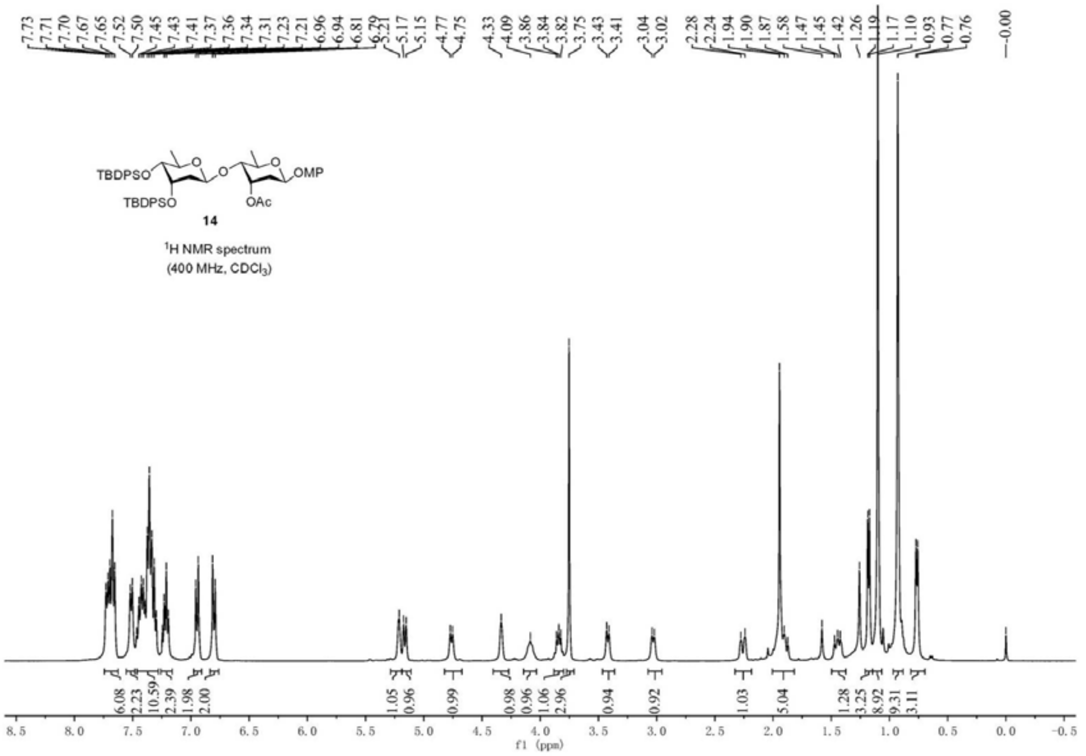

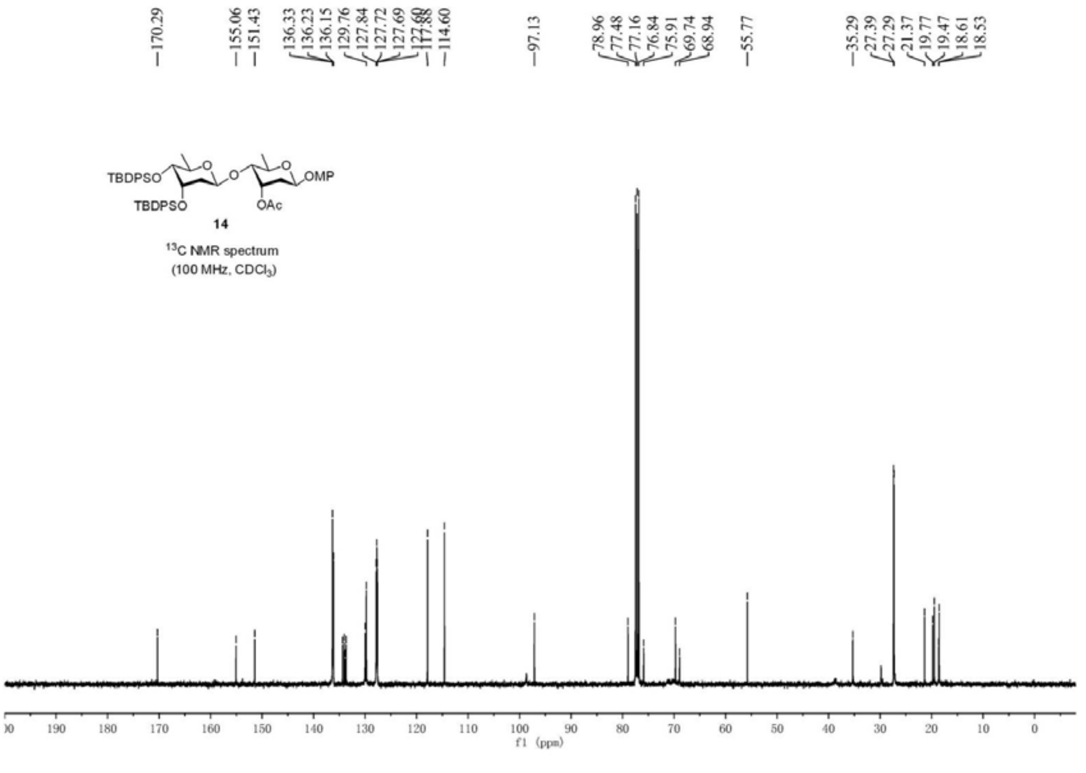

Supplementary Figure 11. ^{1}H and ^{13}C NMR spectra for compound 14.

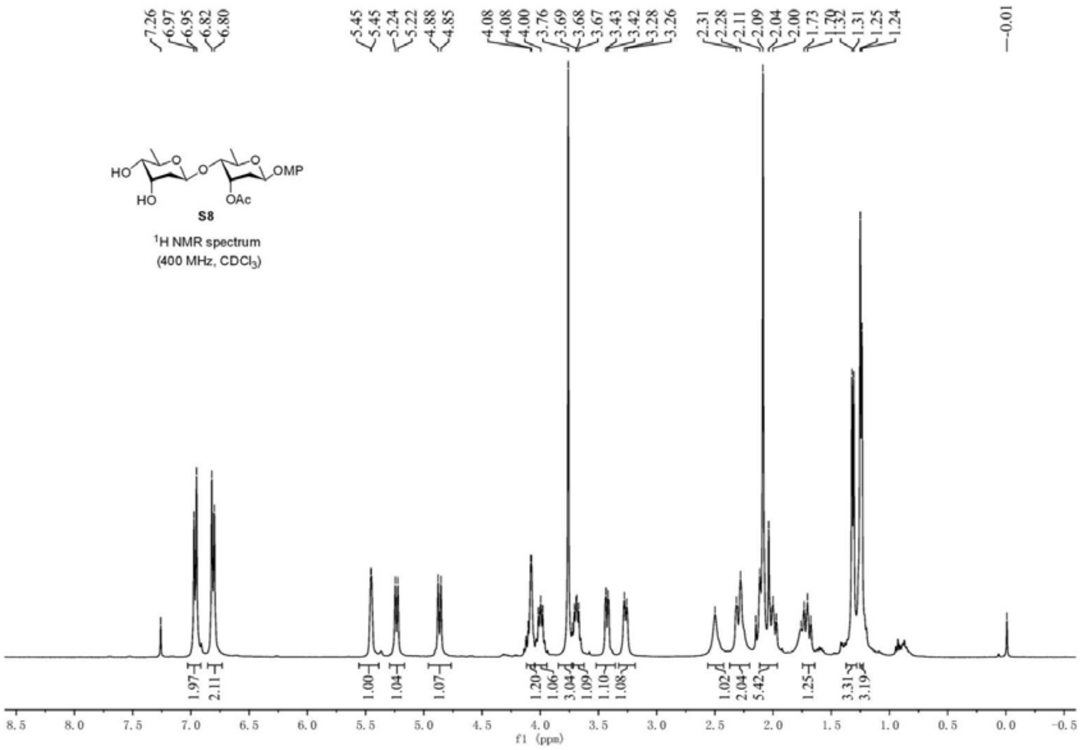

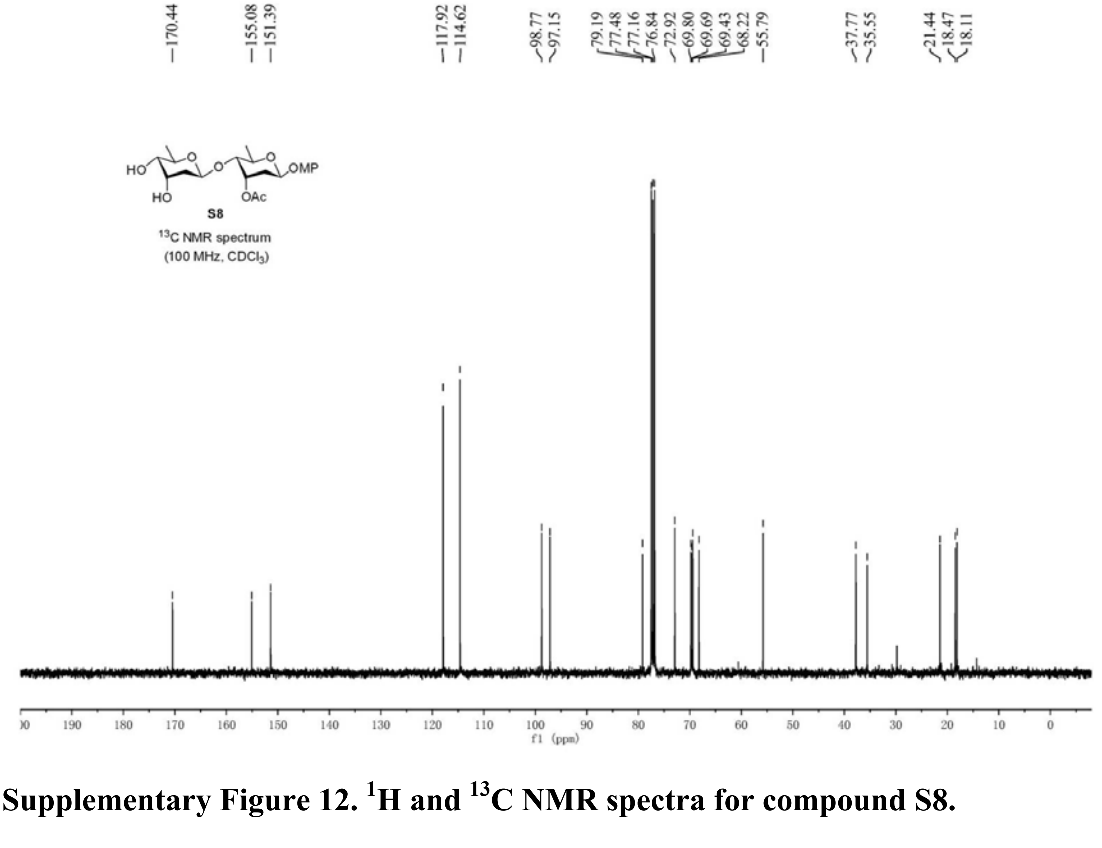

Supplementary Figure 12. ^{1}H and ^{13}C NMR spectra for compound S8.

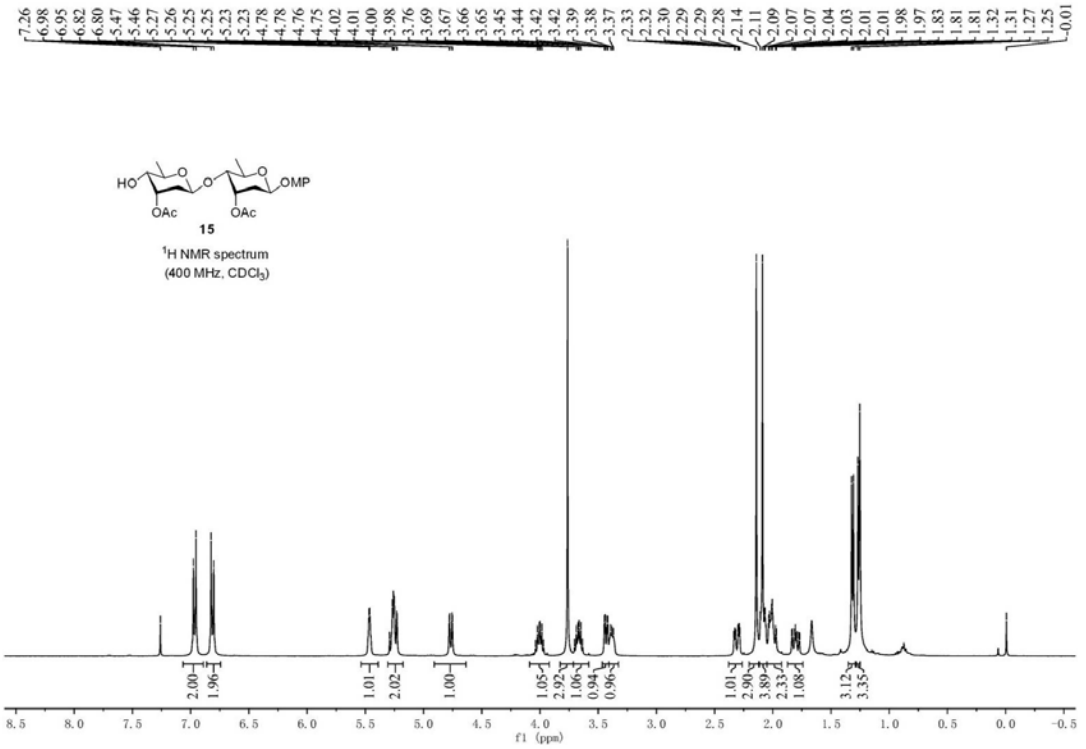

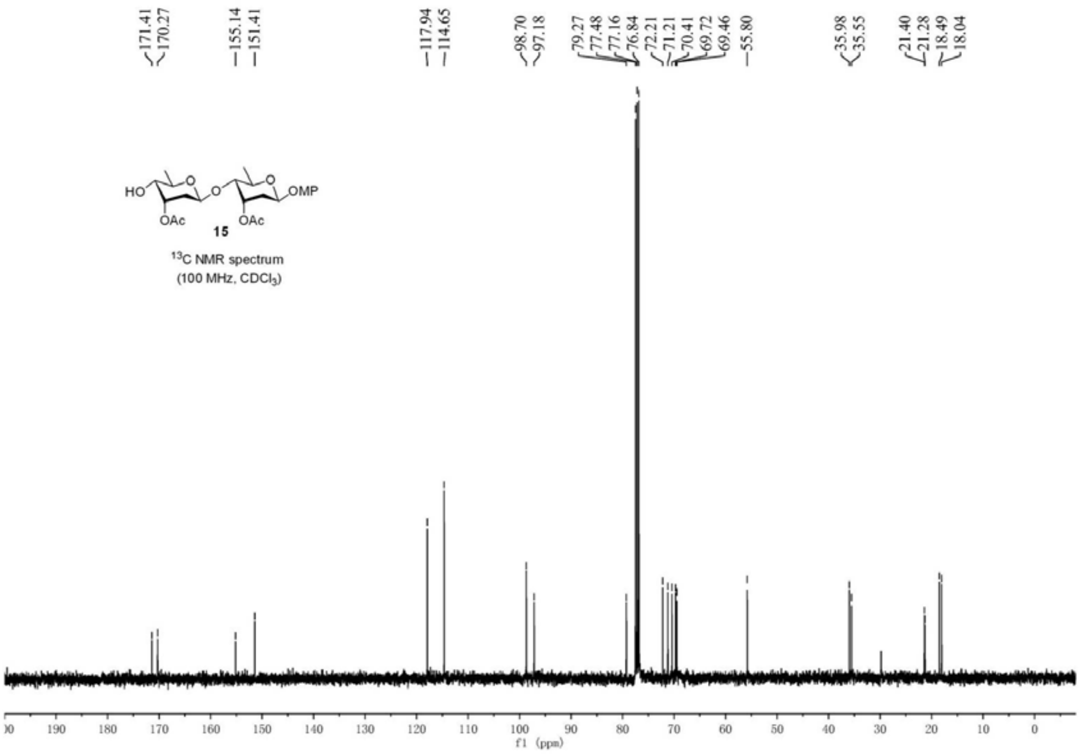

Supplementary Figure 13. ^{1}H and ^{13}C NMR spectra for compound 15.

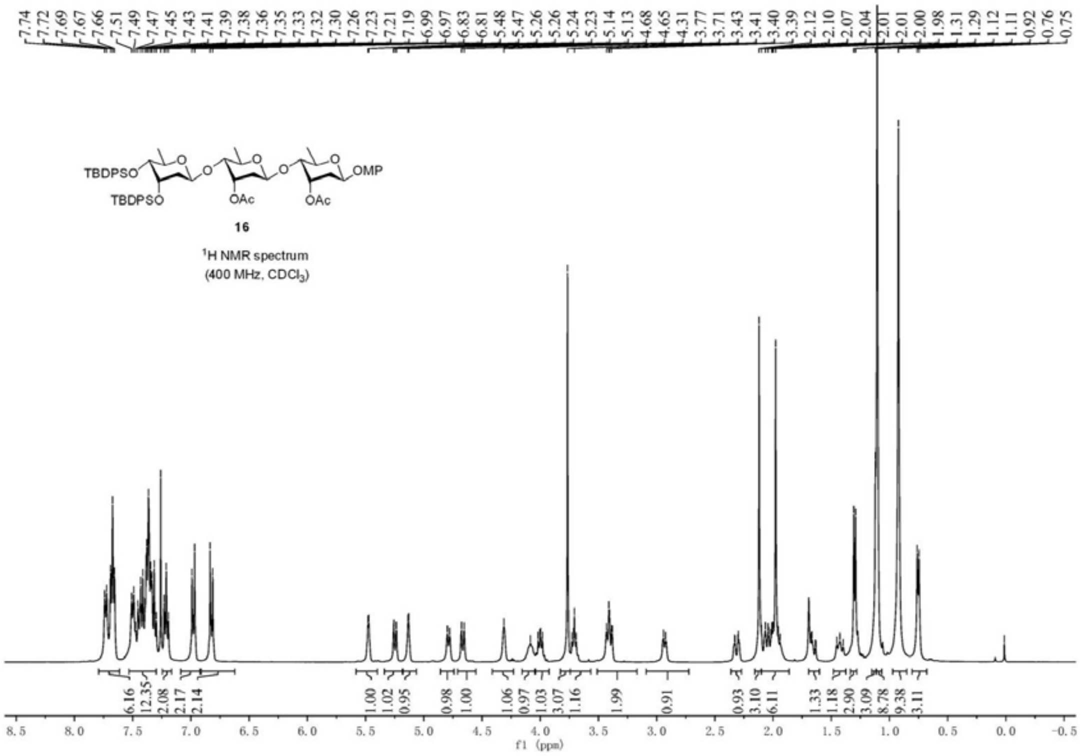

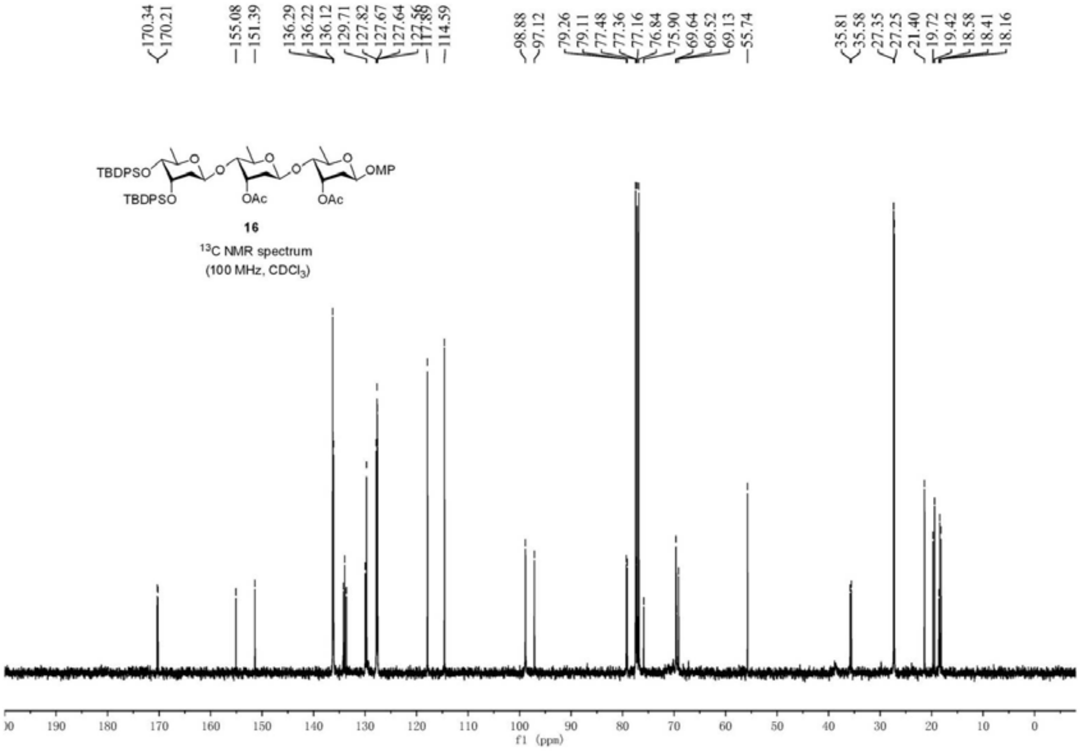

Supplementary Figure 14. ^{1}H and ^{13}C NMR spectra for compound 16.

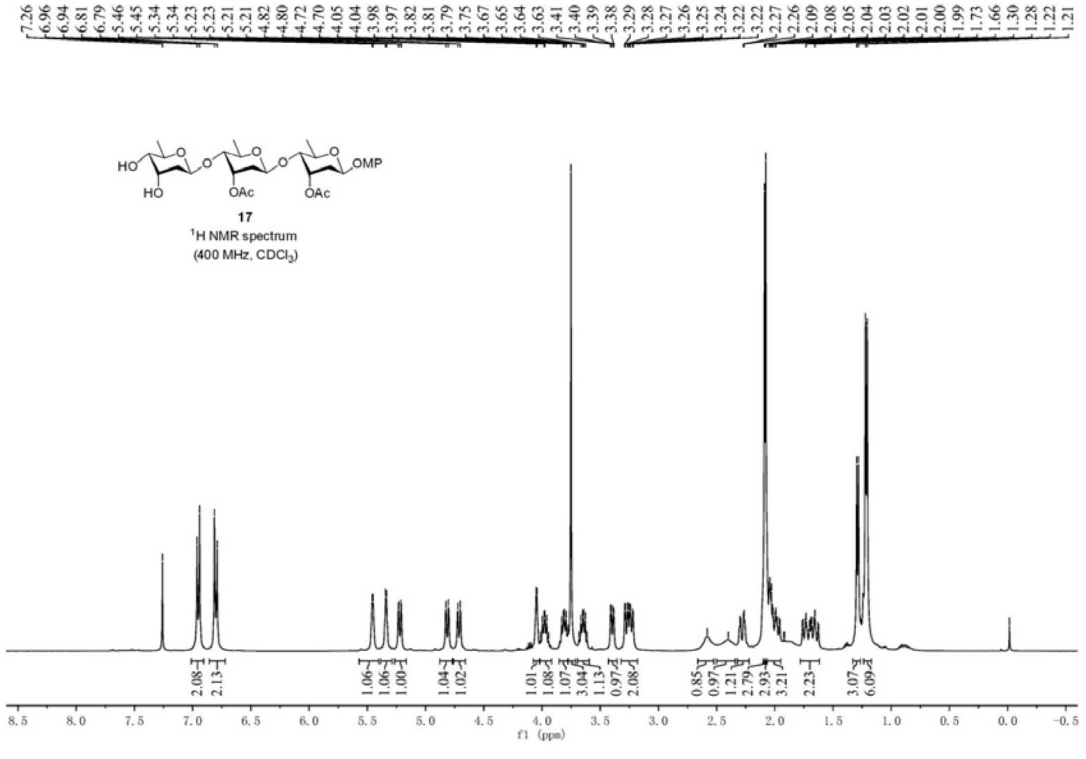

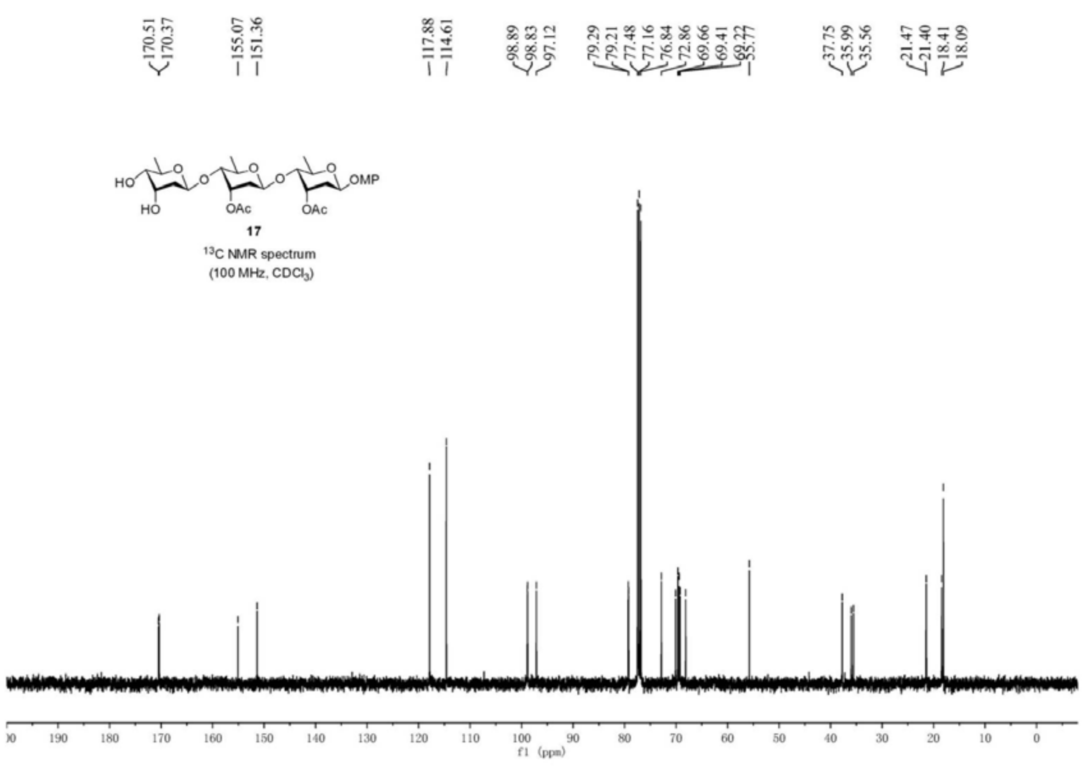

Supplementary Figure 15. ^{1}H and ^{13}C NMR spectra for compound 17.

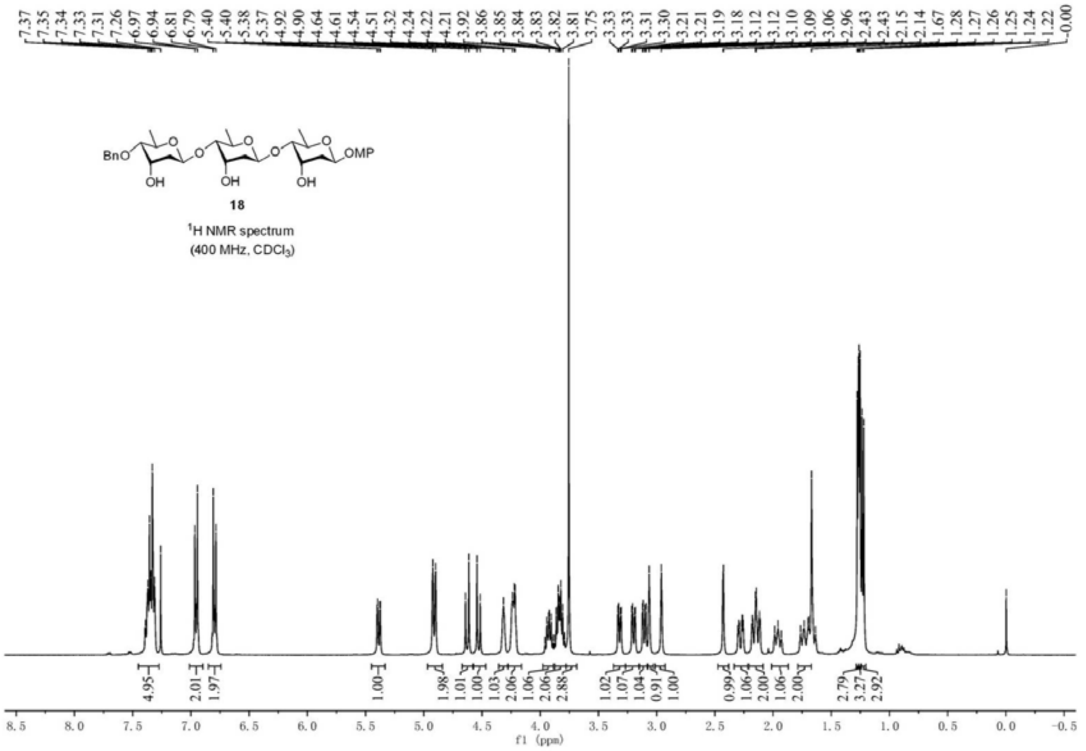

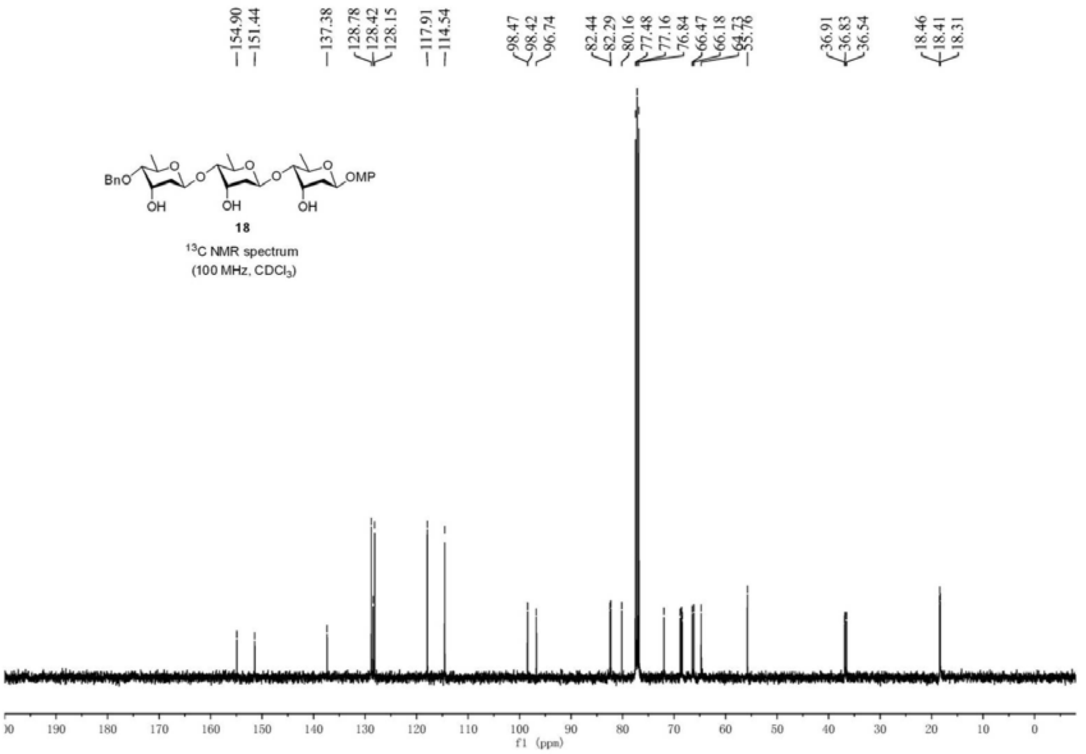

Supplementary Figure 16. ^{1}H and ^{13}C NMR spectra for compound 18.

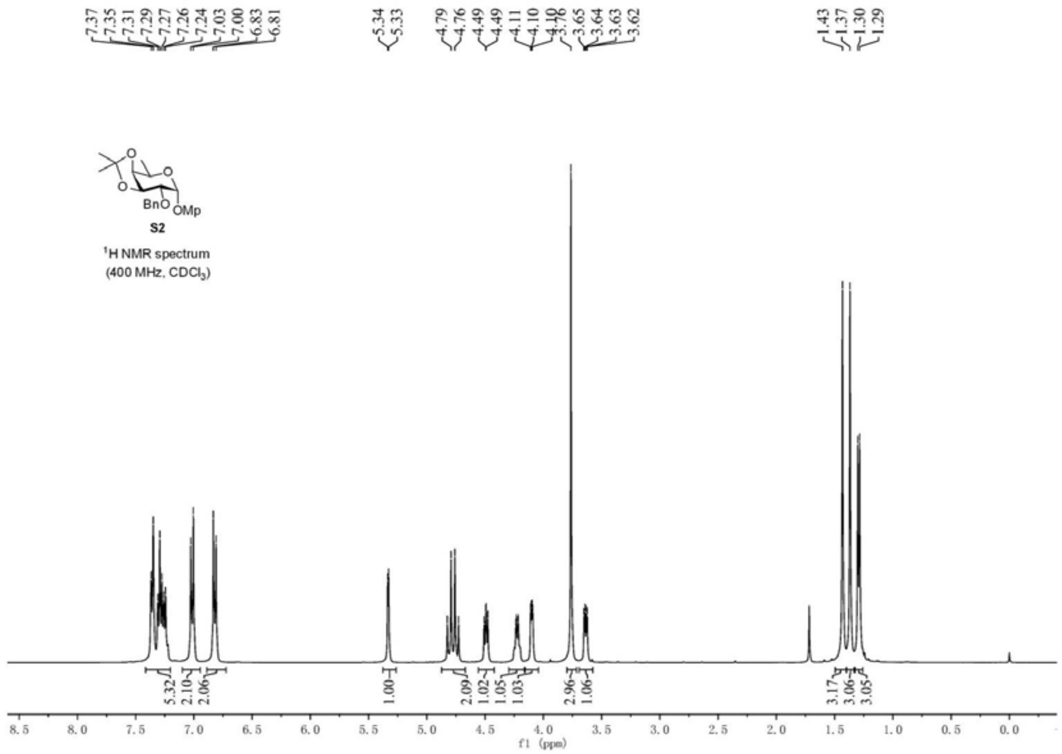

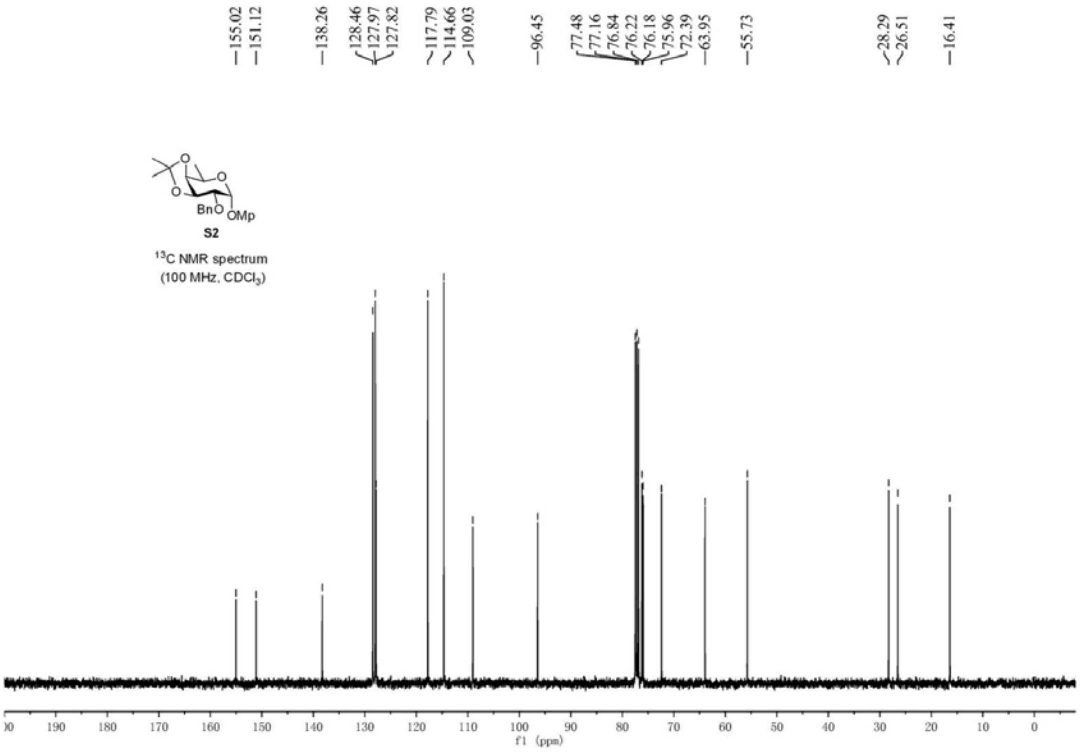

Supplementary Figure 17. ^{1}H and ^{13}C NMR spectra for compound S2.

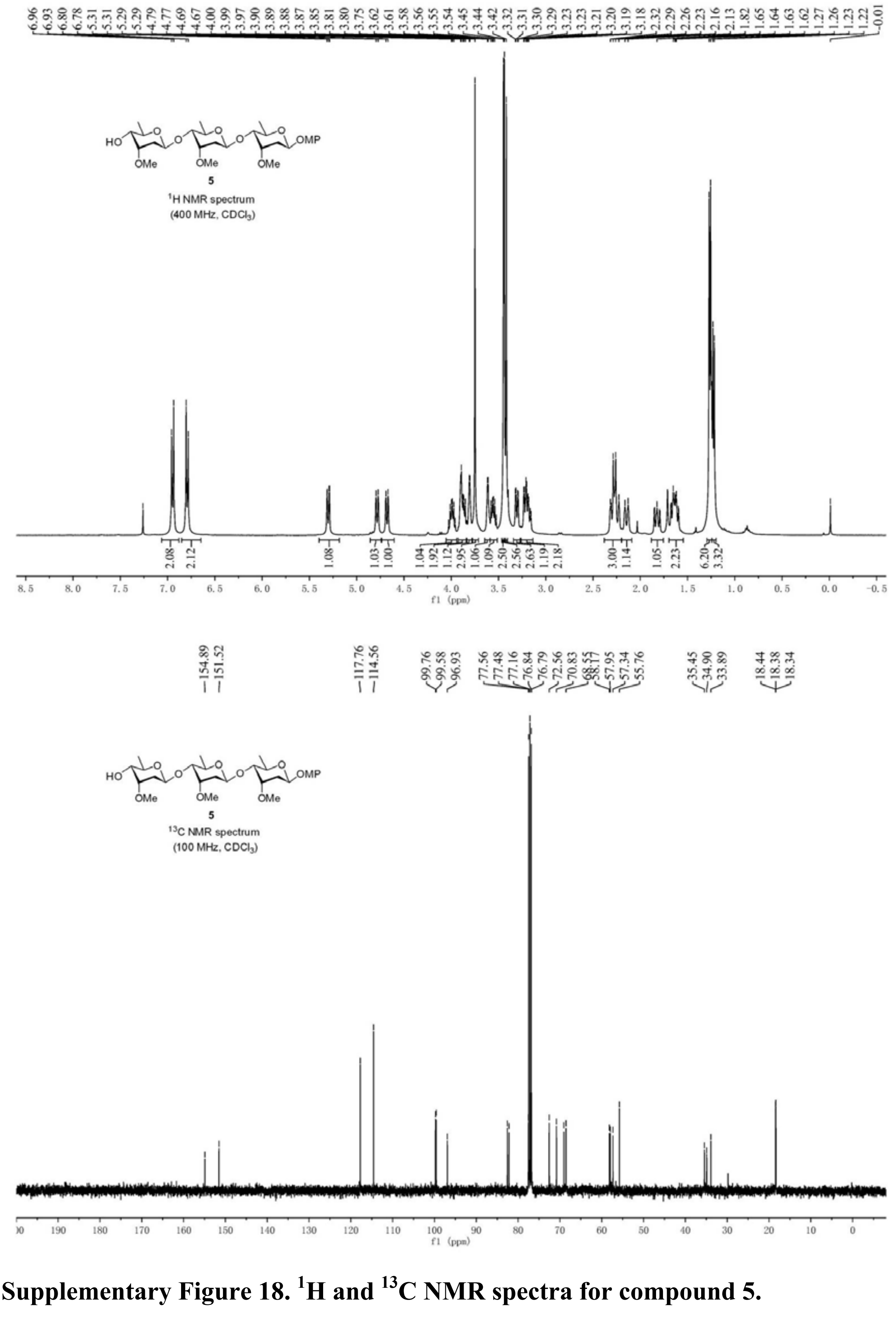

Supplementary Figure 18. ^{1}H and ^{13}C NMR spectra for compound 5.

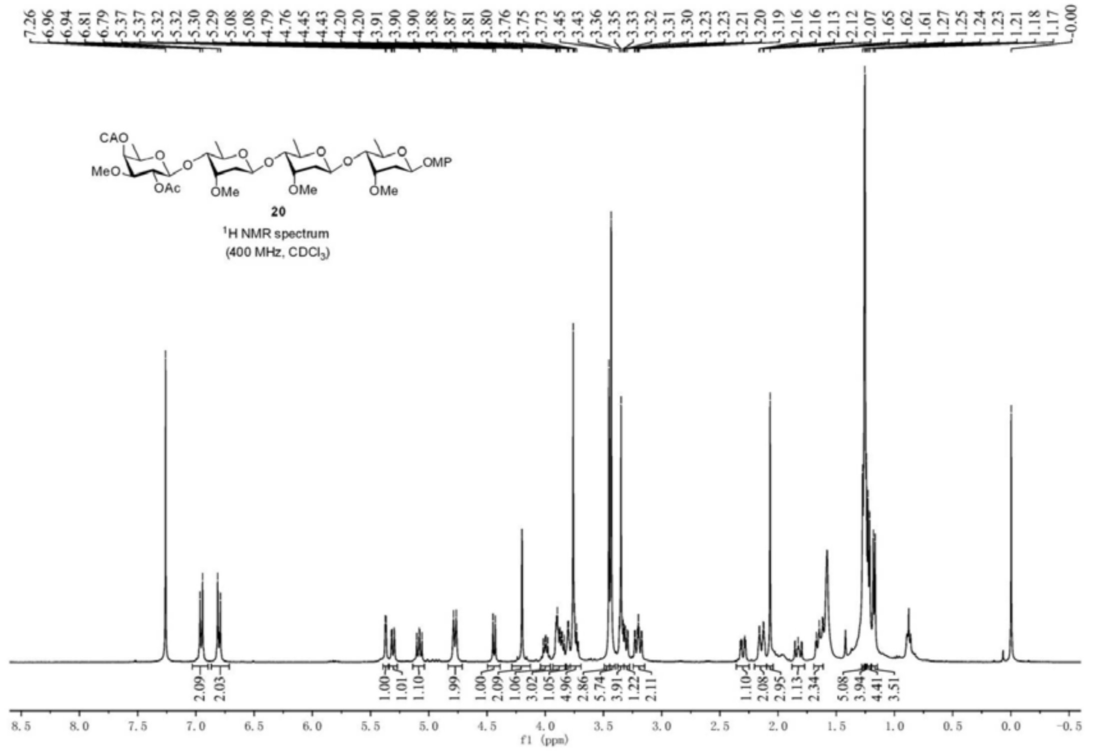

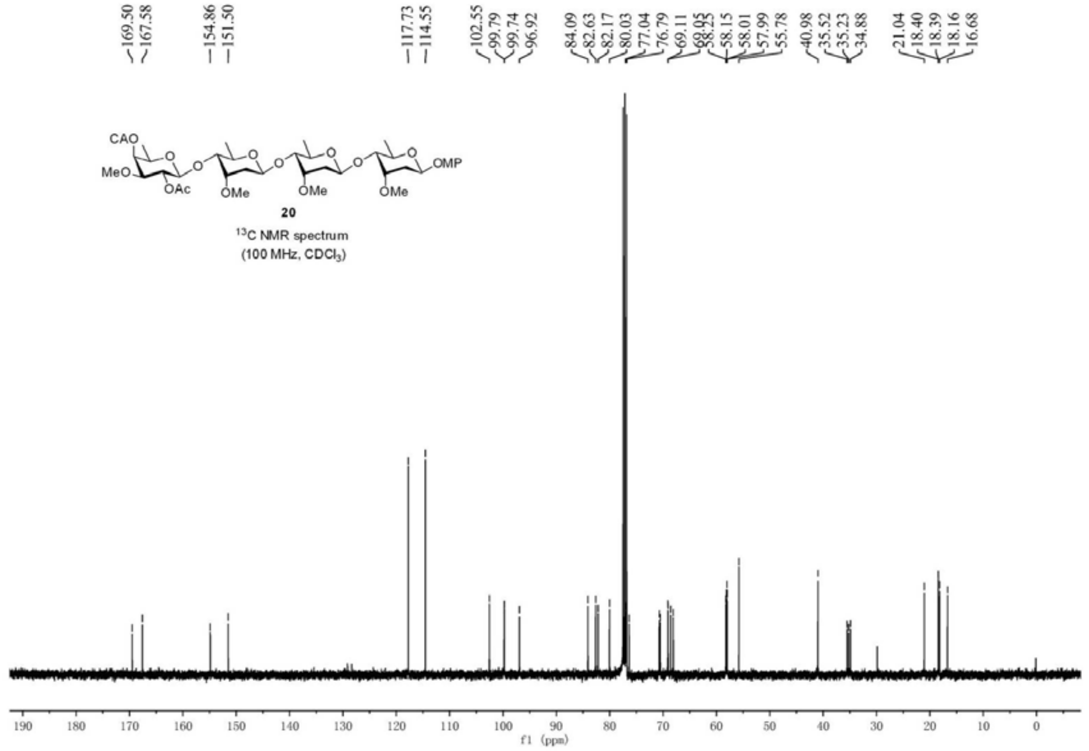

Supplementary Figure 19. ^{1}H and ^{13}C NMR spectra for compound 20.

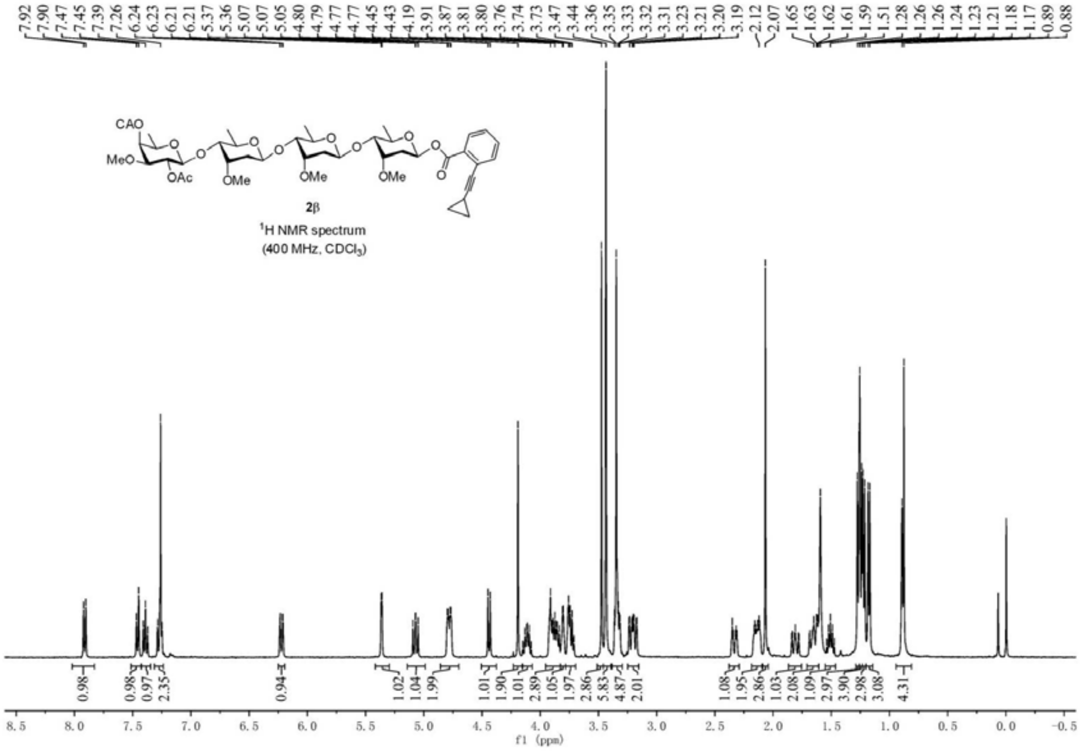

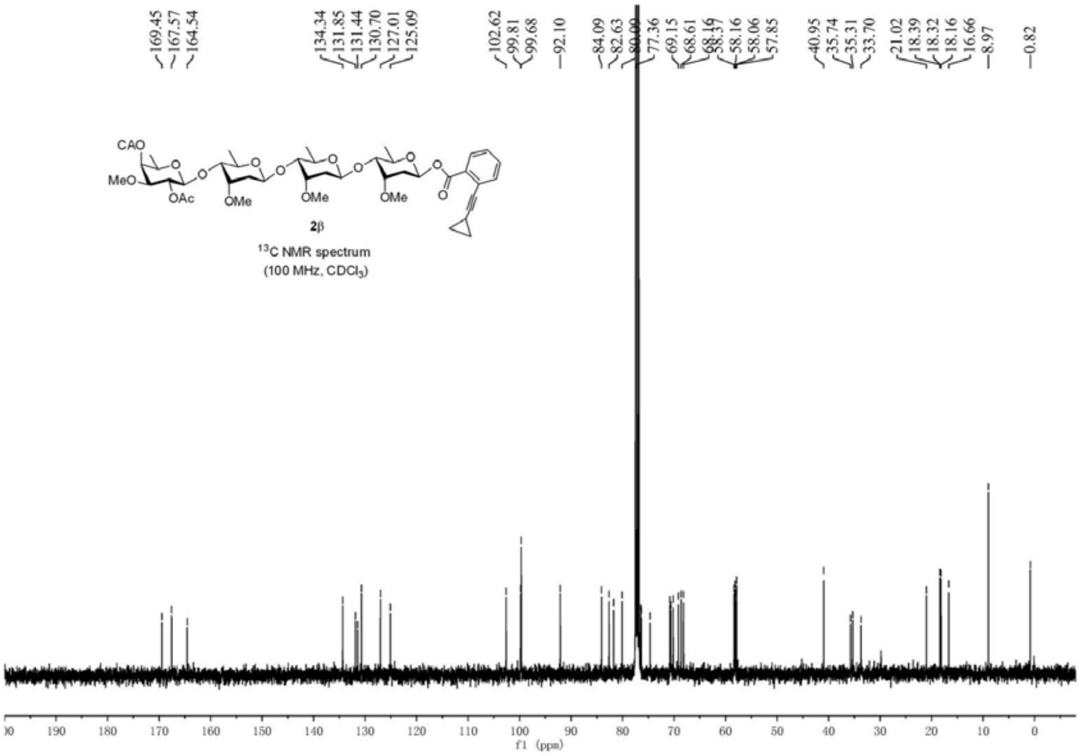

Supplementary Figure 20. ^{1}H and ^{13}C NMR spectra for compound 2β.

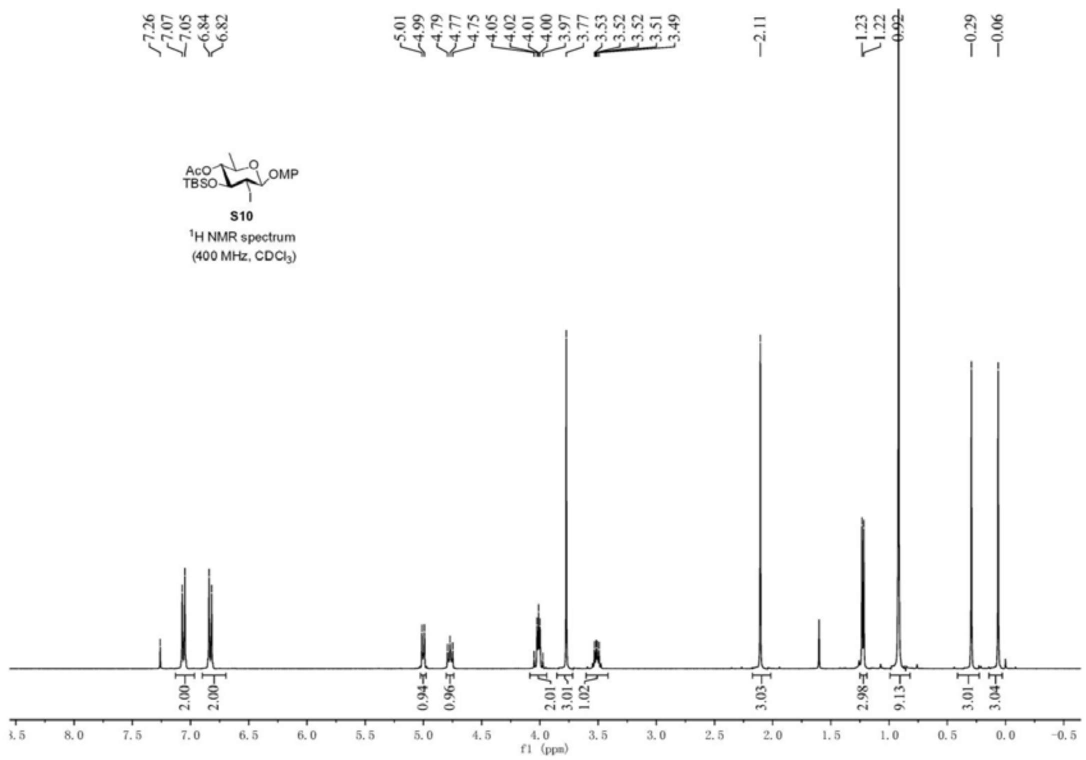

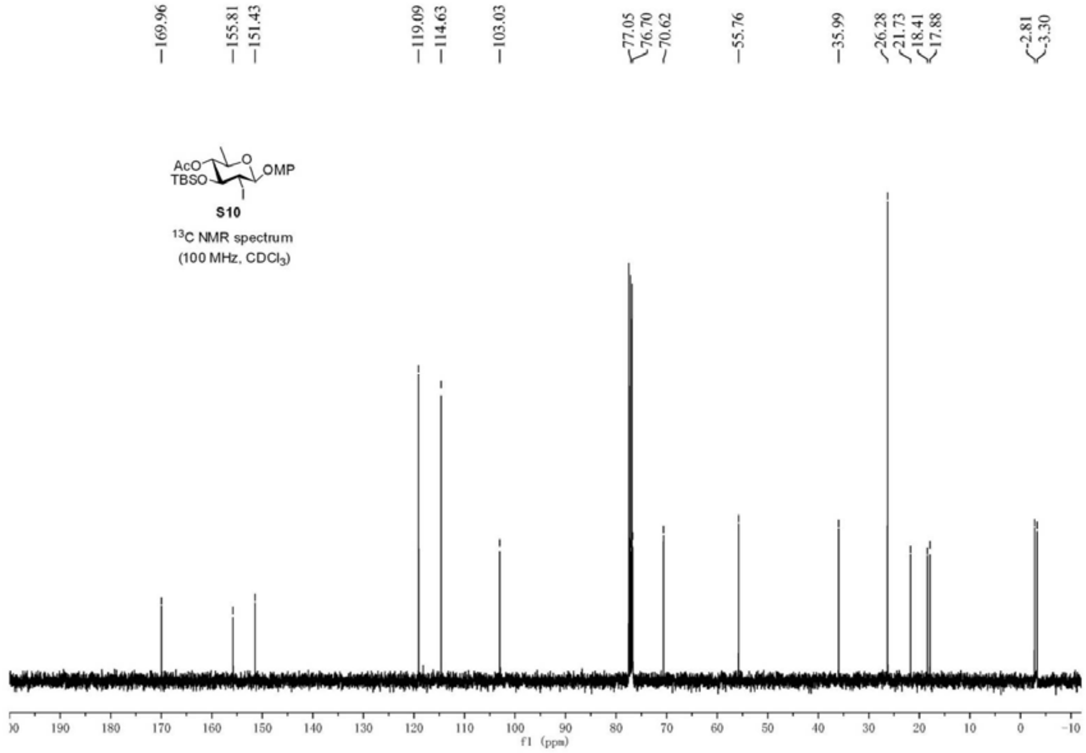

Supplementary Figure 21. ^{1}H and ^{13}C NMR spectra for compound S10.

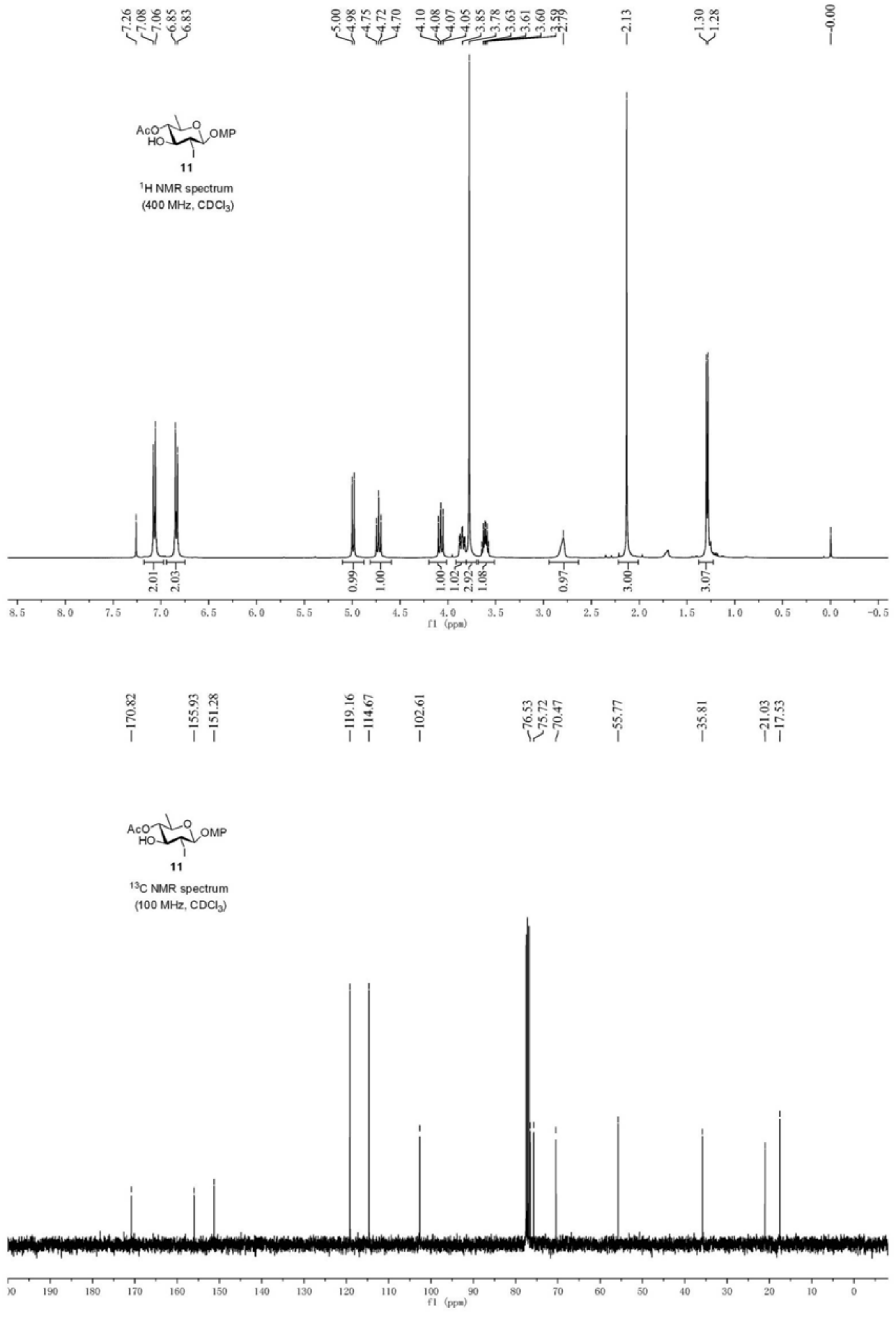

Supplementary Figure 22. ^{1}H and ^{13}C NMR spectra for compound 11.

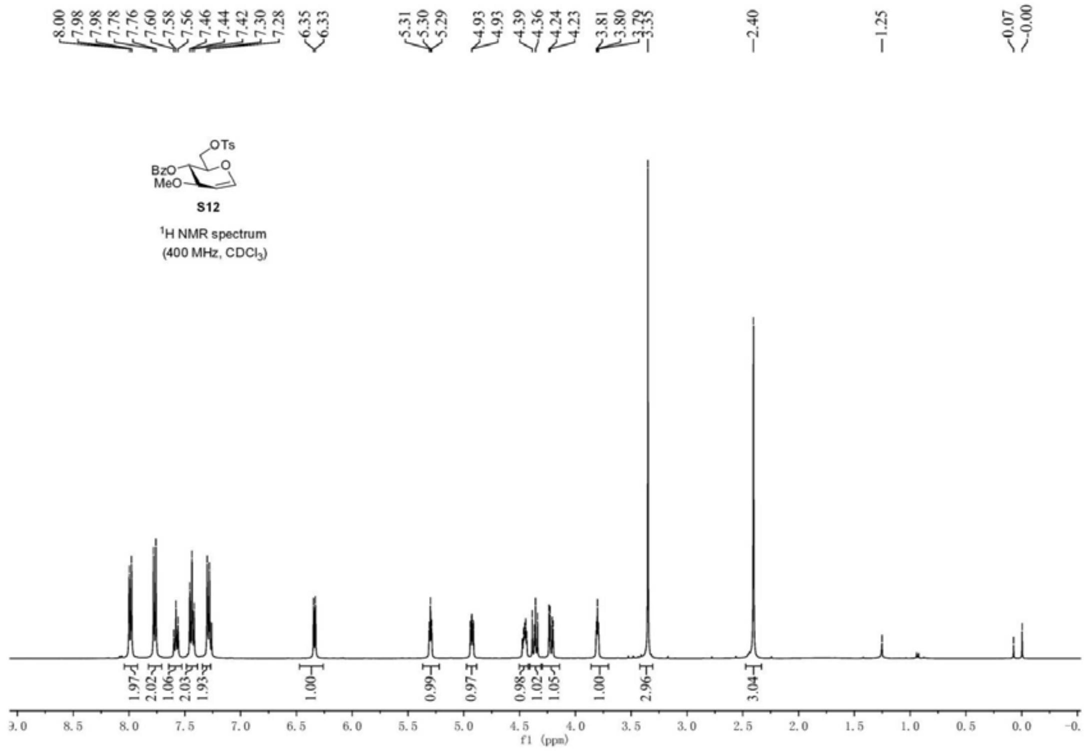

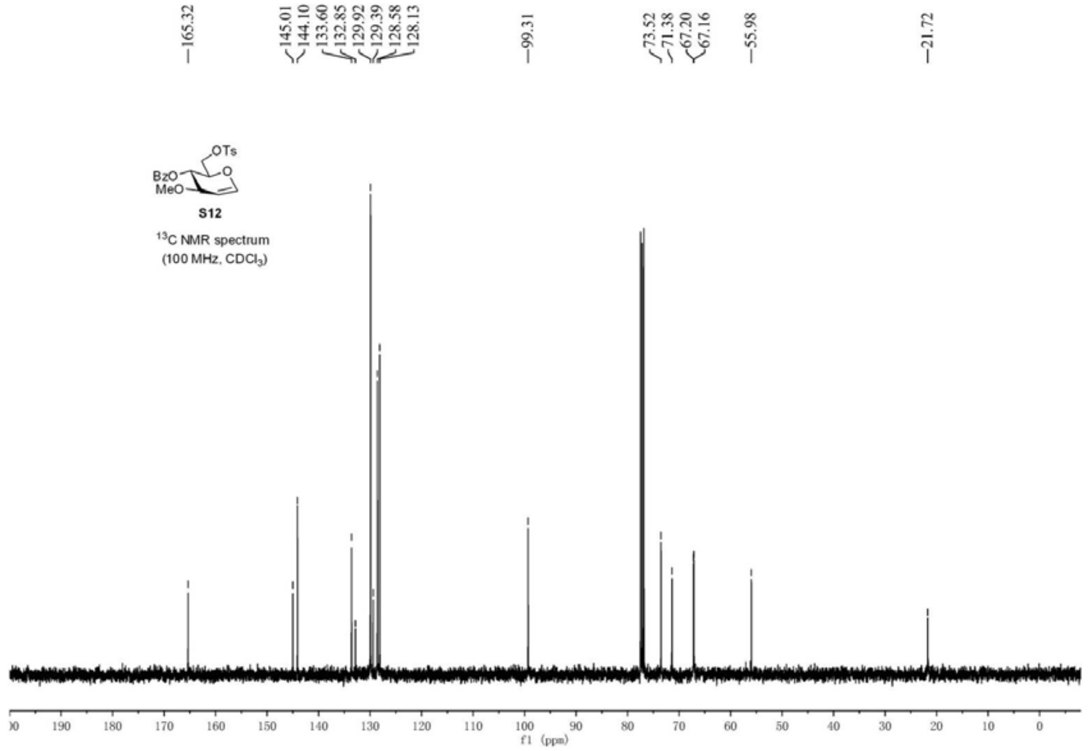

Supplementary Figure 23. ^{1}H and ^{13}C NMR spectra for compound S12.

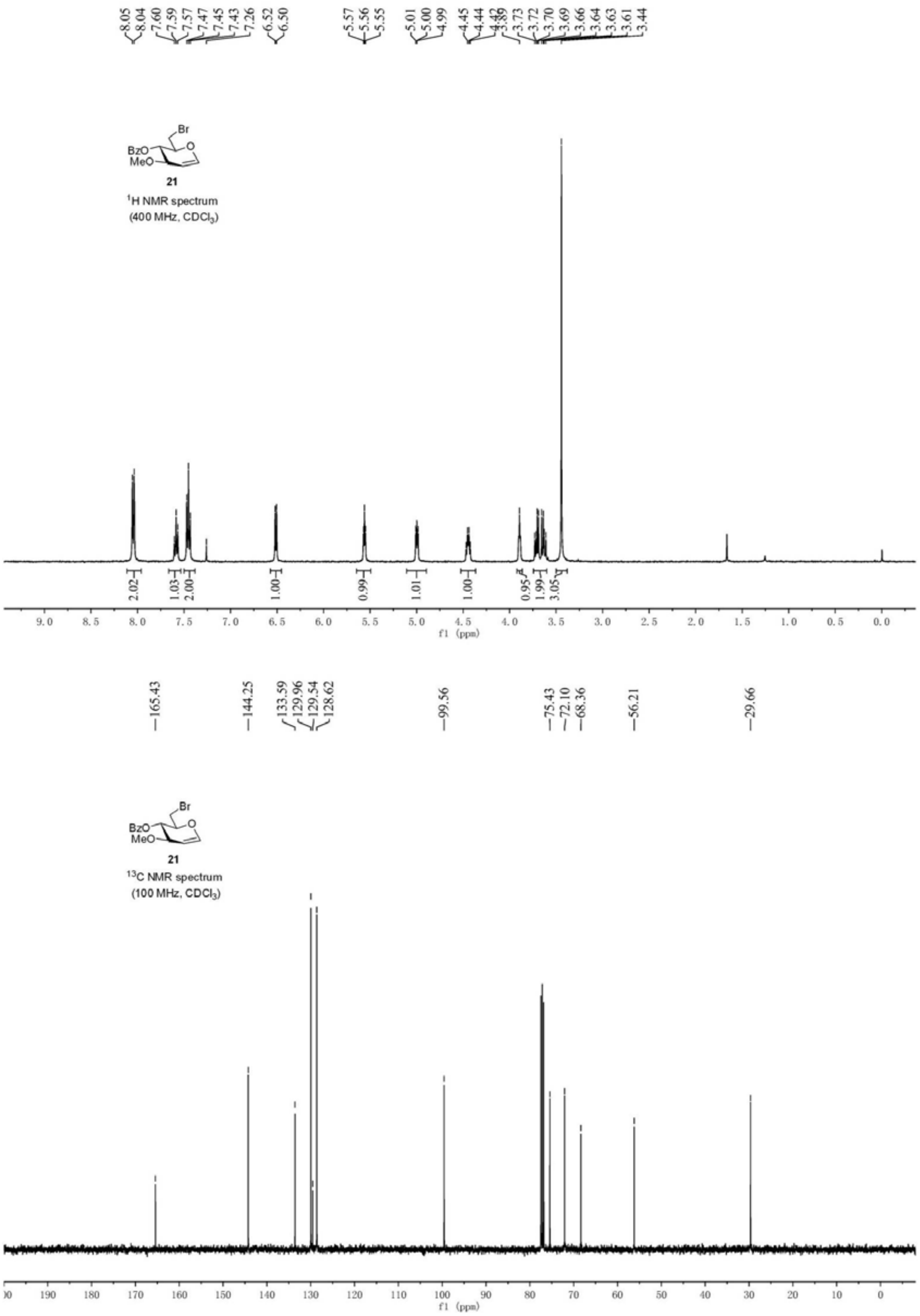

Supplementary Figure 24. ^{1}H and ^{13}C NMR spectra for compound 21.

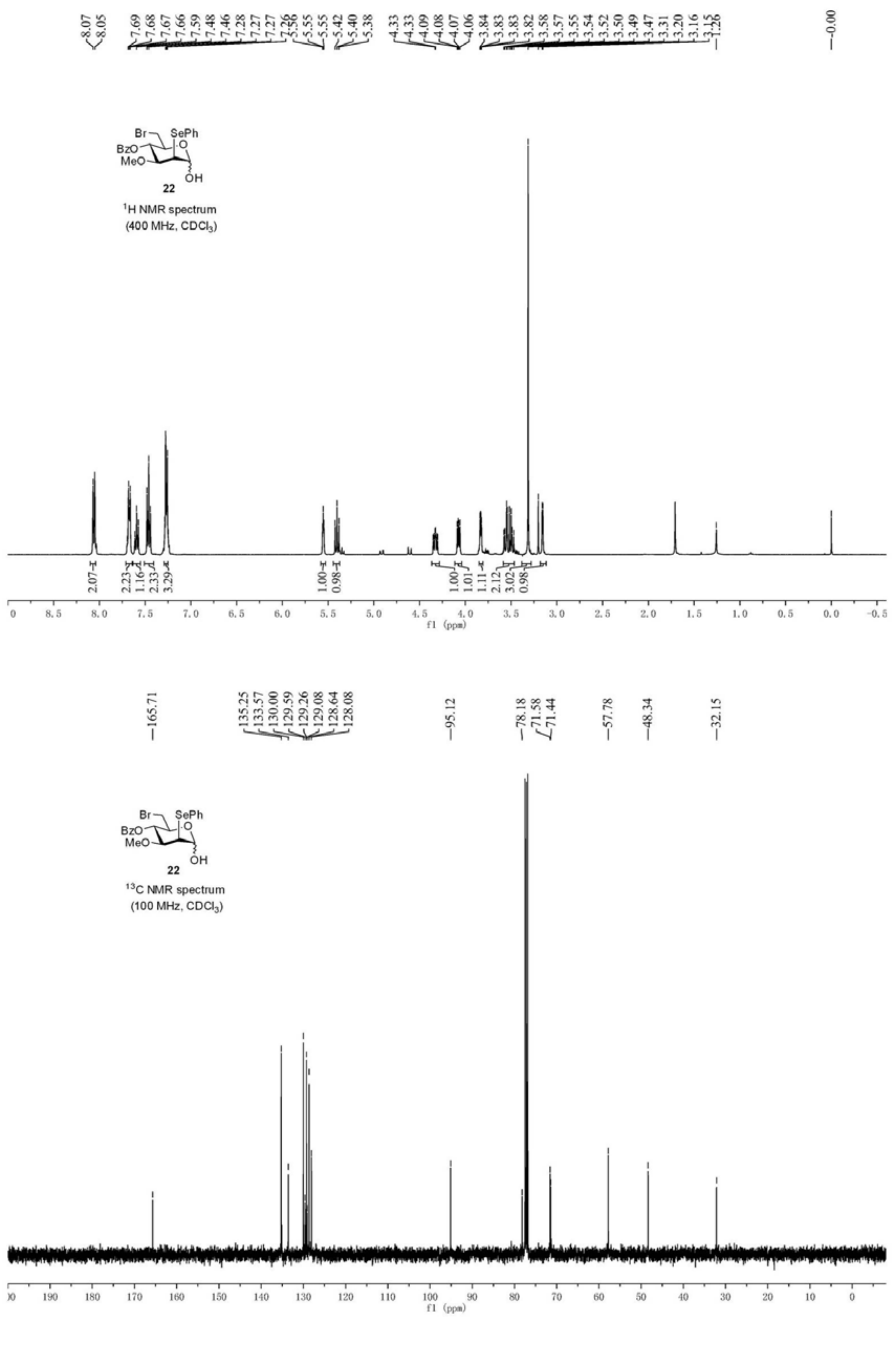

Supplementary Figure 25. ^{1}H and ^{13}C NMR spectra for compound 22.

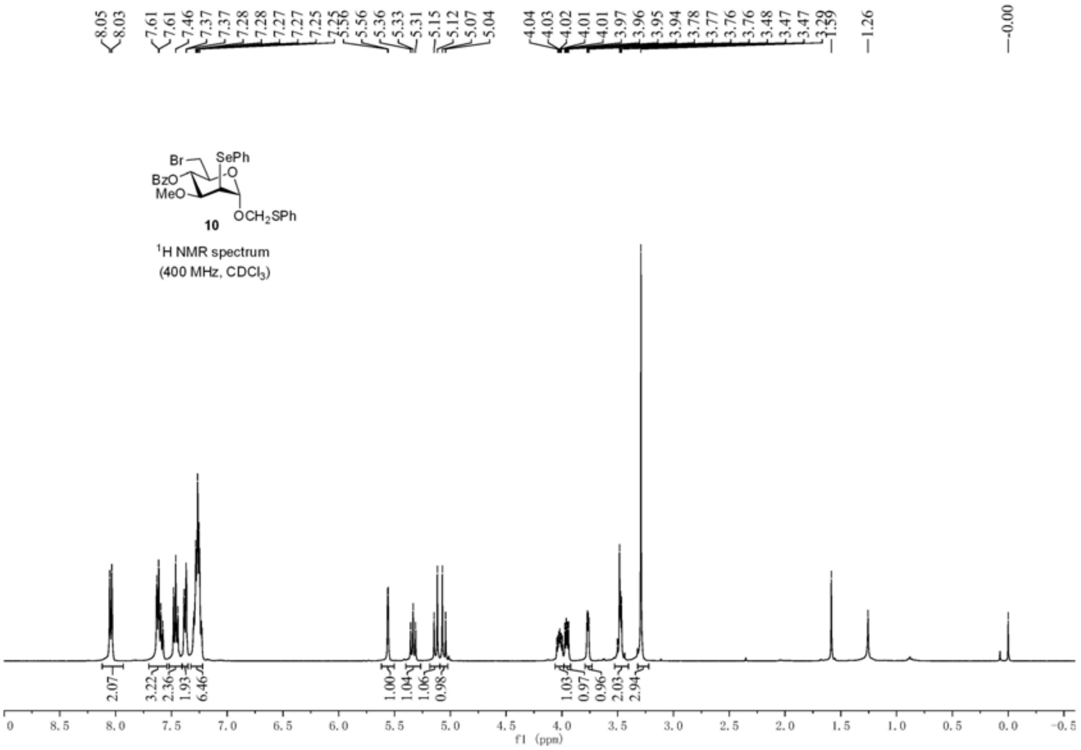

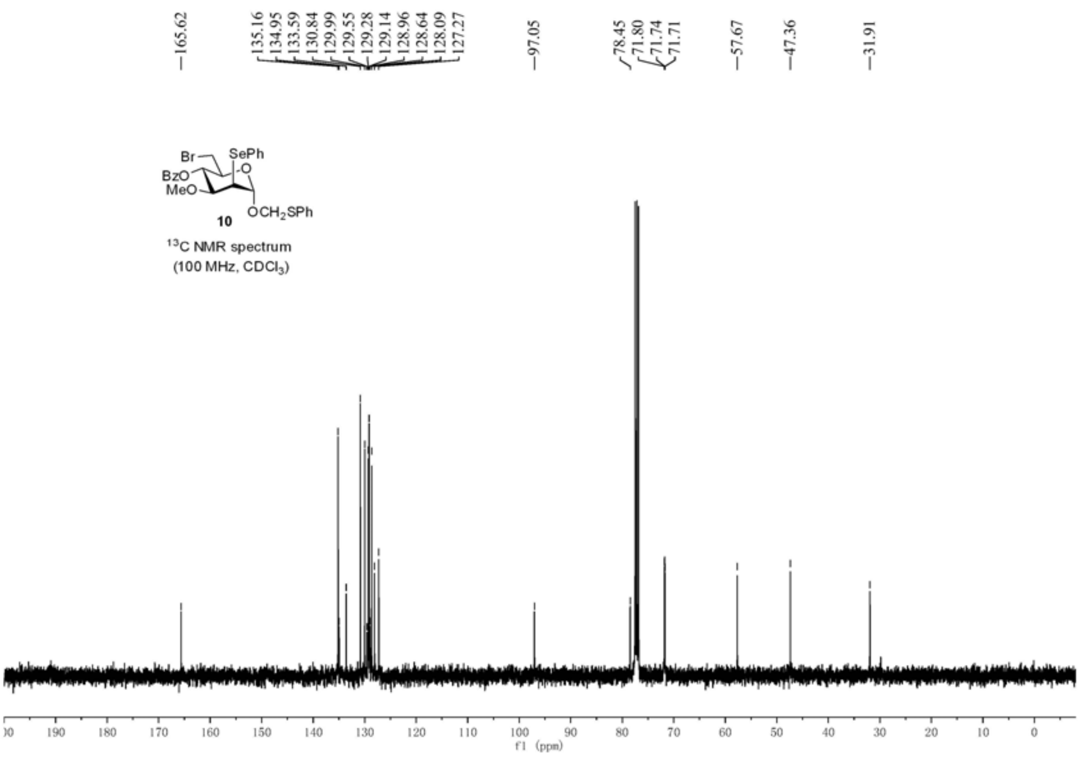

Supplementary Figure 26. ^{1}H and ^{13}C NMR spectra for compound 10.

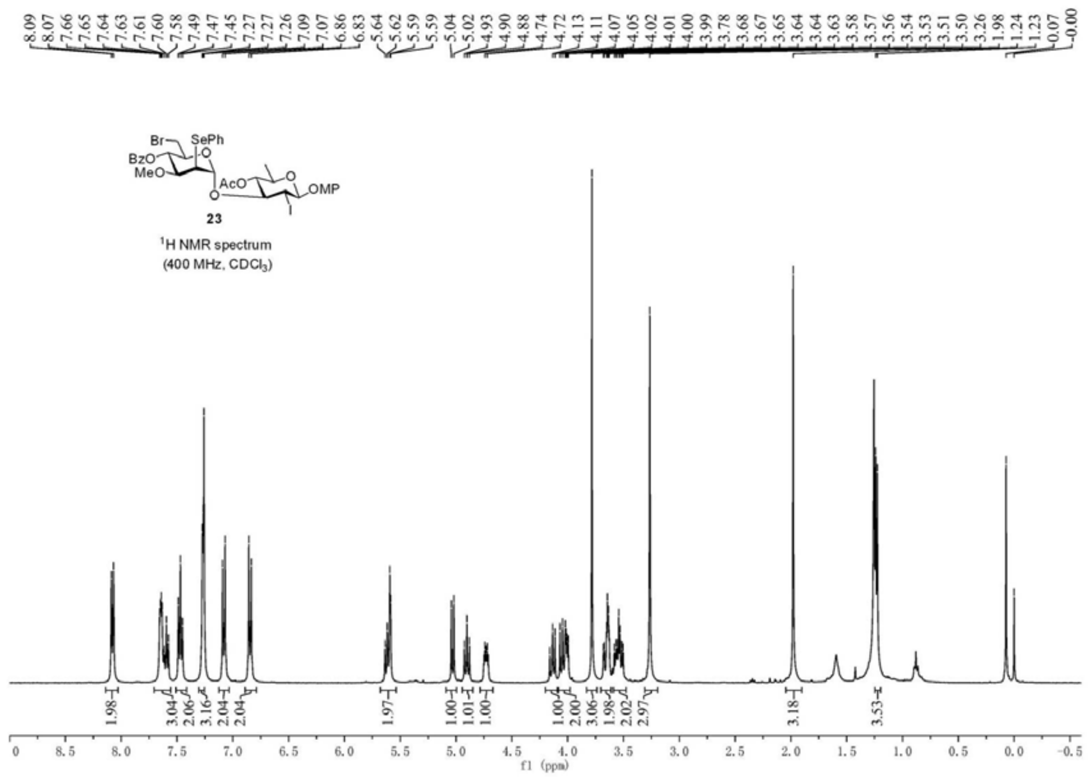

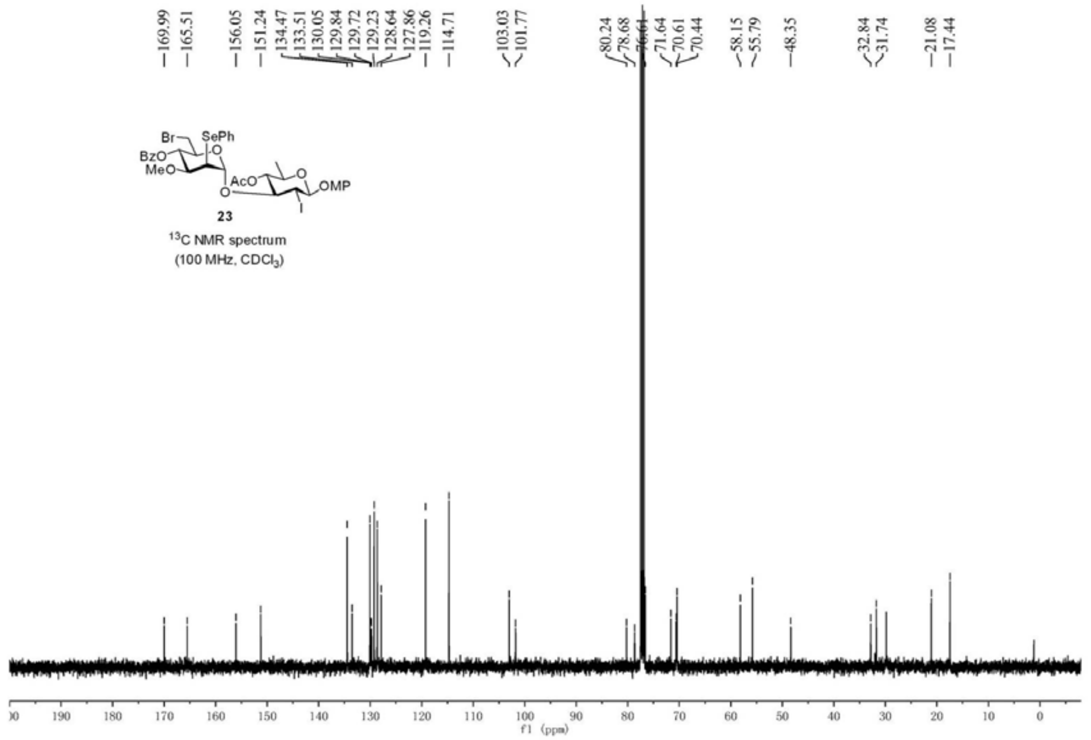

Supplementary Figure 27. ^{1}H and ^{13}C NMR spectra for compound 23.

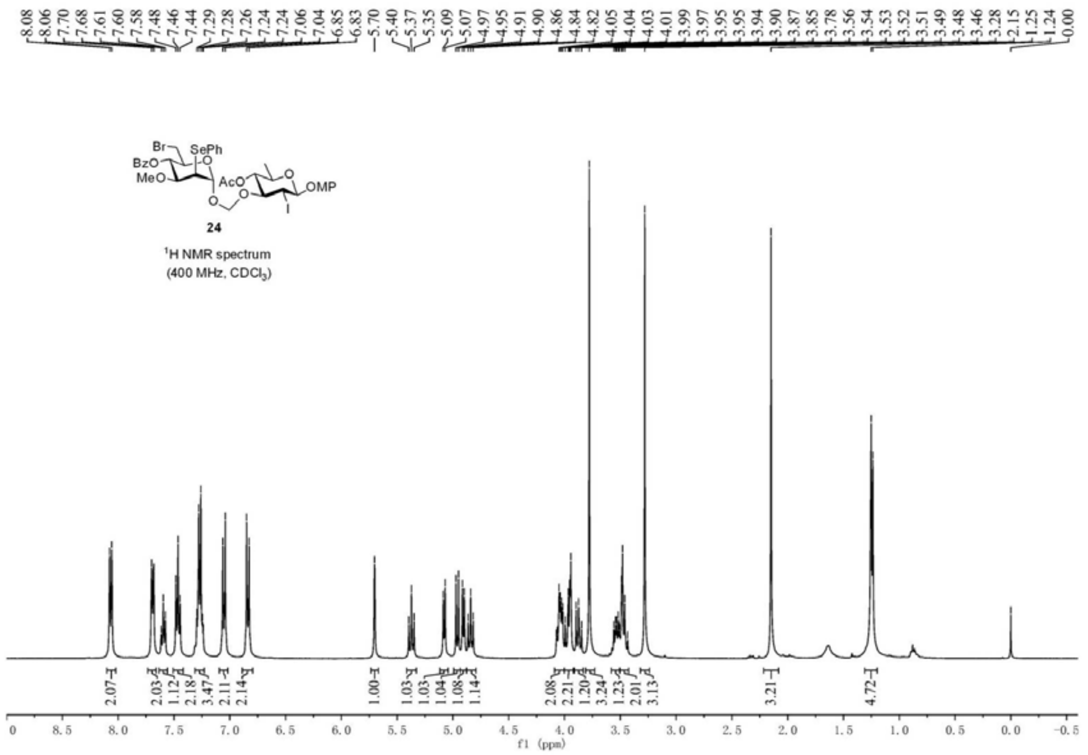

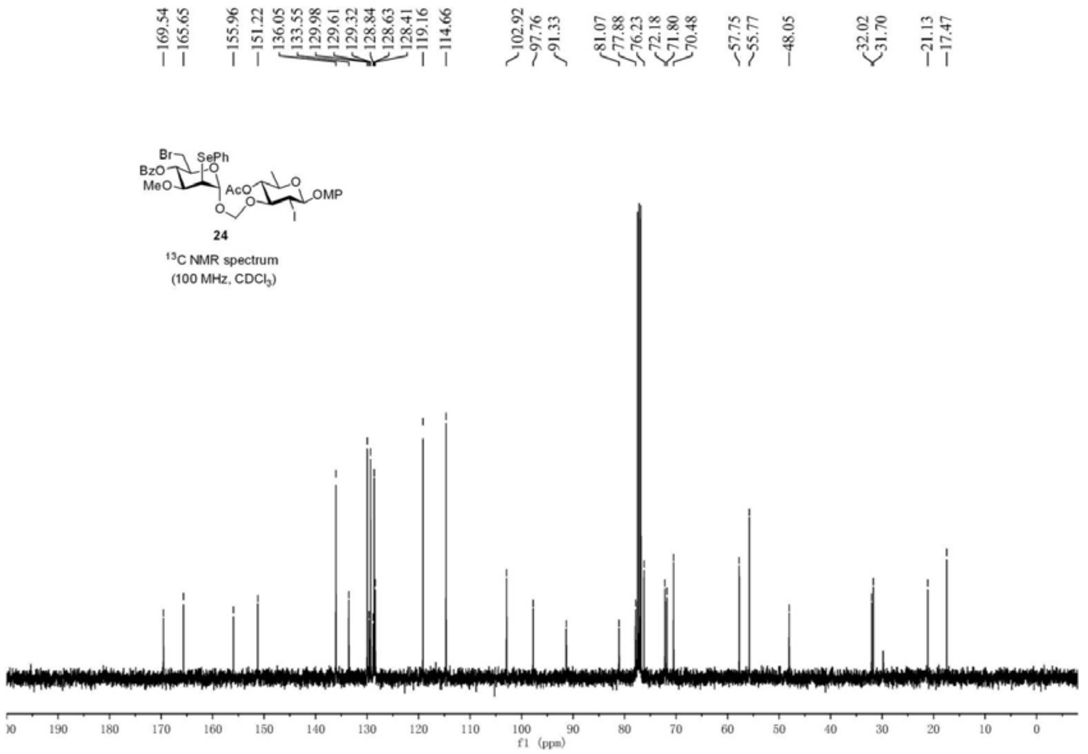

Supplementary Figure 28. ^{1}H and ^{13}C NMR spectra for compound 24.

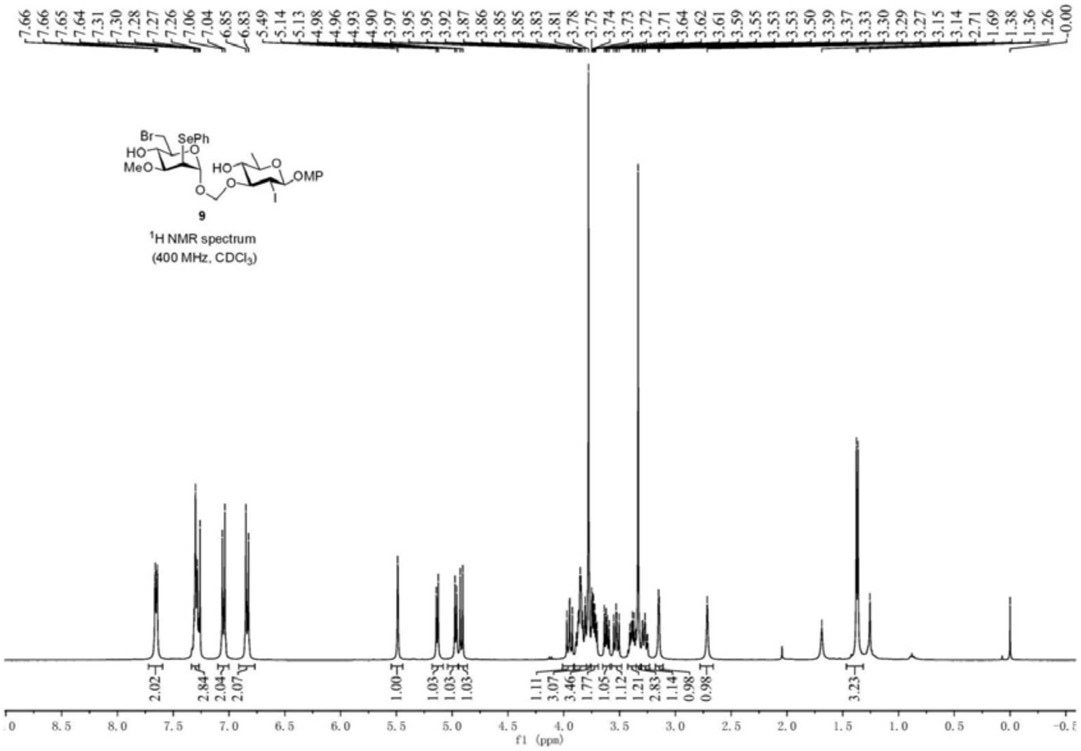

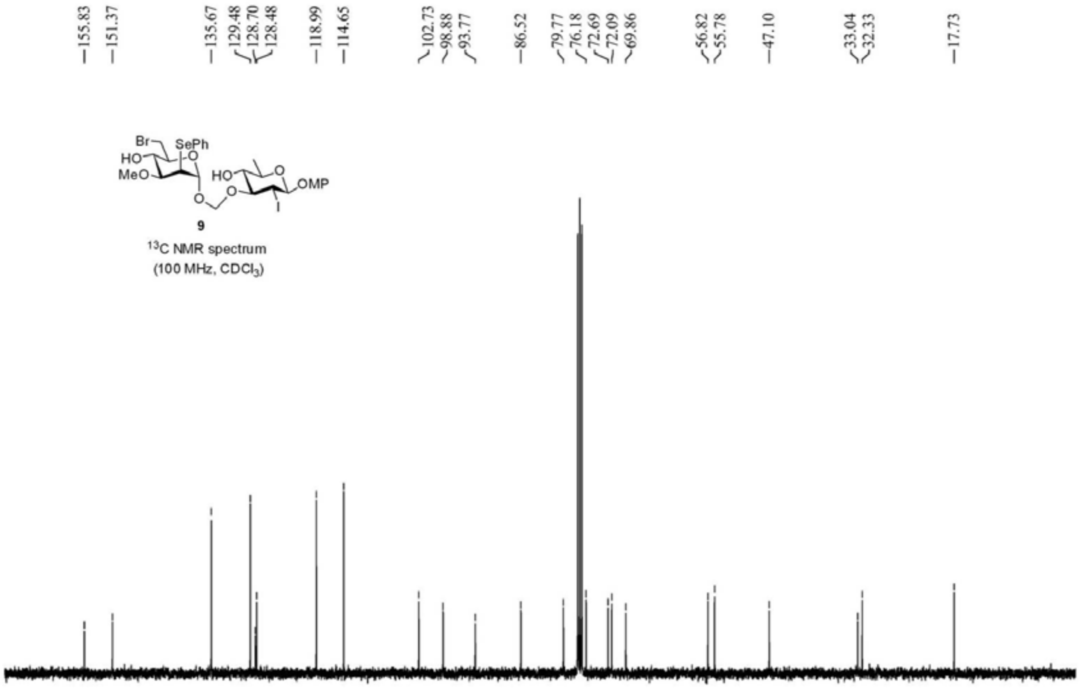

Supplementary Figure 29. ^{1}H and ^{13}C NMR spectra for compound 9.

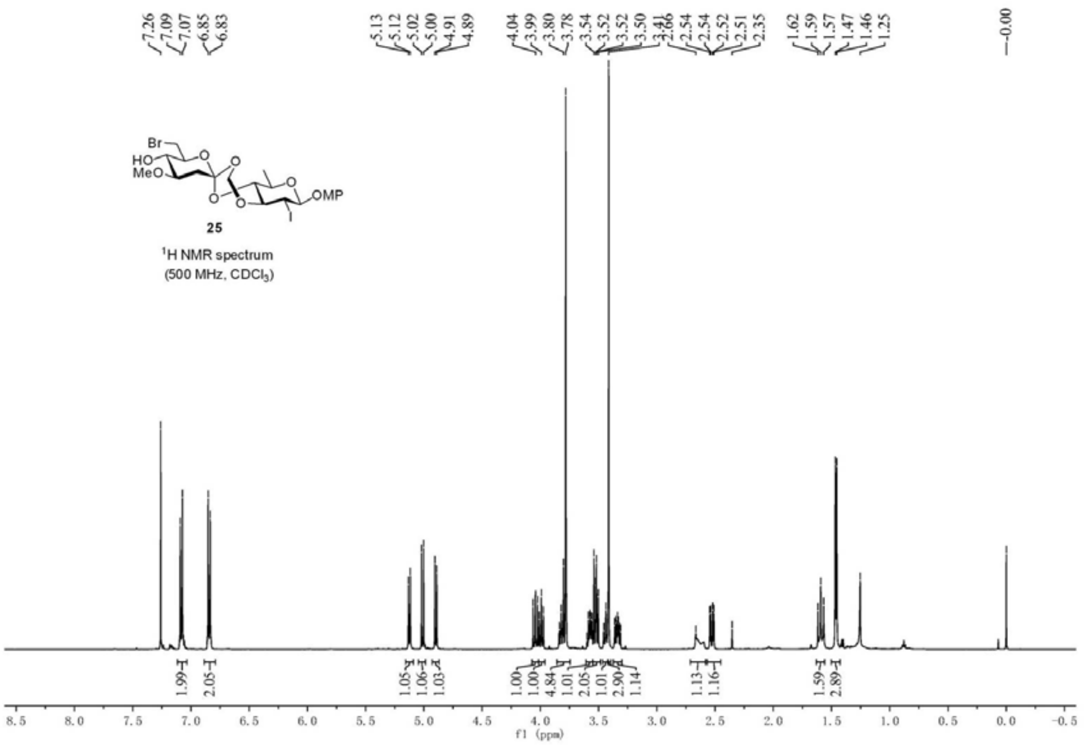

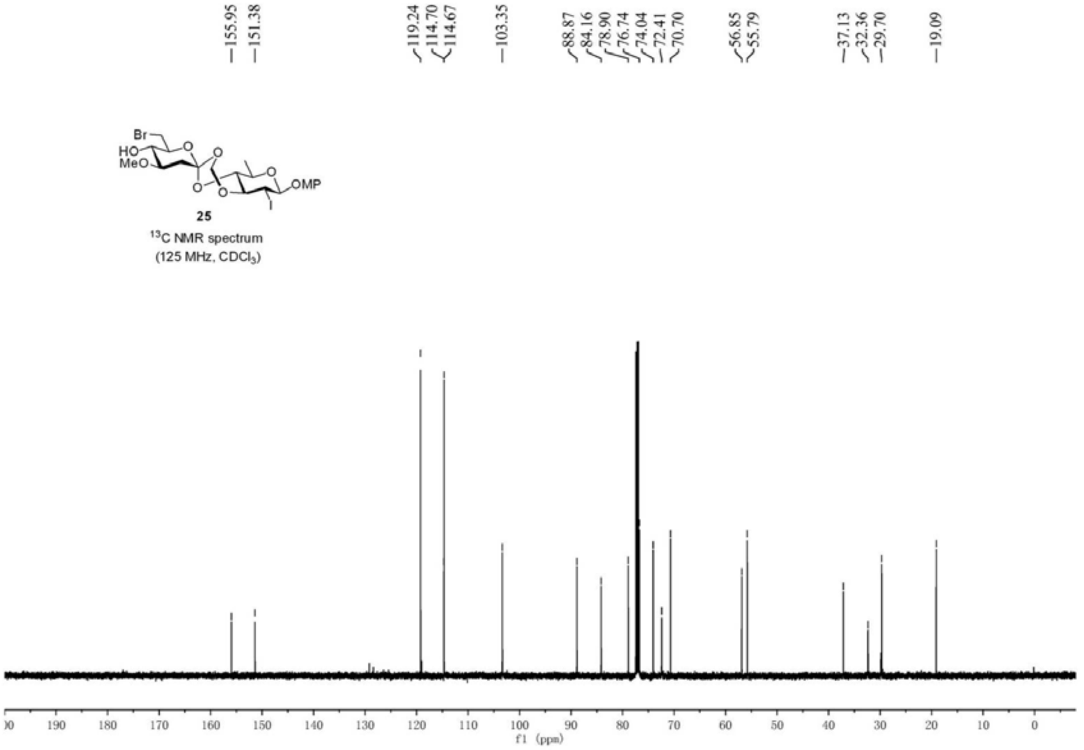

Supplementary Figure 30. ^{1}H and ^{13}C NMR spectra for compound 25.

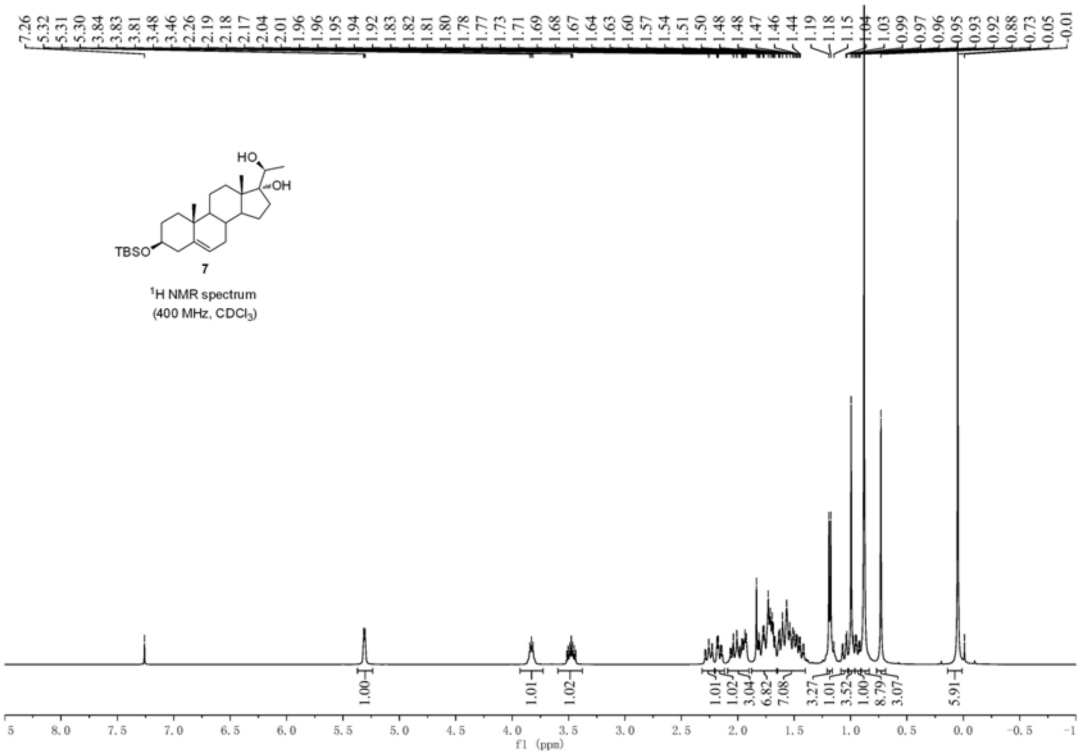

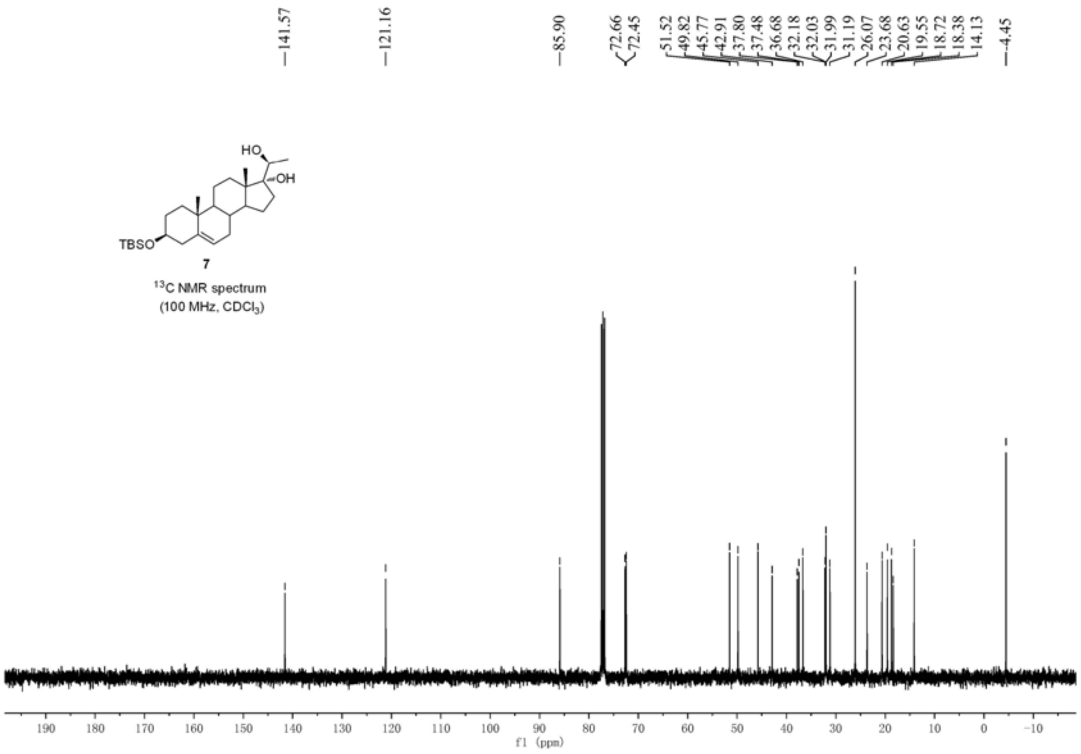

Supplementary Figure 31. ^{1}H and ^{13}C NMR spectra for compound 7.

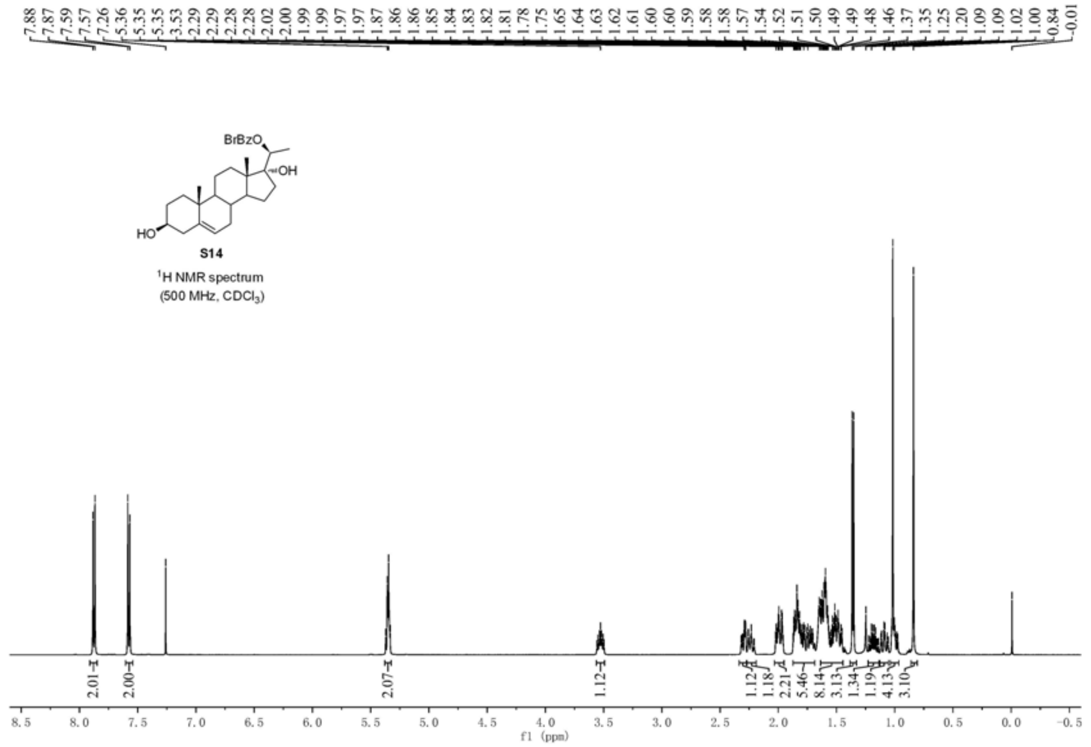

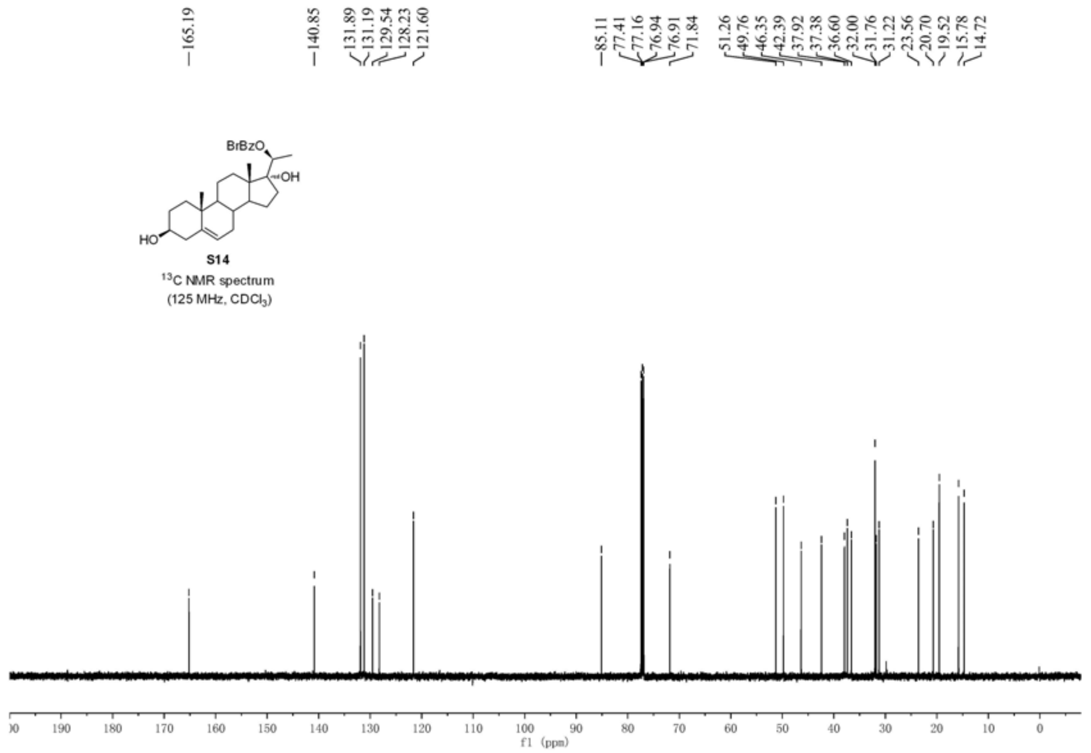

Supplementary Figure 32. ^{1}H and ^{13}C NMR spectra for compound S14.

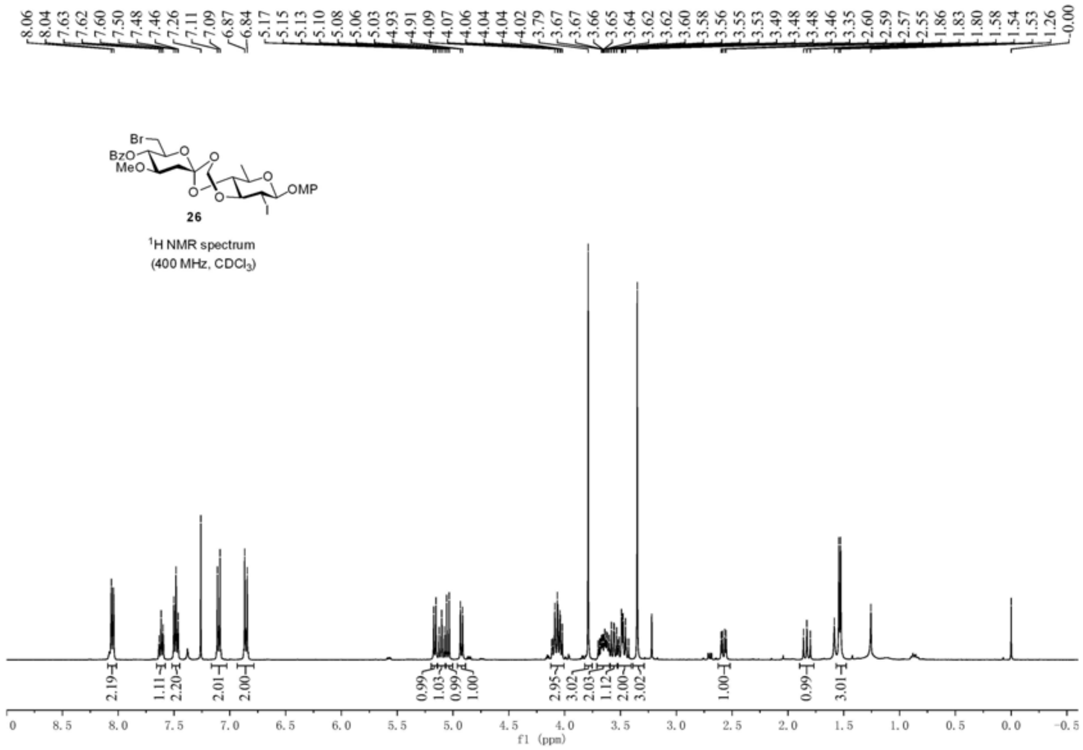

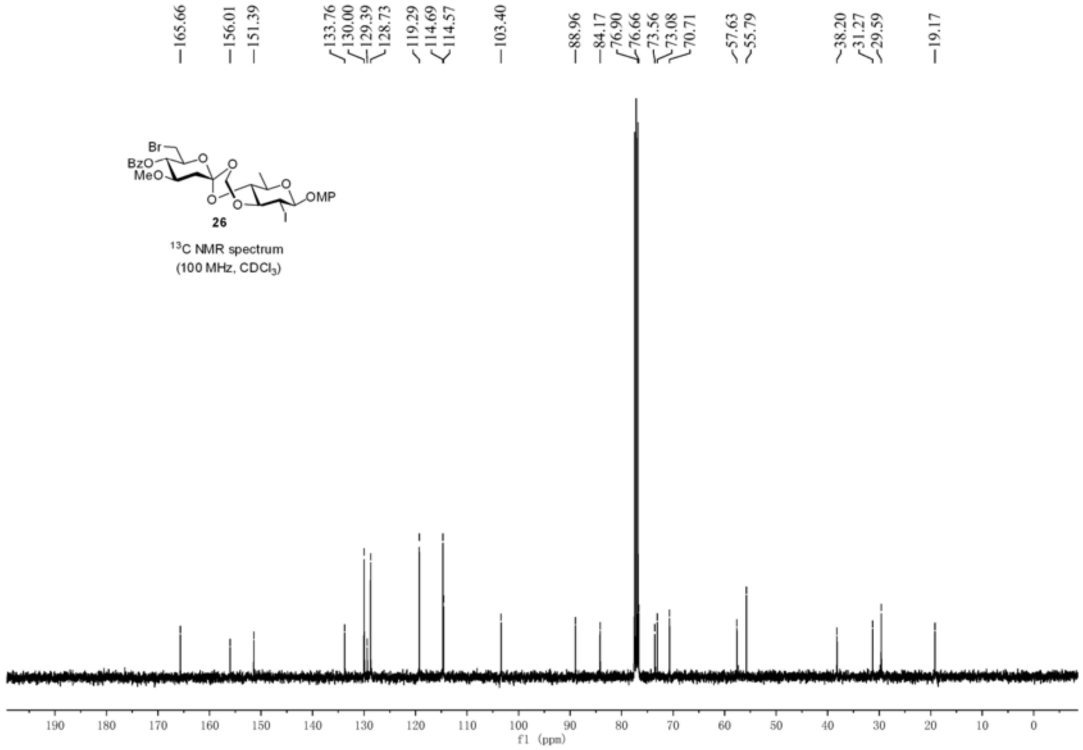

Supplementary Figure 33. ^{1}H and ^{13}C NMR spectra for compound 26.

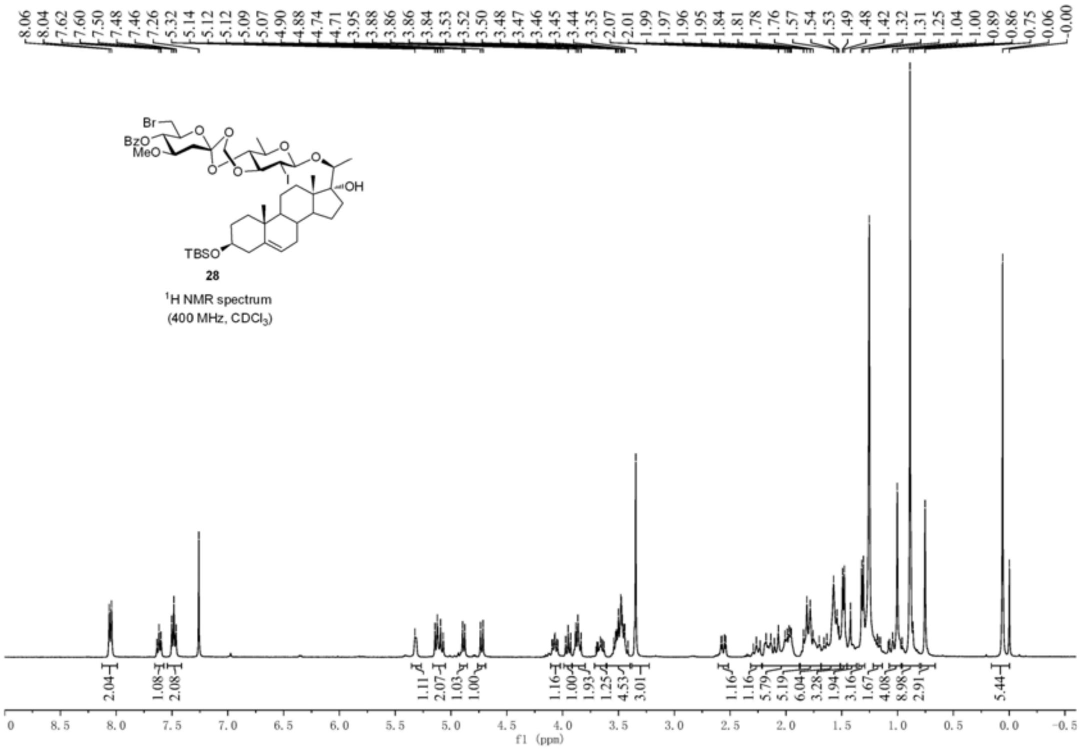

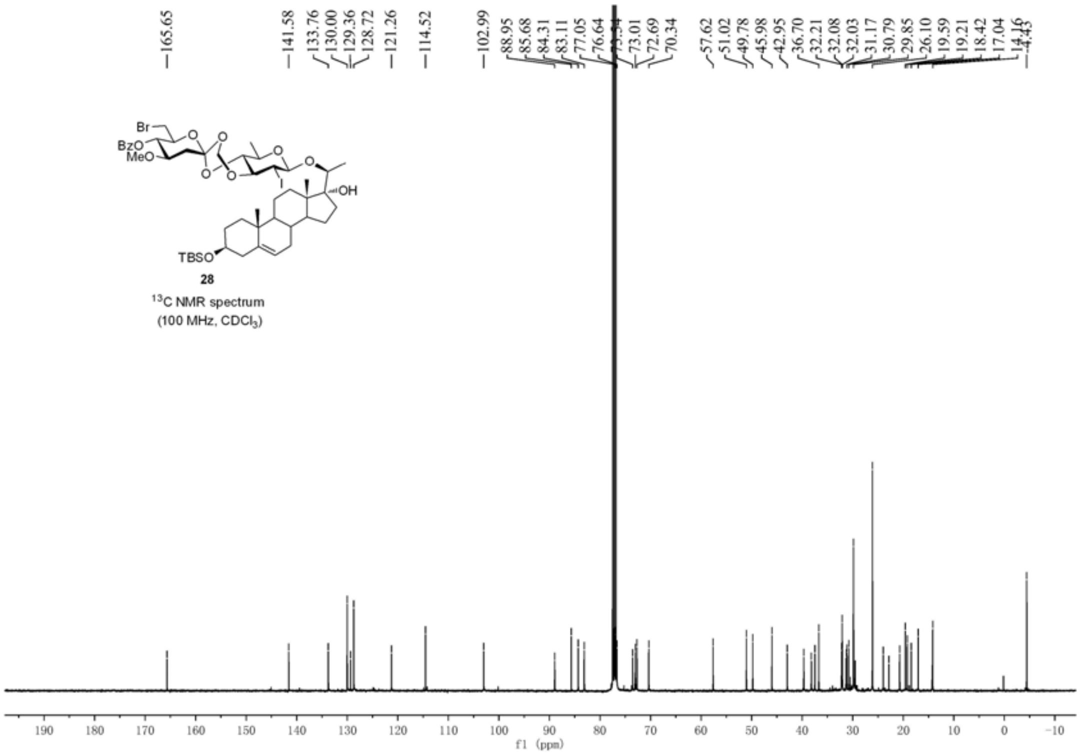

Supplementary Figure 34. ^{1}H and ^{13}C NMR spectra for compound 28.

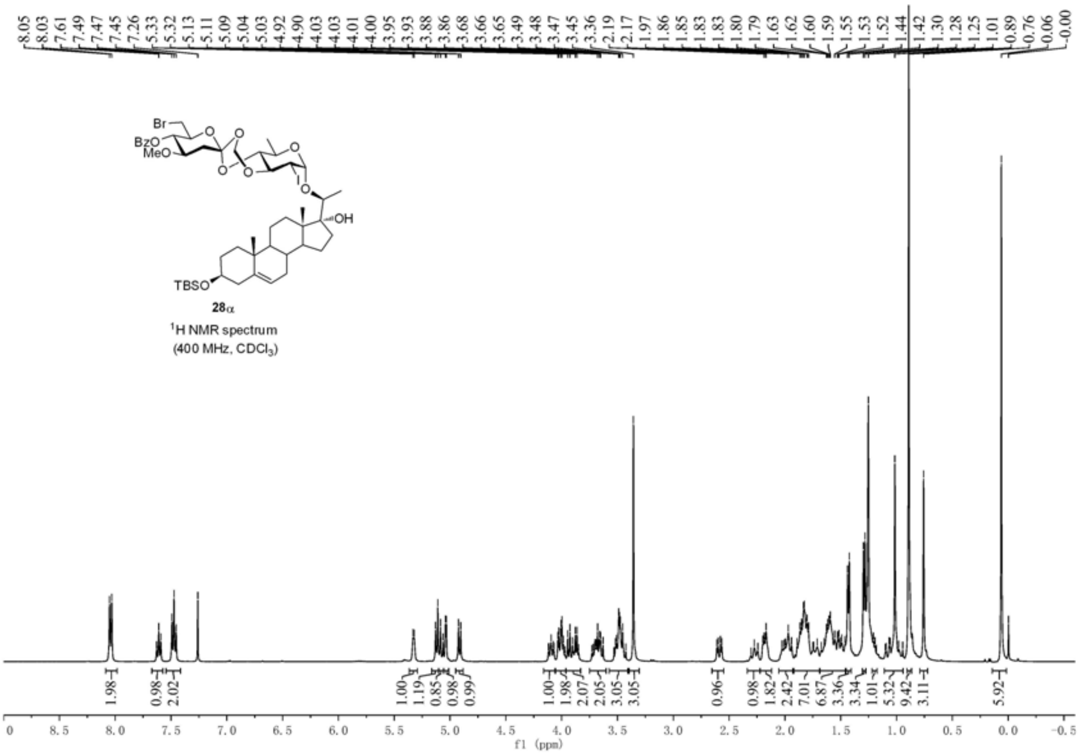

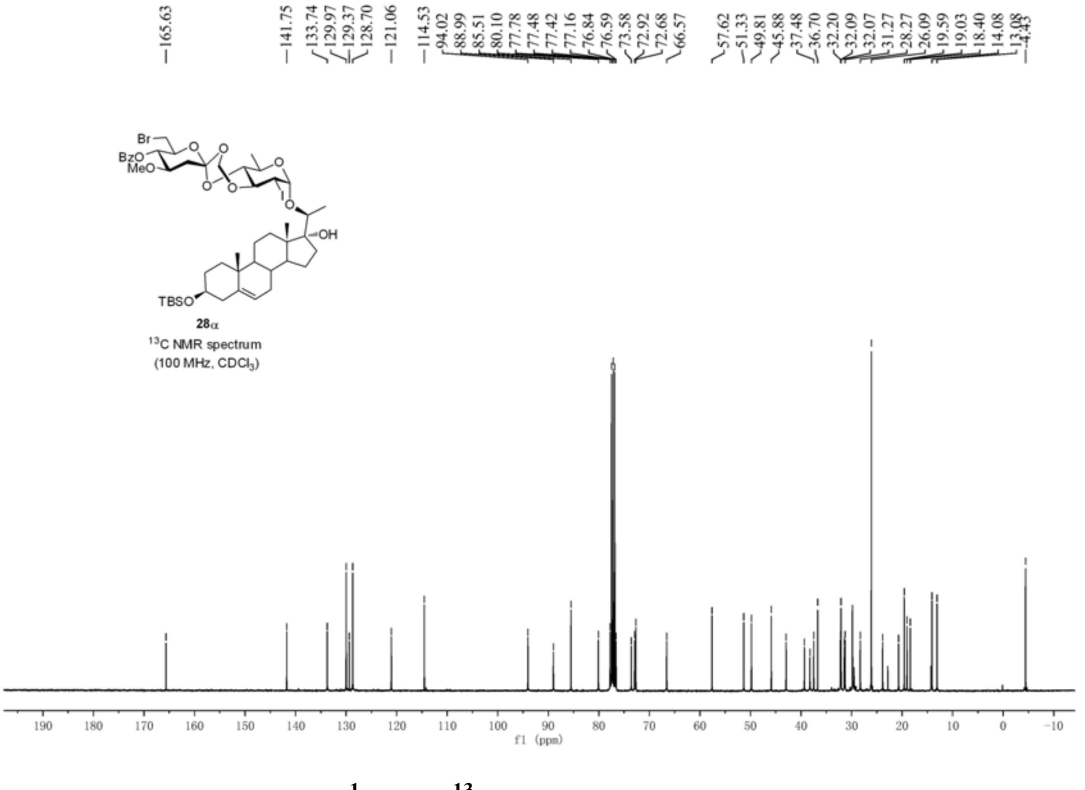

Supplementary Figure 35. ^{1}**H and** ^{13}**C NMR spectra for compound 28α.**

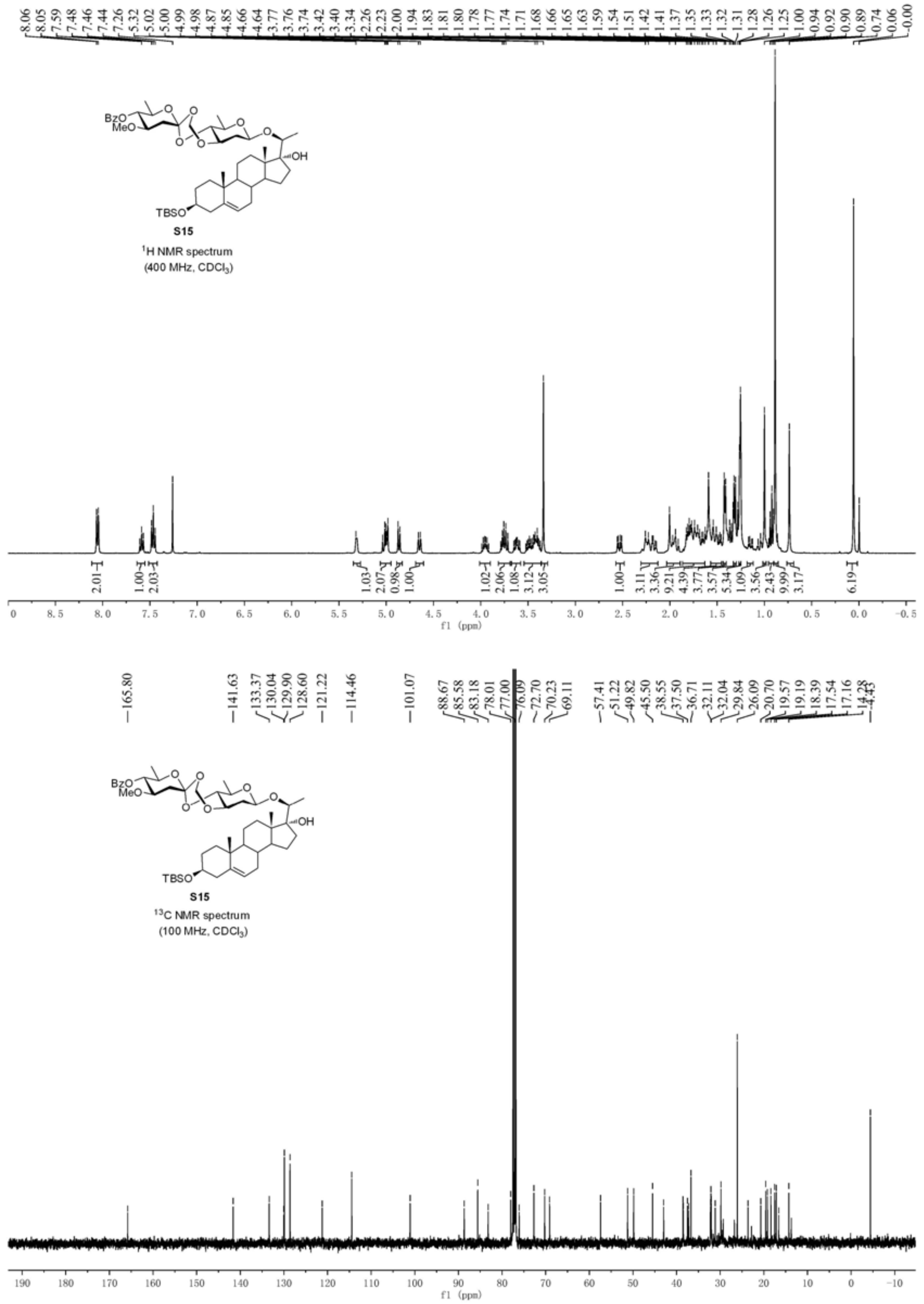

Supplementary Figure 36. ^{1}H and ^{13}C NMR spectra for compound S15.

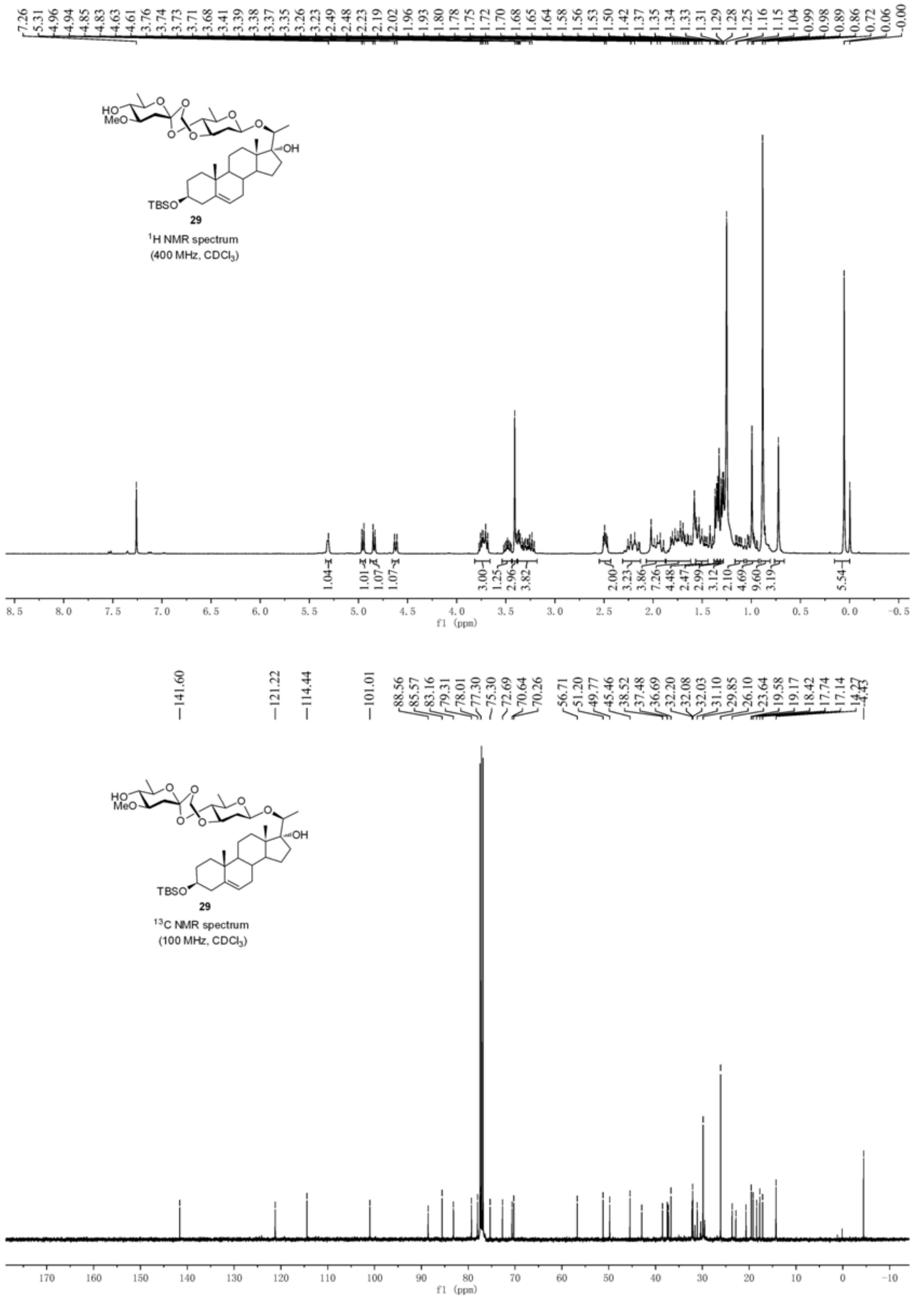

Supplementary Figure 37. ^{1}H and ^{13}C NMR spectra for compound 29.

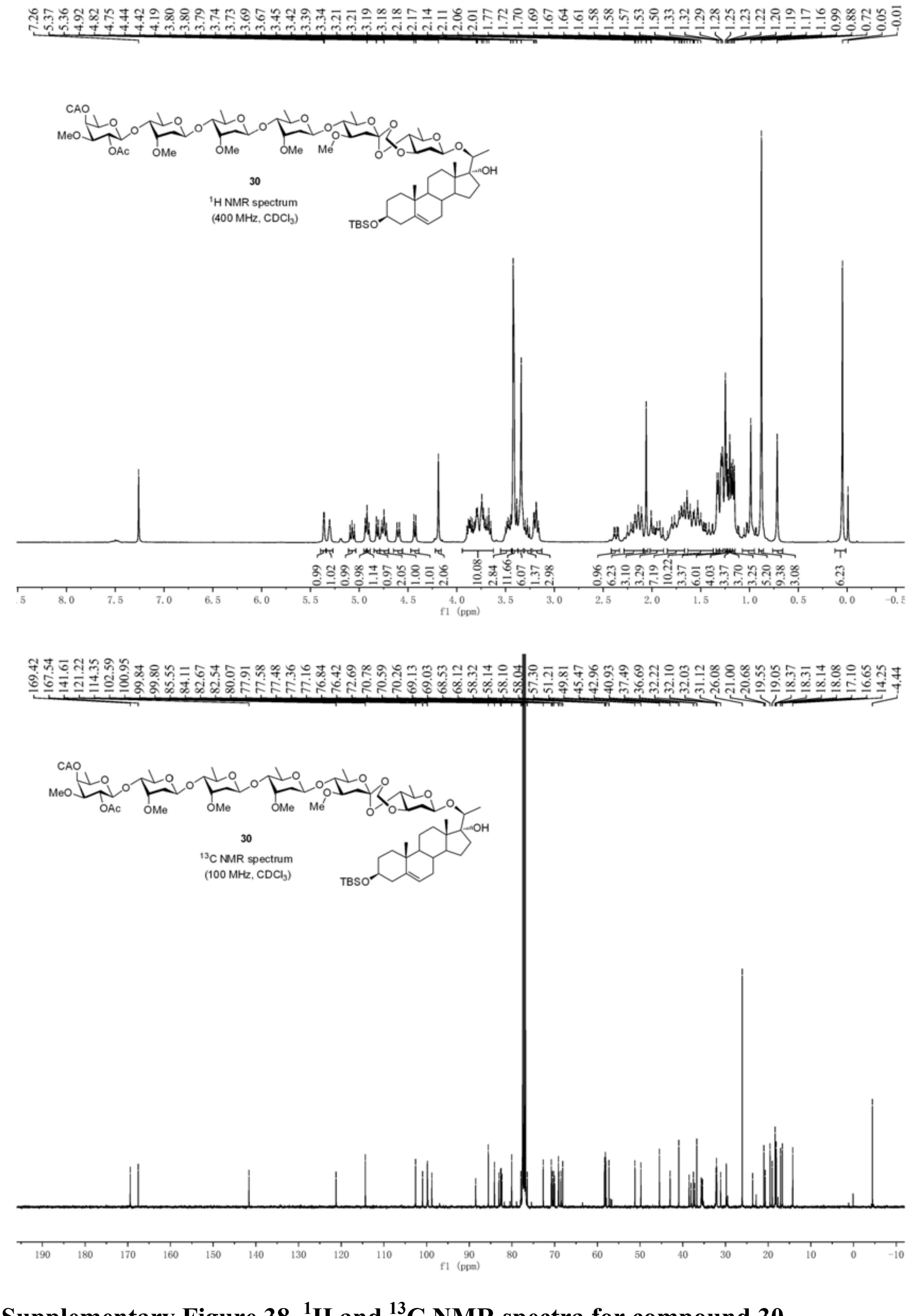

Supplementary Figure 38. ^{1}H and ^{13}C NMR spectra for compound 30.

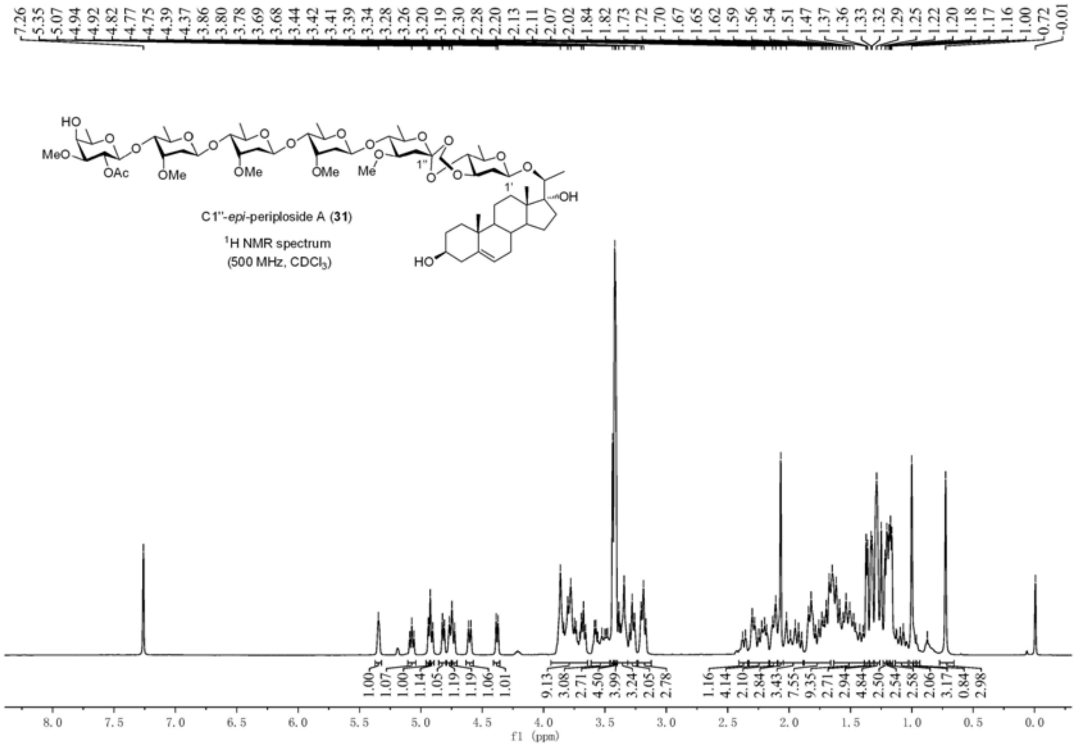

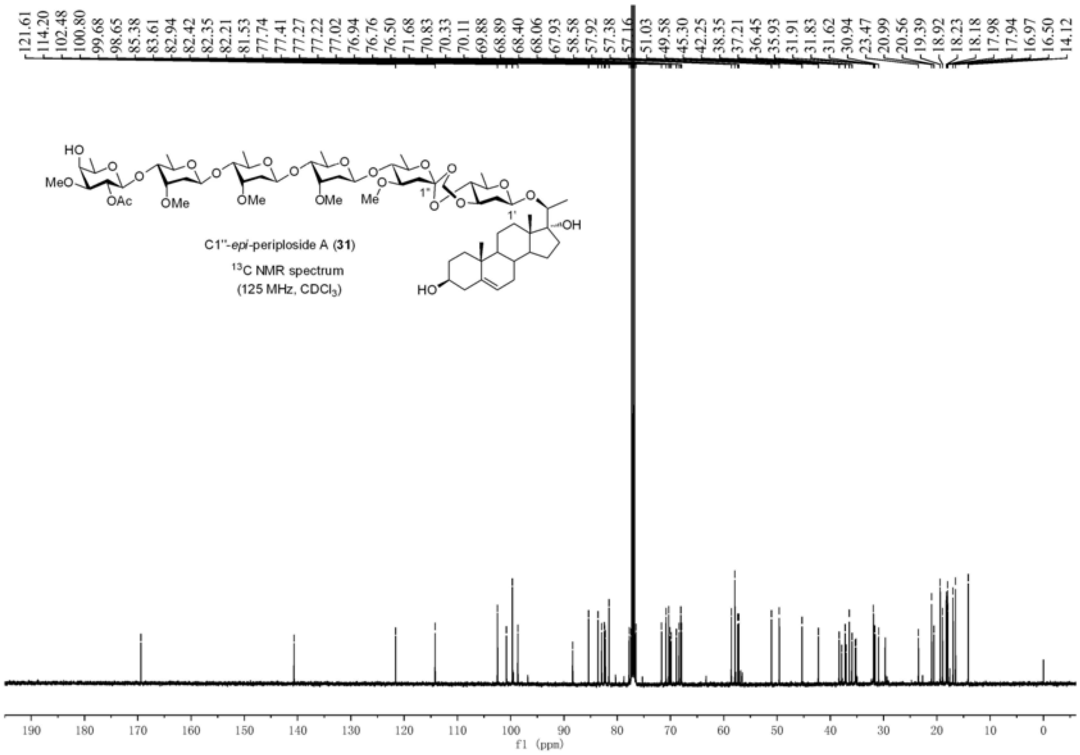

Supplementary Figure 39. ^{1}H and ^{13}C NMR spectra for compound 31.

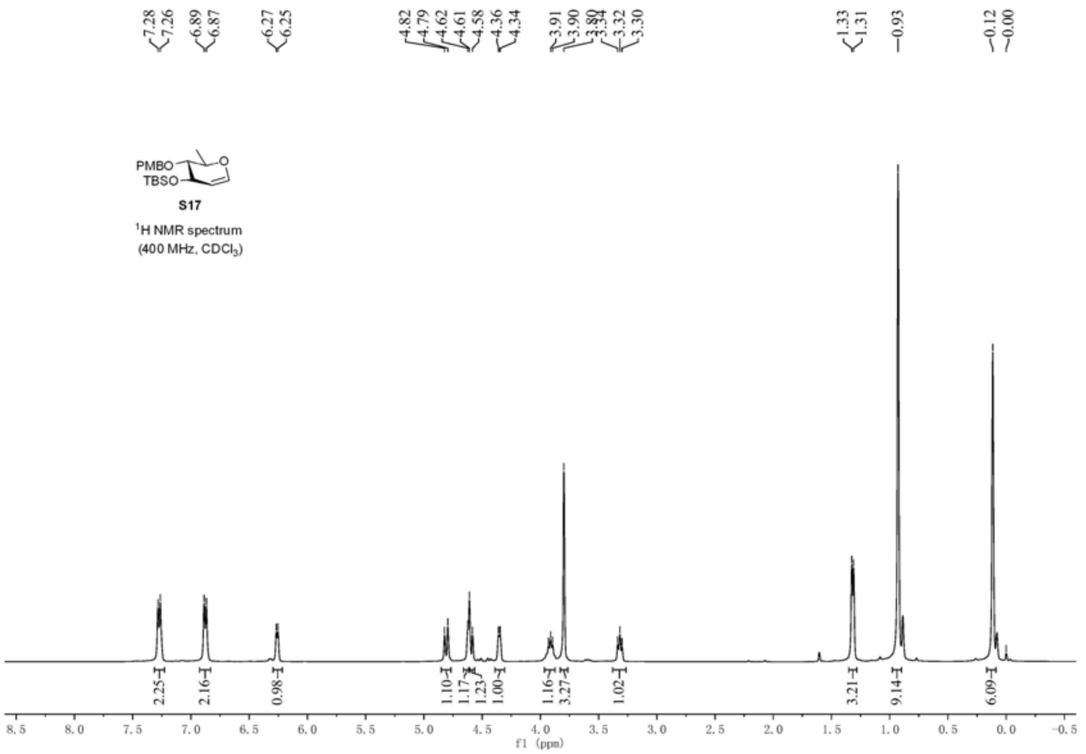

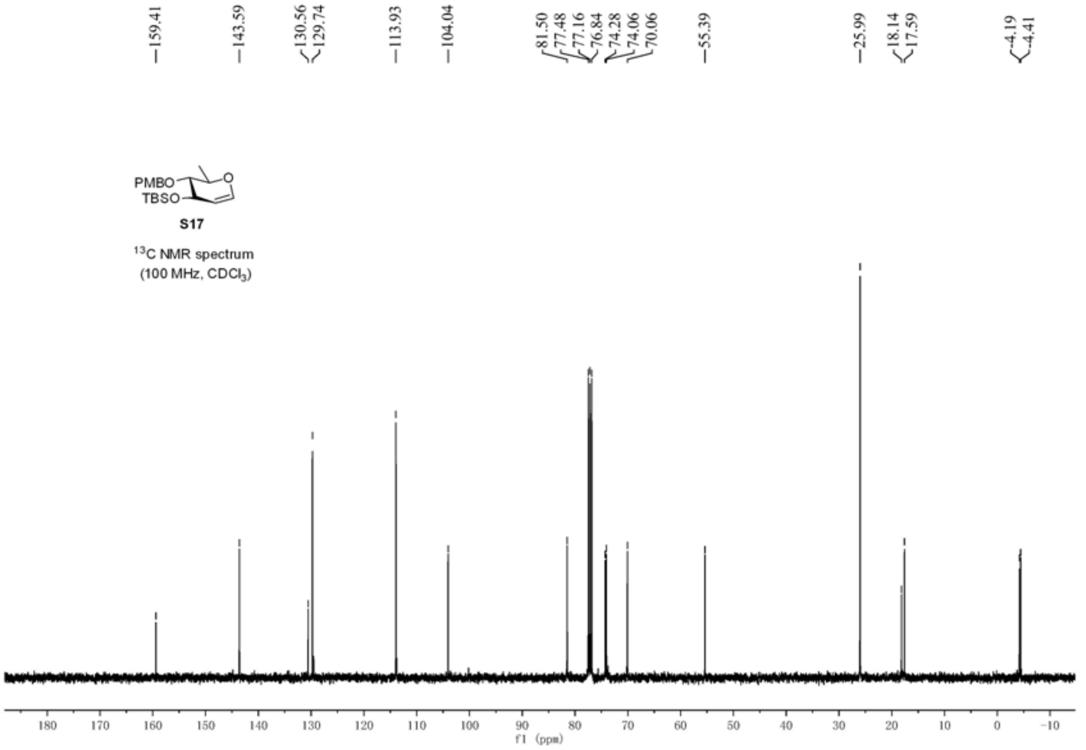

Supplementary Figure 40. ^{1}H and ^{13}C NMR spectra for compound S17.

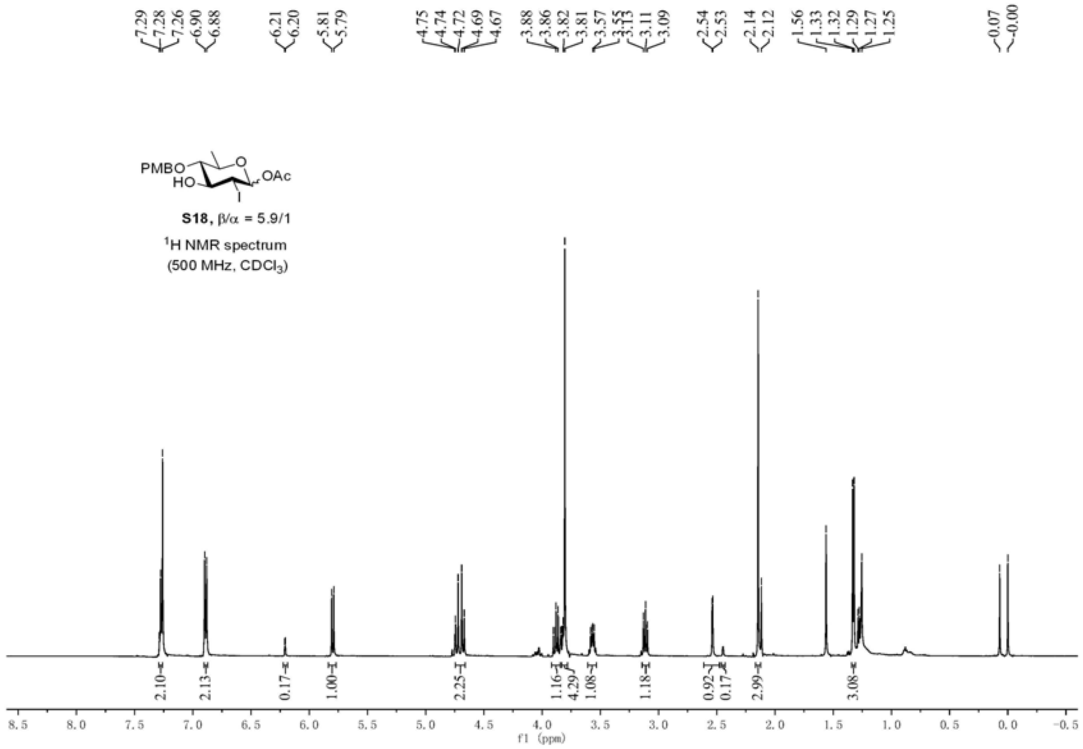

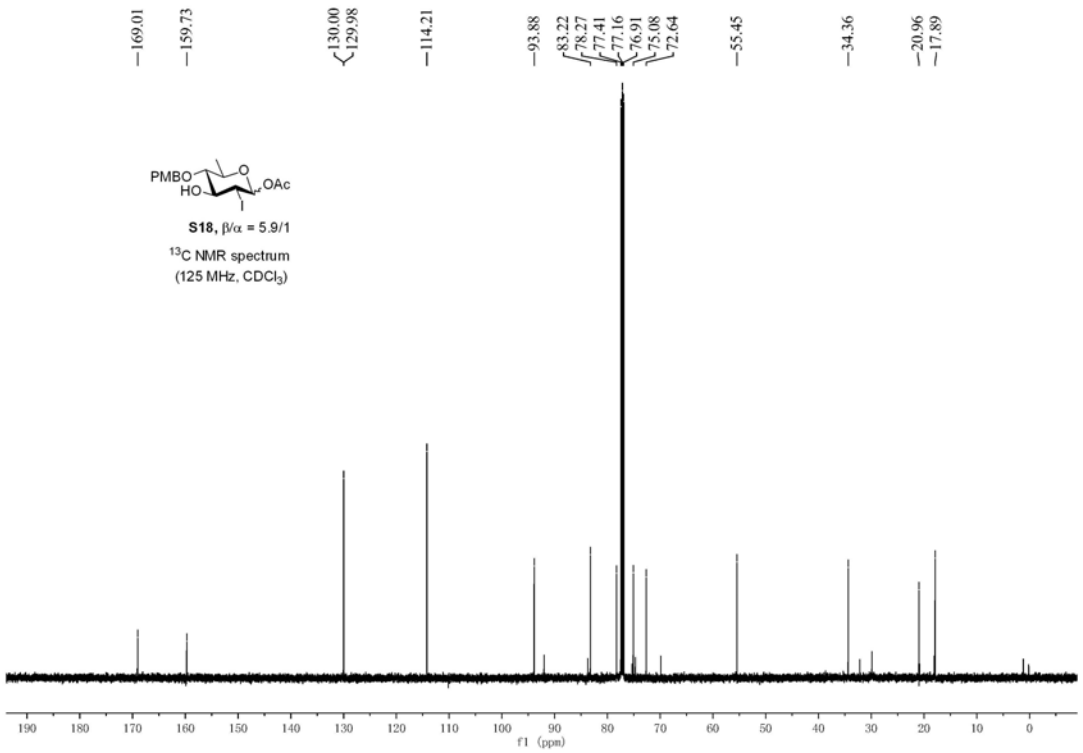

Supplementary Figure 41. ^{1}H and ^{13}C NMR spectra for compound S18.

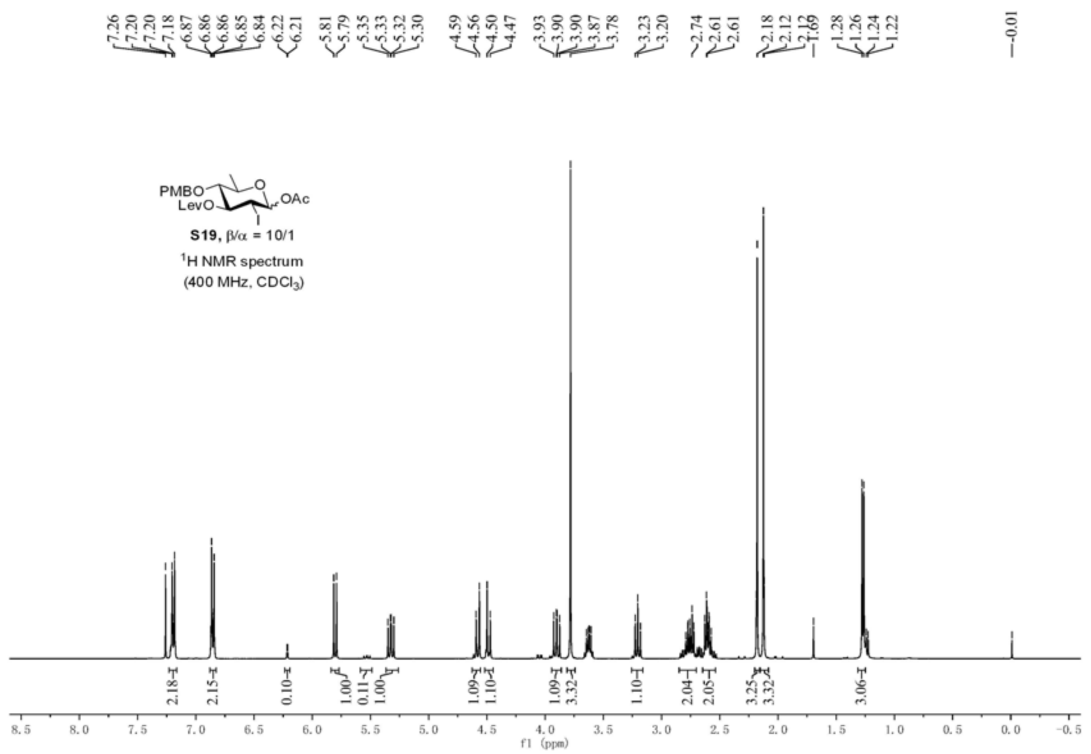

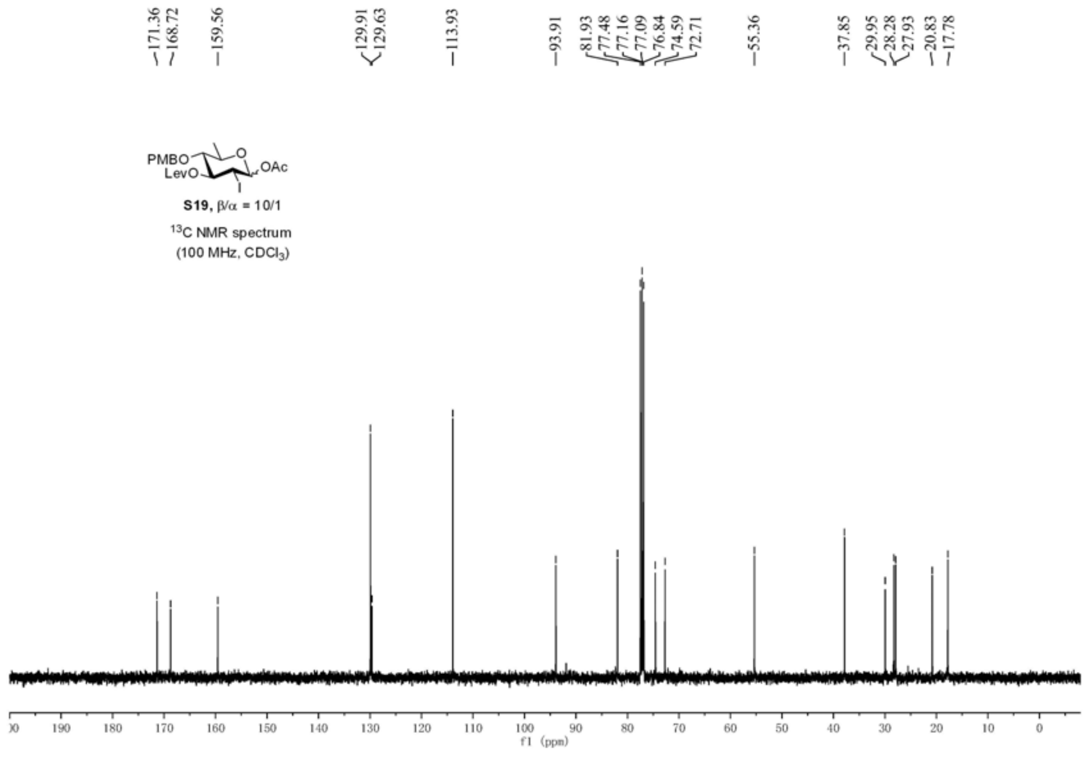

Supplementary Figure 42. ^{1}H and ^{13}C NMR spectra for compound S19.

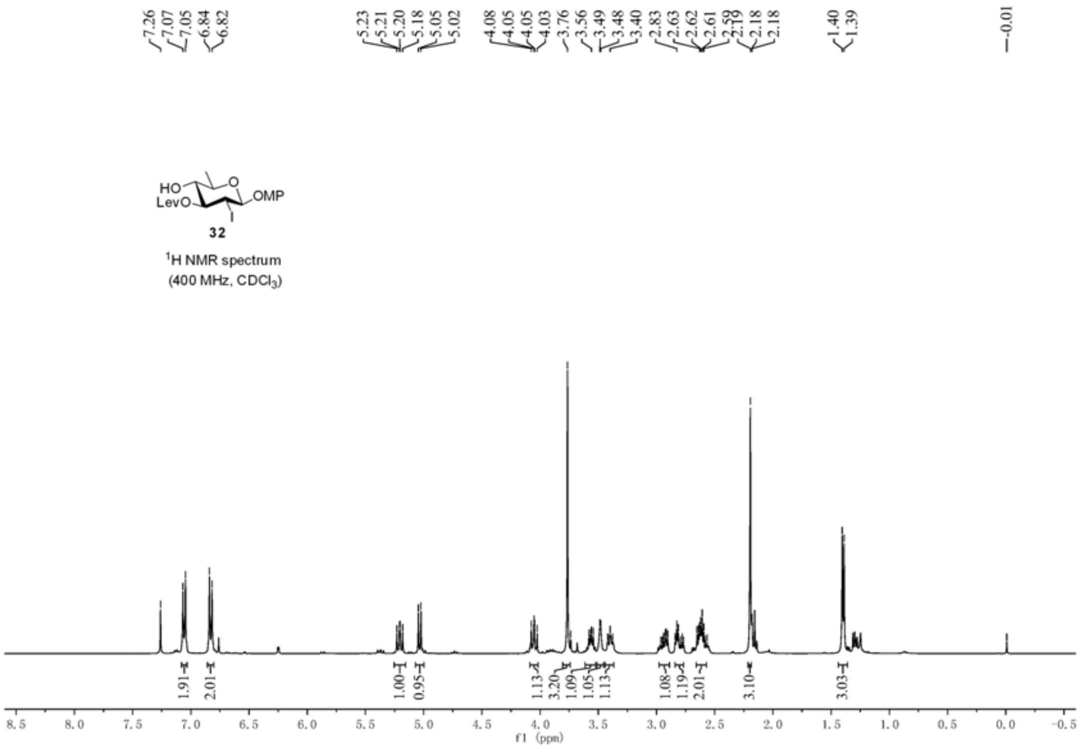

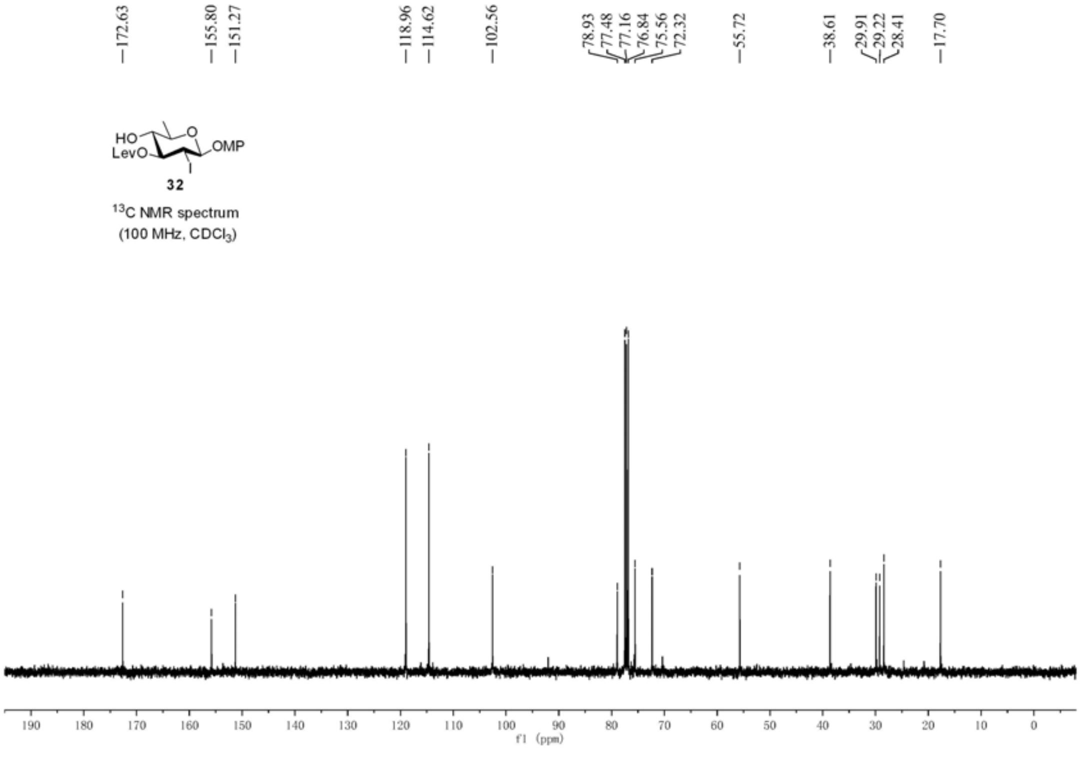

Supplementary Figure 43. ^{1}H and ^{13}C NMR spectra for compound 32.

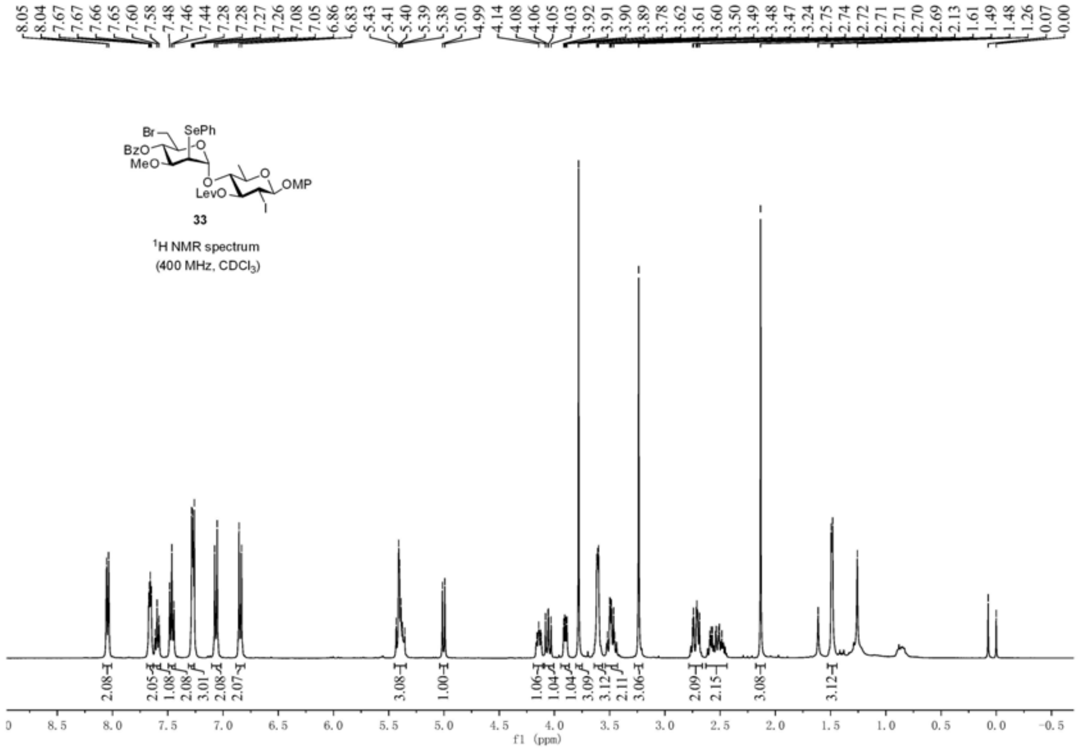

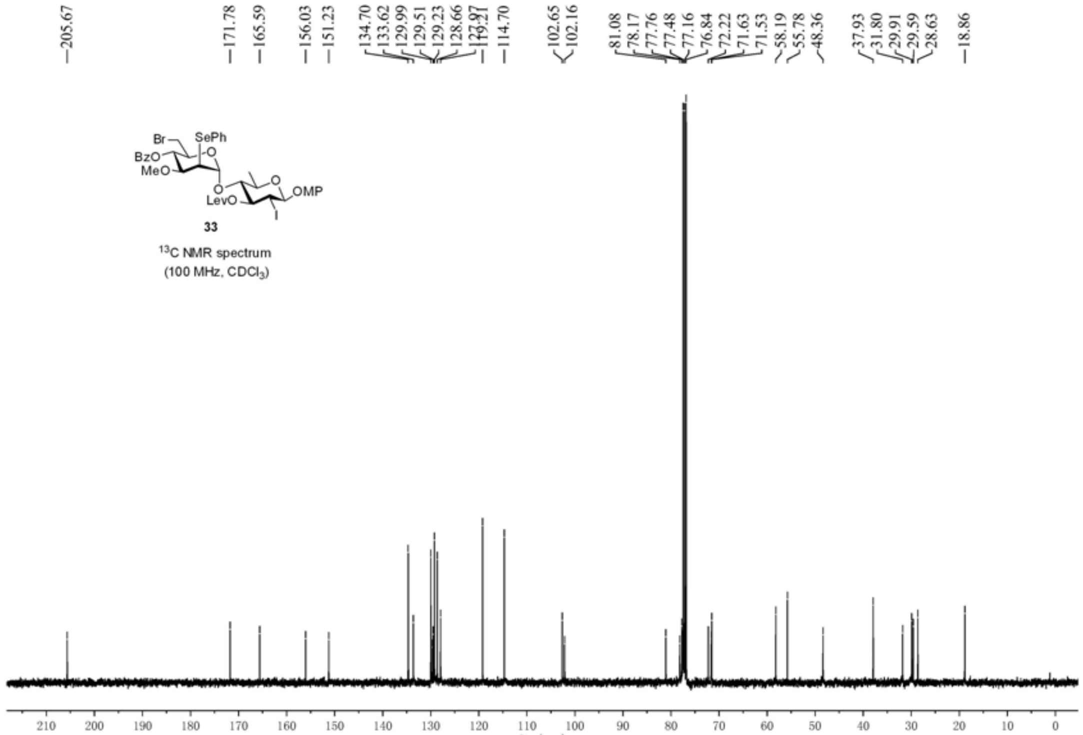

Supplementary Figure 44. ^{1}H and ^{13}C NMR spectra for compound 33.

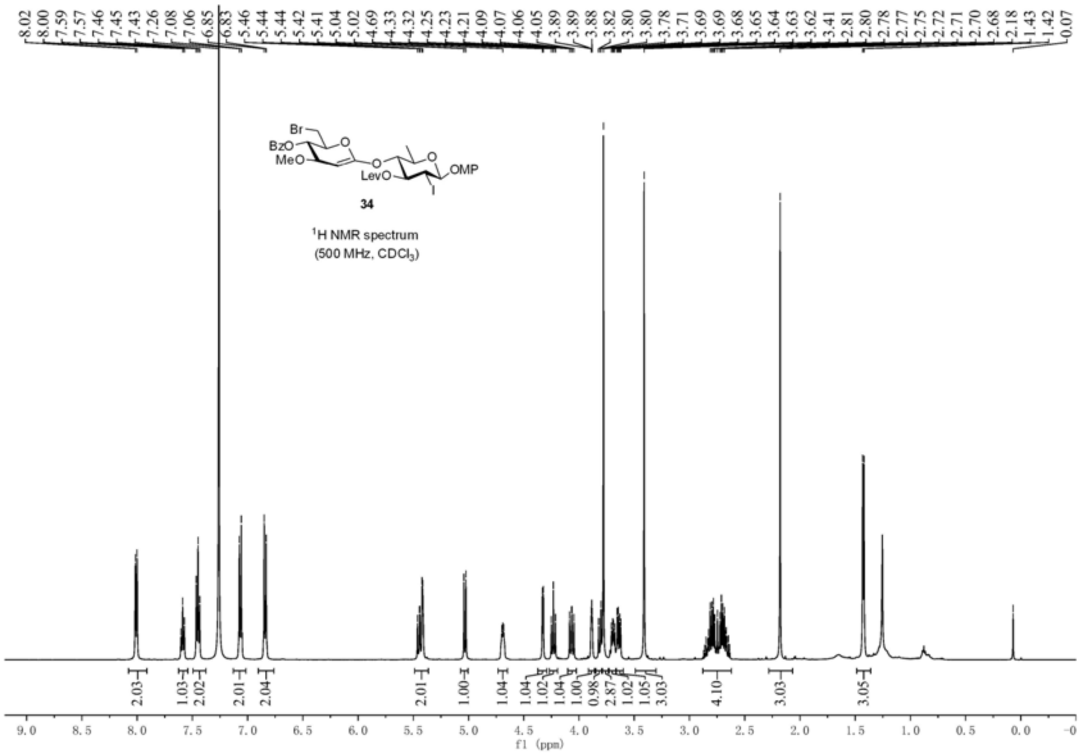

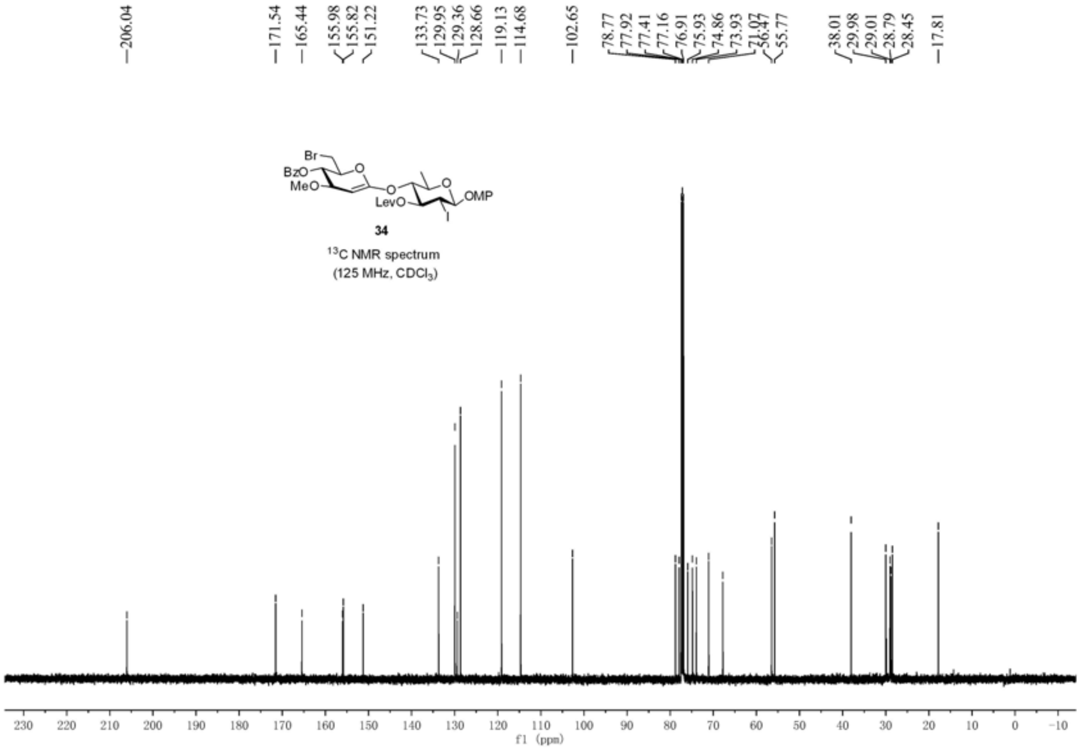

Supplementary Figure 45. ^{1}H and ^{13}C NMR spectra for compound 34.

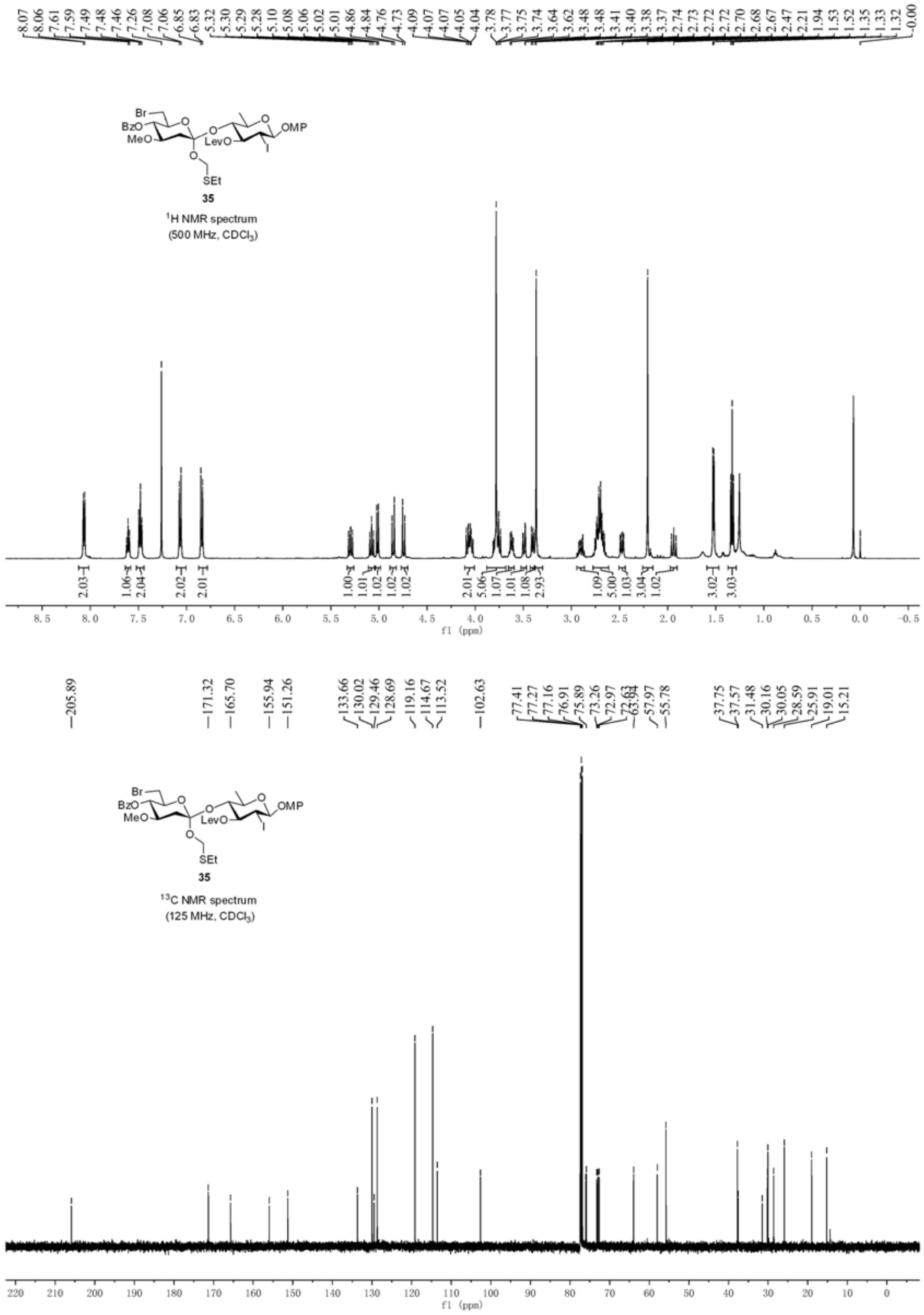

Supplementary Figure 46. ^{1}H and ^{13}C NMR spectra for compound 35.

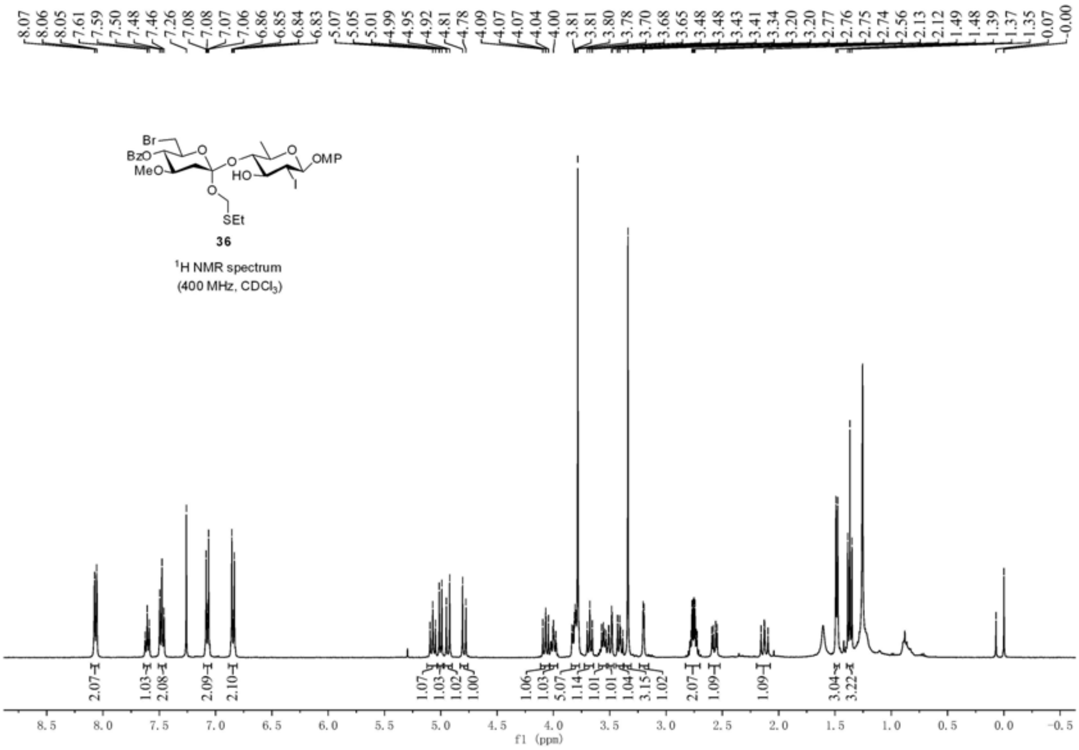

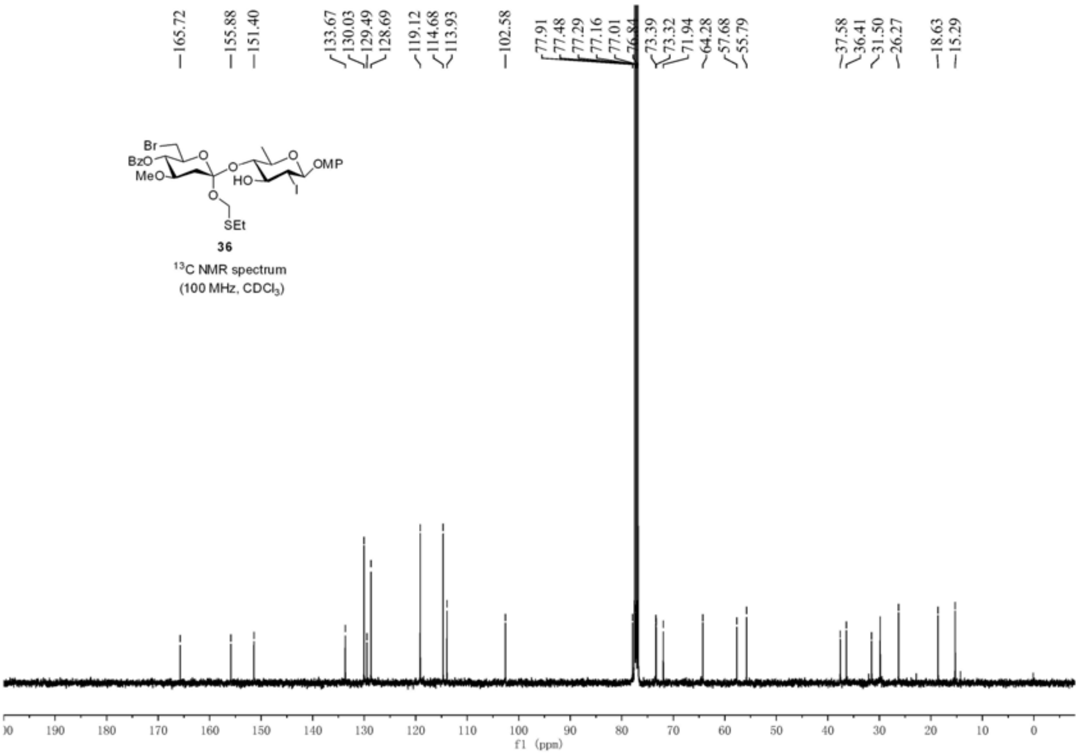

Supplementary Figure 47. ^{1}H and ^{13}C NMR spectra for compound 36.

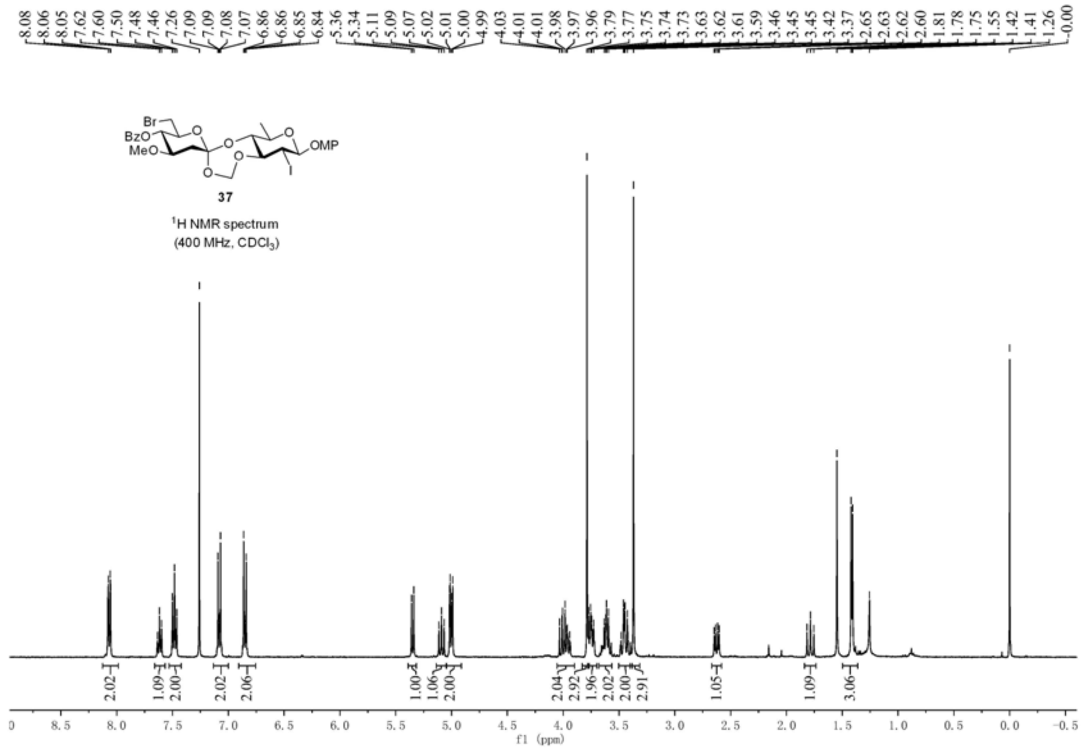

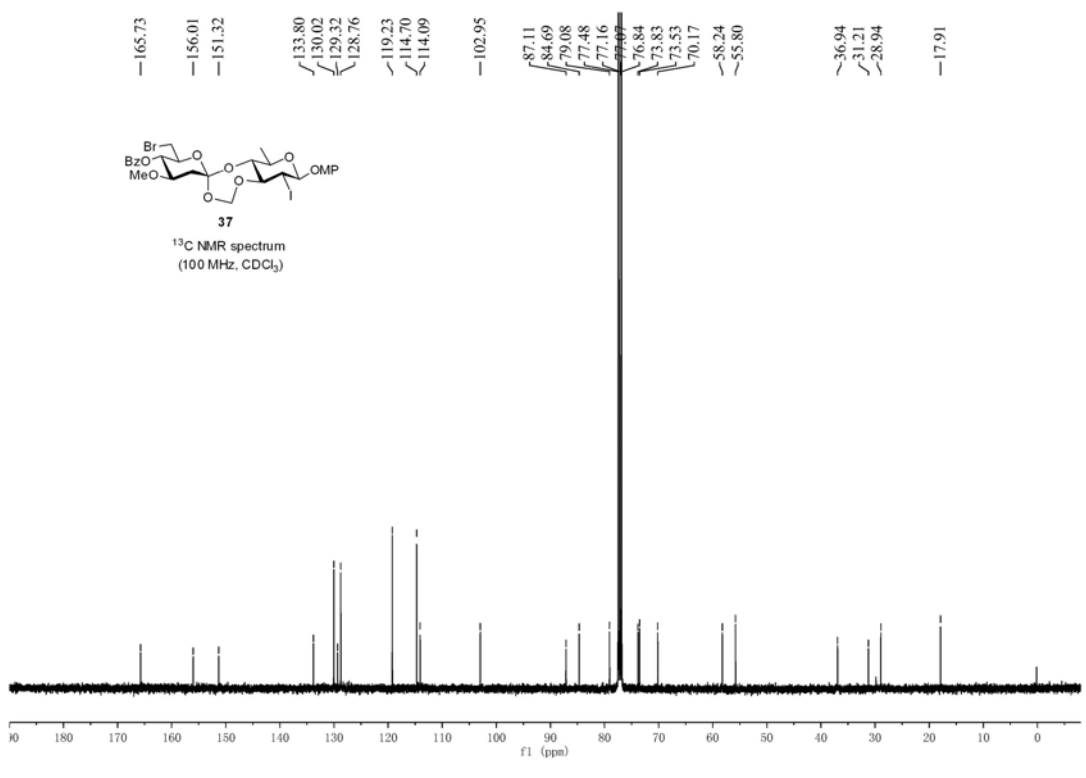

Supplementary Figure 48. ^{1}H and ^{13}C NMR spectra for compound 37.

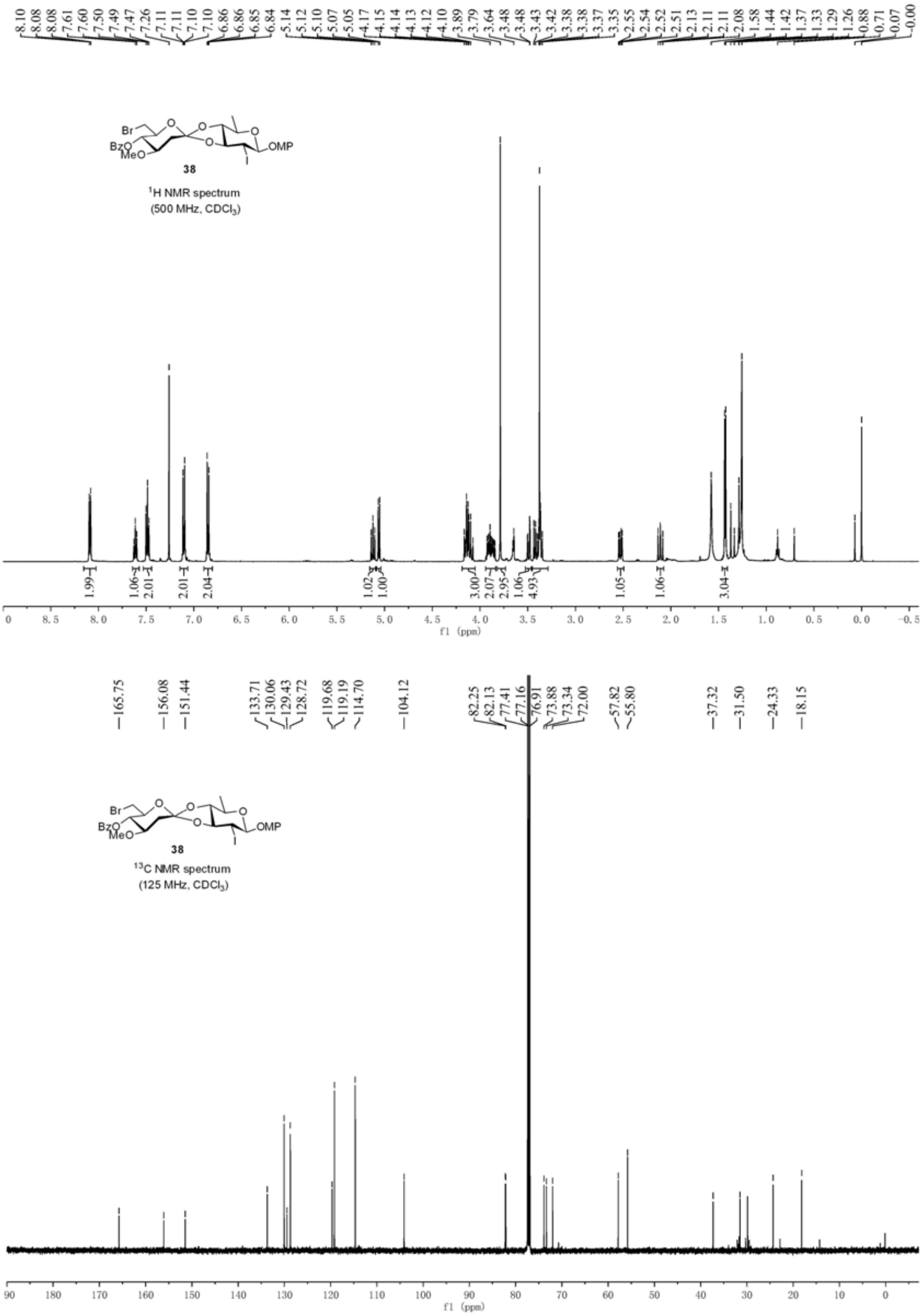

Supplementary Figure 49. ^{1}H and ^{13}C NMR spectra for compound 38.

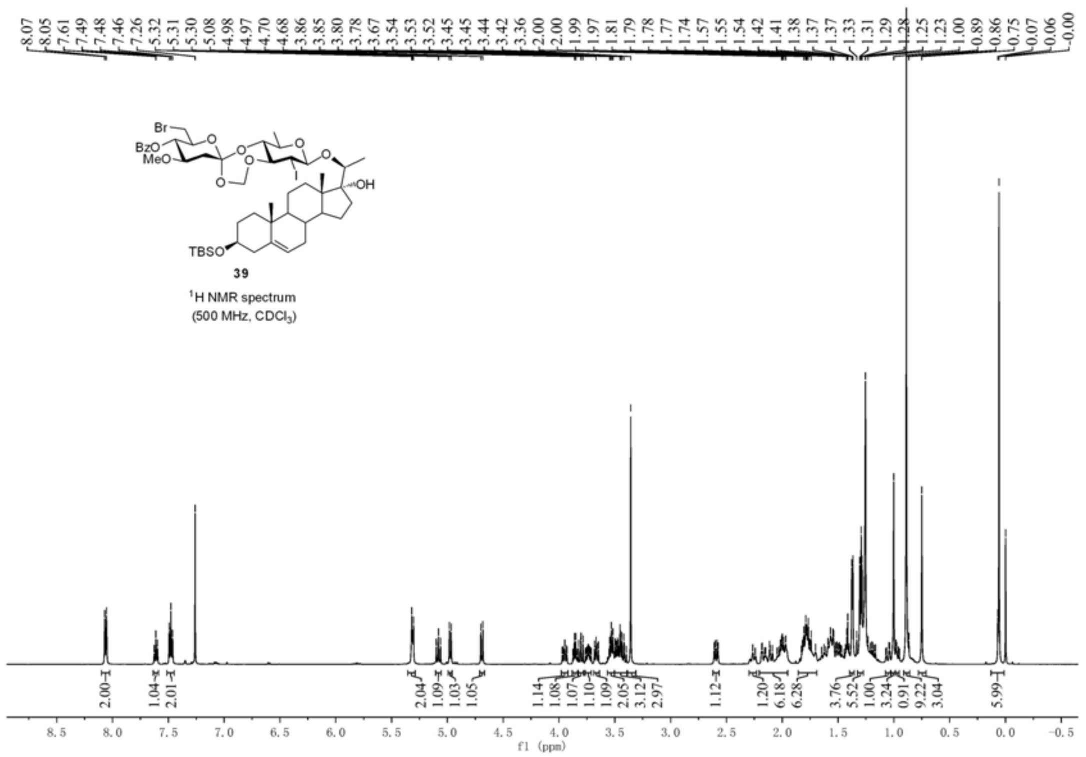

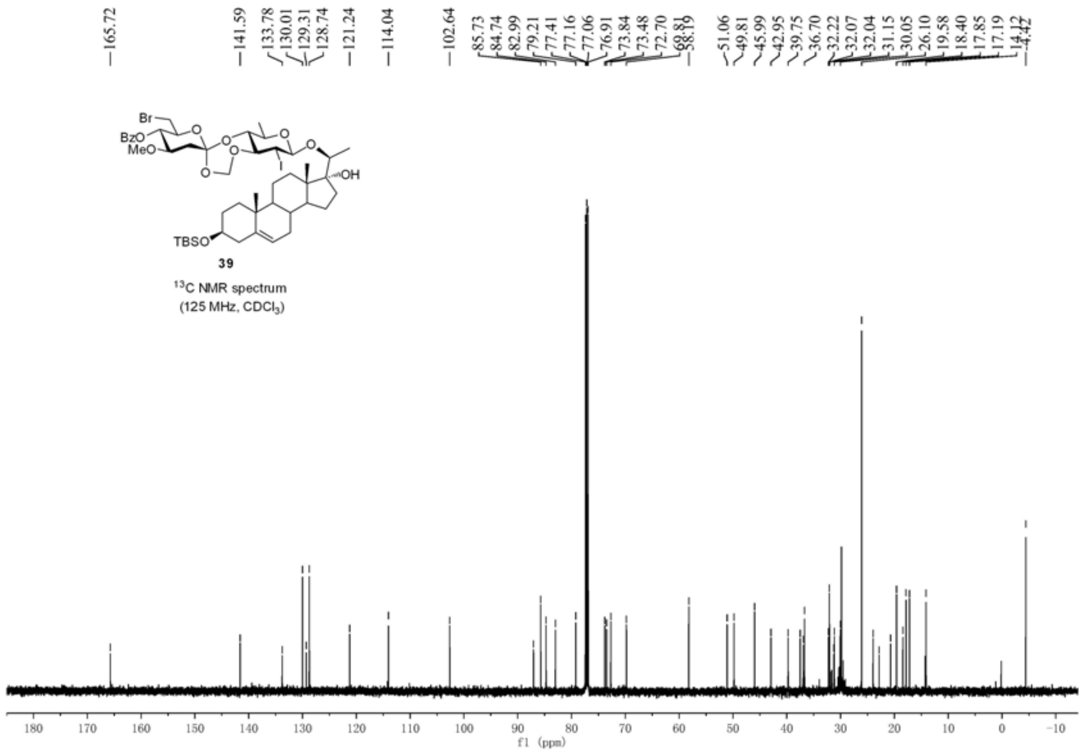

Supplementary Figure 50. ^{1}H and ^{13}C NMR spectra for compound 39.

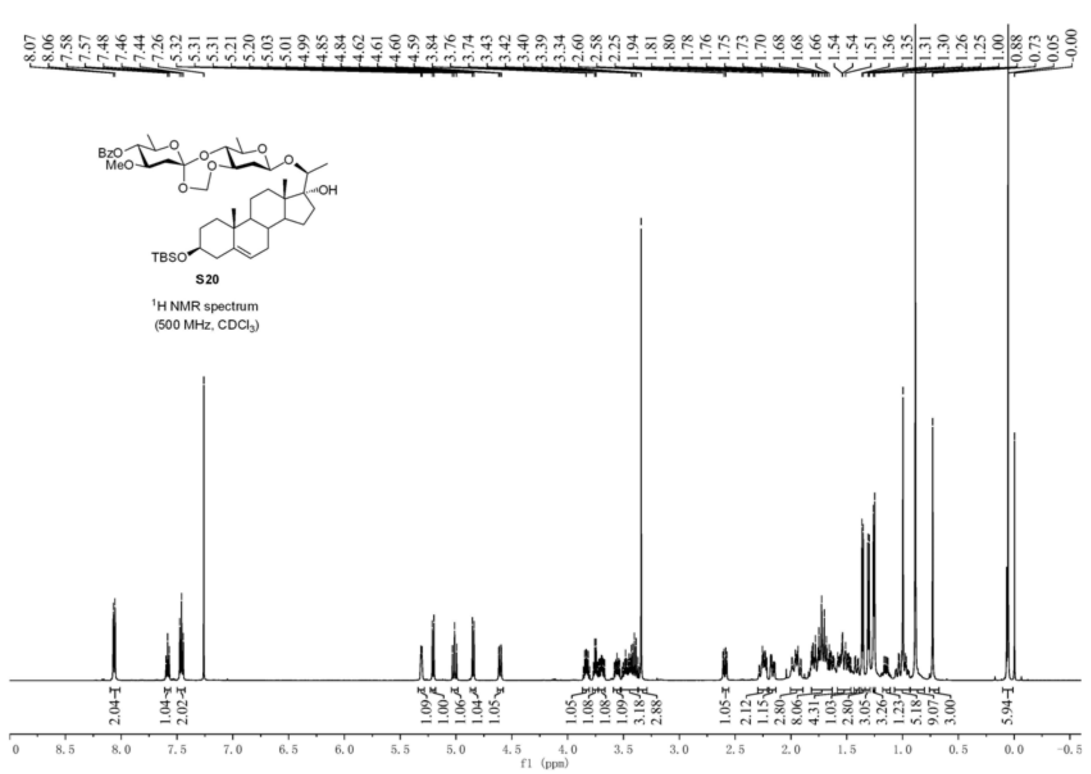

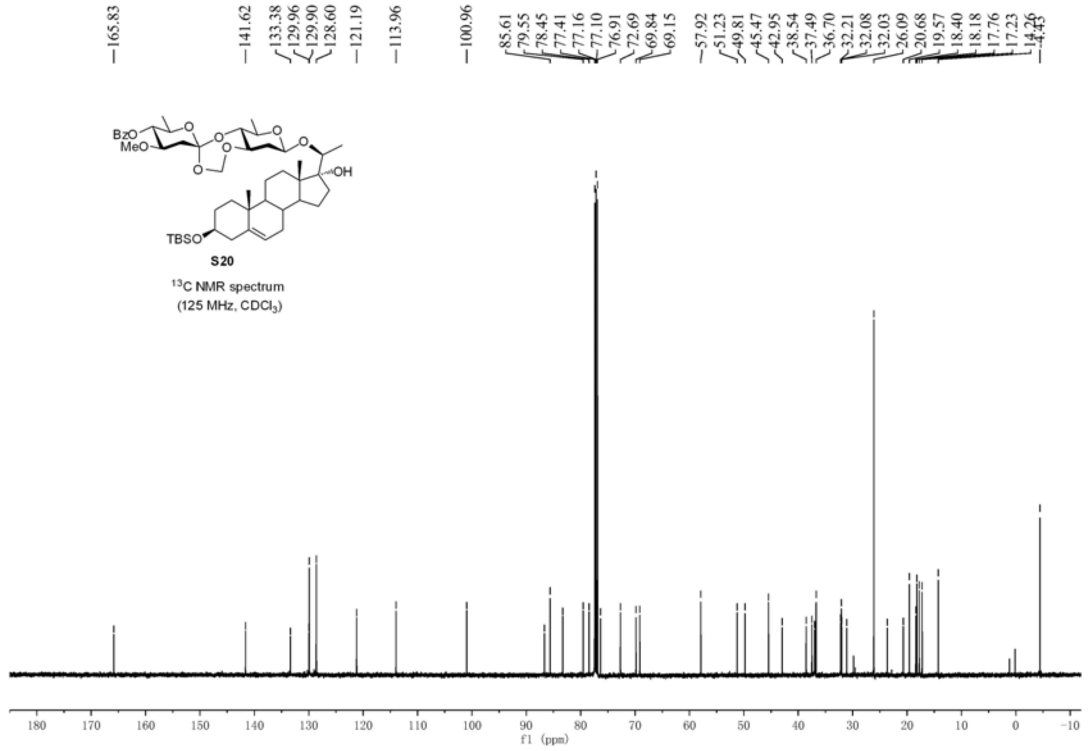

Supplementary Figure 51. ^{1}H and ^{13}C NMR spectra for compound S20.

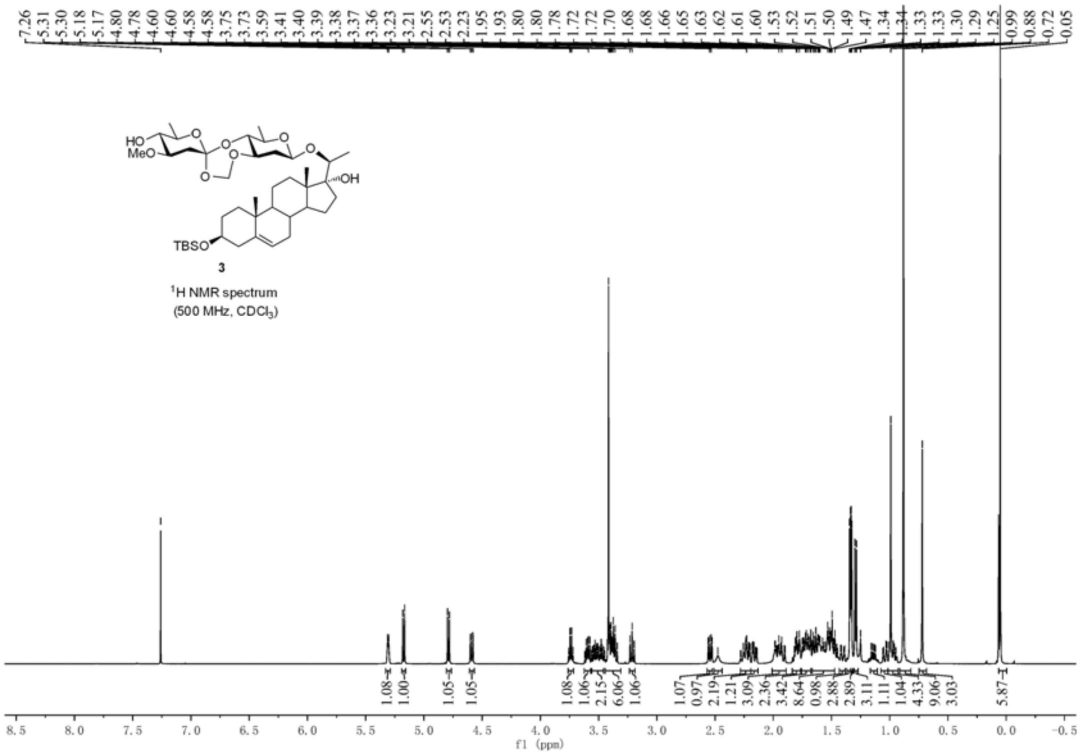

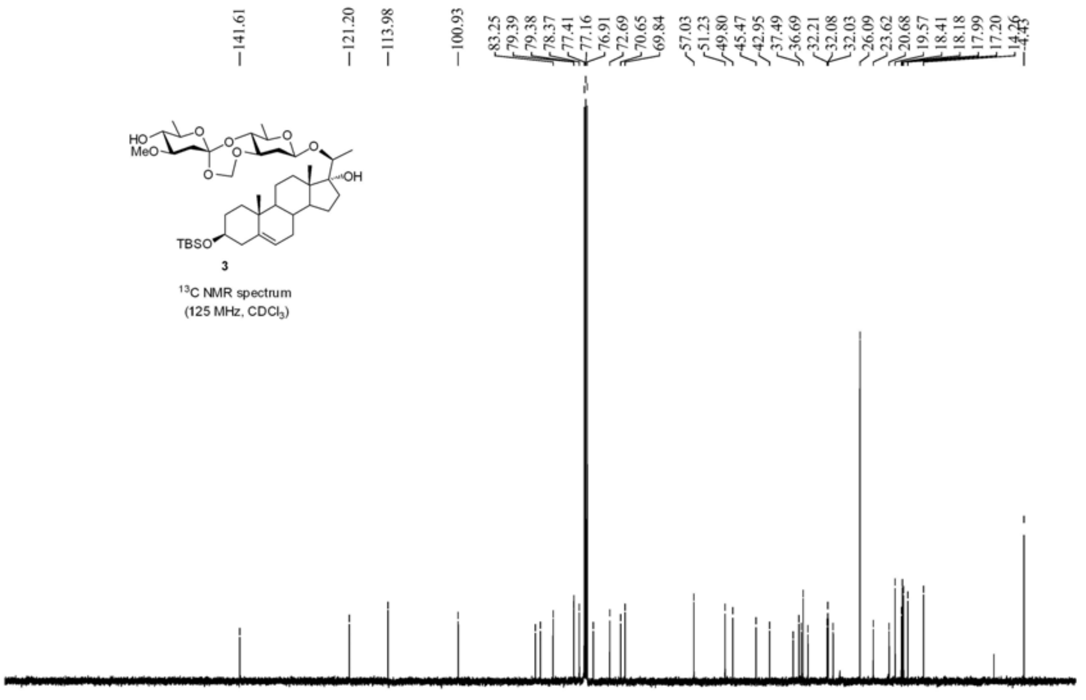

Supplementary Figure 52. ^{1}H and ^{13}C NMR spectra for compound 3.

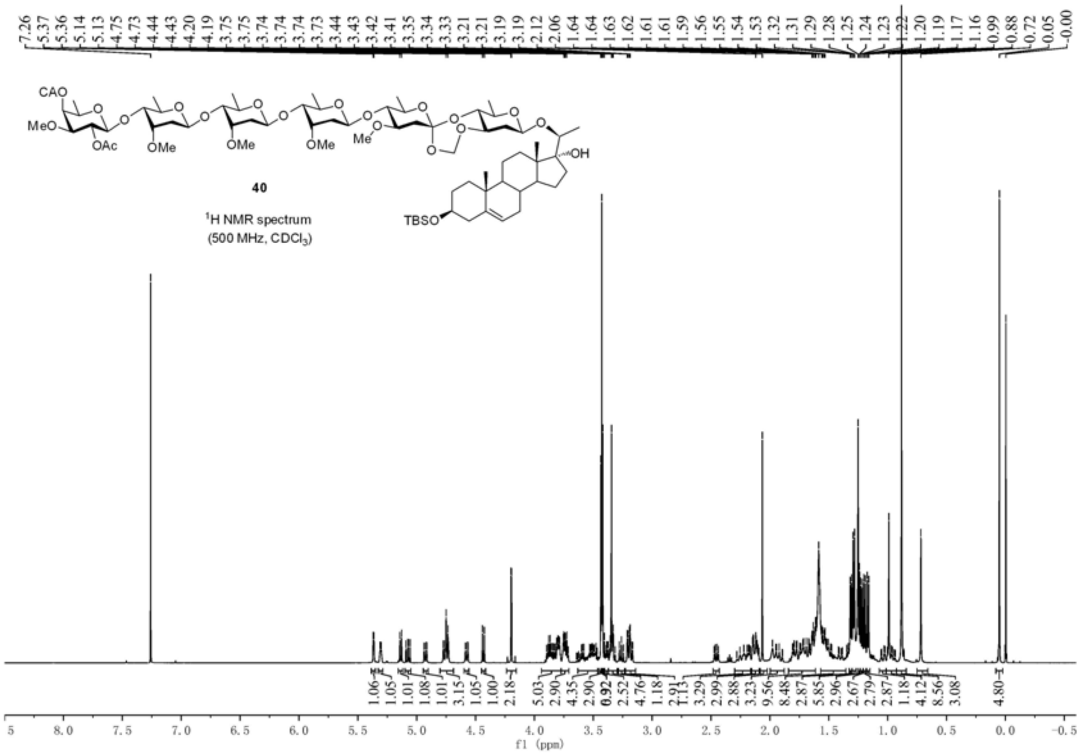

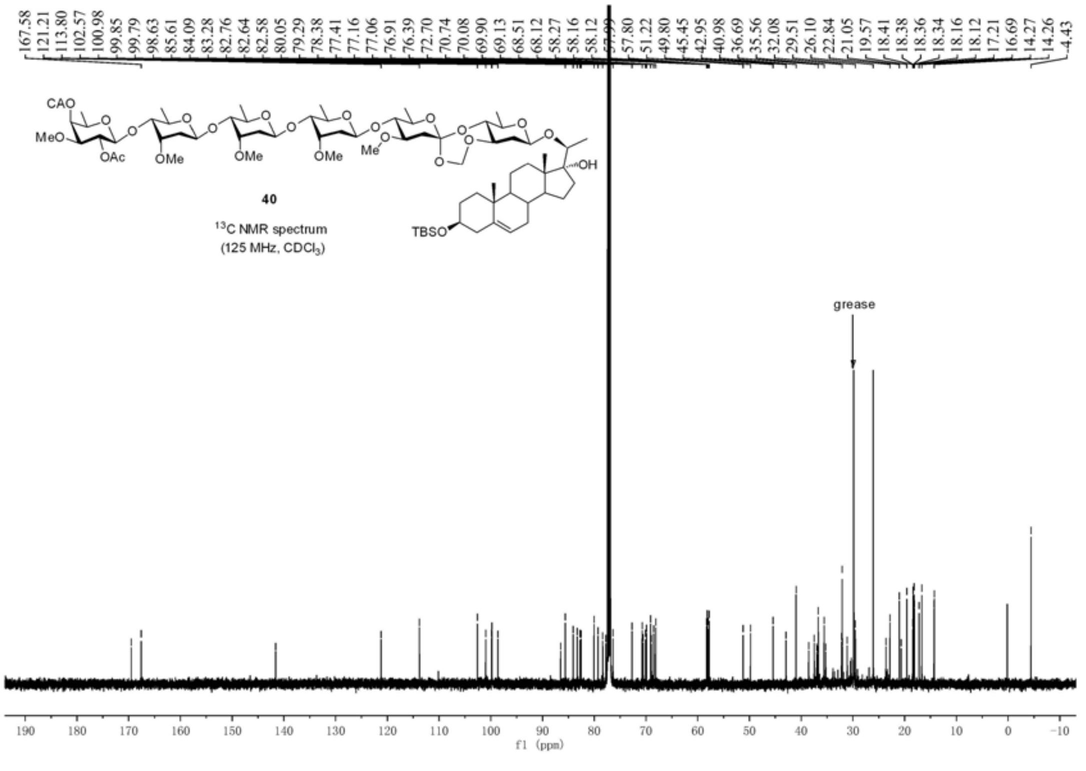

Supplementary Figure 53. ^{1}H and ^{13}C NMR spectra for compound 40.

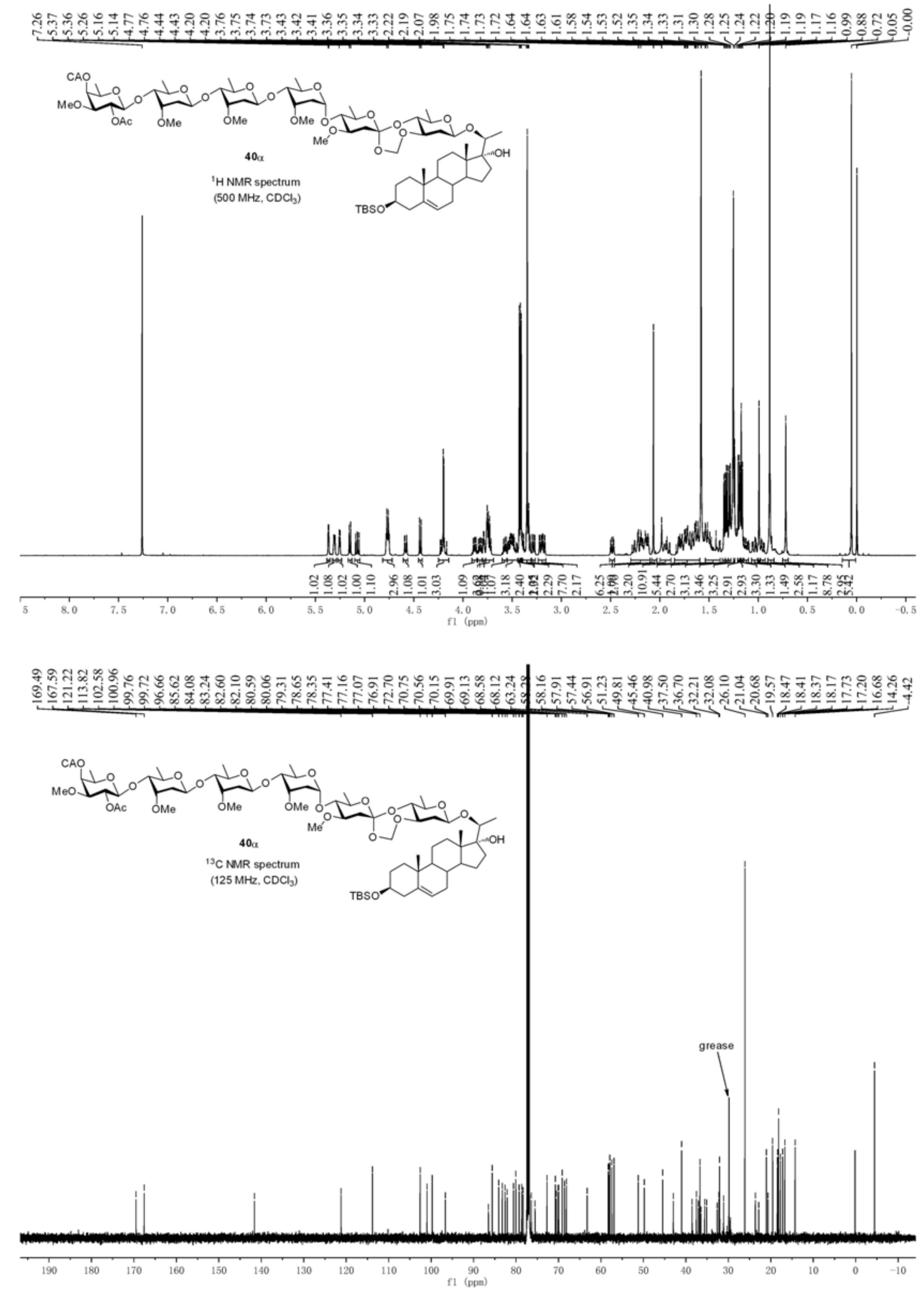

Supplementary Figure 54. ^{1}H and ^{13}C NMR spectra for compound 40α.

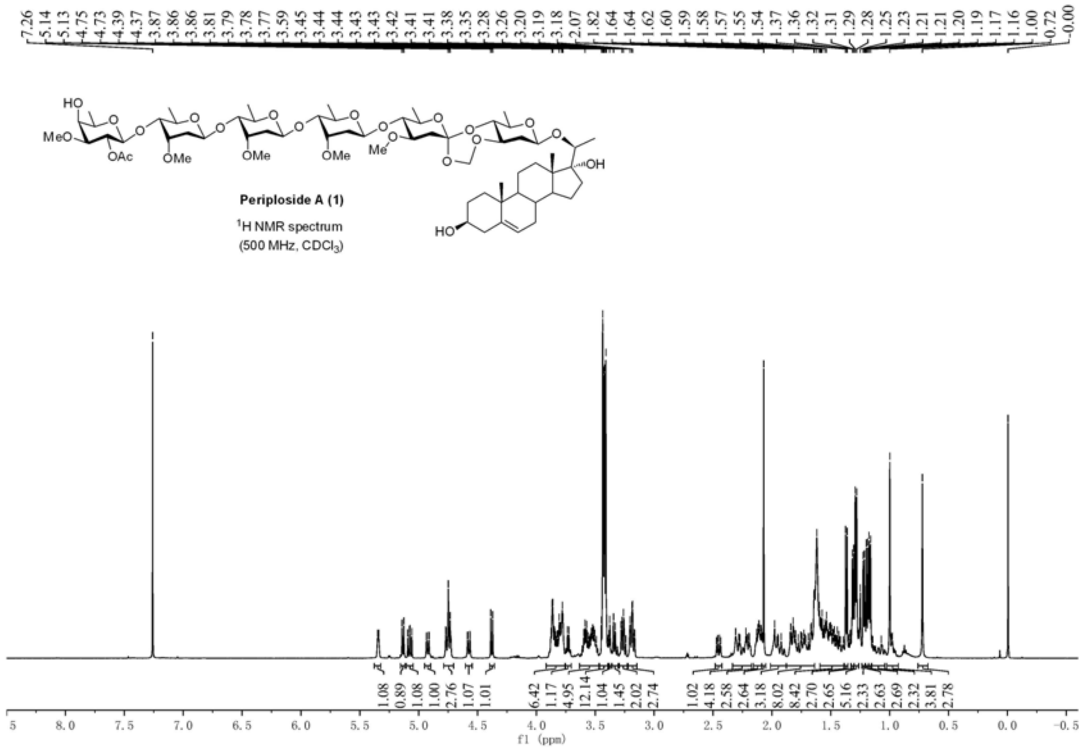

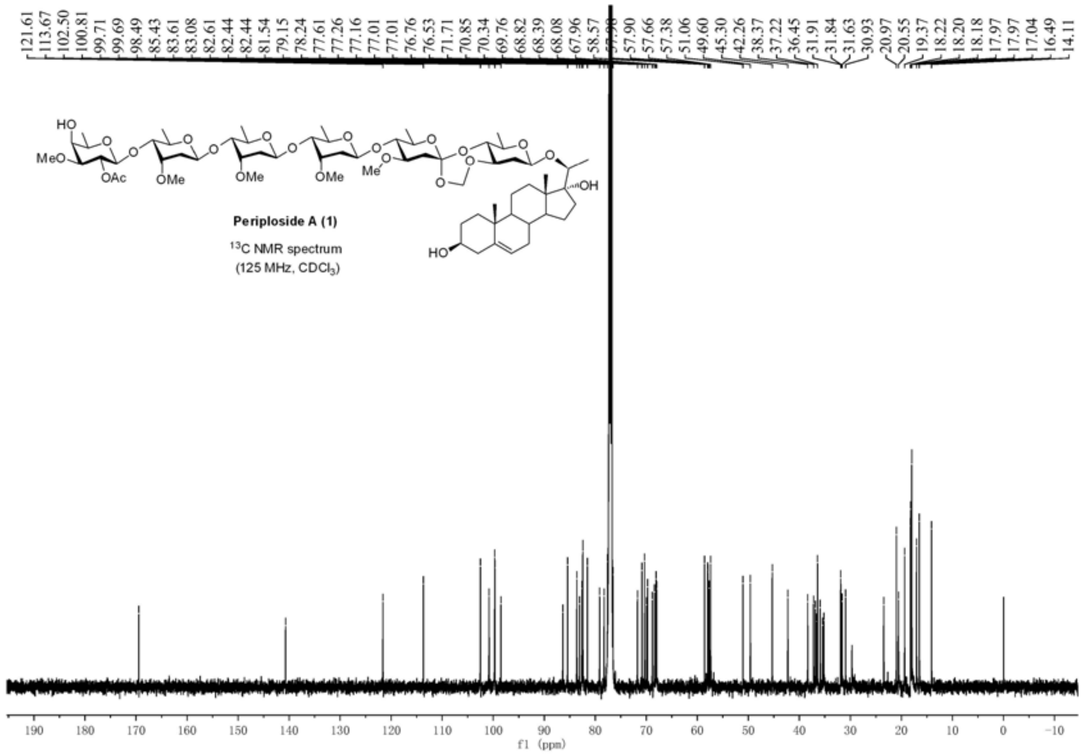

Supplementary Figure 55. ^{1}H and ^{13}C NMR spectra for compound 1.

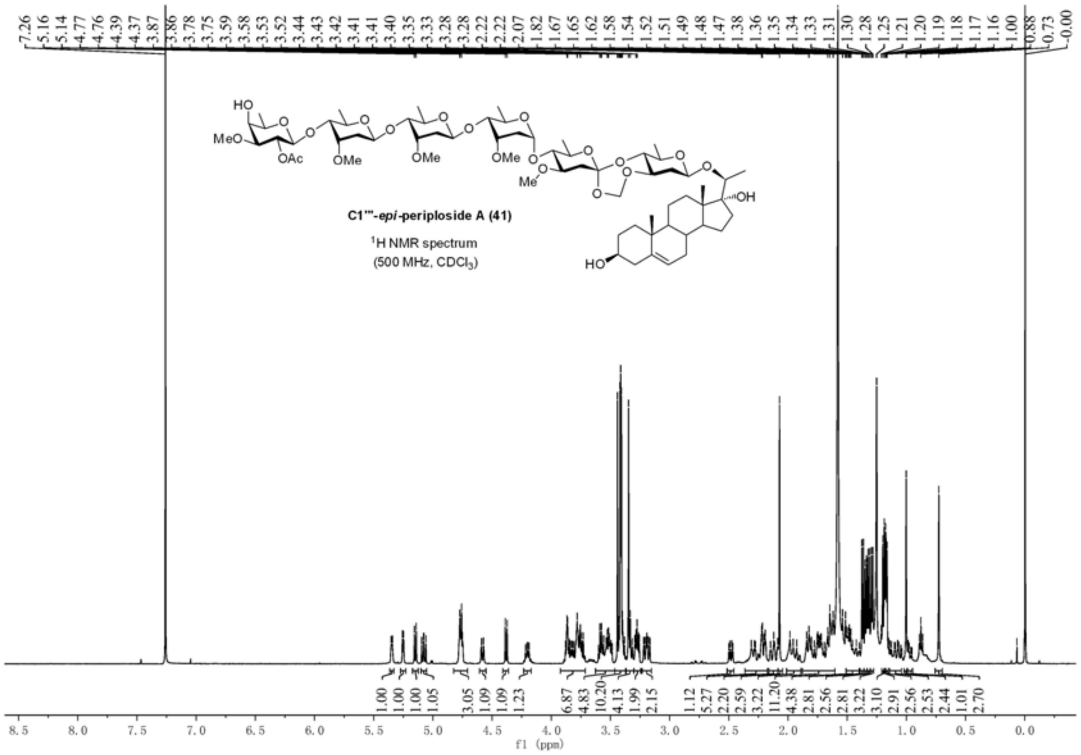

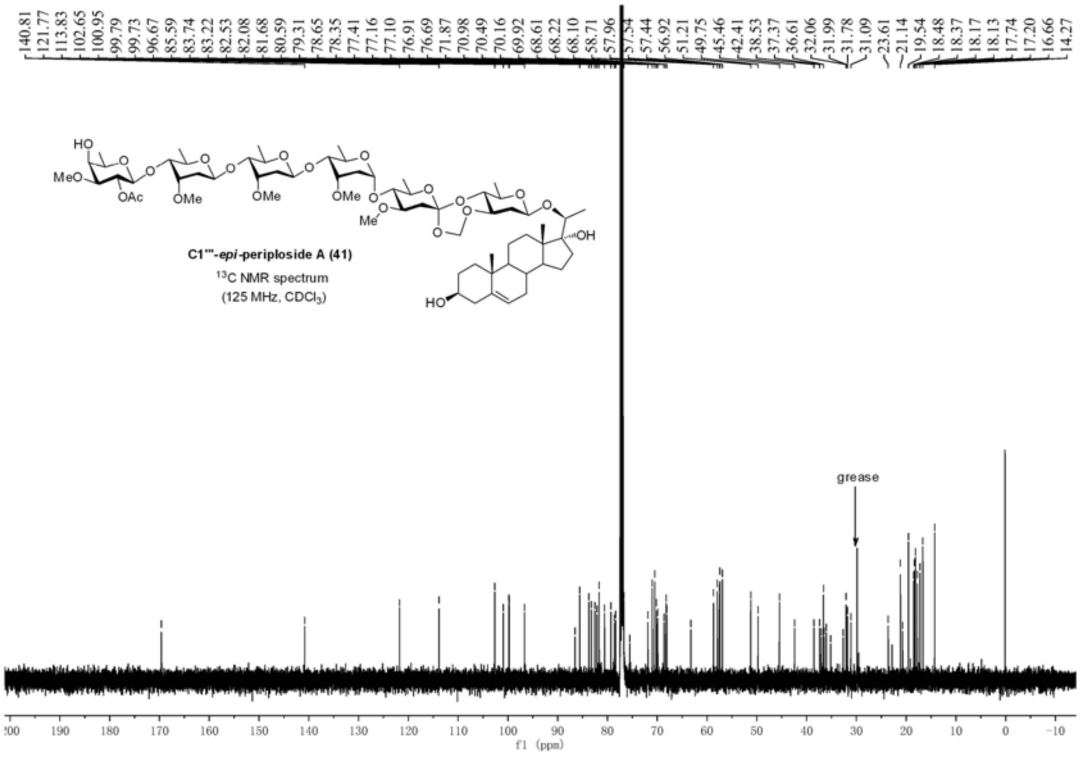

Supplementary Figure 56. ^{1}H and ^{13}C NMR spectra for compound 41.

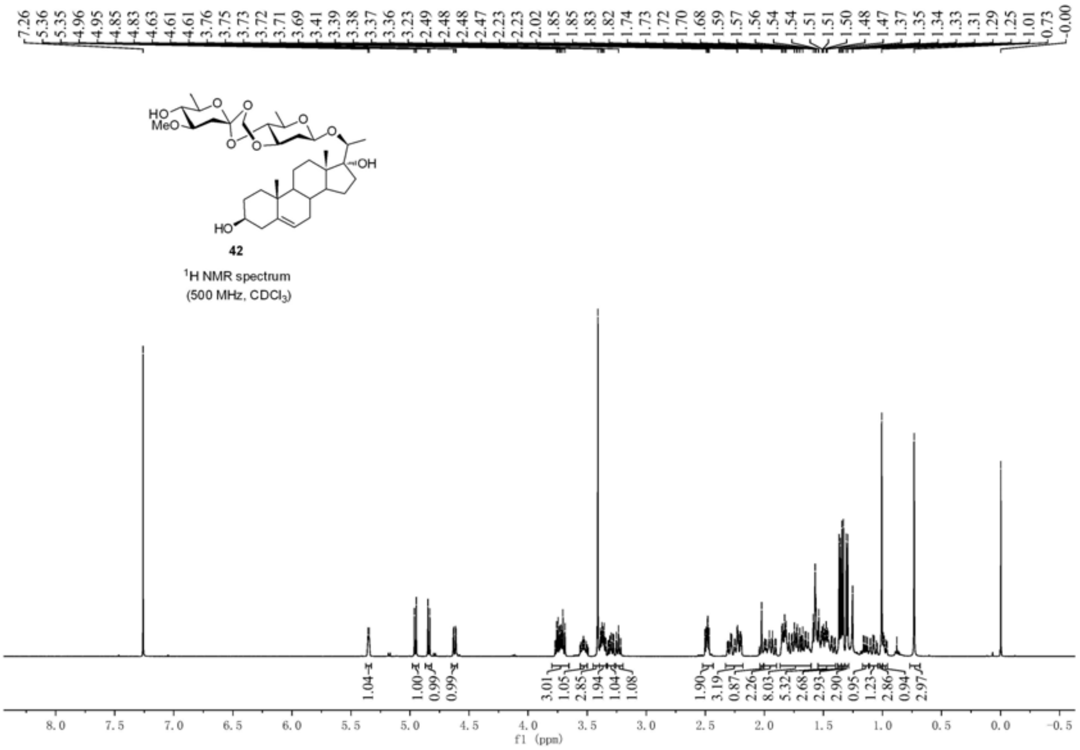

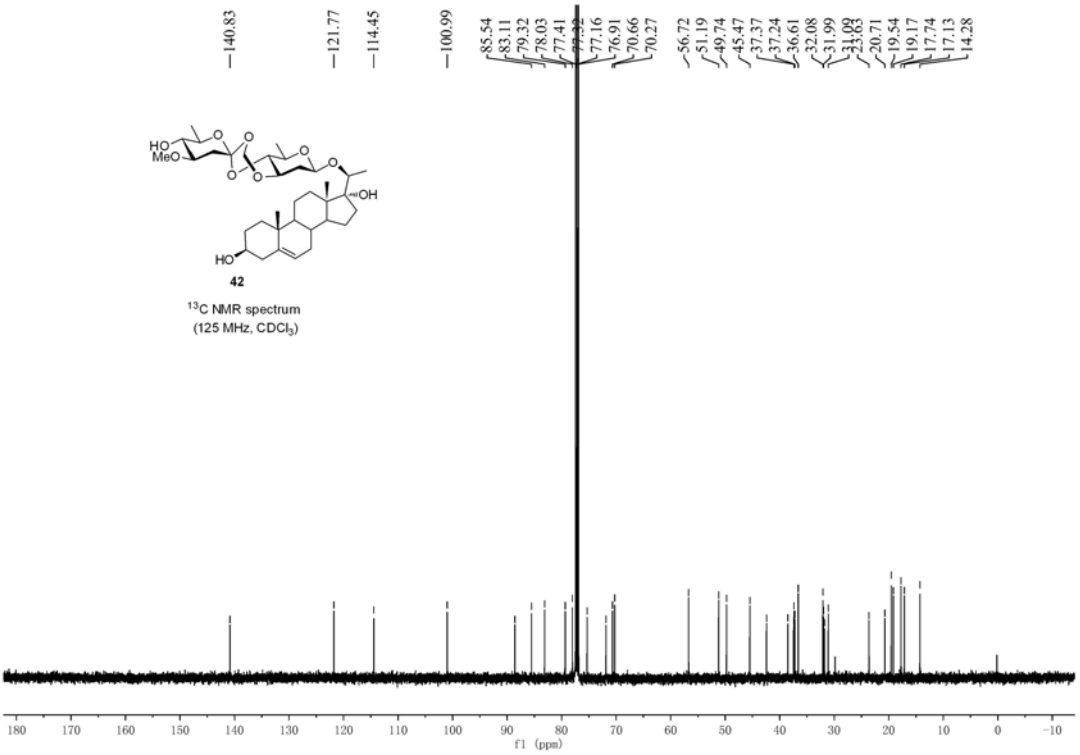

Supplementary Figure 57. ^{1}H and ^{13}C NMR spectra for compound 42.

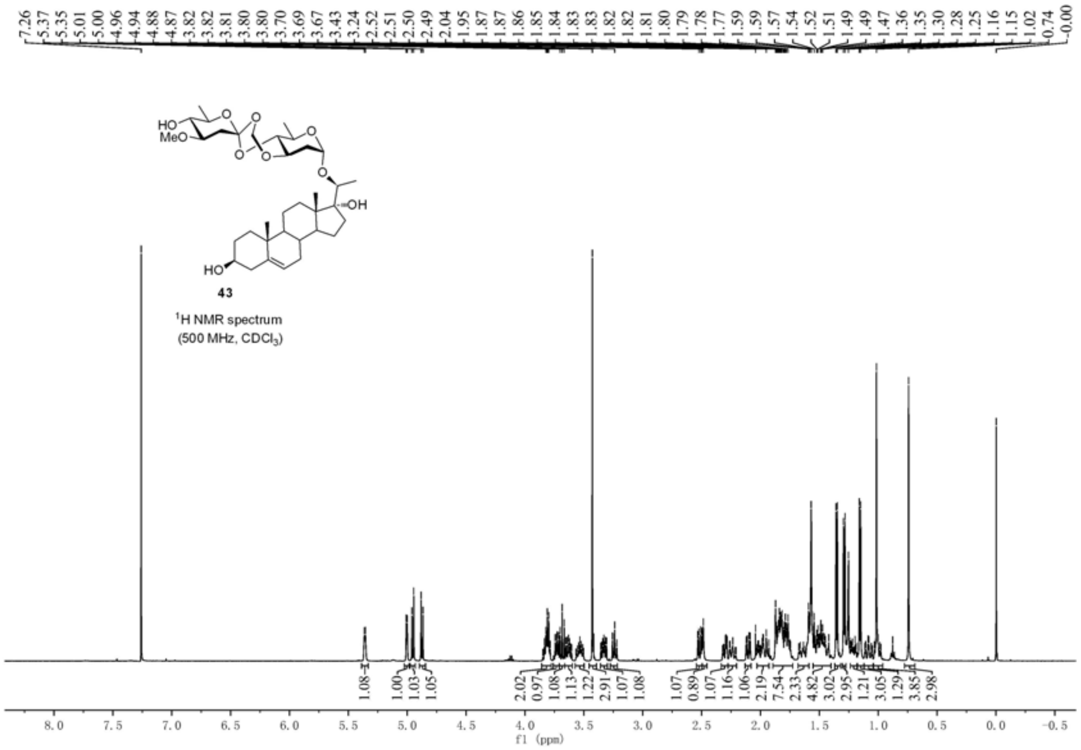

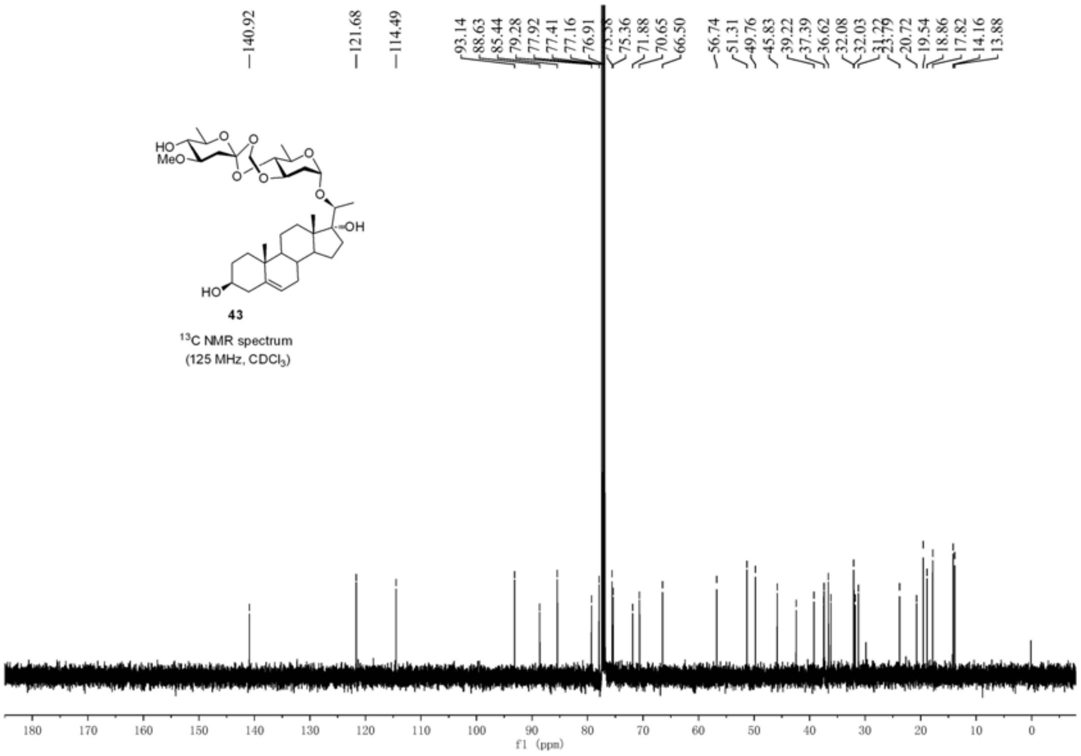

Supplementary Figure 58. ^{1}H and ^{13}C NMR spectra for compound 43.

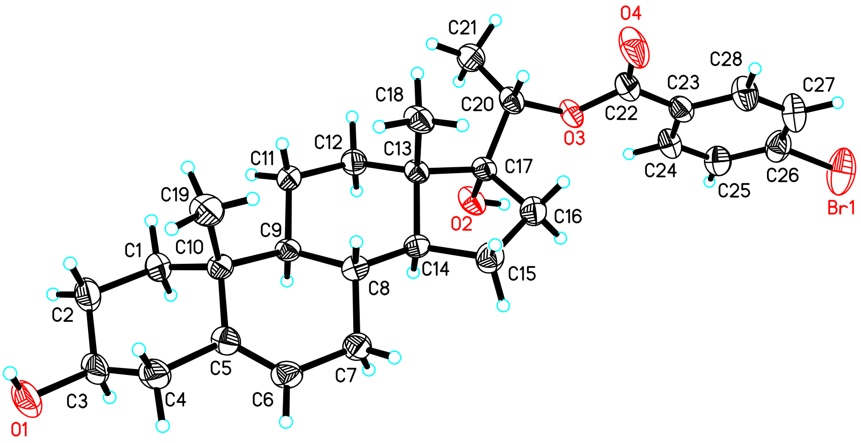

Supplementary Figure 59. X-ray structure of S14. CCDC 1014577 contains the supplementary crystallographic data for **S14** that is available free of charge from The Cambridge Crystallographic Data Centre *via* www.ccdc.cam.ac.uk/data_request/cif.

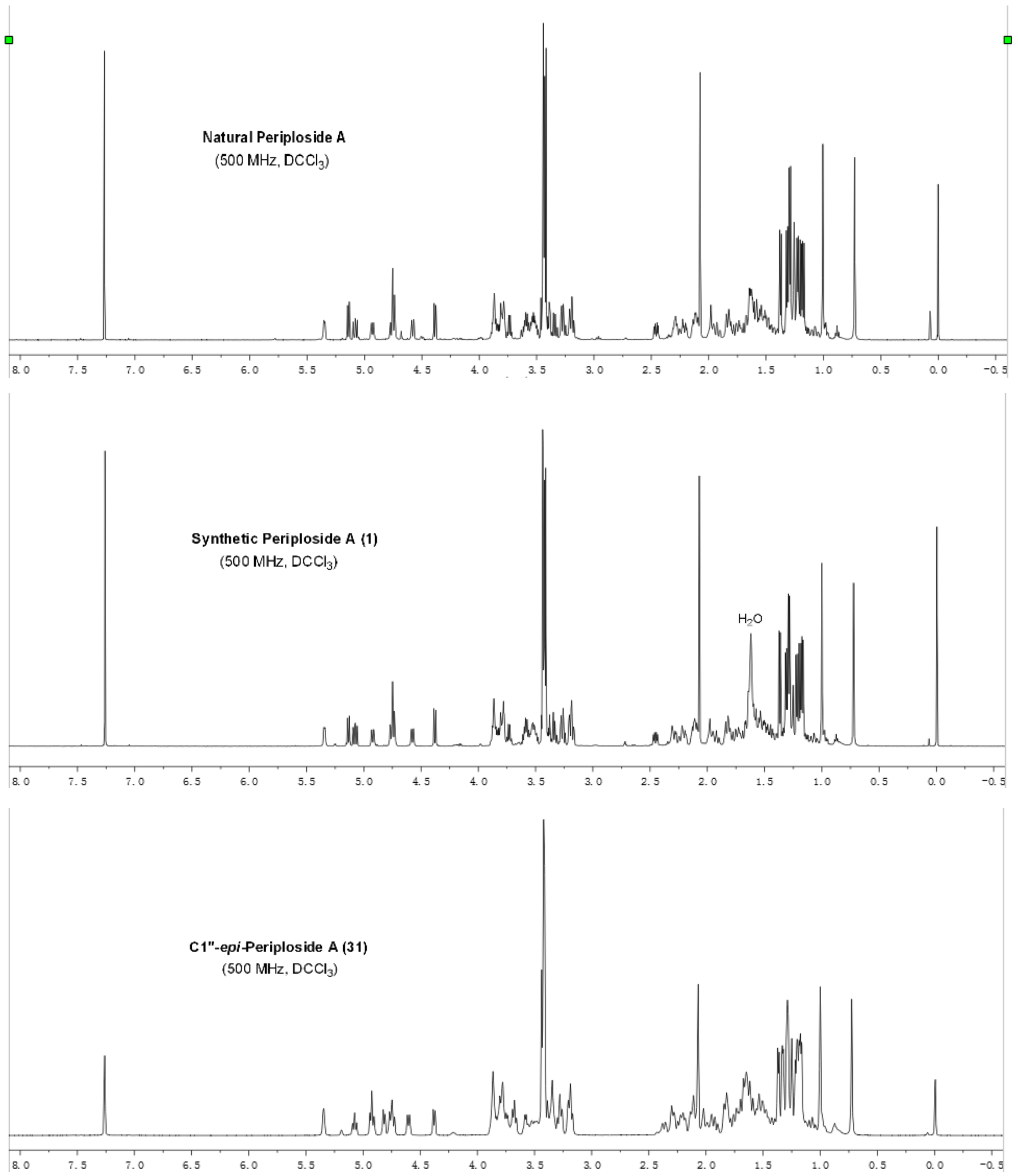

Supplementary Figure 60. Comparison of the ^{1}H NMR spectra of the synthetic 1, 31, and an authentic periploside A. (The authentic sample of periploside A was provided by Prof. Weimin Zhao from Shanghai Institute of Materia Medica, Chinese Academy of Sciences.)

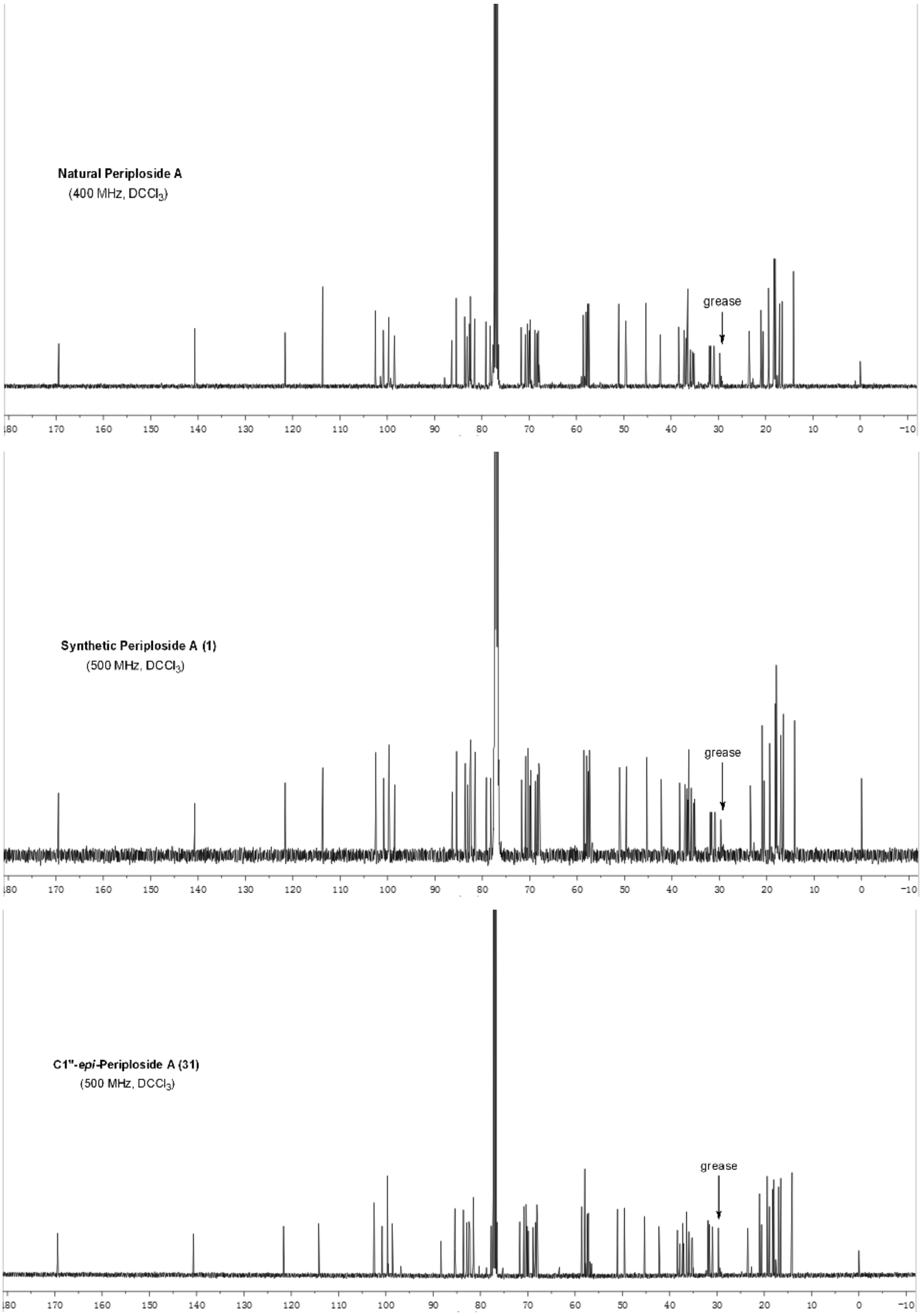

Supplementary Figure 61. Comparison of the ^{13}C NMR spectra of the synthetic 1, 31, and an authentic periploside A.

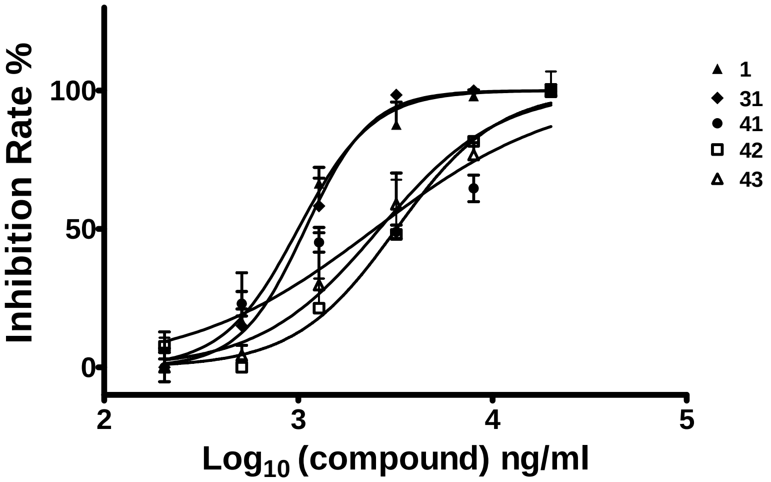

Supplementary Figure 62. Inhibition of the growth of T-lymphocytes by synthetic compounds 1, 31, and 41-43.

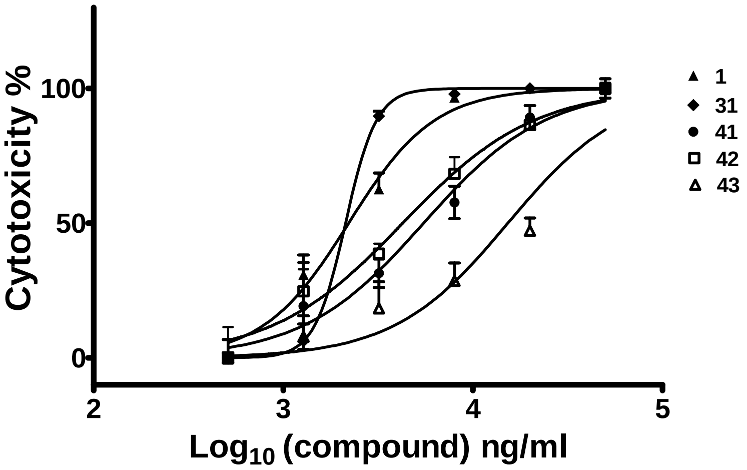

Supplementary Figure 63. Inhibition of concanavalin A (ConA)-induced T-lymphocyte proliferation by synthetic compounds 1, 31, and 41-43.

Supplementary Table 1. Attempts at synthesis of the acetal glycoside 24.

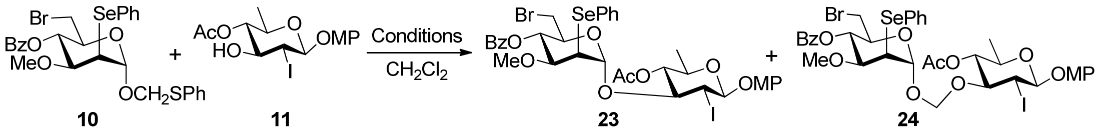

Entry	Promoter	Temp/°C	10/11[a]	Results: 23	24
1	NBS/TfOH	-50~0	1:1	80%	8%
2	Tf_2O,BSP,TTBP	-60~rt	1:1	70%	7%
3	MeOTf,DTBMP	0~rt	no reaction		
4	NIS/TfOH	-40~-30	1:1	65%	31%
5	NIS/TfOH	-40~-30	1:2	41%	41%
6	NIS/TfOH	0	1:2	complex	
7	NIS/AgOTf	0	1:2	complex	
8	DMTST,TTBP	0	1:1.8	90%	trace
9[b]	NIS/TfOH	-30	1:2 (inverse procedure)	20%	50%
10	NIS/TfOH	-40~-30	1:5	36%	63%
**11[b]**	**NIS/TfOH**	**-30**	**1:5 (inverse procedure)**	**25%**	**75%**

[a] The mole ratio of **10** and **11**. [b] Reaction was conducted by inverse procedure[1].

Supplementary Table 2. Attempts at the synthesis of the FABO disaccharide 37.

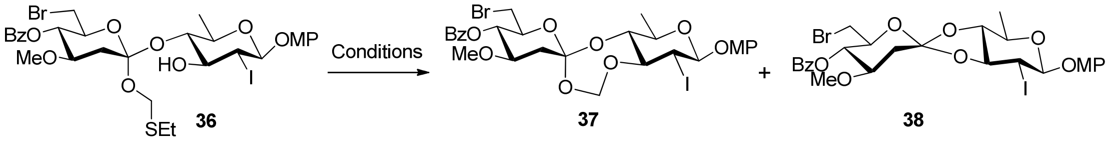

Entry	Promoter	Temp/°C	Solvent	Results: 38	37
1	NIS/TMSOTf	-40 ~ -30	CH_2Cl_2	90%	0
2	MeOTf/TTBP	-72 ~ rt	CH_2Cl_2	80%	0
3	DMTST/TTBP	-50 ~ rt	CH_2Cl_2	90%	0
4	$CuBr_2/Bu_4NBr$	0 ~ rt	CH_2Cl_2	90%	0
5[a]	MeOTf/DTBMP (frozen condition)	-20	p-xylene	80%	**10**
6	$BSP/Tf_2O/TTBP$	-60	CH_2Cl_2	95%	0
7	$BSP/Tf_2O/DTBP$	-78	CH_2Cl_2	80%	**10**
8[b]	$BSP/Tf_2O/DTBP$ (inverse procedure)	-78	CH_2Cl_2	no reaction	
9	$BSP/Tf_2O/DTBP$	-100	CH_2Cl_2/Et_2O	57%	**41%**
10[c]	$BSP/Tf_2O/DTBP$	-114	CH_2Cl_2/Et_2O	35%	**30%**
**11[c]**	**$BSP/Tf_2O/DTBP$**	**-114**	**Et_2O**	**18%**	**64%**

[a] Reaction was conducted at frozen condition[2]. [b] Reaction was conducted by inverse procedure[1]. [c] **38** was isolaterd as a single diastereoisomer.

Supplementary Table 3. Comparison of the ^{13}C NMR (500 MHz, $CDCl_3$) spectroscopic data of the synthetic 1 and C1"-*epi*-periploside A (31) with those reported for the natural periploside A[3].

Position	Natural periploside A	Synthetic **1**	C1''-*epi*-periploside A (**31**)	Position	Natural periploside A	Synthetic**1**	C1''-*epi*-periploside A (**31**)
aglycone				cym.			
C-1	37.3	37.2	37.2	C-1	98.5	98.5	98.7
C-2	31.7	31.6	31.6	C-2	36.0	35.9	35.9
C-3	71.7	71.7	71.7	C-3	77.7	77.6	77.7
C-4	42.3	42.3	42.3	C-4	82.5	82.4	82.4
C-5	140.7	140.7	140.7	C-5	68.9	68.8	68.9
C-6	121.6	121.6	121.6	C-6	18.3	18.2	18.2
C-7	31.9	31.8	31.8	OMe	58.0	58.0	57.9
C-8	32.0	31.9	31.9	cym.			
C-9	49.7	49.6	49.6	C-1	99.7	99.7	99.7
C-10	36.5	36.5	36.5	C-2	35.5	35.4	35.3
C-11	20.6	20.6	20.6	C-3	76.6	76.5	76.5
C-12	31.0	30.9	30.9	C-4	82.5	82.4	82.2
C-13	45.3	45.3	45.3	C-5	68.4	68.4	68.4
C-14	51.1	51.1	51.0	C-6	18.2	18.2	18.2
C-15	23.5	23.5	23.5	OMe	58.0	57.9	57.9
C-16	36.9	36.9	37.1	cym.			
C-17	85.4	85.4	85.4	C-1	99.8	99.7	99.7
C-18	14.1	14.1	14.1	C-2	35.3	35.2	35.2
C-19	19.4	19.4	19.4	C-3	77.1	77.2	77.2
C-20	83.0	83.1	82.9	C-4	83.7	83.6	83.6
C-21	18.0	18.0	17.9	C-5	68.1	68.1	68.1
can.				C-6	18.2	18.0	18.0
C-1	100.8	100.8	100.8	OMe	58.6	58.6	58.6
C-2	38.4	38.4	38.4	dig.			
C-3	77.1	77.0	76.9	C-1	102.5	102.5	102.5
C-4	79.2	79.1	81.5	C-2	70.9	70.9	70.8
C-5	70.0	69.9	70.1	C-3	81.6	81.5	81.5
C-6	17.1	17.0	17.0	C-4	68.0	68.0	67.9
OCH_2O	86.4	86.4	88.4	C-5	70.4	70.3	70.3
ole.				C-6	16.5	16.5	16.5
C-1	113.7	113.7	114.2	OMe	57.4	57.4	57.4
C-2	36.7	36.7	37.9	OAc	169.4	169.4	169.4
C-3	78.3	78.2	77.4		21.0	21.0	21.0
C-4	82.7	82.6	82.4				
C-5	69.8	69.8	69.9				
C-6	18.3	18.2	18.9				
OMe	57.7	57.7	57.2				

Supplementary Methods

General Remarks. All reactions were carried out under nitrogen or argon with anhydrous solvents in flame-dried glassware, unless otherwise noted. All the glycosylation reactions were performed in the presence of 4Å or 5Å molecular sieves, which were flame-dried immediately before use in the reaction under high vacuum. Glycosylation solvents were dried using a solvent purification system and used directly without further drying. The chemicals used were reagent grade as supplied, except where noted. Analytical thin-layer chromatography was performed using silica gel 60 F254 glass plates. Compound spots were visualized by UV light (254 nm) or by heating with a solution with 10% H_2SO_4 in ethanol. Flash column chromatography was performed on silica gel. NMR spectra were recorded on Bruker AV-400 or Agilent 500 instrument and referenced using Me_4Si (0 ppm), residual $CHCl_3$ (^{1}H NMR δ = 7.26 ppm, ^{13}C NMR δ = 77.16 ppm). Splitting patterns are indicated as s (singlet), d (doublet), t (triplet), q (quartet), and brs (broad singlet) for ^{1}H NMR data. ESI-MS and MALDI-MS were run on an IonSpec Ultra instrument using HP5989A or VG Quattro MS. Optical rotations were measured using a Perkin-Elmer 241 polarimeter.

Synthesis of digitalosyl *ortho*-cyclopropylethynylbenzoate 4.

p-Methoxyphenyl 2-*O*-benzyl-3,4-*O*-isopropylidene-α-D-fucopyranoside (S2)

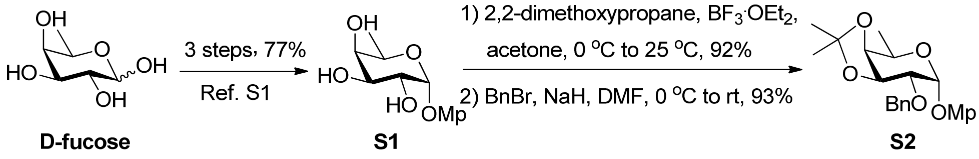

To a solution of *p*-methoxyphenyl α-D-fucopyranoside (**S1**)[4] (3.74 g, 13.8 mmol) in acetone (100 mL) was added 2,2-dimethoxypropane (2.90 mL, 23.5 mmol). The mixture was cooled to 0 °C, then $BF_3{\cdot}OEt_2$ (0.26 mL, 2.08 mmol) was added dropwise. The mixture was stirred for 12 h at rt before being quenched by addition of Et_3N (2 mL). The mixture was concentrated in vacuo. The residue was purified by flash chromatography (petroleum ether/EtOAc = 4:1) to afford a colorless syrup (3.95

g, 92%).

The above syrup (3.95 g, 12.7 mmol) was dissolved in DMF (50 mL) and cooled to 0 °C. Benzyl bromide (3.28 mL, 27.7 mmol) and sodium hydride (60% in oil) (1.1 g, 27.7 mmol) were added carefully. The mixture was stirred for 3 h while warming to rt. The solution was quenched with water and diluted with CH_2Cl_2. The water phase was extracted with CH_2Cl_2 for three times. The combined organic layer was washed with brine, dried over Na_2SO_4, and concentrated. The residue was purified by flash chromatography (petroleum ether/EtOAc = 20:1) to afford **S2** (4.7 g, 93%) as a white solid: $[\alpha]^{28}_D$ = +156.2 (*c* = 1.7, $CHCl_3$); ^1H NMR (400 MHz, $CDCl_3$) δ 7.37-7.24 (m, 5H), 7.02 (d, *J* = 9.0 Hz, 2H), 6.82 (d, *J* = 9.0 Hz, 2H), 5.33 (d, *J* = 3.3 Hz, 1H), 4.82-4.73 (m, 2H), 4.49 (dd, *J* = 7.6, 5.7 Hz, 1H), 4.24-4.21 (m, 1H), 4.10 (dd, *J* = 5.3, 2.3 Hz, 1H), 3.76 (s, 3H), 3.64 (dd, *J* = 7.8, 3.4 Hz, 1H), 1.43 (s, 3H), 1.37 (s, 3H), 1.29 (d, *J* = 6.7 Hz, 3H); ^{13}C NMR (100 MHz, $CDCl_3$) δ 155.0, 151.1, 138.3, 128.5, 128.0, 127.8, 117.8, 114.7, 109.0, 96.5, 76.2, 76.2, 76.0, 72.4, 64.0, 55.7, 28.3, 26.5, 16.4; HRMS (ESI) calcd for $C_{23}H_{28}O_6Na$ $[M+Na]^+$ 423.1778, found 423.1785.

***p*-Methoxyphenyl 2-*O*-benzyl-α-D-fucopyranoside (S3)**

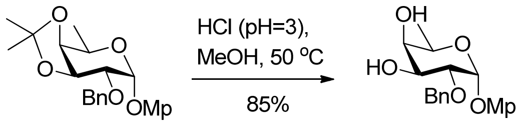

S2 **S3**

To a solution of **S2** (3.94 g, 9.84 mmol) in methanol (50 mL) and water (5 mL) was added an aqueous solution of 5% HCl (a few drops) until pH = 3. The mixture was heated to 50 °C and stirred for 10 h until the TLC showed complete conversion. The mixture was quenched with a saturated $NaHCO_3$ solution. The solvent was then removed. The residue was diluted with CH_2Cl_2, washed with saturated $NaHCO_3$ solution and brine, respectively, and was then dried over Na_2SO_4 and concentrated. The residue was purified by flash chromatography (petroleum ether/EtOAc = 2:1) to afford **S3** (3.0 g, 85%) as a white solid: $[\alpha]^{29}_D$ = +166.4 (*c* = 1.4, $CHCl_3$); ^1H NMR (400 MHz, $CDCl_3$) δ 7.33-7.25 (m, 5H), 7.00 (d, *J* = 9.1 Hz, 2H), 6.82 (d, *J* = 9.1 Hz, 2H), 5.39 (d, *J* = 3.5 Hz, 1H), 4.68-4.61 (m, 2H), 4.19 (dt, *J* = 9.8, 3.1 Hz, 1H), 4.09 (q, *J* = 6.5 Hz, 1H), 3.86-3.83 (m, 2H), 3.77 (s, 3H), 2.94 (d, *J* = 3.1 Hz, 1H), 2.72 (d,

J = 1.8 Hz, 1H), 1.25 (d, J = 6.6 Hz, 3H); ^{13}C NMR (100 MHz, $CDCl_3$) δ 155.1, 151.3, 137.8, 128.7, 128.2, 118.1, 114.7, 96.3, 76.3, 72.6, 71.6, 69.5, 66.4, 55.8, 16.2; HRMS (ESI) calcd for $C_{20}H_{24}O_6Na$ $[M+Na]^+$ 383.1465, found 383.1477.

***p*-Methoxyphenyl 2-*O*-benzyl-3-*O*-methyl-α-D-fucopyranoside (S4)**

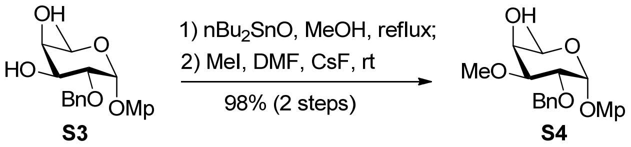

A solution of **S3** (2.25 g, 6.25 mmol) in methanol (20 mL) was refluxed in the presence of dibutyltin oxide (1.87 mg, 7.50 mmol) for 4 h. The solvent was then removed in vacuo, and the residue was dried azeotropically with toluene and dissolved in DMF (20 mL). Methyl iodide (1.9 mL, 31.3 mmol) and CsF (1.4 g, 9.37 mmol) were added, and the mixture was stirred at rt for 12 h. The foamy solution was diluted with 10% HCl and extracted with EtOAc. The combined organic layer was washed with brine, dried over Na_2SO_4, and concentrated. The residue was purified by flash chromatography (petroleum ether/EtOAc = 3:1) to afford **S4** (2.3 g, 98%) as a syrup: $[\alpha]^{28}_{D}$ = +137.2 (c = 1.3, $CHCl_3$); ^{1}H NMR (400 MHz, $CDCl_3$) δ 7.34-7.22 (m, 5H), 7.01 (d, J = 9.1 Hz, 2H), 6.81 (d, J = 9.1 Hz, 2H), 5.31 (d, J = 3.5 Hz, 1H), 4.78 (d, J = 12.1 Hz, 1H), 4.64 (d, J = 12.1 Hz, 1H), 4.07 (q, J = 6.4 Hz, 1H), 3.93 (d, J = 2.3 Hz, 1H), 3.87 (dd, J = 9.8, 3.6 Hz, 1H), 3.80 (dd, J = 9.8, 3.2 Hz, 1H), 3.74 (s, 3H), 3.57 (s, 3H), 2.65 (s, 1H), 1.26 (d, J = 6.6 Hz, 3H); ^{13}C NMR (100 MHz, $CDCl_3$) δ 154.9, 151.2, 138.4, 128.4, 127.8, 127.7, 118.1, 114.5, 97.0, 79.5, 75.2, 73.1, 69.1, 66.1, 58.0, 55.6, 16.2; HRMS (ESI) calcd for $C_{21}H_{26}O_6Na$ $[M+Na]^+$ 397.1622, found 397.1618.

***p*-Methoxyphenyl 2-*O*-benzyl-4-*O*-chloroacetyl-3-*O*-methyl-α-D-fucopyranoside (S5)**

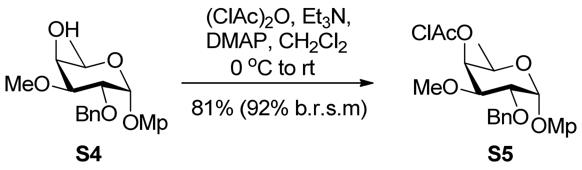

To a solution of **S4** (2.20 g, 5.89 mmol) in CH_2Cl_2 (20 mL) were added Et_3N (2.46

mL, 17.7 mmol) and DMAP (72 mg, 0.59 mmol). The mixture was cooled to 0 °C, $(ClAc)_2O$ (1.51 g, 8.83 mmol) was added under stirring. After stirring for 2 h at rt, the reaction was quenched with MeOH. The solution was diluted with CH_2Cl_2, washed with saturated $NaHCO_3$ solution and brine, respectively, and was then dried over Na_2SO_4 and concentrated. The residue was purified by flash chromatography (petroleum ether/EtOAc = 6:1 to 2:1) to afford **S5** (2.16 g, 92% based on 81% conversion) as a colorless syrup and recovered **S4** (260 mg, 11%). **S5**: $[\alpha]^{28}_{D}$ = +104.7 (c = 1.8, $CHCl_3$); ^1H NMR (400 MHz, $CDCl_3$) δ 7.26-7.14 (m, 5H), 6.91 (d, J = 9.0 Hz, 2H), 6.74 (d, J = 9.0 Hz, 2H), 5.36 (d, J = 2.8 Hz, 1H), 5.24 (d, J = 3.5 Hz, 1H), 4.74 (d, J = 12.1 Hz, 1H), 4.56 (d, J = 12.1 Hz, 1H), 4.15-4.09 (m, 3H), 3.84 (dd, J = 10.0, 3.2 Hz, 1H), 3.71 (dd, J = 10.1, 3.6 Hz, 1H), 3.67 (s, 3H), 3.40 (s, 3H), 1.04 (d, J = 6.5 Hz, 3H); ^{13}C NMR (100 MHz, $CDCl_3$) δ 167.3, 155.1, 151.1, 138.4, 128.4, 127.9, 127.8, 118.2, 114.6, 97.4, 77.9, 75.0, 73.4, 72.4, 65.2, 58.1, 55.7, 40.8, 16.2; HRMS (ESI) calcd for $C_{23}H_{27}O_7ClNa$ $[M+Na]^+$ 473.1338, found 473.1329.

***p*-Methoxyphenyl 4-*O*-chloroacetyl-3-*O*-methyl-α-D-fucopyranoside (S6)**

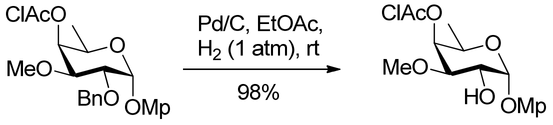

S5 **S6**

To a solution of **S5** (2.06 g, 4.57 mmol) in EtOAc (15 mL) was added palladium on carbon (10% Pd/C, 206 mg). The suspension was stirred under hydrogen pressure (1 atm) for 5 h and then filtered. The filtrate was concentrated. The residue was purified by flash chromatography (petroleum ether/EtOAc = 2:1) to afford **S6** (1.62 g, 98%) as a white solid: $[\alpha]^{28}_{D}$ = +171.1 (c = 1.2, $CHCl_3$); ^1H NMR (400 MHz, $CDCl_3$) δ 7.01 (d, J = 9.1 Hz, 2H), 6.83 (d, J = 9.1 Hz, 2H), 5.46 (d, J = 3.8 Hz, 1H), 5.44 (d, J = 2.8 Hz, 1H), 4.23 (dd, J = 13.1, 6.6 Hz, 1H), 4.18 (s, 2H), 3.97 (ddd, J = 10.4, 7.0, 3.8 Hz, 1H), 3.76 (s, 3H), 3.71 (dd, J = 10.0, 3.2 Hz, 1H), 3.47 (s, 3H), 2.45 (d, J = 7.2 Hz, 1H), 1.16 (d, J = 6.6 Hz, 3H); ^{13}C NMR (100 MHz, $CDCl_3$) δ 167.4, 155.4, 150.8, 118.2, 114.8, 98.5, 78.7, 71.5, 68.1, 65.7, 57.9, 55.8, 40.8, 16.3; HRMS (ESI) calcd for $C_{16}H_{21}O_7ClNa$ $[M+Na]^+$ 383.0868, found 383.0880.

p-Methoxyphenyl 2-O-acetyl-4-O-chloroacetyl-3-O-methyl-α-D-fucopyranoside (S7)

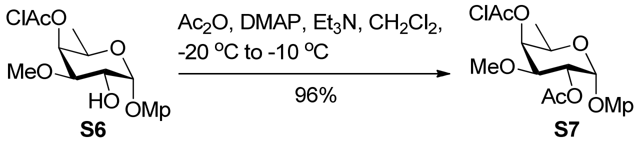

To a solution of **S6** (530 mg, 1.47 mmol) in CH_2Cl_2 (10 mL) were added Et_3N (0.61 mL, 4.41 mmol) and DMAP (18 mg, 0.15 mmol). The mixture was cooled to –20 °C, then Ac_2O (0.17 mL, 1.76 mmol) was added under stirring. The mixture was stirred for 1 h while warming to –10 °C. The reaction was quenched with MeOH. The solution was diluted with CH_2Cl_2, washed with saturated $NaHCO_3$ solution and brine, respectively. The organic phase was dried over Na_2SO_4 and concentrated. The residue was purified by flash chromatography (petroleum ether/EtOAc = 6:1) to afford **S7** (570 mg, 96%) as a white solid: $[\alpha]^{25}_{D}$ = +178.2 (c = 1.1, $CHCl_3$); ^{1}H NMR (400 MHz, $CDCl_3$) δ 6.97 (d, J = 9.1 Hz, 2H), 6.82 (d, J = 9.1 Hz, 2H), 5.55 (d, J = 3.7 Hz, 1H), 5.48 (d, J = 2.8 Hz, 1H), 5.09 (dd, J = 10.5, 3.7 Hz, 1H), 4.25 (dd, J = 13.1, 6.5 Hz, 1H), 4.20 (s, 2H), 3.93 (dd, J = 10.5, 3.3 Hz, 1H), 3.76 (s, 3H), 3.44 (s, 3H), 2.10 (s, 3H), 1.17 (d, J = 6.5 Hz, 3H); ^{13}C NMR (100 MHz, $CDCl_3$) δ 170.6, 167.4, 155.4, 150.9, 118.1, 114.8, 96.2, 75.6, 71.9, 69.7, 65.3, 58.1, 55.8, 40.8, 21.0, 16.2; HRMS (ESI) calcd for $C_{18}H_{23}O_8ClNa$ $[M+Na]^+$ 425.0974, found 425.0972.

Digitalosyl *ortho*-cyclopropylethynylbenzoate 4

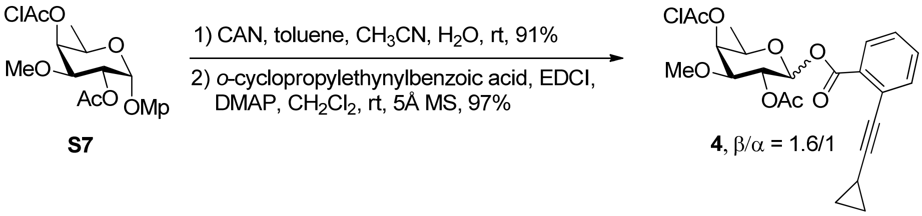

To a solution of **S7** (1.20 g, 2.98 mmol) in toluene/CH_3CN/H_2O (6 mL/9 mL/6 mL) was added CAN (8.20 g, 14.9 mmol). After stirring for 1 h at rt, the solution was poured into ice water and extracted with CH_2Cl_2. The organic layer was washed with saturated aqueous $NaHCO_3$ and brine, respectively, and was then dried over Na_2SO_4 and concentrated. The residue was purified by flash chromatography (petroleum ether/EtOAc = 1.5:1) to afford the corresponding hemiacetal (810 mg, 91%) as a

colorless syrup.

To a solution of the above hemiacetal (367 mg, 1.24 mmol) in CH_2Cl_2 (5 mL) were added 2-(cyclopropylethynyl)benzoic acid (345 mg, 1.86 mmol)[5], DMAP (227 mg, 1.86 mmol), EDCI (428 mg, 2.23 mmol), and 5 Å MS (500 mg). The mixture was stirred at rt for 3.5 h, and then filtered. The filtrate was diluted with CH_2Cl_2, washed with saturated aqueous $NaHCO_3$ and brine, respectively. The organic phase was dried over Na_2SO_4 and concentrated. The residue was purified by flash chromatography (petroleum ether/EtOAc = 5:1) to afford **4** (559 mg, 97%, β/α = 1.6/1) as a syrup. **4α**: $[\alpha]^{26}_{D}$ = +117.9 (*c* = 0.9, $CHCl_3$); ^{1}H NMR (400 MHz, $CDCl_3$) δ 7.92 (d, *J* = 7.8 Hz, 1H), 7.52-7.44 (m, 2H), 7.36-7.32 (m, 1H), 6.58 (d, *J* = 3.6 Hz, 1H), 5.53 (d, *J* = 2.5 Hz, 1H), 5.25 (dd, *J* = 10.5, 3.7 Hz, 1H), 4.45 (q, *J* = 6.4 Hz, 1H), 4.22 (s, 2H), 3.93 (dd, *J* = 10.5, 3.2 Hz, 1H), 3.41 (s, 3H), 2.04 (s, 3H), 1.49-1.43 (m, 1H), 1.22 (d, *J* = 6.5 Hz, 3H), 0.95-0.85 (m, 4H); ^{13}C NMR (100 MHz, $CDCl_3$) δ 170.3, 167.5, 164.6, 135.0, 132.2, 131.0, 130.8, 127.4, 124.8, 99.5, 91.1, 75.7, 75.1, 71.5, 68.5, 67.9, 57.9, 40.8, 20.9, 16.4, 9.1, 9.1, 0.8; HRMS (ESI) calcd for $C_{23}H_{25}O_8ClNa$ $[M+Na]^+$ 487.1130, found 487.1122. **4β**: $[\alpha]^{26}_{D}$ = −7.0 (*c* = 1.0, $CHCl_3$); ^{1}H NMR (400 MHz, $CDCl_3$) δ 7.95 (d, *J* = 7.8 Hz, 1H), 7.49-7.41 (m, 2H), 7.30-7.27 (m, 1H), 5.85 (d, *J* = 8.4 Hz, 1H), 5.45 (d, *J* = 3.1 Hz, 1H), 5.31 (dd, *J* = 9.9, 8.6 Hz, 1H), 4.24 (d, *J* = 0.9 Hz, 2H), 3.97 (q, *J* = 6.3 Hz, 1H), 3.50 (dd, *J* = 10.1, 3.4 Hz, 1H), 3.40 (s, 3H), 2.01 (s, 3H), 1.57-1.50 (m, 1H), 1.28 (d, *J* = 6.4 Hz, 3H), 0.90 (d, *J* = 6.7 Hz, 4H); ^{13}C NMR (100 MHz, $CDCl_3$) δ 169.9, 167.5, 163.7, 134.6, 132.6, 131.1, 129.5, 127.3, 125.9, 100.5, 92.7, 80.0, 74.5, 70.7, 70.4, 69.5, 58.4, 40.9, 20.9, 16.3, 9.1, 9.1, 0.9; HRMS (ESI) calcd for $C_{23}H_{25}O_8ClNa$ $[M+Na]^+$ 487.1130, found 487.1123.

Synthesis of tetrasaccharide donor 2

p-Methoxyphenyl 3,4-di-*O*-*tert*-butyldiphenylsilyl-2,6-dideoxy-β-D-*ribo*-hexopyranoside (12)

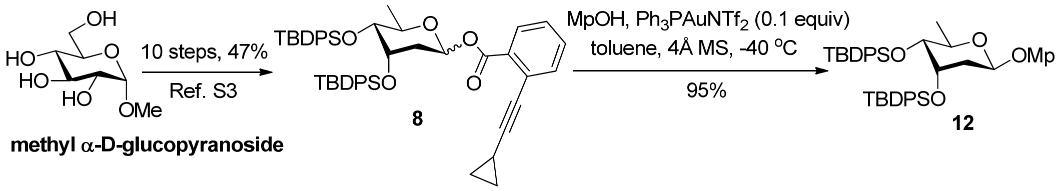

To a solution of **8**[6] (4.05 g, 5.10 mmol) and *p*-methoxyphenol (MpOH) (1.27 g, 10.2 mmol) in toluene (10 mL) was added 4 Å MS (5.0 g) at rt. After stirring for 30 min at –40 °C, PPh_3AuNTf_2 (377 mg, 0.51 mmol) was added to the mixture. After stirring for another 2 h at this temperature, Et_3N was added to quench the reaction. The resulting mixture was filtered and concentrated. The residue was purified by flash chromatography (petroleum ether/EtOAc = 80:1) to afford **12** (3.54 g, 95%) as a white foam: $[\alpha]^{26}_D = -31.7$ (c = 2.6, $CHCl_3$); ^{1}H NMR (400 MHz, $CDCl_3$) δ 7.71-7.58 (m, 8H), 7.41-7.27 (m, 10H), 7.24-7.19 (m, 2H), 6.77 (dd, J = 21.6, 9.1 Hz, 4H), 5.43 (brs, 1H), 4.57-4.33 (brs, 1H), 4.13-4.08 (m, 1H), 3.72 (s, 3H), 3.63 (brs, 1H), 2.34 (brs, 1H), 1.73-1.68 (m, 1H), 1.13-0.94 (m, 18H), 0.80 (brs, 3H); ^{13}C NMR (100 MHz, $CDCl_3$) δ 154.6, 151.5, 136.3, 136.2, 136.2, 134.0, 133.9, 133.6, 129.9, 129.8, 129.7, 127.8, 127.7, 127.7, 117.6, 114.4, 97.5, 75.5, 55.7, 27.4, 27.3, 19.6, 19.5, 19.0; HRMS (ESI) calcd for $C_{45}H_{54}O_5Si_2Na$ $[M+Na]^+$ 753.3402, found 753.3390.

***p*-Methoxyphenyl 3-*O*-acetyl-2,6-dideoxy-β-D-ribo-hexopyranoside (13)**

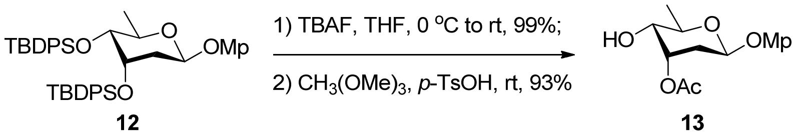

To a solution of **12** (1.68 g, 2.30 mmol) in THF (10 mL) was added TBAF (1 M in THF, 9.2 mL, 9.2 mmol) at 0 °C. After stirring for 14 h at rt, the solution was diluted with CH_2Cl_2. The mixture was washed with water and brine, respectively, and was then dried over Na_2SO_4 and concentrated. The residue was purified by flash chromatography (petroleum ether/EtOAc = 1.5:1) to afford a syrup (562 mg, 99%).

The above syrup (230 mg, 0.90 mmol) was dissolved in CH_2Cl_2 (3 mL). Trimethylorthoacetate (0.35 mL, 2.71 mmol) and a catalytic amount of *p*-toluenesulfonic acid (7.8 mg, 0.045 mmol) were added at rt. The mixture was stirred for 3 h, and TLC indicated that the starting material was consumed. The

solvent was removed and the residue was dissolved in THF/H_2O (4 mL, 1:1). *p*-Toluenesulfonic acid (78 mg, 0.45 mmol) was added, and the stirring was continued for 1 h. The reaction was quenched with saturated $NaHCO_3$ solution. The mixture was extracted with CH_2Cl_2. The combined organic layer was washed with brine, dried over Na_2SO_4, and then concentrated. The residue was purified by flash chromatography (petroleum ether/EtOAc = 3:1) to afford **13** (248 mg, 93%) as a colorless syrup: $[\alpha]^{27}_{D} = +7.0$ (c = 1.5, $CHCl_3$); ^1H NMR (400 MHz, $CDCl_3$) δ 6.98 (d, J = 8.4 Hz, 2H), 6.82 (d, J = 8.6 Hz, 2H), 5.37 (d, J = 2.6 Hz, 1H), 5.27 (d, J = 9.1 Hz, 1H), 3.89-3.83 (m, 1H), 3.77 (s, 3H), 3.53 (d, J = 3.6 Hz, 1H), 2.28 (d, J = 14.1 Hz, 1H), 2.19 (d, J = 6.1 Hz, 1H), 2.13 (s, 3H), 2.09-2.04 (m, 1H), 1.36 (d, J = 6.1 Hz, 3H); ^{13}C NMR (100 MHz, $CDCl_3$) δ 171.3, 155.1, 151.3, 117.9, 114.6, 97.0, 72.1, 70.9, 70.8, 55.8, 35.5, 21.3, 18.3; HRMS (ESI) calcd for $C_{15}H_{20}O_6Na$ $[M+Na]^+$ 319.1152, found 319.1150.

Disaccharide 14

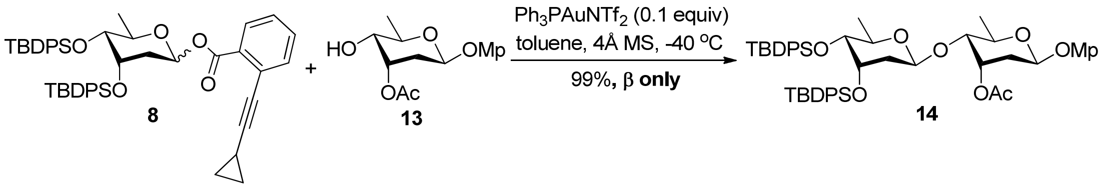

To a solution of **8** (2.55 g, 3.22 mmol) and **13** (636 mg, 2.15 mmol) in toluene (15 mL) was added 4 Å MS at rt. After stirring for 30 min at –40 °C, PPh_3AuNTf_2 (159 mg, 0.215 mmol) was added to the mixture. After stirring for another 2 h at this temperature, Et_3N was added to quench the reaction. The resulting mixture was filtered and concentrated. The residue was purified by flash chromatography (petroleum ether/EtOAc = 12:1) to afford **14** (1.92 g, 99%) as a white foam: $[\alpha]^{26}_{D}$ = +19.8 (c = 1.2, $CHCl_3$); ^1H NMR (400 MHz, $CDCl_3$) δ 7.73-7.65 (m, 6H), 7.51 (d, J = 7.0 Hz, 2H), 7.476-7.30 (m, 10H), 7.24-7.19 (m, 2H), 6.95 (d, J = 8.7 Hz, 2H), 6.80 (d, J = 8.7 Hz, 2H), 5.21 (s, 1H), 5.16 (d, J = 8.7 Hz, 1H), 4.76 (d, J = 8.1 Hz, 1H), 4.33 (s, 1H), 4.09 (brs, 1H), 3.86-3.83 (m, 1H), 3.75 (s, 3H), 3.42 (d, 1H), 3.03 (d, J = 7.6 Hz, 1H), 2.26 (d, J = 13.9 Hz, 1H), 1.94-1.87 (m, 5H), 1.47-1.42 (m, 1H), 1.18 (d, J = 6.1 Hz, 3H), 1.10 (s, 9H), 0.93 (s, 9H), 0.77 (d, J = 6.1 Hz, 3H); ^{13}C NMR (100

MHz, $CDCl_3$) δ 170.3, 155.1, 151.4, 136.3, 136.2, 136.2, 134.4, 134.0, 133.9, 133.7, 130.0, 129.8, 129.8, 127.8, 127.7, 127.7, 127.6, 117.9, 114.6, 97.1, 79.0, 75.9, 69.7, 68.9, 55.8, 35.3, 27.4, 27.3, 21.4, 19.8, 19.5, 18.6, 18.5; HRMS (ESI) calcd for $C_{53}H_{66}O_9Si_2Na$ $[M+Na]^+$ 925.4138, found 925.4123.

Disaccharide S8

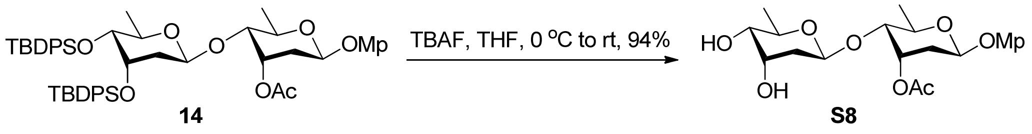

To a solution of **14** (1.92 g, 2.13 mmol) in THF (8 mL) was added TBAF (1 M in THF, 13.6 mL, 13.6 mmol) at 0 °C. After stirring for 30 h at rt, the solution was diluted with CH_2Cl_2. The mixture was washed with water and brine, dried over Na_2SO_4, and then concentrated. The residue was purified by flash chromatography (petroleum ether/EtOAc = 1.5:1) to afford **S8** (863 mg, 94%) as a white foam: $[\alpha]^{26}_D$ = +31.7 (*c* = 1.1, $CHCl_3$); ^1H NMR (400 MHz, $CDCl_3$) δ 6.96 (d, *J* = 8.4 Hz, 2H), 6.81 (d, *J* = 8.5 Hz, 2H), 5.45 (d, *J* = 2.2 Hz, 1H), 5.23 (d, *J* = 8.9 Hz, 1H), 4.86 (d, *J* = 9.5 Hz, 1H), 4.09 (brs, 1H), 4.02-3.98 (m, 1H), 3.76 (s, 3H), 3.72-3.67 (m, 1H), 3.43 (d, *J* = 7.0 Hz, 1H), 3.27 (d, *J* = 8.0 Hz, 1H), 2.50 (brs, 1H), 2.30 (d, *J* = 14.2 Hz, 2H), 2.15-1.97 (m, 5H), 1.76-1.68 (m, 1H), 1.32 (d, *J* = 6.1 Hz, 3H), 1.24 (d, *J* = 5.7 Hz, 3H); ^{13}C NMR (100 MHz, $CDCl_3$) δ 170.4, 155.1, 151.4, 117.9, 114.6, 98.8, 97.2, 79.2, 72.9, 69.8, 69.7, 69.4, 68.2, 55.8, 37.8, 35.6, 21.4, 18.5, 18.1; HRMS (ESI) calcd for $C_{21}H_{30}O_9Na$ $[M+Na]^+$ 449.1782, found 449.1788.

Disaccharide 15

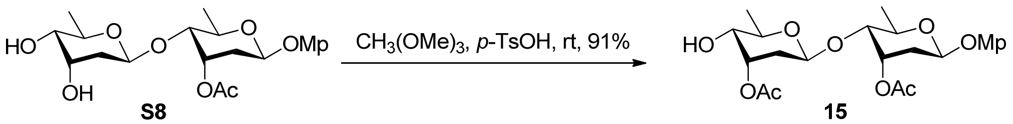

The white foam **S8** (863 mg, 2.02 mmol) was dissolved in CH_2Cl_2 (5 mL). Trimethylorthoacetate (0.77 mL, 6.07 mmol) and a catalytic amount of *p*-toluenesulfonic acid (17 mg, 0.10 mmol) were added at rt. The mixture was stirred for 1 h, and TLC indicated that the starting material was consumed. The solvent was removed and the residue was dissolved in THF/H_2O (10 mL, 1:1). *p*-Toluenesulfonic

acid (170 mg, 1.01 mmol) was added, and the stirring was continued for 1 h. The reaction was quenched with saturated $NaHCO_3$ solution. The mixture was extracted with CH_2Cl_2. The combined organic layer was washed with brine, dried over Na_2SO_4, and concentrated. The residue was purified by flash chromatography (petroleum ether/EtOAc = 1:1) to afford **15** (866 mg, 91%) as a white foam: $[\alpha]^{27}_D$ = +31.5 (*c* = 0.9, $CHCl_3$); ^1H NMR (400 MHz, $CDCl_3$) δ 6.97 (d, *J* = 9.0 Hz, 2H), 6.81 (d, *J* = 9.1 Hz, 2H), 5.46 (d, *J* = 3.3 Hz, 1H), 5.29-5.23 (m, 2H), 4.77 (dd, *J* = 9.6, 1.7 Hz, 1H), 4.04-3.97 (m, 1H), 3.76 (s, 3H), 3.70-3.63 (m, 1H), 3.43 (dd, *J* = 9.0, 3.1 Hz, 1H), 3.39-3.37 (m, 1H), 2.31 (ddd, *J* = 14.1, 4.0, 2.2 Hz, 1H), 2.14 (s, 3H), 2.11-2.07 (m, 4H), 2.04-1.97 (m, 2H), 1.84-1.77 (m, 1H), 1.32 (d, *J* = 6.3 Hz, 3H), 1.26 (d, *J* = 6.3 Hz, 3H); ^{13}C NMR (100 MHz, $CDCl_3$) δ 171.4, 170.3, 155.1, 151.4, 117.9, 114.7, 98.7, 97.2, 79.3, 72.2, 71.2, 70.4, 69.7, 69.5, 55.8, 36.0, 35.6, 21.4, 21.3, 18.5, 18.0; HRMS (ESI) calcd for $C_{23}H_{32}O_{10}Na$ $[M+Na]^+$ 491.1888, found 491.1876.

Trisaccharide 16

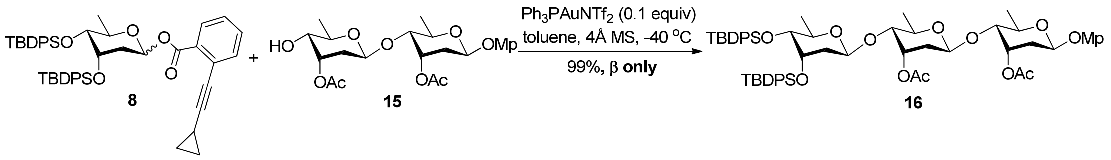

To a solution of **8** (2.40 g, 3.03 mmol) and **15** (887 mg, 1.89 mmol) in toluene (15 mL) was added 4Å MS at rt. After stirring for 30 min at –40 °C, PPh_3AuNTf_2 (140 mg, 0.189 mmol) was added to the mixture. After stirring for another 2 h at this temperature, Et_3N was added to quench the reaction. The resulting mixture was filtered and concentrated. The residue was purified by flash chromatography (petroleum ether/EtOAc = 8:1) to afford **16** (2.02 g, 99%) as a white foam: $[\alpha]^{25}_D$ = +28.7 (*c* = 2.2, $CHCl_3$); ^1H NMR (400 MHz, $CDCl_3$) δ 7.74-7.66 (m, 6H), 7.51-7.30 (m, 12H), 7.21 (t, *J* = 7.5 Hz, 2H), 6.98 (d, *J* = 9.1 Hz, 2H), 6.82 (d, *J* = 9.1 Hz, 2H), 5.48 (d, *J* = 3.1 Hz, 1H), 5.25 (dd, *J* = 9.1, 1.7 Hz, 1H), 5.13 (d, *J* = 2.8 Hz, 1H), 4.79 (d, *J* = 7.6 Hz, 1H), 4.66 (d, *J* = 9.4 Hz, 1H), 4.31 (s, 1H), 4.08 (brs, 1H), 4.02-3.98 (m, 1H), 3.77 (s, 3H), 3.73-3.69 (m, 1H), 3.43-3.38 (m, 2H), 2.93 (d, 1H), 2.31 (dd, *J* = 10.4, 2.2 Hz, 1H), 2.12 (s, 3H), 2.10-1.96 (m, 6H), 1.67-1.63 (m, 1H), 1.45-1.40 (m, 1H), 1.30 (d, *J* = 6.2 Hz, 3H), 1.11 (d, *J* = 5.9 Hz, 3H), 1.11 (s, 9H), 0.92 (s, 9H), 0.75

(d, J = 6.3 Hz, 3H); ^{13}C NMR (100 MHz, $CDCl_3$) δ 170.3, 170.2, 155.1, 151.4, 136.3, 136.2, 136.1, 134.2, 134.0, 133.7, 130.0, 129.8, 129.7, 127.9, 127.7, 127.6, 127.6, 117.9, 114.6, 98.9, 97.1, 79.3, 79.1, 77.4, 75.9, 69.6, 69.5, 69.1, 55.7, 35.8, 35.6, 27.4, 27.3, 21.4, 19.7, 19.4, 18.6, 18.4, 18.2; HRMS (ESI) calcd for $C_{61}H_{78}O_{13}Si_2Na$ $[M+Na]^+$ 1097.4873, found 1097.4847.

Trisaccharide 17

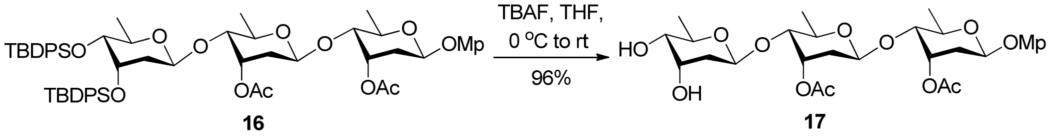

To a solution of **16** (1.92 g, 1.78 mmol) in THF (5 mL) was added TBAF (1 M in THF, 9.9 mL, 9.9 mmol) at 0 °C. After stirring for 12 h at rt, the solution was diluted with CH_2Cl_2, washed with water and brine, dried over Na_2SO_4, and concentrated. The residue was purified by flash chromatography (petroleum ether/EtOAc = 2:1) to afford **17** (1.02 g, 96%) as a white foam: $[\alpha]^{25}_D$ = +48.8 (c = 1.3, $CHCl_3$); ^{1}H NMR (400 MHz, $CDCl_3$) δ 6.95 (d, J = 9.0 Hz, 2H), 6.80 (d, J = 9.1 Hz, 2H), 5.45 (d, J = 3.0 Hz, 1H), 5.34 (d, J = 2.9 Hz, 1H), 5.22 (dd, J = 9.0, 1.6 Hz, 1H), 4.81 (d, J = 8.1 Hz, 1H), 4.71 (d, J = 8.2 Hz, 1H), 4.05 (d, J = 2.9 Hz, 1H), 4.01-3.94 (m, 1H), 3.83-3.78 (m, 1H), 3.75 (s, 3H), 3.68-3.61 (m, 1H), 3.40 (dd, J = 9.0, 3.0 Hz, 1H), 3.25 (ddd, J = 17.2, 9.5, 2.9 Hz, 2H), 2.58 (brs, 1H), 2.40 (brs, 1H), 2.30-2.26 (m, 1H), 2.09 (s, 3H), 2.08 (s, 3H), 2.05-1.91 (m, 3H), 1.76-1.62 (m, 2H), 1.29 (d, J = 6.2 Hz, 3H), 1.21 (d, J = 6.0 Hz, 6H); ^{13}C NMR (100 MHz, $CDCl_3$) δ 170.5, 170.4, 155.1, 151.4, 117.9, 114.6, 98.9, 98.8, 97.1, 79.3, 79.2, 72.9, 70.1, 69.7, 69.4, 69.2, 68.2, 55.8, 37.8, 36.0, 35.6, 21.5, 21.4, 18.4, 18.1; HRMS (ESI) calcd for $C_{29}H_{42}O_{13}Na$ $[M+Na]^+$ 621.2518, found 621.2503.

Trisaccharide 18

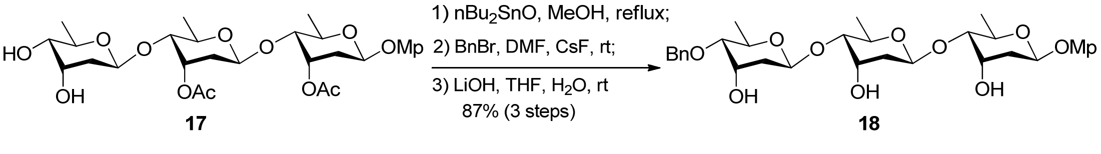

A solution of **17** (88 mg, 0.147 mmol) in methanol (3 mL) was refluxed in the presence of dibutyltin oxide (44 mg, 0.177 mmol) for 4 h. The solvent was then

removed in vacuo. The residue was dried azeotropically with toluene and then dissolved in DMF (5 mL). Benzyl bromide (0.034 mL, 0.294 mmol) and CsF (45 mg, 0.294 mmol) were added, the resulting mixture was stirred at rt for 12 h. The solution was diluted with CH_2Cl_2, washed with water and brine, dried over Na_2SO_4, and concentrated. The residue was purified by flash chromatography (petroleum ether/EtOAc = 2:1) to provide a mixture of acetyl group transfer products as a white foam.

To a solution of the above mixture in THF/H_2O (6 mL, 5:1) at rt was added LiOH (35 mg, 1.47 mmol). The mixture was stirred overnight and was then quenched with addition of the pH = 7.0 buffering solution (8 mL). The mixture was extracted with CH_2Cl_2. The combined organic layer was washed with brine, dried over Na_2SO_4 and concentrated. The residue was purified by flash chromatography (petroleum ether/EtOAc = 1:1) to afford **18** (77 mg, 87%) as a white solid: $[\alpha]^{28}_D$ = +11.6 (*c* = 1.0, $CHCl_3$); ^{1}H NMR (400 MHz, $CDCl_3$) δ 7.39-7.31 (m, 5H), 6.96 (d, *J* = 9.1 Hz, 2H), 6.80 (d, *J* = 9.1 Hz, 2H), 5.39 (dd, *J* = 9.1, 2.0 Hz, 1H), 4.91 (d, *J* = 9.4 Hz, 2H), 4.63 (d, *J* = 11.4 Hz, 1H), 4.53 (d, *J* = 11.4 Hz, 1H), 4.32 (s, 1H), 4.24-4.21 (m, 2H), 3.96-3.89 (m, 1H), 3.88-3.79 (m, 2H), 3.75 (s, 3H), 3.32 (dd, *J* = 8.9, 2.9 Hz, 1H), 3.20 (dd, *J* = 9.4, 2.8 Hz, 1H), 3.11 (dd, *J* = 9.4, 2.8 Hz, 1H), 3.06 (s, 1H), 2.96 (s, 1H), 2.43 (d, *J* = 1.6 Hz, 1H), 2.30-2.25 (m, 1H), 2.19-2.11 (m, 2H), 1.99-1.93 (m, 1H), 1.76-1.64 (m, 2H), 1.27 (d, *J* = 3.7 Hz, 3H), 1.26 (d, *J* = 3.6 Hz, 3H), 1.23 (d, *J* = 6.2 Hz, 3H); ^{13}C NMR (100 MHz, $CDCl_3$) δ 154.9, 151.4, 137.4, 128.8, 128.4, 128.2, 117.9, 114.5, 98.5, 98.4, 96.7, 82.4, 82.3, 80.2, 72.0, 68.8, 68.6, 68.4, 66.5, 66.2, 64.7, 55.8, 36.9, 36.8, 36.5, 18.5, 18.4, 18.3; HRMS (ESI) calcd for $C_{32}H_{44}O_{11}Na$ $[M+Na]^+$ 627.2776, found 627.2768.

Trisaccharide 19

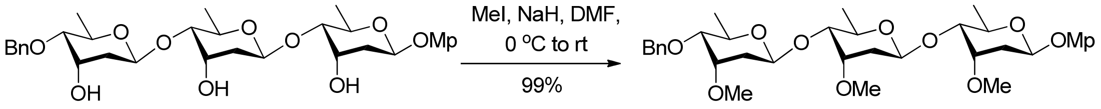

18 19

To a solution of **18** (717 mg, 1.19 mmol) in DMF (10 mL) were added sodium hydride (60% in oil; 381 mg, 9.52 mmol) and methyl iodide (0.37 mL, 5.93 mmol) at

0 °C. The mixture was stirred for 3 h while warming to rt. The solution was quenched with MeOH, diluted with CH_2Cl_2, washed with brine, dried over Na_2SO_4, and concentrated. The residue was purified by flash chromatography (petroleum ether/EtOAc = 4:1) to afford **19** (760 mg, 99%) as a colorless syrup: $[\alpha]^{28}_D$ = +28.7 (*c* = 1.1, $CHCl_3$); ^1H NMR (400 MHz, $CDCl_3$) δ 7.35-7.26 (m, 5H), 6.96 (d, *J* = 9.1 Hz, 2H), 6.80 (d, *J* = 9.1 Hz, 2H), 5.31 (dd, *J* = 9.3, 1.7 Hz, 1H), 4.79-4.75 (m, 2H), 4.65 (d, *J* = 11.9 Hz, 1H), 4.52 (d, *J* = 11.8 Hz, 1H), 4.03-3.96 (m, 1H), 3.93-3.84 (m, 3H), 3.81 (d, *J* = 2.7 Hz, 1H), 3.76-3.74 (m, 4H), 3.45 (s, 3H), 3.44 (s, 3H), 3.43 (s, 3H), 3.31 (dd, *J* = 9.4, 2.8 Hz, 1H), 3.23 (dd, *J* = 9.6, 2.8 Hz, 1H), 3.10 (dd, *J* = 9.4, 2.7 Hz, 1H), 2.32-2.28 (m, 1H), 2.20-2.13 (m, 2H), 1.83 (ddd, *J* = 13.4, 9.6, 2.3 Hz, 1H), 1.68-1.54 (m, 2H), 1.27 (d, *J* = 3.7 Hz, 3H), 1.26 (d, *J* = 3.6 Hz, 3H), 1.23 (d, *J* = 6.2 Hz, 3H); ^{13}C NMR (100 MHz, $CDCl_3$) δ 154.9, 151.5, 138.1, 128.5, 128.0, 127.9, 117.7, 114.5, 99.7, 99.7, 96.9, 82.5, 82.1, 80.7, 77.1, 76.8, 74.1, 71.5, 69.0, 69.0, 68.6, 58.1, 58.0, 58.0, 55.7, 35.3, 35.0, 34.9, 18.6, 18.4, 18.3; HRMS (ESI) calcd for $C_{35}H_{50}O_{11}Na$ $[M+Na]^+$ 669.3245, found 669.3237.

Cymarose trisaccharide 5

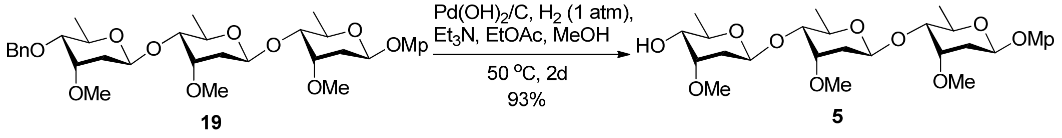

To a solution of **19** (315 mg, 0.487 mmol) in EtOAc/MeOH (12 mL, 2:1) were added $Pd(OH)_2/C$ (315 mg) and Et_3N (0.68 mL, 4.87 mmol). The suspension was stirred under hydrogen pressure (1 atm) for 2 days at 50 °C and then filtered. The filtrate was concentrated. The residue was purified by flash chromatography (petroleum ether/EtOAc = 1:1) to afford **5** (252 mg, 93%) as a white solid: $[\alpha]^{28}_D$ = +22.1 (*c* = 1.5, $CHCl_3$); ^1H NMR (400 MHz, $CDCl_3$) δ 6.95 (d, *J* = 9.0 Hz, 2H), 6.79 (d, *J* = 9.0 Hz, 2H), 5.30 (dd, *J* = 9.4, 1.7 Hz, 1H), 4.78 (d, *J* = 8.2 Hz, 1H), 4.68 (d, *J* = 8.3 Hz, 1H), 4.03-3.96 (m, 1H), 3.90-3.87 (m, 2H), 3.81 (d, *J* = 2.6 Hz, 1H), 3.75 (s, 3H), 3.61 (d, *J* = 2.9 Hz, 1H), 3.58-3.52 (m, 1H), 3.45 (s, 3H), 3.44 (s, 3H), 3.42 (s, 3H), 3.31 (dd, *J* = 9.4, 2.7 Hz, 1H), 3.23-3.16 (m, 2H), 2.32-2.23 (m, 3H), 2.14 (d, *J* = 12.9 Hz, 1H), 1.85-1.79 (m, 1H), 1.68-1.59 (m, 2H), 1.26 (d, *J* = 6.2 Hz, 6H), 1.22 (d, *J* = 6.2 Hz,

3H); ^{13}C NMR (100 MHz, $CDCl_3$) δ 154.9, 151.5, 117.8, 114.6, 99.8, 99.6, 96.9, 82.6, 82.2, 77.6, 76.8, 72.6, 70.8, 69.1, 68.6, 58.2, 58.0, 57.3, 55.8, 35.5, 34.9, 33.9, 18.4, 18.4, 18.3; HRMS (ESI) calcd for $C_{28}H_{44}O_{11}Na$ $[M+Na]^+$ 579.2776, found 579.2772.

Tetrasaccharide 20

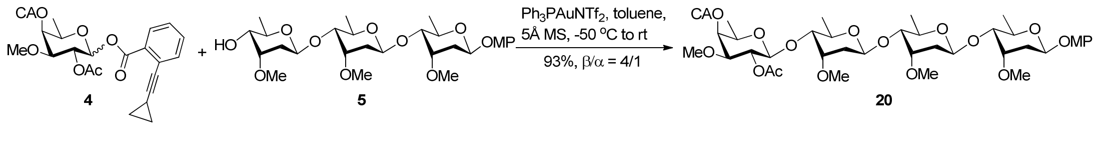

To a solution of **4** (270 mg, 0.581 mmol) and **5** (152 mg, 0.273 mmol) in toluene (5 mL) was added 5Å MS at rt. After stirring for 30 min at –50 °C, PPh_3AuNTf_2 (40 mg, 0.055 mmol) was added to the mixture. The mixture was stirred for 3 h while warming to rt, then Et_3N was added to quench the reaction. The resulting mixture was filtered and concentrated. The residue was purified by flash chromatography (petroleum ether/EtOAc = 2:1) to afford **20** (169 mg, 74%) and its α anomer (42 mg, 19%) as white foams. **20**: $[\alpha]^{30}_D$ = +19.1 (*c* = 0.5, $CHCl_3$); ^{1}H NMR (400 MHz, $CDCl_3$) δ 6.95 (d, *J* = 9.1 Hz, 2H), 6.80 (d, *J* = 9.1 Hz, 2H), 5.37 (d, *J* = 3.0 Hz, 1H), 5.31 (dd, *J* = 9.3, 1.7 Hz, 1H), 5.08 (dd, *J* = 9.9, 8.1 Hz, 1H), 4.78 (d, *J* = 9.6 Hz, 2H), 4.44 (d, *J* = 8.0 Hz, 1H), 4.20 (d, *J* = 0.9 Hz, 2H), 4.02-3.98 (m, 1H), 3.91-3.84 (m, 3H), 3.80 (d, *J* = 2.6 Hz, 1H), 3.76 (s, 3H), 3.75-3.72 (m, 2H), 3.45 (s, 3H), 3.43 (s, 6H), 3.36-3.33 (m, 1H), 3.35 (s, 3H), 3.30 (dd, *J* = 9.5, 2.8 Hz, 1H), 3.20 (ddd, *J* = 12.5, 9.7, 2.7 Hz, 2H), 2.32-2.28 (m, 1H), 2.16-2.12 (m, 2H), 2.07 (s, 3H), 1.86-1.79 (m, 1H), 1.67-1.61 (m, 2H), 1.27 (d, *J* = 6.2 Hz,3H), 1.24 (d, *J* = 6.2 Hz, 3H), 1.22 (d, *J* = 6.2 Hz, 3H), 1.17 (d, *J* = 6.2 Hz, 3H); ^{13}C NMR (100 MHz, $CDCl_3$) δ 169.5, 167.6, 154.9, 151.5, 117.7, 114.6, 102.6, 99.8, 99.7, 96.9, 84.1, 82.6, 82.2, 80.0, 77.0, 76.8, 76.4, 70.7, 70.5, 69.1, 69.1, 68.5, 68.1, 58.3, 58.2, 58.0, 58.0, 55.8, 41.0, 35.5, 35.2, 34.9, 21.0, 18.4, 18.4, 18.2, 16.7; HRMS (ESI) calcd for $C_{39}H_{59}ClO_{17}Na$ $[M+Na]^+$ 857.3333, found 857.3329.

Tetrasaccharide donor 2

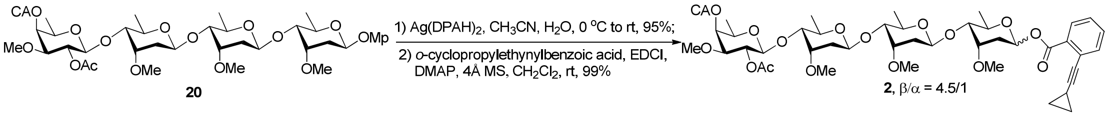

To a solution of **20** (169 mg, 0.202 mmol) in CH_3CN/H_2O (2.5 mL/2.5 mL) was

added $Ag(DPAH)_2$[7] (205 mg, 0.445 mmol) at 0 °C. After stirring for 10 min at this temperature, the mixture was filtered. The filtrate was diluted with CH_2Cl_2, washed with saturated $NaHCO_3$ solution and brine, dried over Na_2SO_4, and concentrated. The residue was purified by flash chromatography (petroleum ether/ EtOAc = 1:2) to yield the corresponding hemiacetal (140 mg, 95%) as a colorless syrup.

To a solution of the above hemiacetal (84 mg, 0.115 mmol) in CH_2Cl_2 (3 mL) were added 2-(cyclopropylethynyl)benzoic acid (43 mg, 0.230 mmol)[S2], DMAP (28 mg, 0.230 mmol), EDCI (55 mg, 0.288 mmol), and 4 Å MS (250 mg). The mixture was stirred at rt for 4 h, and then filtered. The filtrate was diluted with CH_2Cl_2, washed with saturated $NaHCO_3$ solution and brine, dried over Na_2SO_4, and concentrated. The residue was purified by flash chromatography (petroleum ether/EtOAc = 1:1) to afford **2** (102 mg, 99%, β/α = 4.5/1) as a syrup. **2β**: $[\alpha]^{31}_{D}$ = +38.7 (*c* = 0.7, $CHCl_3$); ^{1}H NMR (400 MHz, $CDCl_3$) δ 7.91 (d, *J* = 7.8 Hz, 1H), 7.46 (d, *J* = 7.6 Hz, 1H), 7.39 (t, *J* = 7.5 Hz, 1H), 7.26 (t, *J* = 7.5 Hz, 1H), 6.22 (dd, *J* = 9.3, 1.8 Hz, 1H), 5.36 (d, *J* = 3.1 Hz, 1H), 5.07 (dd, *J* = 9.9, 8.1 Hz, 1H), 4.78 (dd, *J* = 9.4, 2.3 Hz, 2H), 4.44 (d, *J* = 8.0 Hz, 1H), 4.19 (s, 2H), 4.15-4.08 (m, 1H), 3.91-3.84 (m, 3H), 3.81 (d, *J* = 2.4 Hz, 1H), 3.76-3.71 (m, 2H), 3.47 (s, 3H), 3.44 (s, 6H), 3.36-3.31 (m, 2H), 3.35 (s, 3H), 3.20 (ddd, *J* = 15.5, 9.6, 2.6 Hz, 2H), 2.35-2.31 (m, 1H), 2.16-2.11 (m, 2H), 2.07 (s, 3H), 1.84-1.78 (m, 1H), 1.69-1.61 (m, 2H), 1.54-1.47 (m, 1H), 1.27 (d, *J* = 6.5 Hz, 3H), 1.25 (d, *J* = 6.5 Hz, 3H), 1.22 (d, *J* = 6.2 Hz, 3H), 1.18 (d, *J* = 6.2 Hz, 3H), 0.88 (d, *J* = 6.2 Hz, 4H); ^{13}C NMR (100 MHz, $CDCl_3$) δ 169.5, 167.6, 164.5, 134.3, 131.9, 131.4, 130.7, 127.0, 125.1, 102.6, 99.8, 99.7, 92.1, 84.1, 82.6, 81.8, 80.1, 77.4, 76.5, 76.4, 74.7, 70.8, 70.6, 70.2, 69.2, 68.6, 68.2, 58.4, 58.2, 58.1, 57.9, 41.0, 35.7, 35.3, 33.7, 21.0, 18.4, 18.3, 18.2, 16.7, 9.0, 0.8; HRMS (ESI) calcd for $C_{44}H_{61}ClO_{17}Na$ $[M+Na]^+$ 919.3490, found 919.3494.

Synthesis of FABO disaccharide 25

p-Methoxyphenyl 4-*O*-acetyl-3-*O*-(*tert*-butyldimethyl)silyl-2,6-dideoxy-2-iodo-β-D-glucopyranoside (S10)

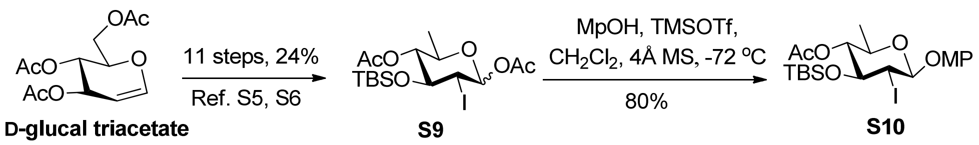

To a solution of **S9**[8,9] (1.32 g, 2.79 mmol) and *p*-methoxyphenol (624 mg, 5.03 mmol) in CH_2Cl_2 (10 mL) was added 4 Å MS (2.5 g) at rt. After stirring for 1 h at –72 °C, TMSOTf (0.43 ml, 2.24 mmol) was added to the mixture. After stirring for another 3 h at this temperature, Et_3N was added to quench the reaction. The resulting mixture was filtered, diluted with CH_2Cl_2, washed with saturated $NaHCO_3$ solution and brine, respectively, dried over Na_2SO_4, and then concentrated. The residue was purified by flash chromatography (petroleum ether/EtOAc = 40:1) to afford **S10** (1.2 g, 80%) as a syrup: $[\alpha]^{28}_{D}$ = +10.5 (*c* = 1.0, $CHCl_3$); ^{1}H NMR (400 MHz, $CDCl_3$) δ 7.06 (d, *J* = 9.0 Hz, 2H), 6.83 (d, *J* = 9.0 Hz, 2H), 5.00 (d, *J* = 8.5 Hz, 1H), 4.77 (t, *J* = 8.8 Hz, 1H), 4.05-3.97 (m, 2H), 3.77 (s, 3H), 3.53-3.49 (m, 1H), 2.11 (s, 3H), 1.23 (d, *J* = 6.2 Hz, 3H), 0.92 (s, 9H), 0.29 (s, 3H), 0.06 (s, 3H); ^{13}C NMR (100 MHz, $CDCl_3$) δ 170.0, 155.8, 151.4, 119.1, 114.6, 103.0, 77.1, 76.7, 70.6, 55.8, 36.0, 26.3, 21.7, 18.4, 17.9, -2.8, -3.3; HRMS (ESI) calcd for $C_{21}H_{33}IO_6SiNa$ $[M+Na]^+$ 559.0983, found 559.0998.

_p_-Methoxyphenyl 4-_O_-acetyl-2,6-dideoxy-2-iodo-β-D-glucopyranoside (11)

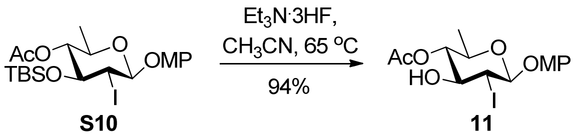

To a solution of **S10** (1.26 g, 2.36 mmol) in MeCN (10 mL) was added 3HF·Et_3N (1.90 mL, 11.8 mmol) at rt. After stirring for 10 h at 65 °C, a saturated $NaHCO_3$ solution was added slowly to the mixture at rt. The resulting mixture was diluted with EtOAc, washed with saturated $NaHCO_3$ solution, and was then extracted with EtOAc twice. The combined organic layer was washed with brine, dried over Na_2SO_4, and concentrated. The residue was purified by flash chromatography (petroleum ether/EtOAc = 4:1) to afford **11** (930 mg, 94%) as a white solid: $[\alpha]^{27}_{D}$ = +45.8 (*c* = 1.3, $CHCl_3$); ^{1}H NMR (400 MHz, $CDCl_3$) δ 7.07 (d, *J* = 9.1 Hz, 2H), 6.84 (d, *J* = 9.1 Hz, 2H), 4.99 (d, *J* = 9.0 Hz, 1H), 4.72 (t, *J* = 9.3 Hz, 1H), 4.07 (dd, *J* = 10.7, 9.1 Hz, 1H), 3.85 (td, *J* = 10.6, 3.8 Hz, 1H), 3.78 (s, 3H), 3.64-3.57 (m, 1H), 2.79 (brs, 1H),

2.13 (s, 3H), 1.29 (d, J = 6.2 Hz, 3H); ^{13}C NMR (100 MHz, $CDCl_3$) δ 170.8, 155.9, 151.3, 119.2, 114.7, 102.6, 76.5, 75.7, 70.5, 55.8, 35.8, 21.0, 17.5; HRMS (ESI) calcd for $C_{15}H_{19}IO_6Na$ $[M+Na]^+$ 445.0119, found 445.0104.

4-*O*-Benzoyl-3-*O*-methyl-6-*O*-tosyl-D-glucal (S12)

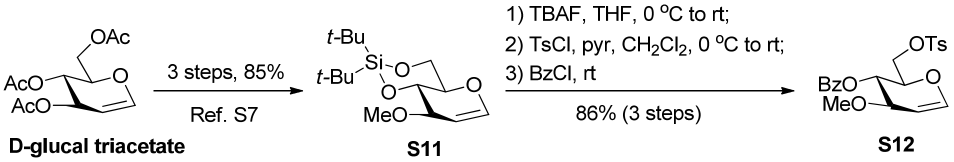

To a solution of **S11**[10] (454 mg, 1.51 mmol) in THF (10 mL) was added TBAF (1 M in THF, 3.8 mL, 3.8 mmol) at 0 °C. After stirring for 3 h at rt, the solution was concentrated. The residue was purified by flash chromatography (CH_2Cl_2/MeOH = 30:1) to afford a colorless syrup. The above syrup was dissolved in CH_2Cl_2 (5 mL) and pyridine (5 mL). TsCl (720 mg, 3.78 mmol) was added to the mixture at 0 °C. After stirring for 22 h at rt, BzCl (0.35 ml, 3.0 mmol) was added at 0 °C. After stirring for 2 h at rt, the mixture was quenched with water at 0 °C. The mixture was diluted with CH_2Cl_2, washed with saturated $NaHCO_3$ solution and brine, dried over Na_2SO_4, and then concentrated. The residue was purified by flash chromatography (petroleum ether/EtOAc = 8:1) to afford **S12** (543 mg, 86%) as a colorless syrup: $[\alpha]^{28}_{D}$ = +38.8 (c = 1.1, $CHCl_3$); ^{1}H NMR (400 MHz, $CDCl_3$) δ 7.99 (d, J = 7.1 Hz, 2H), 7.77 (d, J = 8.3 Hz, 2H), 7.58 (t, J = 7.4 Hz, 1H), 7.44 (t, J = 7.7 Hz, 2H), 7.29 (d, J = 8.1 Hz, 2H), 6.34 (d, J = 6.3 Hz, 1H), 5.30 (t, J = 4.0 Hz, 1H), 4.91-4.94 (m, 1H), 4.48-4.44 (m, 1H), 4.36 (dd, J = 11.0, 7.6 Hz, 1H), 4.22 (dd, J = 11.0, 3.5 Hz, 1H), 3.81-3.79 (m, 1H), 3.35 (s, 3H), 2.40 (s, 3H); ^{13}C NMR (100 MHz, $CDCl_3$) δ 165.3, 145.0, 144.1, 133.6, 132.9, 129.9, 129.4, 128.6, 128.1, 99.3, 73.5, 71.4, 67.2, 67.2, 56.0, 21.7; HRMS (ESI) calcd for $C_{21}H_{22}SO_7Na$ $[M+Na]^+$ 441.0978, found 441.0961.

4-*O*-Benzoyl-6-bromo-6-deoxy-3-*O*-methyl-D-glucal (21)

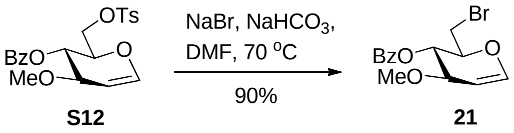

To a solution of **S12** (596 mg, 1.42 mmol) in DMF (10 mL) were added NaBr (733

mg, 7.12mmol) and $NaHCO_3$ (358 mg, 4.26 mmol) at rt. After stirring for 6 h at 70 °C, the mixture was cooled to rt and concentrated. Diluted with EtOAc, this mixture was washed with water and brine, dried over Na_2SO_4, and then concentrated. The residue was purified by flash chromatography (petroleum ether/EtOAc = 30:1) to afford **21** (420 mg, 90%) as a colorless syrup: $[\alpha]^{27}_D = -2.9$ (c = 1.0, $CHCl_3$); ^1H NMR (400 MHz, $CDCl_3$) δ 8.05 (d, J = 7.4 Hz, 2H), 7.59 (t, J = 7.4 Hz, 1H), 7.45 (t, J = 7.7 Hz, 2H), 6.51 (d, J = 6.3 Hz, 1H), 5.56 (t, J = 4.3 Hz, 1H), 5.00 (dd, J = 5.9, 4.4 Hz, 1H), 4.45 (dd, J = 12.0, 5.9 Hz, 1H), 3.89 (t, J = 3.8 Hz, 1H), 3.73-3.61 (m, 2H), 3.44 (s, 3H); ^{13}C NMR (100 MHz, $CDCl_3$) δ 165.4, 144.3, 133.6, 130.0, 129.5, 128.6, 99.6, 75.4, 72.1, 68.4, 56.2, 29.7; HRMS (ESI) calcd for $C_{14}H_{15}BrO_4Na$ $[M+Na]^+$ 349.0046, found 349.0032.

4-*O*-Benzoyl-6-bromo-2,6-dideoxy-3-*O*-methyl-2-(phenyl)seleno-D-mannopyranoside (22)

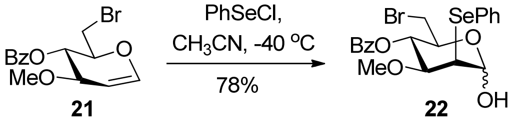

To a stirring solution of **21** (1.77 g, 5.41 mmol) in CH_3CN (30 mL) was added a solution of PhSeCl (1.14 g, 5.95 mmol) in CH_3CN (10 mL) dropwise at –40 °C. The reaction was monitored by TLC until no glycal remained, then saturated $NaHCO_3$ solution was added. The mixture was extracted with CH_2Cl_2. The combined organic layer was washed with brine, dried over Na_2SO_4, and concentrated. The residue was purified by flash chromatography (petroleum ether/EtOAc = 8:1) to afford **22** (2.1 g, 78%) as a white solid. **22*α***: $[\alpha]^{27}_D = +6.9$ (c = 1.0, $CHCl_3$); ^1H NMR (400 MHz, $CDCl_3$) δ 8.06 (d, J = 8.4 Hz, 2H), 7.70-7.66 (m, 2H), 7.59 (t, J = 7.4 Hz, 1H), 7.46 (t, J = 7.7 Hz, 2H), 7.28-7.25 (m, 3H), 5.55 (t, J = 3.0 Hz, 1H), 5.40 (t, J = 8.5 Hz, 1H), 4.35-4.30 (m, 1H), 4.07 (dd, J = 8.1, 4.4 Hz, 1H), 3.83 (dd, J = 4.4, 2.7 Hz, 1H), 3.58-3.47 (m, 2H), 3.31 (s, 3H), 3.16 (d, J = 3.7 Hz, 1H); ^{13}C NMR (100 MHz, $CDCl_3$) δ 165.7, 135.3, 133.6, 130.0, 129.6, 129.3, 129.1, 128.6, 128.1, 95.1, 78.2, 71.6, 71.4, 57.8, 48.3, 32.2; MS (ESI) $[M+Na]^+$ 523.0.

Phenylthiomethyl 4-*O*-benzoyl-6-bromo-2,6-dideoxy-3-*O*-methyl-2-(phenyl)seleno-α-D-mannopyranoside (10)

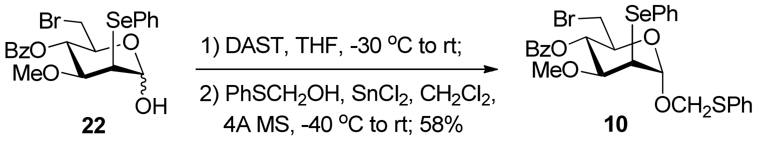

To a solution of **22** (671 mg, 1.34 mmol) in THF (5 mL) was added dimethylaminosulfur trifluoride (DAST) (0.41 mL, 3.35 mmol) at –30 °C. After stirring for 3 h at rt, a saturated $NaHCO_3$ solution was added slowly to the mixture. The resulting mixture was extracted with CH_2Cl_2. The combined organic layer was washed with brine, dried over Na_2SO_4, and concentrated. The crude glycosyl fluoride was azeotroped with toluene (3 x 5 mL). After drying under high vacuum for at least 2 h, the above product was dissolved in CH_2Cl_2 (8 mL) and 4 Å MS (1.5 g) was added. The reaction mixture was stirred at –40 °C for 30 min before $PhSCH_2OH$[11] (366 mg, 2.68 mmol) and $SnCl_2$ (381 mg, 2.01 mmol) were added. The reaction mixture was allowed to warm to rt and stirred for 1 h. The reaction mixture was quenched with Et_3N (1 mL) and filtered. The solution was diluted with EtOAc, and washed with water. The water layer was extracted with EtOAc twice. The combined organic layer was washed with saturated $NaHCO_3$ solution and brine, dried over Na_2SO_4, and then concentrated. The residue was purified by flash chromatography (petroleum ether/EtOAc = 60:1) to afford **10** (480 mg, 58%) as a syrup: $[\alpha]^{28}_D = +121.9$ (c = 1.1, $CHCl_3$); ^1H NMR (400 MHz, $CDCl_3$) δ 8.04 (d, J = 7.2 Hz, 2H), 7.63-7.58 (m, 3H), 7.46 (t, J = 7.7 Hz, 2H), 7.38 (dd, J = 7.9, 1.4 Hz, 2H), 7.30-7.23 (m, 6H), 5.56 (d, J = 1.8 Hz, 1H), 5.33 (t, J = 8.9 Hz, 1H), 5.13 (d, J = 11.7 Hz, 1H), 5.06 (d, J = 11.8 Hz, 1H), 4.05-4.00 (m, 1H), 3.96 (dd, J = 8.4, 4.7 Hz, 1H), 3.77 (dd, J = 4.5, 2.1 Hz, 1H), 3.50-3.47 (m, 2H), 3.29 (s, 3H); ^{13}C NMR (100 MHz, $CDCl_3$) δ 165.6, 135.2, 135.0, 133.6, 130.8, 130.0, 129.6, 129.3, 129.1, 129.0, 128.6, 128.1, 127.3, 97.1, 78.5, 71.8, 71.7, 71.7, 57.7, 47.4, 31.9; MS (ESI) $[M+Na]^+$ 645.0.

Acetal glycoside 24

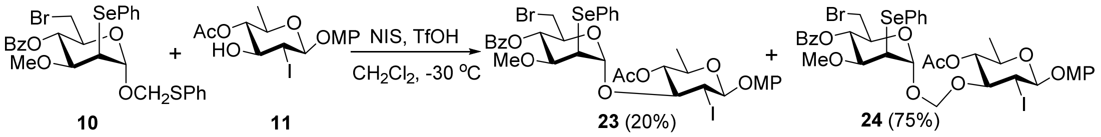

To a solution of **11** (620 mg, 1.47 mmol) in CH_2Cl_2 (5 mL) was added acid washed 3 Å MS at rt. After stirring for 30 min at rt, NIS (142 mg, 0.631 mmol) and TfOH (2.6 µL, 0.0294 mmol) were added to the mixture at –30 ºC, followed by addition of a solution of **10** (183 mg, 0.294 mmol) in CH_2Cl_2 (3 mL) via a syringe pump. The reaction mixture was stirred at –30 °C for 1 h. When TLC showed the donor had been consumed, saturated aqueous $NaHCO_3$ was added at 0 ºC. The mixture was filtered. The filtrate was washed with a solution of NaS_2O_3 and brine, respectively, and was then dried over Na_2SO_4 and concentrated. The residue was purified by flash chromatography (petroleum ether/ CH_2Cl_2/EtOAc = 8:1:1) to afford **24** (206 mg, 75%) and **23** (53 mg, 20%) as syrups. **24**: $[\alpha]^{28}_{D}$ = +53.4 (*c* = 1.2, $CHCl_3$); ^{1}H NMR (400 MHz, $CDCl_3$) δ 8.07 (d, *J* = 7.4 Hz, 2H), 7.69 (d, *J* = 7.7 Hz, 2H), 7.60 (t, *J* = 7.4 Hz, 1H), 7.46 (t, *J* = 7.7 Hz, 2H), 7.29-7.24 (m, 3H), 7.05 (d, *J* = 9.0 Hz, 2H), 6.84 (d, *J* = 9.0 Hz, 2H), 5.70 (s, 1H), 5.37 (t, *J* = 9.4 Hz, 1H), 5.08 (d, *J* = 6.5 Hz, 1H), 4.96 (d, *J* = 8.9 Hz, 1H), 4.91 (d, *J* = 6.5 Hz, 1H), 4.84 (t, *J* = 9.2 Hz, 1H), 4.07-4.01 (m, 2H), 3.99-3.94 (m, 2H), 3.90-3.85 (m, 1H), 3.78 (s, 3H), 3.56-3.51 (m, 1H), 3.49-3.43 (m, 2H), 3.28 (s, 3H), 2.15 (s, 3H), 1.24 (d, *J* = 6.3 Hz, 3H); ^{13}C NMR (100 MHz, $CDCl_3$) δ 169.5, 165.7, 156.0, 151.2, 136.1, 133.6, 130.0, 129.6, 129.3, 128.8, 128.6, 128.4, 119.2, 114.7, 102.9, 97.8, 91.3, 81.1, 77.9, 76.2, 72.2, 71.8, 70.5, 57.8, 55.8, 48.1, 32.0, 31.7, 21.1, 17.5; MS (ESI) $[M+Na]^+$ 956.9. **23**: $[\alpha]^{28}_{D}$ = +53.4 (*c* = 0.9, $CHCl_3$); ^{1}H NMR (400 MHz, $CDCl_3$) δ 8.08 (d, *J* = 7.5 Hz, 2H), 7.66-7.58 (m, 3H), 7.47 (t, *J* = 7.7 Hz, 2H), 7.30-7.25 (m, 3H), 7.08 (d, *J* = 9.0 Hz, 2H), 6.85 (d, *J* = 9.0 Hz, 2H), 5.62 (t, *J* = 8.0 Hz, 1H), 5.59 (d, *J* = 2.7 Hz, 1H), 5.03 (d, *J* = 8.9 Hz, 1H), 4.90 (t, *J* = 9.1 Hz, 1H), 4.75-4.71 (m, 1H), 4.16-4.11 (m, 1H), 4.07-3.99 (m, 2H), 3.78 (s, 3H), 3.68-3.63 (m, 2H), 3.58-3.50 (m, 2H), 3.26 (s, 3H), 1.98 (s, 3H), 1.23 (d, *J* = 6.2 Hz, 3H); ^{13}C NMR (100 MHz, $CDCl_3$) δ 170.0, 165.5, 156.1, 151.2, 134.5, 133.5, 130.1, 129.8, 129.7, 129.2, 128.6, 127.9, 119.3, 114.7, 103.0, 101.8, 80.2, 78.7, 76.6, 71.6,

70.6, 70.4, 58.2, 55.8, 48.4, 32.8, 31.7, 21.1, 17.4; MS (ESI) $[M+Na]^+$ 927.2.

Disaccharide 9

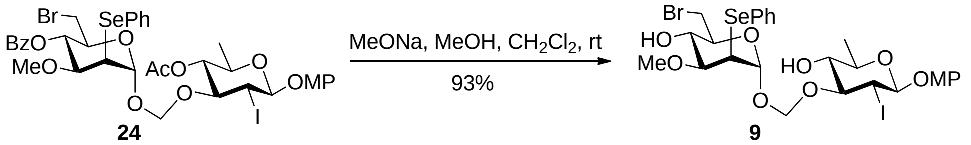

To a solution of **24** (236 mg, 0.253 mmol) in CH_2Cl_2/MeOH (3 mL/3 mL) was added NaOMe (28 mg, 0.506 mmol) at rt. After stirring for 9 h at rt, the mixture was filtered through silica gel. The filtrate was evaporated in vacuo to give a residue, which was purified by flash chromatography (petroleum ether/ EtOAc = 2:1) to afford **9** (185 mg, 93%) as a colorless syrup: $[\alpha]^{30}_D = -7.6$ (c = 1.0, $CHCl_3$); ^{1}H NMR (400 MHz, $CDCl_3$) δ 7.65 (dd, J = 7.4, 1.8 Hz, 2H), 7.31-7.27 (m, 3H), 7.05 (d, J = 9.0 Hz, 2H), 6.84 (d, J = 9.0 Hz, 2H), 5.49 (s, 1H), 5.13 (d, J = 6.5 Hz, 1H), 4.97 (d, J = 6.5 Hz, 1H), 4.92 (d, J = 9.1 Hz, 1H), 3.95 (dd, J = 10.6, 9.2 Hz, 1H), 3.89-3.81 (m, 3H), 3.78 (s, 3H), 3.75-3.70 (m, 2H), 3.61 (dd, J = 11.0, 6.2 Hz, 1H), 3.53 (dd, J = 10.6, 8.4 Hz, 1H), 3.41-3.35 (m, 1H), 3.33 (s, 3H), 3.30-3.25 (m, 1H), 3.15 (d, J = 2.5 Hz, 1H), 2.71 (s, 1H), 1.37 (d, J = 6.0 Hz, 3H); ^{13}C NMR (100 MHz, $CDCl_3$) δ 155.8, 151.4, 135.7, 129.5, 128.7, 128.5, 119.0, 114.7, 102.7, 98.9, 93.8, 86.5, 79.8, 76.2, 72.7, 72.1, 69.9, 56.8, 55.8, 47.1, 33.0, 32.3, 17.7; MS (ESI) $[M+Na]^+$ 811.4.

FABO disaccharide 25

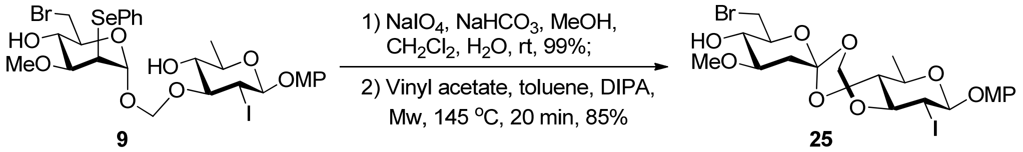

To a solution of **9** (187 mg, 0.237 mmol) in MeOH/CH_2Cl_2/H_2O (3 mL/2 mL/1 mL) were added $NaIO_4$ (507 mg, 2.37 mmol) and $NaHCO_3$ (159 mg, 1.90 mmol) at rt. After stirring for 3 h at rt, the mixture was diluted with CH_2Cl_2, washed with saturated NH_4Cl solution and brine, and was then dried over Na_2SO_4 and concentrated. The resultant selenoxide was azeotroped with toluene (3 x 5 mL) and dried under high vacuum for at least 2 h to afford a white solid (190 mg, 99%). The above selenoxide (125 mg, 0.155 mmol) was dissolved in toluene (6 mL). Diisopropylamine (3 mL) and vinyl acetate (6 mL) were added, and the reaction was conducted under microwave at

145 °C for 20 min. The mixture was cooled to rt and concentrated. The residue was purified by flash chromatography (petroleum ether/EtOAc = 1.5:1) to afford **25** (83 mg, 85%) as a colorless syrup: $[\alpha]^{25}_{D}$ = +34.4 (*c* = 0.8, $CHCl_3$); ^1H NMR (500 MHz, $CDCl_3$) δ 7.08 (d, *J* = 9.1 Hz, 2H), 6.84 (d, *J* = 9.1 Hz, 2H), 5.12 (d, *J* = 8.0 Hz, 1H), 5.01 (d, *J* = 9.1 Hz, 1H), 4.90 (d, *J* = 8.0 Hz, 1H), 4.04 (dd, *J* = 10.9, 9.1 Hz, 1H), 3.99 (t, *J* = 8.9 Hz, 1H), 3.84-3.78 (m, 2H), 3.78 (s, 3H), 3.60-3.56 (m, 1H), 3.52 (dd, *J* = 11.2, 7.9 Hz, 2H), 3.45-3.41 (m, 1H), 3.41 (s, 3H), 3.36-3.31 (m, 1H), 2.66 (brs, 1H), 2.53 (dd, *J* = 12.7, 4.6 Hz, 1H), 1.59 (t, *J* = 12.1 Hz, 1H), 1.46 (d, *J* = 6.2 Hz, 3H); ^{13}C NMR (125 MHz, $CDCl_3$) δ 156.0, 151.4, 119.2, 114.7, 114.7, 103.4, 88.9, 84.2, 78.9, 76.7, 74.0, 72.4, 70.7, 56.9, 55.8, 37.1, 32.4, 29.7, 19.1; HRMS (ESI) calcd for $C_{21}H_{28}BrIO_9Na$ $[M+Na]^+$ 652.9854, found 652.9863.

Preparation of pregnane diol 7 and its derivative S14 for X-ray diffraction analysis

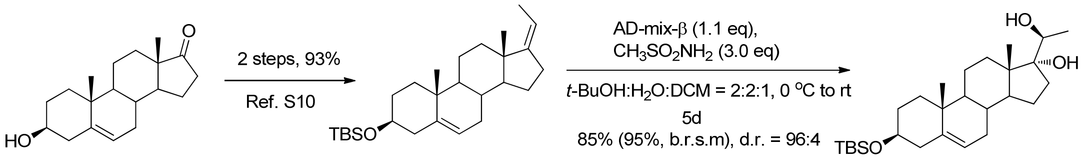

hydroxyandrost-5-en-17-one **S13** **7**

To a stirred solution of AD-mix-β[12] (18.6 g) in *t*-BuOH/H_2O (80 mL/80 mL) were added methanesulfonamide $CH_3SO_2NH_2$ (3.44 g, 36.2 mmol) and a solution of **S13**[13] (5.0 g, 12.1 mmol) in CH_2Cl_2 (40 mL) at 0 °C. The solution was kept at 0 °C for 2 days and then kept at rt for another 3 days. Solid Na_2SO_3 was added, and the solution was stirred for 1 h. The solution was extracted with EtOAc. The resulting organic layer was washed with 2 N KOH and brine, respectively, and was then dried over Na_2SO_4 and concentrated. The residue was purified by flash chromatography (CH_2Cl_2/ EtOAc = 10:1) to afford **7** (4.57 g, 95% based on 85% conversion, d.r. = 96:4) as a white solid and recovered **S13** (0.53 g, 10%). **7**: $[\alpha]^{27}_{D}$ = –58.8 (*c* = 1.0, $CHCl_3$); ^1H NMR (400 MHz, $CDCl_3$) δ 5.31 (brs, 1H), 3.84-3.81 (m, 1H), 3.52-3.44 (m, 1H), 2.29-2.22 (m, 1H), 2.19-2.14 (m, 1H), 2.07-1.92 (m, 3H), 1.83-1.77 (m, 3H), 1.73-1.67 (m, 4H), 1.64-1.41 (m, 7H), 1.18 (d, *J* = 6.3 Hz, 3H), 1.07-1.03 (m, 1H), 0.99 (s, 3H), 0.96-0.92 (m, 1H), 0.88 (s, 9H), 0.73 (s, 3H), 0.05 (s, 6H); ^{13}C NMR

(100 MHz, $CDCl_3$) δ 141.6, 121.2, 85.9, 72.7, 72.5, 51.5, 49.8, 45.8, 42.9, 37.8, 37.5, 36.7, 32.2, 32.0, 32.0, 31.2, 26.1, 23.7, 20.6, 19.6, 18.7, 18.4, 14.1, -4.5; HRMS (ESI) calcd for $C_{27}H_{48}O_3SiNa$ $[M+Na]^+$ 471.3265, found 471.3265.

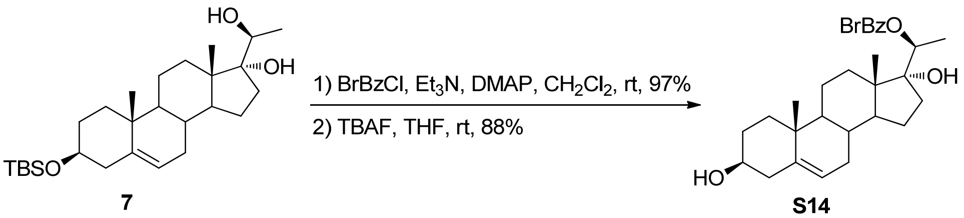

7 **S14**

To a solution of **7** (103 mg, 0.230 mmol) in CH_2Cl_2 (5 mL) were added Et_3N (0.26 mL, 1.84 mmol) and DMAP (2.8 mg, 0.023 mmol). The mixture was cooled to 0 °C, then BrBzCl (202 mg, 0.921 mmol) was added under stirring. After stirring for 10 h at rt, the mixture was quenched with water at 0 ^oC. The mixture was diluted with CH_2Cl_2, washed with saturated $NaHCO_3$ solution and brine, respectively, and was then dried over Na_2SO_4 and concentrated. The residue was purified by flash chromatography (petroleum ether/CH_2Cl_2/EtOAc = 30:1:1) to afford a white solid (141 mg, 97%).

The above residue (60 mg, 0.095 mmol) was dissolved in THF (5 mL). TBAF (1 M in THF, 1.5 mL, 1.5 mmol) was added. After stirring for 22 h at rt, the solution was diluted with CH_2Cl_2, washed with water and brine, respectively, and was then dried over Na_2SO_4 and concentrated. The residue was purified by flash chromatography (petroleum ether/CH_2Cl_2/EtOAc = 3:1:1) to afford **S14** (43 mg, 88%) as a white solid: $[\alpha]^{22}_D$ = –35.0 (c = 1.3, $CHCl_3$); ^{1}H NMR (500 MHz, $CDCl_3$) δ 7.88 (d, J = 8.6 Hz, 2H), 7.58 (d, J = 8.6 Hz, 2H), 5.37-5.33 (m, 2H), 3.53 (ddd, J = 15.8, 11.0, 4.6 Hz, 1H), 2.30 (ddd, J = 13.0, 5.0, 1.9 Hz, 1H), 2.26-2.21 (m, 1H), 2.02-1.96 (m, 2H), 1.87-1.70 (m, 5H), 1.65-1.45 (m, 7H), 1.36 (d, J = 6.4 Hz, 3H), 1.18 (ddd, J = 23.9, 12.0, 5.8 Hz, 1H), 1.12-1.06 (m, 1H), 1.03-0.97 (m, 1H), 1.02 (s, 3H), 0.84 (s, 3H); ^{13}C NMR (125 MHz, $CDCl_3$) δ 165.2, 140.9, 131.9, 131.2, 129.5, 128.2, 121.6, 85.1, 76.9, 71.8, 51.3, 49.8, 46.4, 42.4, 37.9, 37.4, 36.6, 32.0, 31.8, 31.2, 23.6, 20.7, 19.5, 15.8, 14.7; HRMS (ESI) calcd for $C_{56}H_{74}O_8Br_2Na$ $[2M+Na]^+$ 1055.3643, found 1055.3616.

Synthesis of C1''-*epi*-periploside A (31)

FABO disaccharide 26

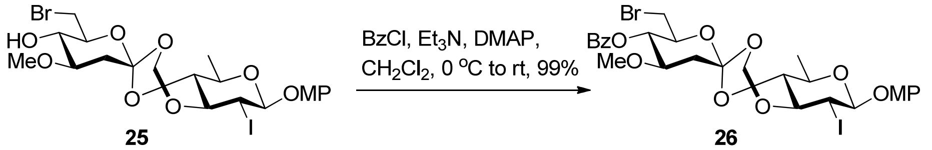

To a solution of **25** (81 mg, 0.128 mmol) in CH_2Cl_2 (3 mL) were added Et_3N (0.11 mL, 0.77 mmol) and DMAP (3 mg, 0.025 mmol). The mixture was cooled to 0 °C, then BzCl (45μL, 0.385 mmol) was added under stirring. The mixture was stirred for 4 h at rt, and then concentrated. The residue was purified by flash chromatography (petroleum ether/EtOAc = 3:1) to afford **26** (94 mg, 99%) as a white solid: $[\alpha]^{29}_{D}$ = +11.7 (*c* = 1.0, $CHCl_3$); ^{1}H NMR (400 MHz, $CDCl_3$) δ 8.05 (d, *J* = 8.4 Hz, 2H), 7.62 (t, *J* = 7.4 Hz, 1H), 7.48 (t, *J* = 7.7 Hz, 2H), 7.10 (d, *J* = 9.0 Hz, 2H), 6.86 (d, *J* = 9.0 Hz, 2H), 5.16 (d, *J* = 8.0 Hz, 1H), 5.10 (t, *J* = 9.5 Hz, 1H), 5.05 (d, *J* = 9.0 Hz, 1H), 4.92 (d, *J* = 8.0 Hz, 1H), 4.11-4.02 (m, 3H), 3.79 (s, 3H), 3.70-3.60 (m, 2H), 3.56 (dd, *J* = 10.9, 8.4 Hz, 1H), 3.52-3.43 (m, 2H), 3.35 (s, 3H), 2.58 (dd, *J* = 12.9, 4.9 Hz, 1H), 1.85 (t, *J* = 12.0 Hz, 1H), 1.53 (d, *J* = 6.2 Hz, 3H); ^{13}C NMR (100 MHz, $CDCl_3$) δ 165.7, 156.0, 151.4, 133.8, 130.0, 129.4, 128.7, 119.3, 114.7, 114.6, 103.4, 89.0, 84.2, 76.9, 76.7, 73.6, 73.1, 70.7, 57.6, 55.8, 38.2, 31.3, 29.6, 19.2; HRMS (ESI) calcd for $C_{28}H_{32}BrIO_{10}Na$ $[M+Na]^+$ 757.0116, found 757.0148.

FABO disaccharide trifluoroacetimidate 27

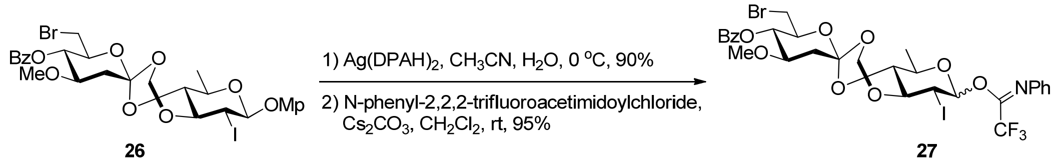

To a solution of **26** (44 mg, 0.0598 mmol) in CH_3CN/H_2O (2 mL/2 mL) was added $Ag(DPAH)_2$[7] (61 mg, 0.132 mmol) at 0 °C. After stirring for 10 min at this temperature, the mixture was filtered. The filtrate was diluted with CH_2Cl_2, washed with saturated $NaHCO_3$ solution and brine, respectively, and was then dried over Na_2SO_4 and concentrated. The residue was purified by flash chromatography (petroleum ether/EtOAc = 3:1) to yield the corresponding hemiacetal (34 mg, 90%) as a colorless syrup.

To a solution of the above hemiacetal (70 mg, 0.111 mmol) in CH_2Cl_2 (3 mL) were added Cs_2CO_3 (145 mg, 0.444 mmol) and *N*-phenyl-2,2,2-trifluoroacetimidoyl chloride (35 μL, 0.333 mmol)[14] at rt. After stirring for 3 h, the mixture was filtered. The filtrate was evaporated in vacuo to give a residue, which was subjected to chromatography on Davisil™ silica (pH = 7.0, petroleum ether/EtOAc, 5:1) to give **27** (85 mg, 95%) as a colorless syrup. This compound was used directly without further characterization.

Pregnane β-disaccharide 28

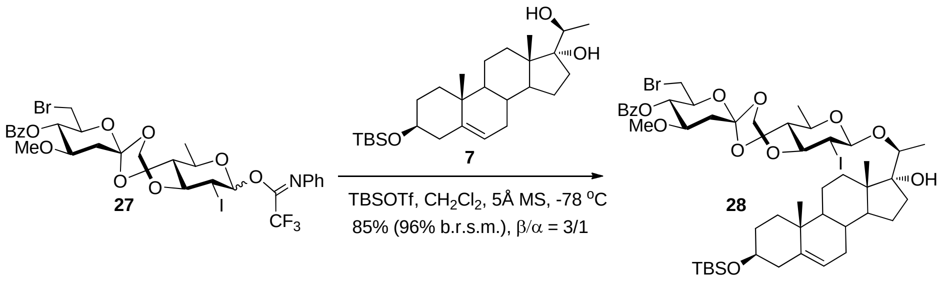

To a solution of **27** (63 mg, 0.079 mmol) and **7** (33 mg, 0.074 mmol) in CH_2Cl_2 (2.5 mL) was added 5Å MS at rt. After stirring for 30 min at –78 °C, TBSOTf (1.7 μL, 0.0074 mmol) was added to the mixture. After stirring for 4 h at this temperature, Et_3N was added to quench the reaction. The resulting mixture was filtered. The filtrate was evaporated in vacuo to give a residue, which was purified by flash chromatography (petroleum ether/EtOAc = 10:1) to afford **28** (50 mg, 64%), its α anomer (16 mg, 21%) as colorless syrups, and recovered **7** (4 mg, 11%). **28**: $[\alpha]^{28}_{D}$ = +8.7 (*c* = 0.5, $CHCl_3$); ^1H NMR (400 MHz, $CDCl_3$) δ 8.05 (d, *J* = 7.5 Hz, 2H), 7.62 (t, *J* = 7.4 Hz, 1H), 7.48 (t, *J* = 7.7 Hz, 2H), 5.32 (brs, 1H), 5.13 (d, *J* = 8.0 Hz, 1H), 5.08 (t, *J* = 9.5 Hz, 1H), 4.89 (d, *J* = 8.0 Hz, 1H), 4.72 (d, *J* = 8.8 Hz, 1H), 4.09-4.04 (m, 1H), 3.95 (t, *J* = 8.8 Hz, 1H), 3.88-3.84 (m, 2H), 3.70-3.63 (m, 1H), 3.53-3.44 (m, 5H), 3.35 (s, 3H), 2.56 (dd, *J* = 13.1, 4.8 Hz, 1H), 2.26 (t, *J* = 12.3 Hz, 1H), 2.18-1.95 (m, 6H), 1.84-1.70 (m, 5H), 1.66-1.53 (m, 6H), 1.48 (d, *J* = 6.2 Hz, 3H), 1.45-1.37 (m, 1H), 1.31 (d, *J* = 6.3 Hz, 3H), 1.22-1.14 (m, 1H), 1.08-0.96 (m, 1H), 1.00 (s, 3H), 0.89 (s, 9H), 0.75 (s, 3H), 0.06 (s, 6H); ^{13}C NMR (100 MHz, $CDCl_3$) δ 165.7, 141.6, 133.8, 130.0, 129.4, 128.7, 121.3, 114.5, 103.0, 89.0, 85.7, 84.3, 83.1, 77.1, 76.6, 73.5,

73.0, 72.7, 70.3, 57.6, 51.0, 49.8, 46.0, 43.0, 39.7, 38.2, 37.5, 36.7, 32.2, 32.1, 32.0, 31.3, 31.2, 30.8, 26.1, 24.0, 22.9, 20.7, 19.6, 19.2, 18.4, 17.0, 14.2, -4.4; HRMS (ESI) calcd for $C_{48}H_{72}O_{11}BrISiNa$ [M+Na]$^+$ 1081.2964, found 1081.2992. **28α:** $[\alpha]^{26}_{D}$ = +43.6 (*c* = 0.75, $CHCl_3$); ^{1}H NMR (400 MHz, $CDCl_3$) δ 8.04 (d, *J* = 7.7 Hz, 2H), 7.61 (t, *J* = 7.4 Hz, 1H), 7.47 (t, *J* = 7.7 Hz, 2H), 5.33 (brs, *J* = 4.1 Hz, 1H), 5.12 (d, *J* = 8.0 Hz, 1H), 5.09 (t, *J* = 9.5 Hz, 1H), 5.04 (d, *J* = 3.5 Hz, 1H), 4.91 (d, *J* = 8.0 Hz, 1H), 4.12-4.07 (m, 1H), 4.00 (dt, *J* = 11.6, 4.8 Hz, 2H), 3.95-3.85 (m, 2H), 3.73-3.63 (m, 2H), 3.53-3.43 (m, 3H), 3.36 (s, 3H), 2.59 (dd, *J* = 12.9, 4.8 Hz, 1H), 2.27 (t, *J* = 12.1 Hz, 1H), 2.22-2.12 (m, 2H), 2.03-1.93 (m, 2H), 1.92-1.69 (m, 7H), 1.67-1.46 m, 7H), 1.43 (d, *J* = 6.1 Hz, 3H), 1.29 (d, *J* = 6.1 Hz, 3H), 1.21-1.18 (m, 1H), 1.10-0.94 (m, 2H), 1.01 (s, 3H), 0.89 (s, 9H), 0.76 (s, 3H), 0.06 (s, 6H); ^{13}C NMR (100 MHz, $CDCl_3$) δ 165.6, 141.8, 133.7, 130.0, 129.4, 128.7, 121.1, 114.5, 94.0, 89.0, 85.5, 80.1, 77.8, 77.4, 76.6, 73.6, 72.9, 72.7, 66.6, 57.6, 51.3, 49.8, 45.9, 43.0, 39.3, 38.2, 37.5, 36.7, 32.2, 32.1, 32.1, 31.4, 31.3, 28.3, 26.1, 23.9, 20.7, 19.6, 19.0, 18.4, 14.1, 13.1, -4.4; HRMS (ESI) calcd for $C_{48}H_{73}BrIO_{11}Si$ [M+H]$^+$ 1059.3145, found 1059.3134.

Pregnane disaccharide S15

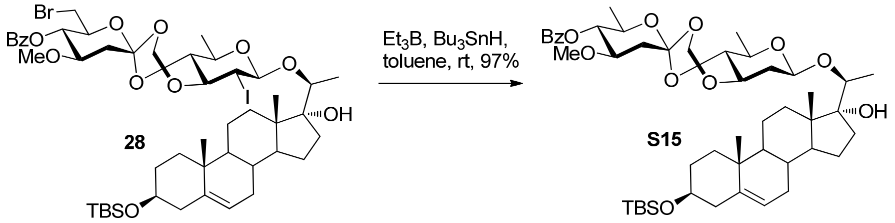

To a solution of **28** (49 mg, 0.046 mmol) in toluene (2 mL) were added Bu_3SnH (75 μL, 0.28 mmol) and Et_3B (9 μL, 0.009 mmol) at 0 °C. After stirring for 1 h at rt, the mixture was concentrated in vacuo to give a residue, which was purified by flash chromatography (petroleum ether/EtOAc = 4:1) to afford **S15** (38 mg, 97%) as a white solid: $[\alpha]^{27}_{D}$ = –10.2 (*c* = 0.9, $CHCl_3$); ^{1}H NMR (400 MHz, $CDCl_3$) δ 8.05 (d, *J* = 7.3 Hz, 2H), 7.59 (t, *J* = 7.4 Hz, 1H), 7.46 (t, *J* = 7.7 Hz, 2H), 5.32 (brs, 1H), 5.03 (t, *J* = 9.5 Hz, 1H), 4.99 (d, *J* = 7.8 Hz, 1H), 4.86 (d, *J* = 7.9 Hz, 1H), 4.65 (d, *J* = 8.6 Hz, 1H), 3.95 (dq, *J* = 12.5, 6.2 Hz, 1H), 3.79-3.71 (m, 2H), 3.65-3.59 (m, 1H), 3.52-3.37 (m, 3H), 3.34 (s, 3H), 2.53 (dd, *J* = 12.8, 4.9 Hz, 1H), 2.26-2.14 (m, 3H), 2.00-1.91

(m, 3H), 1.83-1.63 (m, 9H), 1.54-1.46 (m, 4H), 1.42 (d, *J* = 6.0 Hz, 3H), 1.31 (d, *J* = 6.3 Hz, 3H), 1.26 (d, *J* = 4.0 Hz, 3H), 1.15 (dd, *J* = 12.0, 5.3 Hz, 1H), 1.00 (s, 3H), 0.96-0.86 (m, 3H), 0.89 (s, 9H), 0.74 (s, 3H), 0.06 (s, 6H); ^{13}C NMR (100 MHz, $CDCl_3$) δ 165.8, 141.6, 133.4, 130.0, 129.9, 128.6, 121.2, 114.5, 101.1, 88.7, 85.6, 83.2, 78.0, 77.0, 76.1, 72.7, 70.2, 69.1, 57.4, 51.2, 49.8, 45.5, 43.0, 38.6, 38.5, 37.5, 37.2, 36.7, 32.2, 32.1, 32.0, 31.1, 26.1, 23.6, 20.7, 19.6, 19.2, 18.4, 17.5, 17.2, 16.6, 14.3, -4.4; HRMS (ESI) calcd for $C_{48}H_{74}O_{11}SiNa$ $[M+Na]^+$ 877.4893, found 877.4884.

Pregnane disaccharide 29

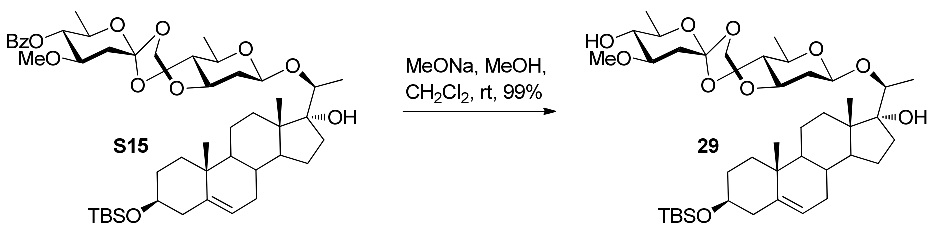

To a solution of **S15** (38 mg, 0.044 mmol) in CH_2Cl_2/MeOH (1.5 mL/1.5 mL) was added NaOMe (24 mg, 0.44 mmol) at rt. After stirring for 18 h at rt, the mixture was filtered through silica gel. The filtrate was evaporated in vacuo to give a residue, which was purified by flash chromatography (petroleum ether/EtOAc = 2:1) to afford **29** (33 mg, 99%) as a white solid: $[\alpha]^{25}_D = -17.0$ (*c* = 0.5, $CHCl_3$); ^{1}H NMR (400 MHz, $CDCl_3$) δ 5.31 (brs, 1H), 4.95 (d, *J* = 7.8 Hz, 1H), 4.84 (d, *J* = 7.9 Hz, 1H), 4.62 (d, *J* = 9.5 Hz, 1H), 3.77-3.68 (m, 3H), 3.52-3.44 (m, 1H), 3.41 (s, 3H), 3.39-3.21 (m, 4H), 2.51-2.46 (m, 2H), 2.26-2.14 (m, 3H), 2.02-1.89 (m, 4H), 1.82-1.62 (m, 7H), 1.57-1.44 (m, 5H), 1.36 (d, *J* = 6.2 Hz, 3H), 1.34 (d, *J* = 5.9 Hz, 3H), 1.30 (d, *J* = 6.3 Hz, 3H), 1.17-1.07 (m, 2H), 1.05-0.93 (m, 2H), 0.99 (s, 3H), 0.89 (s, 9H), 0.72 (s, 3H), 0.06 (s, 6H); ^{13}C NMR (100 MHz, $CDCl_3$) δ 141.6, 121.2, 114.4, 101.0, 88.6, 85.6, 83.2, 79.3, 78.0, 77.3, 75.3, 72.7, 70.6, 70.3, 56.7, 51.2, 49.8, 45.5, 43.0, 38.5, 37.5, 37.4, 37.2, 36.7, 32.2, 32.1, 32.0, 31.1, 26.1, 23.6, 22.9, 20.7, 19.6, 19.2, 18.4, 17.7, 17.1, 14.3, -4.4; HRMS (ESI) calcd for $C_{41}H_{70}O_{10}SiNa$ $[M+Na]^+$ 773.4631, found 773.4633.

Pregnane hexasaccharide 30

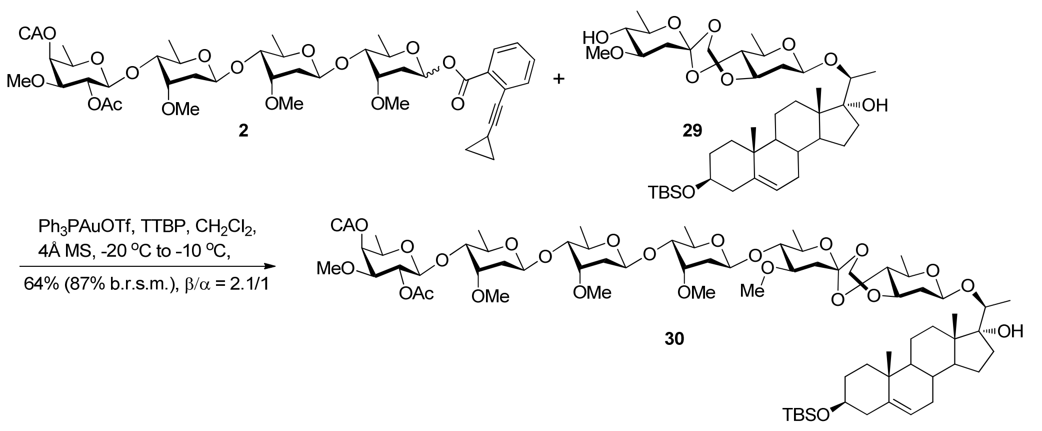

To a solution of **2** (19.0 mg, 0.0212 mmol), **29** (13.0 mg, 0.0173 mmol), and 2,4,6-tri-*tert*-butylpyrimidine (TTBP) (6.5 mg, 0.026 mmol) in CH_2Cl_2 (2.5 mL) was added 4 Å MS at rt. After stirring for 30 min at –20 °C, a solution of PPh_3AuOTf in CH_2Cl_2 (0.10 mL, 0.1 M) was added to the mixture. The mixture was stirred for 1.5 h while warming to –10 °C, then another portion of PPh_3AuOTf in CH_2Cl_2 (33 μL, 0.1 M) was added to the reaction mixture. After stirring for 1.5 h at –10 °C, Et_3N was added to quench the reaction. The resulting mixture was filtered and concentrated. The residue was purified by flash chromatography (petroleum ether/EtOAc = 1:1) to afford **30** (10.9 mg, 43%), its α anomer (5.2 mg, 21%) as white foams, and recovered **29** (3.5 mg, 27%). **30**: $[\alpha]^{22}_{D}$ = +20.8 (*c* = 0.97, $CHCl_3$); ^{1}H NMR (400 MHz, $CDCl_3$) δ 5.36 (d, *J* = 2.7 Hz, 1H), 5.30 (brs, 1H), 5.07 (t, *J* = 8.5 Hz, 1H), 4.93 (d, *J* = 7.5 Hz, 1H), 4.91 (d, *J* = 7.8 Hz, 1H), 4.81 (d, *J* = 7.9 Hz, 1H), 4.76 (d, *J* = 9.5 Hz, 1H), 4.73 (d, *J* = 9.6 Hz, 1H), 4.60 (d, *J* = 9.5 Hz, 1H), 4.43 (d, *J* = 8.0 Hz, 1H), 4.19 (s, 2H), 3.90-3.65 (m, 10H), 3.55-3.45 (m, 3H), 3.40 (s, 12H), 3.34 (s, 6H), 3.33-3.24 (m, 3H), 3.23-3.13 (m, 3H), 2.37 (dd, *J* = 13.1, 4.7 Hz, 1H), 2.29-2.09 (m, 6H), 2.06 (s, 3H), 2.02-1.89 (m, 3H), 1.84-1.67 (m, 7H), 1.63-1.37 (m, 10H), 1.33 (d, *J* = 5.9 Hz, 3H), 1.29 (d, *J* = 5.8 Hz, 6H), 1.24 (d, *J* = 6.0 Hz, 3H), 1.21 (d, *J* = 6.8 Hz, 3H), 1.19 (d, *J* = 6.9 Hz, 3H), 1.16 (d, *J* = 6.1 Hz, 3H), 1.07- 1.00 (m, 2H), 0.99 (s, 3H), 0.88 (s, 9H), 0.72 (s, 3H), 0.05 (s, 6H); ^{13}C NMR (100 MHz, $CDCl_3$) δ 169.4, 167.5, 141.6, 121.2, 114.4, 102.6, 101.0, 99.8, 99.8, 98.8, 88.5, 85.6, 84.1, 83.1, 82.7, 82.5, 82.4, 80.1, 77.9, 77.6, 77.4, 76.4, 72.7, 70.8, 70.6, 70.3, 70.0, 69.1, 69.0, 68.5, 68.1, 58.3, 58.1, 58.1, 58.0, 57.3, 51.2, 49.8, 45.5, 43.0, 40.9, 38.5, 38.1, 37.5, 37.2, 36.7, 35.7, 35.5,

35.3, 32.2, 32.1, 32.1, 32.0, 31.1, 26.1, 23.6, 21.0, 20.7, 19.6, 19.1, 18.4, 18.3, 18.1, 18.1, 17.1, 16.7, 14.3, -4.4; HRMS (ESI) calcd for $C_{73}H_{121}O_{25}ClSiNa$ $[M+Na]^+$ 1483.7547, found 1483.7551.

C1”-*epi*-periploside A (31)

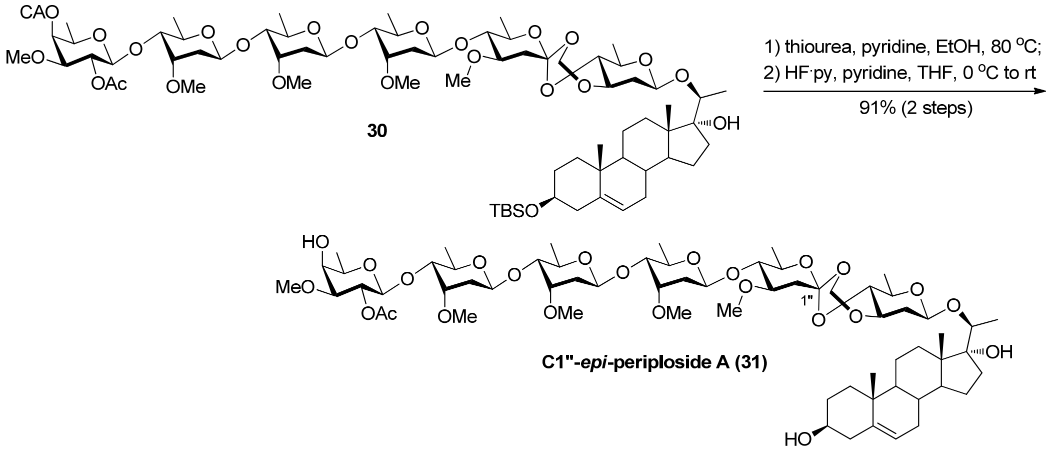

To a solution of **30** (19 mg, 0.013 mmol) in pyridine/EtOH (1.5 mL/1.5 mL) was added thiourea (28 mg, 0.37 mmol) at rt. After stirring for 2 h at 80 °C, the mixture was concentrated in vacuo to give a residue, which was purified by flash chromatography (CH_2Cl_2/MeOH = 20:1) to afford a syrup. The syrup was dissolved in THF/pyridine (3 mL/1.5 mL). HF·py (70% HF in pyridine, 0.18 mL) was added dropwise at 0 °C. After stirring for 34 h at rt, a saturated $NaHCO_3$ solution was added slowly to the mixture. The resulting mixture was diluted with CH_2Cl_2, washed with a saturated $NaHCO_3$ solution, and was then extracted with CH_2Cl_2 twice. The combined organic layer was washed with brine, dried over Na_2SO_4, and concentrated. The residue was purified by flash chromatography (CH_2Cl_2/MeOH = 20:1) to afford C1”-*epi*-periploside A (**31**) (15 mg, 91%) as a white foam: $[\alpha]^{24}_D$ = +55.2 (c = 0.27, MeOH); ^{1}H NMR (500 MHz, $CDCl_3$) δ 5.35 (brs, 1H), 5.07 (t, J = 8.7 Hz, 1H), 4.93 (d, J = 7.7 Hz, 1H), 4.91 (d, J = 9.8 Hz, 1H), 4.82 (d, J = 7.7 Hz, 1H), 4.76 (d, J = 10.7 Hz, 1H), 4.74 (d, J = 11.0 Hz, 1H), 4.60 (d, J = 9.5 Hz, 1H), 4.38 (d, J = 7.8 Hz, 1H), 3.86-3.64 (m, 9H), 3.61-3.48 (m, 3H), 3.44 (s, 3H), 3.42 (s, 6H), 3.41 (s, 6H), 3.40-3.31 (m, 3H), 3.30-3.26 (m, 2H), 3.20-3.17 (m, 3H), 2.37 (d, J = 10.8 Hz, 1H), 2.30-2.18 (m, 4H), 2.13-2.08 (m, 2H), 2.07 (s, 3H), 2.02-1.90 (m, 3H), 1.84-1.65 (m, 7H), 1.64-1.39 (m, 9H), 1.37 (d, J = 6.0 Hz, 3H), 1.33 (d, J = 4.4 Hz, 3H), 1.29 (d, J =

6.4 Hz, 6H), 1.21 (d, J = 7.7 Hz, 3H), 1.19 (d, J = 9.3 Hz, 3H), 1.17 (d, J = 5.5 Hz, 3H), 1.14-1.03 (m, 2H), 1.00 (s, 3H), 0.99-0.95 (m, 1H), 0.72 (s, 3H); ^{13}C NMR (125 MHz, $CDCl_3$) δ 169.4, 140.7, 121.6, 114.2, 102.5, 100.8, 99.7, 99.7, 98.7, 88.4, 85.4, 83.6, 82.9, 82.4, 82.4, 82.2, 81.5, 81.5, 77.7, 77.4, 77.2, 76.9, 76.5, 71.7, 70.8, 70.3, 70.1, 69.9, 68.9, 68.4, 68.1, 67.9, 58.6, 57.9, 57.9, 57.4, 57.2, 51.0, 49.6, 45.3, 42.3, 38.4, 37.9, 37.2, 37.1, 36.5, 35.9, 35.3, 35.2, 31.9, 31.8, 31.6, 30.9, 23.5, 21.0, 20.6, 19.4, 18.9, 18.2, 18.2, 18.0, 17.9, 17.0, 16.5, 14.1; HRMS (ESI) calcd for $C_{65}H_{106}O_{24}Na$ $[M+Na]^+$ 1293.6966, found 1293.6961.

Synthesis of FABO disaccharide 37 with the natural configuration.

3-*O*-(*tert*-Butyldimethyl)silyl-6-deoxy-4-*O*-*p*-methoxybenzyl-D-glucal (S17)

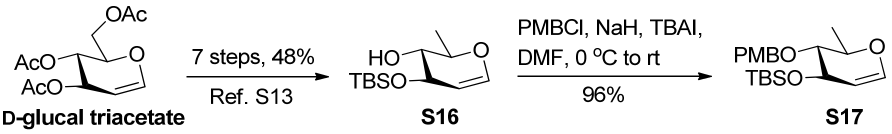

To a solution of **S16**[15] (1.56 g, 6.38 mmol) in DMF (16 mL) were added PMBCl (0.95 mL, 7.02 mmol) and TBAI (118 mg, 0.320 mmol). The mixture was cooled to 0 °C, then NaH (60% dispersion in mineral oil, 511 mg, 12.8 mmol) was added under stirring. After stirring for 30 min at 0 °C, the mixture was warmed up to rt and stirred for another 30 min. The mixture was quenched with water at 0 °C and diluted with CH_2Cl_2. The mixture was washed with a saturated NH_4Cl solution and brine, respectively, and was then dried over Na_2SO_4 and concentrated. The residue was purified by flash chromatography (petroleum ether/EtOAc = 50:1) to afford **S17** (2.23 g, 96%) as a colorless syrup: $[\alpha]^{26}_D$ = –22.1 (c = 1.4, $CHCl_3$); ^{1}H NMR (400 MHz, $CDCl_3$) δ 7.27 (d, J = 7.6 Hz, 2H), 6.88 (d, J = 7.5 Hz, 2H), 6.26 (d, J = 5.5 Hz, 1H), 4.81 (d, J = 10.9 Hz, 1H), 4.62 (d, J = 5.1 Hz, 1H), 4.60 (d, J = 11.1 Hz, 1H), 4.35 (d, J = 5.7 Hz, 1H), 3.96-3.87 (m, 1H), 3.80 (s, 3H), 3.32 (t, J = 7.4 Hz, 1H), 1.32 (d, J = 6.1 Hz, 3H), 0.93 (s, 9H), 0.12 (s, 6H); ^{13}C NMR (100 MHz, $CDCl_3$) δ 159.4, 143.6, 130.6, 129.7, 113.9, 104.0, 81.5, 74.3, 74.06, 70.1, 55.4, 26.0, 18.1, 17.6, -4.2, -4.4; HRMS (ESI) calcd for $C_{20}H_{32}O_4SiNa$ $[M+Na]^+$ 387.1962, found 387.1967.

1-*O*-Acetyl 2,6-dideoxy-4-*O*-*p*-methoxybenzyl-2-iodo-D-glucopyranoside (S18)

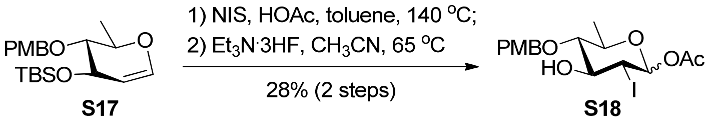

To a solution of **S17** (3.0 g, 8.23 mmol) in toluene (30 mL) were added HOAc (1.40 mL, 24.6 mmol) and NIS (3.7 g, 16.5 mmol) at 140 °C. After stirring for 10 min at this temperature, the mixture was cooled to rt and stirred with a saturated $Na_2S_2O_3$ solution until the mixture turned colorless. The resulting mixture was diluted with EtOAc, washed with saturated $NaHCO_3$ solution twice and then washed with brine. The organic phase was dried over Na_2SO_4 and concentrated. The residue was purified by flash chromatography (petroleum ether/EtOAc = 20:1) to afford a yellow syrup (4.2 g, 93%).

To a solution of the above residue (4.2 g, 7.63 mmol) in MeCN (15 mL) was added 3HF·Et_3N (3.10 mL, 19.1 mmol) at rt. After stirring for 10 h at 65 ^{o}C, a saturated $NaHCO_3$ solution was added slowly to the mixture at rt. The resulting mixture was diluted with EtOAc, washed with saturated $NaHCO_3$ solution, and was then extracted with EtOAc twice. The combined organic layer was washed with brine, dried over Na_2SO_4, and concentrated. The residue was purified by flash chromatography (petroleum ether/EtOAc = 10:1 to 6:1) to afford **S18** (1.0 g, 30%, β/α = 5.9/1, inseparable) as a white solid: $[\alpha]^{28}_{D}$ = +84.9 (*c* = 0.35, $CHCl_3$). **S18β**: ^{1}H NMR (500 MHz, $CDCl_3$) δ 7.27 (d, *J* = 8.9 Hz, 2H), 6.89 (d, *J* = 8.5 Hz, 2H), 5.80 (d, *J* = 9.2 Hz, 1H), 4.73 (d, *J* = 11.0 Hz, 1H), 4.68 (d, *J* = 11.0 Hz, 1H), 3.90-3.86 (m, 1H), 3.84-3.82 (m, 1H), 3.81 (s, 3H), 3.60-3.54 (m, 1H), 3.11 (t, *J* = 8.8 Hz, 1H), 2.54 (d, *J* = 2.8 Hz, 1H), 2.14 (s, 3H), 1.33 (d, *J* = 6.2 Hz, 3H); ^{13}C NMR (125 MHz, $CDCl_3$) δ 169.0, 159.7, 130.0, 130.0, 114.2, 93.9, 83.2, 78.3, 75.1, 72.6, 55.5, 34.4, 21.0, 17.9; HRMS (ESI) calcd for $C_{16}H_{21}IO_6Na$ $[M+Na]^+$ 459.0275, found 459.0274.

1-*O*-Acetyl

2,6-dideoxy-3-*O*-levulinoyl-4-*O*-*p*-methoxybenzyl-2-iodo-D-glucopyranoside (S19)

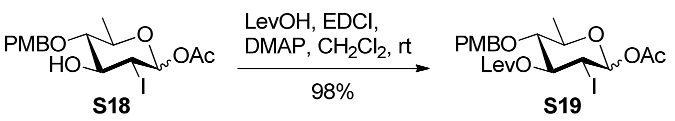

To a solution of **S18** (890 mg, 2.04 mmol) in CH_2Cl_2 (15 mL) were added EDCI (1.2 g, 6.10 mmol), DMAP (501 mg, 4.10 mmol), and LevOH (0.42 mL, 4.10 mmol) at rt. After stirring at rt for 4 h, the mixture was diluted with CH_2Cl_2, washed with brine, dried over Na_2SO_4, and then concentrated. The residue was purified by flash chromatography (petroleum ether/EtOAc = 4:1) to afford **S19** (1.08 g, 98%, β/α = 10/1, inseparable) as a colorless syrup: $[\alpha]^{28}_{D}$ = +62.3 (*c* = 0.17, $CHCl_3$). **S19β**: ^1H NMR (400 MHz, $CDCl_3$) δ 7.19 (d, *J* = 8.6 Hz, 2H), 6.85 (d, *J* = 8.7 Hz, 2H), 5.80 (d, *J* = 9.5 Hz, 1H), 5.32 (dd, *J* = 11.2, 8.9 Hz, 1H), 4.58 (d, *J* = 10.9 Hz, 1H), 4.48 (d, *J* = 10.9 Hz, 1H), 3.90 (dd, *J* = 11.2, 9.5 Hz, 1H), 3.78 (s, 3H), 3.66-3.59 (m, 1H), 3.20 (t, *J* = 9.1 Hz, 1H), 2.84-2.72 (m, 2H), 2.64-2.55 (m, 2H), 2.18 (s, 3H), 2.12 (s, 3H), 1.27 (d, *J* = 6.2 Hz, 3H); ^{13}C NMR (100 MHz, $CDCl_3$) δ 206.2, 171.4, 168.7, 159.6, 129.9, 129.6, 113.9, 93.9, 81.9, 77.1, 74.6, 72.7, 55.4, 37.9, 30.0, 28.3, 27.9, 20.8, 17.8; HRMS (ESI) calcd for $C_{21}H_{27}IO_8Na$ $[M+Na]^+$ 557.0643, found 557.0654.

***p*-Methoxyphenyl 2,6-dideoxy-3-*O*-levulinoyl-2-iodo-β-D-glucopyranoside (32)**

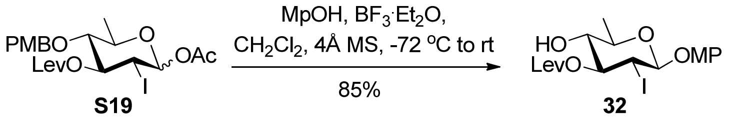

To a solution of **S19** (1.08 g, 2.01 mmol) in CH_2Cl_2 (15 mL) were added MPOH (499 mg, 4.02 mmol) and 4Å MS at rt. After stirring for 30 min at –72 °C, $BF_3 \cdot Et_2O$ (0.62 mL, 5.03 mmol) was added to the mixture. After stirring for another 1 h at this temperature, the mixture was slowly warmed up to rt for 3h, and then quenched with Et_3N and filtered. The resulting mixture was diluted with EtOAc, washed with a saturated $NaHCO_3$ solution and brine, respectively, and was then dried over Na_2SO_4 and concentrated. The residue was purified by flash chromatography (petroleum ether/EtOAc = 1.5:1) to afford **32** (820 mg, 85%) as a colorless syrup: $[\alpha]^{27}_{D}$ = +10.3 (*c* = 1.2, $CHCl_3$); ^1H NMR (400 MHz, $CDCl_3$) δ 7.06 (d, *J* = 9.0 Hz, 2H), 6.83 (d, *J* = 9.0 Hz, 2H), 5.21 (dd, *J* = 11.1, 8.8 Hz, 1H), 5.03 (d, *J* = 9.0 Hz, 1H), 4.05 (dd, *J* = 11.1, 9.0 Hz, 1H), 3.76 (s, 3H), 3.60-3.53 (m, 1H), 3.48 (d, *J* = 3.1 Hz, 1H), 3.42-3.38

(m, 1H), 2.99-2.90 (m, 1H), 2.84-2.77 (m, 1H), 2.69-2.54 (m, 2H), 2.19 (s, 3H), 1.40 (d, J = 6.1 Hz, 3H); ^{13}C NMR (101 MHz, $CDCl_3$) δ 208.3, 172.6, 155.8, 151.3, 119.0, 114.6, 102.6, 78.9, 75.6, 72.3, 55.7, 38.6, 29.9, 29.2, 28.4, 17.7; HRMS (ESI) calcd for $C_{18}H_{23}IO_7Na$ $[M+Na]^+$ 501.0381, found 501.0392.

α-Disaccharide 33

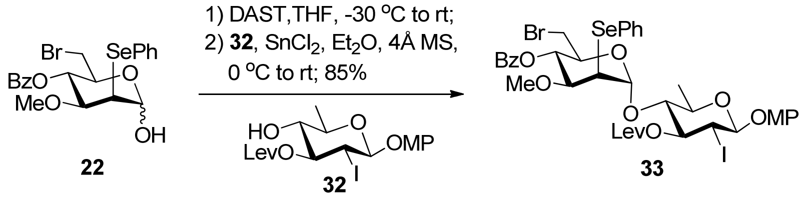

To a solution of **22** (603 mg, 1.21 mmol) in THF (10 mL) was added dimethylaminosulfur trifluoride (DAST) (0.44 mL, 3.63 mmol) at –30 °C. After stirring for 1.5 h while warming to rt, a saturated $NaHCO_3$ solution was added slowly to the mixture. The resulting mixture was extracted with CH_2Cl_2. The combined organic layer was washed with brine, dried over Na_2SO_4, and concentrated. The crude glycosyl fluoride was azeotroped with toluene (3 x 5 mL). After drying under high vacuum for 2 h, the above product was dissolved in Et_2O (10 mL). 4 Å MS (1.2 g) and **32** (330 mg, 0.69 mmol) were added and the reaction mixture was stirred at 0 °C for 30 min. $SnCl_2$ (235 mg, 1.24 mmol) was added in one portion and the reaction mixture was allowed to warm to rt and stirred for 4 h. The mixture was quenched with Et_3N (1 mL) and filtered. The solution was diluted with EtOAc and washed with water. The water layer was extracted with EtOAc twice. The combined organic layer was washed with saturated $NaHCO_3$ solution and brine, respectively, and was then dried over Na_2SO_4 and concentrated. The residue was purified by flash chromatography (petroleum ether/EtOAc = 4:1) to afford **33** (560 mg, 85%) as a colorless syrup: $[\alpha]^{29}_D$ = +27.1 (c = 1.1, $CHCl_3$); ^1H NMR (400 MHz, $CDCl_3$) δ 8.05 (d, J = 7.3 Hz, 2H), 7.67-7.65 (m, 2H), 7.60 (t, J = 7.4 Hz, 1H), 7.46 (t, J = 7.7 Hz, 2H), 7.28-7.27 (m, 3H), 7.06 (d, J = 9.0 Hz, 2H), 6.84 (d, J = 9.0 Hz, 2H), 5.43-5.36 (m, 3H), 5.00 (d, J = 9.0 Hz, 1H), 4.17-4.12 (m, 1H), 4.06 (dd, J = 11.1, 9.1 Hz, 1H), 3.90 (dd, J = 8.1, 4.1 Hz, 1H), 3.78 (s, 3H), 3.62-3.60 (m, 3H), 3.53-3.44 (m, 2H), 3.24 (s, 3H), 2.76-2.69 (m, 2H), 2.61-2.46 (m, 2H), 2.13 (s, 3H), 1.49 (d, J = 5.1 Hz,

3H); ^{13}C NMR (100 MHz, $CDCl_3$) δ 205.7, 171.8, 165.6, 156.0, 151.2, 134.7, 133.6, 130.0, 129.6, 129.5, 129.2, 128.7, 128.0, 119.2, 114.7, 102.7, 102.2, 81.1, 78.2, 77.8, 72.2, 71.6, 71.5, 58.2, 55.8, 48.4, 37.9, 31.8, 29.9, 29.6, 28.6, 18.9; HRMS (ESI) calcd for $C_{38}H_{42}BrIO_{11}SeNa$ $[M+Na]^+$ 983.0014, found 983.0021.

Ketene acetal 34

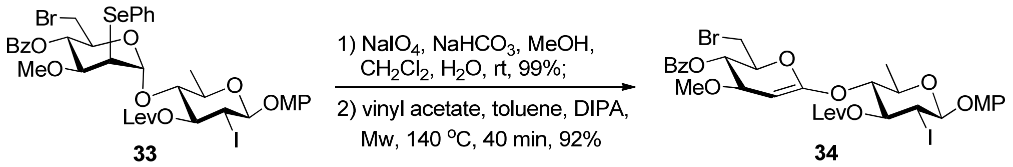

To a solution of **33** (248 mg, 0.258 mmol) in $MeOH/CH_2Cl_2/H_2O$ (3 mL/2 mL/1 mL) were added $NaIO_4$ (552 mg, 2.58 mmol) and $NaHCO_3$ (173 mg, 2.06 mmol) at rt. After stirring for 12 h at rt, the mixture was diluted with CH_2Cl_2, and washed with saturated NH_4Cl solution and brine, respectively. The organic layer was dried over Na_2SO_4 and concentrated. The crude selenoxide was azeotroped with toluene (3 x 5 mL) and dried under high vacuum for 2 h to afford a colorless syrup (251 mg, 99%).

The above selenoxide (104 mg, 0.107 mmol) was dissolved in toluene (2 mL). Diisopropylamine (1 mL) and vinyl acetate (2 mL) were added, and the reaction was conducted under microwave at 140 ºC for 40 min. The mixture was cooled to rt and concentrated. The residue was purified by flash chromatography (petroleum ether/ EtOAc = 5:1, containing 1% Et_3N) to afford **34** (85 mg, 92%) as a colorless syrup: $[\alpha]^{27}_D = +51.7$ (c = 0.88, $CHCl_3$); ^{1}H NMR (500 MHz, $CDCl_3$) δ 8.01 (d, J = 7.3 Hz, 2H), 7.59 (t, J = 7.4 Hz, 1H), 7.45 (t, J = 7.8 Hz, 2H), 7.07 (d, J = 9.0 Hz, 2H), 6.84 (d, J = 9.0 Hz, 2H), 5.46-5.41 (m, 2H), 5.03 (d, J = 9.0 Hz, 1H), 4.70-4.68 (m, 1H), 4.33 (d, J = 4.4 Hz, 1H), 4.23 (t, J = 9.3 Hz, 1H), 4.07 (dd, J = 11.2, 9.1 Hz, 1H), 3.89-3.88 (m, 1H), 3.82-3.79 (m, 1H), 3.78 (s, 3H), 3.69 (dd, J = 9.5, 6.2 Hz, 1H), 3.64 (dd, J = 11.2, 4.7 Hz, 1H), 3.41 (s, 3H), 2.87-2.63 (m, 4H), 2.18 (s, 3H), 1.43 (d, J = 6.1 Hz, 3H); ^{13}C NMR (125 MHz, $CDCl_3$) δ 206.0, 171.5, 165.4, 156.0, 155.8, 151.2, 133.7, 130.0, 129.4, 128.7, 119.1, 114.7, 102.7, 78.8, 77.9, 75.9, 74.9, 73.9, 71.1, 67.8, 56.5, 55.8, 38.0, 30.0, 29.0, 28.8, 28.5, 17.8; HRMS (ESI) calcd for $C_{32}H_{36}BrIO_{11}Na$ $[M+Na]^+$ 825.0378, found 825.0381.

Orthoester 35

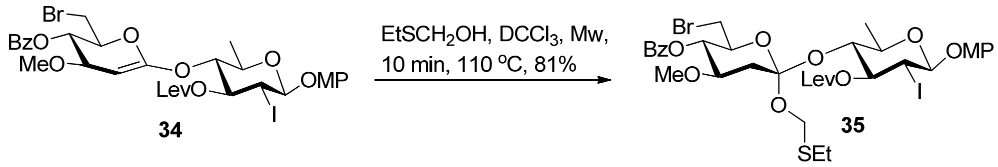

To a solution of **34** (75 mg, 0.093 mmol) in $CDCl_3$ (3 mL) was added $EtSCH_2OH$[16] (0.05 mL) at rt. The reaction was conducted under microwave at 110 ºC for 10 min. The mixture was cooled to rt and concentrated. The residue was purified by flash chromatography (petroleum ether/ EtOAc = 5:1, containing 1% Et_3N) to afford **35** (67 mg, 81%) as a colorless syrup: $[\alpha]^{28}_D$ = +11.9 (*c* = 1.2, $CHCl_3$); ^1H NMR (500 MHz, $CDCl_3$) δ 8.07 (d, *J* = 7.2 Hz, 2H), 7.61 (t, *J* = 7.4 Hz, 1H), 7.48 (t, *J* = 7.7 Hz, 2H), 7.07 (d, *J* = 9.0 Hz, 2H), 6.84 (d, *J* = 9.0 Hz, 2H), 5.30 (dd, *J* = 11.2, 9.1 Hz, 1H), 5.08 (t, *J* = 9.6 Hz, 1H), 5.01 (d, *J* = 9.0 Hz, 1H), 4.85 (d, *J* = 11.3 Hz, 1H), 4.74 (d, *J* = 11.3 Hz, 1H), 4.09-4.02 (m, 2H), 3.81-3.74 (m, 2H), 3.78 (s, 3H), 3.65-3.59 (m, 1H), 3.49 (dd, *J* = 11.3, 2.1 Hz, 1H), 3.41-3.38 (m, 1H), 3.37 (s, 3H), 2.94-2.87 (m, 1H), 2.75-2.66 (m, 5H), 2.48 (dd, *J* = 12.8, 5.0 Hz, 1H), 2.21 (s, 3H), 1.96-1.91 (m, 1H), 1.52 (d, *J* = 6.2 Hz, 3H), 1.33 (t, *J* = 7.4 Hz, 3H); ^{13}C NMR (125 MHz, $CDCl_3$) δ 205.9, 171.3, 165.7, 155.9, 151.3, 133.7, 130.0, 129.5, 128.7, 119.2, 114.7, 113.5, 102.6, 77.3, 76.1, 75.9, 73.3, 73.0, 72.6, 63.9, 58.0, 55.8, 37.8, 37.6, 31.5, 30.2, 30.1, 28.6, 25.9, 19.0, 15.2; HRMS (ESI) calcd for $C_{35}H_{44}BrIO_{12}SNa$ $[M+Na]^+$ 917.0674, found 917.0680.

Orthoester 36

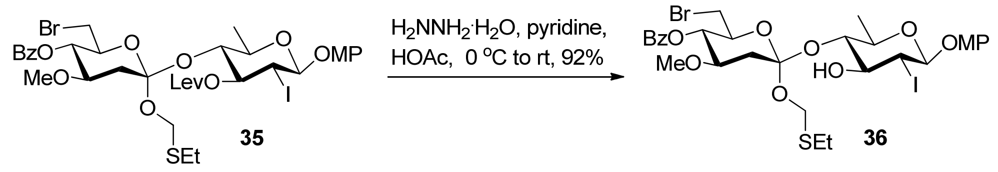

To a solution of **35** (110 mg, 0.120 mmol) in pyridine/HOAc (3 mL/2 mL) was added $H_2NNH_2{\cdot}H_2O$ (0.10 mL, 1.60 mmol) at 0 ºC. After stirring at rt for 5 h, the mixture was diluted with CH_2Cl_2, and washed with ice water, and then with a saturated $NaHCO_3$ solution and brine, respectively. The organic layer was dried over Na_2SO_4 and concentrated. The residue was purified by flash chromatography (petroleum ether/ EtOAc = 5:1, containing 1% Et_3N) to afford **36** (90 mg, 92%) as a colorless syrup:

$[\alpha]^{27}_{D}$ = +33.0 (*c* = 0.46, $CHCl_3$); ^{1}H NMR (400 MHz, $CDCl_3$) δ 8.07-8.05 (m, 2H), 7.61 (t, *J* = 7.4 Hz, 1H), 7.48 (t, *J* = 7.7 Hz, 2H), 7.07 (d, *J* = 9.0 Hz, 2H), 6.84 (d, *J* = 9.0 Hz, 2H), 5.07 (t, *J* = 9.5 Hz, 1H), 5.00 (d, *J* = 9.0 Hz, 1H), 4.94 (d, *J* = 11.6 Hz, 1H), 4.79 (d, *J* = 11.6 Hz, 1H), 4.07 (dd, *J* = 10.6, 9.1 Hz, 1H), 4.00 (ddd, *J* = 9.9, 7.9, 2.2 Hz, 1H), 3.84-3.80 (m, 2H), 3.78 (s, 3H), 3.68 (t, *J* = 8.8 Hz, 1H), 3.59-3.53 (m, 1H), 3.49 (dd, *J* = 11.3, 2.2 Hz, 1H), 3.41 (dd, *J* = 11.3, 7.8 Hz, 1H), 3.34 (s, 3H), 3.20 (d, *J* = 3.3 Hz, 1H), 2.80-2.71 (m, 2H), 2.57 (dd, *J* = 13.3, 5.0 Hz, 1H), 2.13 (dd, *J* = 13.2, 11.5 Hz, 1H), 1.48 (d, *J* = 6.2 Hz, 3H), 1.37 (t, *J* = 7.4 Hz, 3H); ^{13}C NMR (100 MHz, $CDCl_3$) δ 165.7, 155.9, 151.4, 133.7, 130.0, 129.5, 128.7, 119.1, 114.7, 113.9, 102.6, 77.9, 77.3, 77.0, 73.4, 73.3, 71.9, 64.3, 57.7, 55.8, 37.6, 36.4, 31.5, 26.3, 18.6, 15.3; HRMS (ESI) calcd for $C_{30}H_{38}BrIO_{10}SNa$ $[M+Na]^+$ 819.0306, found 819.0286.

FABO disaccharide 37

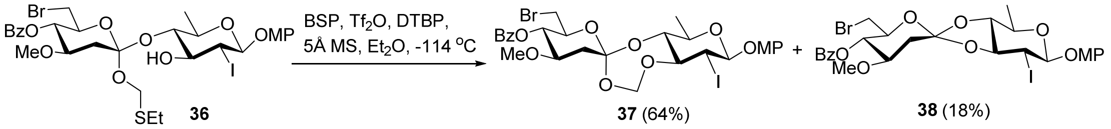

To a solution of **36** (19.1 mg, 0.024 mmol) in Et_2O (3 mL) were added BSP (8.2 mg, 0.036 mmol), 2,6-di-*tert*-butylpyridine (16.0 μL, 0.072 mmol), and 5Å MS at rt. After stirring for 20 min at –114 °C (liq. N_2-EtOH), Tf_2O (6.0 μL, 0.036 mmol) was added to the mixture. The reaction mixture was stirred for 1 h at –114 °C and then warmed to room temperature and filtered. The filtrate was washed with a saturated aqueous $NaHCO_3$ solution and brine, respectively, and was then dried over Na_2SO_4 and concentrated. The residue was purified by flash chromatography (petroleum ether/ EtOAc = 5:1) to afford **37** (11.3 mg, 64%) and a five-membered orthoester **38** (3.0 mg, 18%) as colorless syrups. **37**: $[\alpha]^{27}_{D}$ = +57.5 (*c* = 0.28, $CHCl_3$); ^{1}H NMR (400 MHz, $CDCl_3$) δ 8.08-8.05 (m, 2H), 7.62 (t, *J* = 7.4 Hz, 1H), 7.48 (t, *J* = 7.7 Hz, 2H), 7.08 (d, *J* = 9.1 Hz, 2H), 6.85 (d, *J* = 9.1 Hz, 2H), 5.35 (d, *J* = 7.8 Hz, 1H), 5.09 (t, *J* = 9.6 Hz, 1H), 5.01 (d, *J* = 7.8 Hz, 1H), 5.00 (d, *J* = 9.2 Hz, 1H), 4.03-3.94 (m, 2H), 3.79 (s, 3H), 3.77-3.72 (m, 2H), 3.66-3.57 (m, 2H), 3.49-3.42 (m, 2H), 3.37 (s, 3H), 2.63 (dd, *J* = 12.6, 5.2 Hz, 1H), 1.81-1.75 (m, 1H), 1.41 (d, *J* = 5.7 Hz, 3H); ^{13}C NMR (100

MHz, $CDCl_3$) δ 165.7, 156.0, 151.3, 133.8, 130.0, 129.3, 128.8, 119.2, 114.7, 114.1, 103.0, 87.1, 84.7, 79.08, 77.1, 73.8, 73.5, 70.2, 58.2, 55.8, 36.9, 31.2, 28.9, 17.9; HRMS (ESI) calcd for $C_{28}H_{32}BrIO_{10}Na$ $[M+Na]^+$ 757.0116, found 757.0107. **38**: $[\alpha]^{27}_D$ = +27.8 (*c* = 0.39, $CHCl_3$); ^1H NMR (500 MHz, $CDCl_3$) δ 8.10-8.08 (m, 2H), 7.61 (t, *J* = 7.4 Hz, 1H), 7.49 (t, *J* = 7.8 Hz, 2H), 7.10 (d, *J* = 9.1 Hz, 2H), 6.85 (d, *J* = 9.1 Hz, 2H), 5.12 (t, *J* = 9.6 Hz, 1H), 5.06 (d, *J* = 7.7 Hz, 1H), 4.17-4.07 (m, 3H), 3.93-3.84 (m, 2H), 3.79 (s, 3H), 3.49 (dd, *J* = 11.2, 2.4 Hz, 1H), 3.42 (dd, *J* = 11.2, 8.4 Hz, 1H), 3.38 (s, 3H), 3.37 (t, *J* = 9.0 Hz, 1H), 2.53 (dd, *J* = 13.1, 5.1 Hz, 1H), 2.11 (dd, *J* = 12.9, 11.8 Hz, 1H), 1.43 (d, *J* = 6.1 Hz, 3H); ^{13}C NMR (125 MHz, $CDCl_3$) δ 165.8, 156.1, 151.4, 133.7, 130.1, 129.4, 128.7, 119.7, 119.2, 114.7, 104.1, 82.3, 82.1, 73.9, 73.3, 72.0, 57.8, 55.8, 37.3, 31.5, 24.3, 18.2; HRMS (ESI) calcd for $C_{27}H_{30}BrIO_9Na$ $[M+Na]^+$ 727.0010, found 727.0010.

Completion of the synthesis of periploside A (1)

FABO disaccharide trifluoroacetimidate 6

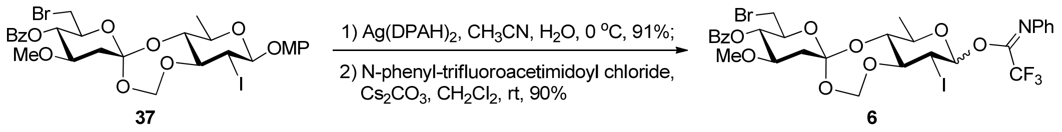

To a solution of **37** (36 mg, 0.0490 mmol) in CH_3CN/H_2O (3 mL/3 mL) was added $Ag(DPAH)_2$[7] (79 mg, 0.172 mmol) at 0 °C. After stirring for 30 min at this temperature, the mixture was filtered. The filtrate was diluted with CH_2Cl_2, washed with saturated $NaHCO_3$ solution and brine, respectively, and was then dried over Na_2SO_4 and concentrated. The residue was purified by flash chromatography (petroleum ether/EtOAc = 3:1) to yield the corresponding hemiacetal (28 mg, 91%) as a colorless syrup.

To a solution of the above hemiacetal (29 mg, 0.046 mmol) in CH_2Cl_2 (3 mL) were added Cs_2CO_3 (75 mg, 0.23 mmol) and *N*-phenyl-2,2,2-trifluoroacetimidoyl chloride (14 μL, 0.138 mmol)[14] at rt. After stirring for 3 h, the mixture was filtered. The filtrate was evaporated in vacuo to give a residue, which was subjected to chromatography on Davisil™ silica (pH = 7.0, petroleum ether/EtOAc, 5:1) to give **6**

(33 mg, 90%) as a colorless syrup. This compound was used directly without further characterization.

Pregnane β-disaccharide 39

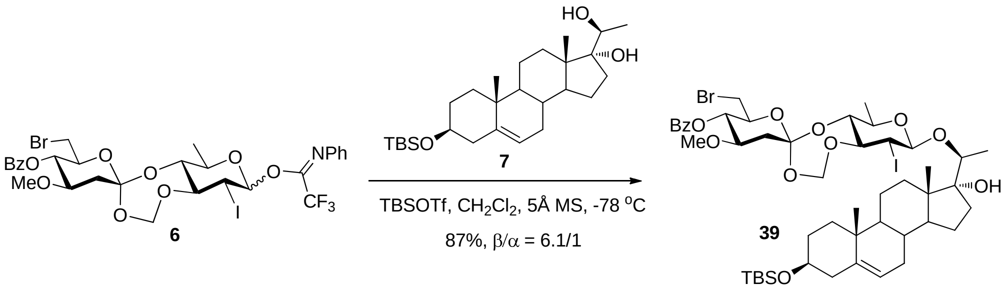

To a solution of **6** (33.0 mg, 0.0413 mmol) and **7** (15.6 mg, 0.0345 mmol) in CH_2Cl_2 (3 mL) was added 5Å MS at rt. After stirring for 30 min at –78 °C, TBSOTf (1.2 μL, 0.0052 mmol) was added to the mixture. After stirring for 6 h at this temperature, Et_3N was added to quench the reaction. The resulting mixture was filtered. The filtrate was evaporated in vacuo to give a residue, which was purified by flash chromatography (petroleum ether/CH_2Cl_2/EtOAc = 10:5:1) to afford **39** (27.6 mg, 75%) and its α anomer (4.5 mg, 12%) as white solids. **39**: $[\alpha]^{23}_D$ = +26.2 (*c* = 1.1, $CHCl_3$); ^{1}H NMR (500 MHz, $CDCl_3$) δ 8.06 (d, *J* = 7.3 Hz, 2H), 7.61 (t, *J* = 7.4 Hz, 1H), 7.48 (t, *J* = 7.8 Hz, 2H), 5.32 (brs, 1H), 5.31 (d, *J* = 7.8 Hz, 1H), 5.08 (t, *J* = 9.6 Hz, 1H), 4.97 (d, *J* = 7.8 Hz, 1H), 4.69 (d, *J* = 8.8 Hz, 1H), 3.97-3.93 (m, 1H), 3.86 (q, *J* = 6.3 Hz, 1H), 3.80 (dd, *J* = 10.7, 8.9 Hz, 1H), 3.74 (ddd, *J* = 11.5, 9.1, 5.0 Hz, 1H), 3.66 (dd, *J* = 10.8, 7.3 Hz, 1H), 3.56-3.52 (m, 2H), 3.50-3.40 (m, 3H), 3.36 (s, 3H), 2.59 (dd, *J* = 12.6, 5.1 Hz, 1H), 2.26 (t, *J* = 11.1 Hz, 1H), 2.18-1.97 (m, 6H), 1.83-1.70 (m, 5H), 1.63-1.49 (m, 6H), 1.37 (d, *J* = 3.8 Hz, 3H), 1.30 (d, *J* = 6.6 Hz, 3H), 1.05 (dd, *J* = 13.7, 3.4 Hz, 1H), 1.00 (s, 3H), 0.98-0.95 (m, 1H), 0.89 (s, 9H), 0.75 (s, 3H), 0.06 (s, 6H); ^{13}C NMR (125 MHz, $CDCl_3$) δ 165.7, 141.6, 133.8, 130.0, 129.3, 128.7, 121.2, 114.0, 102.6, 87.1, 85.7, 84.7, 83.0, 79.2, 77.1, 73.8, 73.5, 72.7, 69.8, 58.2, 51.1, 49.8, 46.0, 43.0, 39.8, 37.5, 36.9, 36.7, 32.2, 32.1, 32.0, 31.2, 31.2, 30.1, 26.1, 24.0, 22.8, 20.7, 19.6, 18.4, 17.9, 17.2, 14.1, -4.4; HRMS (ESI) calcd for $C_{48}H_{73}BrIO_{11}Si$ $[M+H]^+$ 1059.3145, found 1059.3142.

Pregnane disaccharide S20

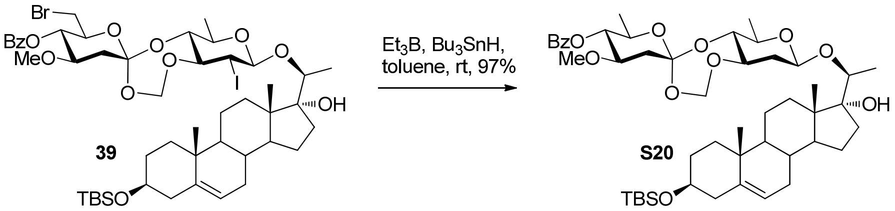

To a solution of **39** (21.0 mg, 0.0199 mmol) in toluene (2 mL) were added Bu_3SnH (32 μL, 0.119 mmol) and Et_3B (12 μL, 0.0116 mmol) at 0 °C. After stirring for 1 h at rt, the mixture was concentrated in vacuo. The residue was purified by flash chromatography (petroleum ether/EtOAc = 5:1) to afford **S20** (16.5 mg, 97%) as a white solid: $[\alpha]^{21}_{D}$ = –14.0 (*c* = 0.81, $CHCl_3$); ^{1}H NMR (500 MHz, $CDCl_3$) δ 8.06 (d, *J* = 7.1 Hz, 2H), 7.58 (t, *J* = 7.4 Hz, 1H), 7.46 (t, *J* = 7.8 Hz, 2H), 5.31 (brs, 1H), 5.21 (d, *J* = 7.6 Hz, 1H), 5.01 (t, *J* = 9.5 Hz, 1H), 4.85 (d, *J* = 7.6 Hz, 1H), 4.61 (dd, *J* = 9.7, 1.7 Hz, 1H), 3.86-3.80 (m, 1H), 3.75 (q, *J* = 6.3 Hz, 1H), 3.69 (ddd, *J* = 11.6, 9.3, 5.0 Hz, 1H), 3.58-3.53 (m, 1H), 3.51-3.37 (m, 3H), 3.34 (s, 3H), 2.59 (dd, *J* = 12.5, 5.1 Hz, 1H), 2.29-2.22 (m, 2H), 2.16 (ddd, *J* = 13.5, 4.8, 2.0 Hz, 1H), 1.99-1.91 (m, 3H), 1.82-1.63 (m, 8H), 1.58-1.46 (m, 4H), 1.41 (dd, *J* = 13.0, 3.5 Hz, 1H), 1.36 (d, *J* = 5.8 Hz, 3H), 1.30 (d, *J* = 6.3 Hz, 3H), 1.26 (d, *J* = 6.2 Hz, 3H), 1.15 (dd, *J* = 12.0, 5.7 Hz, 1H), 1.06-0.94 (m, 2H), 1.00 (s, 3H), 0.88 (s, 9H), 0.73 (s, 3H), 0.05 (s, 6H); ^{13}C NMR (125 MHz, $CDCl_3$) δ 165.8, 141.6, 133.4, 130.0, 129.9, 128.6, 121.2, 114.0, 101.0, 86.7, 85.6, 83.3, 79.6, 78.5, 77.1, 76.4, 72.7, 69.8, 69.2, 57.9, 51.2, 49.8, 45.5, 43.0, 38.5, 37.5, 37.0, 37.0, 36.7, 32.2, 32.1, 32.0, 31.1, 26.1, 23.6, 20.7, 19.6, 18.4, 18.2, 17.8, 17.2, 14.3, -4.4; HRMS (ESI) calcd for $C_{48}H_{75}O_{11}Si$ $[M+H]^+$ 855.5073, found 855.5070.

Pregnane disaccharide 3

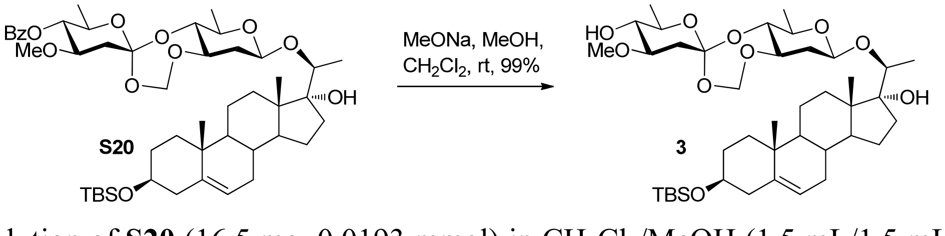

To a solution of **S20** (16.5 mg, 0.0193 mmol) in CH_2Cl_2/MeOH (1.5 mL/1.5 mL) was added NaOMe (20 mg, 0.37 mmol) at rt. After stirring for 40 h, the mixture was filtered through silica gel. The filtrate was evaporated in vacuo to give a residue, which was purified by flash chromatography (petroleum ether/ EtOAc = 2:1) to afford

3 (14.0 mg, 97%) as a white solid: $[\alpha]^{22}_D$ = –25.6 (*c* = 0.56, $CHCl_3$); ^{1}H NMR (500 MHz, $CDCl_3$) δ 5.31 (brs, 1H), 5.17 (d, *J* = 7.6 Hz, 1H), 4.79 (d, *J* = 7.6 Hz, 1H), 4.59 (dd, *J* = 9.8, 1.8 Hz, 1H), 3.74 (q, *J* = 6.3 Hz, 1H), 3.62-3.58 (m, 1H), 3.56-3.46 (m, 2H), 3.42-3.34 (m, 3H), 3.41 (s, 3H), 3.21 (t, *J* = 9.2 Hz, 1H), 2.54 (dd, *J* = 12.2, 4.8 Hz, 1H), 2.48 (brs, 1H), 2.28-2.20 (m, 2H), 2.16 (ddd, *J* = 13.5, 4.9, 2.0 Hz, 1H), 2.01-1.90 (m, 3H), 1.82-1.78 (m, 2H), 1.75-1.68 (m, 3H), 1.66-1.46 (m, 8H), 1.40 (dd, *J* = 13.0, 3.7 Hz, 1H), 1.34 (d, *J* = 3.8 Hz, 3H), 1.33 (d, *J* = 3.4 Hz, 3H), 1.29 (d, *J* = 6.3 Hz, 3H), 1.14 (dd, *J* = 12.0, 5.7 Hz, 1H), 1.04 (dd, *J* = 13.8, 3.7 Hz, 1H), 1.00-0.95 (m, 1H), 0.99 (s, 3H), 0.88 (s, 9H), 0.72 (s, 3H), 0.05 (s, 6H); ^{13}C NMR (125 MHz, $CDCl_3$) δ 141.6, 121.2, 114.0, 100.9, 86.5, 85.6, 83.3, 79.4, 79.4, 78.4, 75.8, 72.7, 70.7, 69.8, 57.0, 51.2, 49.8, 45.5, 43.0, 38.5, 37.5, 37.0, 36.7, 35.8, 32.2, 32.1, 32.0, 31.1, 26.1, 23.6, 20.7, 19.6, 18.4, 18.2, 18.0, 17.2, 14.3, -4.4; HRMS (ESI) calcd for $C_{41}H_{70}O_{10}SiNa$ $[M+Na]^+$ 773.4630, found 773.4625.

Pregnane hexasaccharide 40

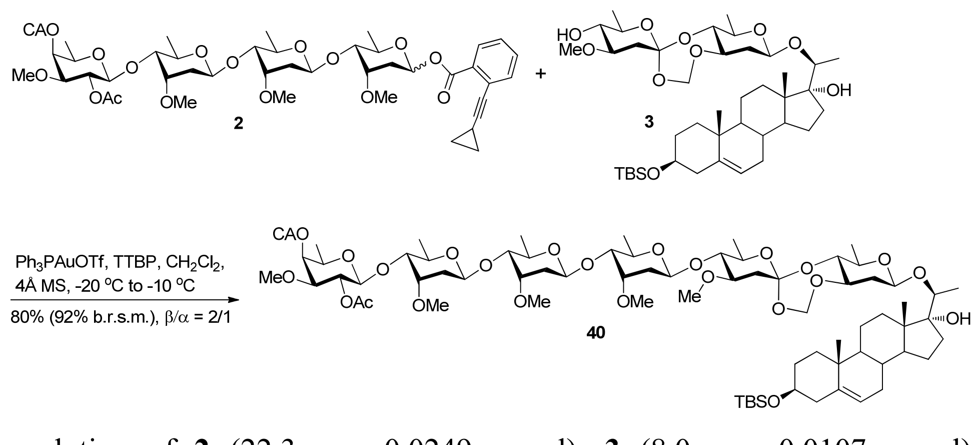

To a solution of **2** (22.3 mg, 0.0249 mmol), **3** (8.0 mg, 0.0107 mmol), and 2,4,6-tri-*tert*-butylpyrimidine (TTBP) (4.0 mg, 0.0161 mmol) in CH_2Cl_2 (2 mL) was added 4 Å MS at rt. After stirring for 30 min at –20 °C, a solution of PPh_3AuOTf in CH_2Cl_2 (0.05 mL, 0.1 M) was added to the mixture. The mixture was stirred for 2 h while warming to –10 °C, then another portion of PPh_3AuOTf in CH_2Cl_2 (0.05 mL, 0.1 M) was added to the reaction mixture. After stirring for 4 h at –10 °C, Et_3N was added to quench the reaction. The resulting mixture was filtered and concentrated. The residue was purified by flash chromatography (petroleum ether/CH_2Cl_2/EtOAc = 1:1:1) to afford **40** (8.3 mg, 53%), its α anomer (4.2 mg, 27%) as white foams, and

recovered **3** (1.0 mg, 13%). **40**: $[\alpha]^{26}_{D}$ = +15.4 (*c* = 0.18, $CHCl_3$); ^1H NMR (500 MHz, $CDCl_3$) δ 5.37 (d, *J* = 3.1 Hz, 1H), 5.31 (brs, 1H), 5.14 (d, *J* = 7.5 Hz, 1H), 5.07 (dd, *J* = 10.0, 8.0 Hz, 1H), 4.93 (dd, *J* = 9.7, 1.7 Hz, 1H), 4.77-4.73 (m, 3H), 4.58 (dd, *J* = 9.7, 1.6 Hz, 1H), 4.43 (d, *J* = 8.0 Hz, 1H), 4.20 (d, *J* = 2.2 Hz, 2H), 3.94-3.77 (m, 5H), 3.75-3.71 (m, 3H), 3.64-3.46 (m, 4H), 3.44 (s, 3H), 3.43 (s, 6H), 3.42 (s, 3H), 3.41-3.37 (m, 1H), 3.37-3.30 (m, 2H), 3.35 (s, 3H), 3.26 (t, *J* = 9.0 Hz, 1H), 3.21-3.17 (m, 3H), 2.46 (dd, *J* = 12.7, 5.1 Hz, 1H), 2.28-2.17 (m, 3H), 2.15-2.10 (m, 3H), 2.06 (s, 3H), 1.98-1.89 (m, 3H), 1.84-1.61 (m, 9H), 1.57-1.35 (m, 8H), 1.31 (d, *J* = 5.9 Hz, 3H), 1.29 (d, *J* = 6.3 Hz, 6H), 1.24 (s, 3H), 1.22 (d, *J* = 6.2 Hz, 3H), 1.20 (d, *J* = 6.2 Hz, 3H), 1.17 (d, *J* = 6.2 Hz, 3H), 1.04 (dd, *J* = 13.8, 3.8 Hz, 1H), 1.00-0.94 (m, 1H), 0.99 (s, 3H), 0.88 (s, 9H), 0.72 (s, 3H), 0.05 (s, 6H); ^{13}C NMR (125 MHz, $CDCl_3$) δ 169.5, 167.6, 141.6, 121.2, 113.8, 102.6, 101.0, 99.9, 99.8, 98.6, 86.5, 85.6, 84.1, 83.3, 82.8, 82.6, 82.6, 80.1, 79.3, 78.4, 77.8, 77.1, 76.4, 72.7, 70.7, 70.5, 70.1, 69.9, 69.1, 69.0, 68.5, 68.1, 58.3, 58.2, 58.1, 58.0, 57.8, 51.2, 49.8, 45.5, 43.0, 41.0, 38.5, 37.5, 37.0, 36.8, 36.7, 35.6, 35.2, 32.2, 32.1, 32.0, 31.1, 29.5, 26.1, 23.6, 22.8, 21.1, 20.7, 19.6, 18.4, 18.4, 18.4, 18.3, 18.2, 18.1, 17.2, 16.7, 14.3, -4.4; HRMS (ESI) calcd for $C_{73}H_{121}O_{25}ClSiNa$ $[M+Na]^+$ 1483.7547, found 1483.7552. **α anomer:** $[\alpha]^{25}_{D}$ = +28.9 (*c* = 0.28, $CHCl_3$); ^1H NMR (500 MHz, $CDCl_3$) δ 5.37 (d, *J* = 3.2 Hz, 1H), 5.31 (brs, 1H), 5.25 (d, *J* = 4.6 Hz, 1H), 5.15 (d, *J* = 7.6 Hz, 1H), 5.07 (dd, *J* = 10.0, 8.0 Hz, 1H), 4.78-4.75 (m, 3H), 4.58 (dd, *J* = 9.7, 1.6 Hz, 1H), 4.43 (d, *J* = 8.0 Hz, 1H), 4.23-4.17 (m, 3H), 3.88 (dd, *J* = 9.5, 6.2 Hz, 1H), 3.83 (dd, *J* = 9.6, 6.3 Hz, 1H), 3.79 (dd, *J* = 5.9, 3.0 Hz, 1H), 3.76-3.72 (m, 4H), 3.57 (dd, *J* = 9.5, 6.3 Hz, 1H), 3.55-3.45 (m, 3H), 3.43 (s, 3H), 3.42 (s, 3H), 3.41 (s, 3H), 3.39-3.31 (m, 2H), 3.35 (s, 6H), 3.28 (dd, *J* = 9.6, 2.6 Hz, 1H), 3.20 (ddd, *J* = 16.3, 9.6, 2.8 Hz, 2H), 2.48 (dd, *J* = 12.4, 4.9 Hz, 1H), 2.30-2.10 (m, 6H), 2.07 (s, 3H), 1.98-1.90 (m, 3H), 1.83-1.61 (m, 11H), 1.54-1.39 (m, 5H), 1.34 (d, *J* = 6.3 Hz, 3H), 1.32 (d, *J* = 6.0 Hz, 3H), 1.29 (d, *J* = 6.3 Hz, 3H), 1.25 (d, *J* = 6.4 Hz, 3H), 1.20 (d, *J* = 6.2 Hz, 3H), 1.18 (d, *J* = 6.0 Hz, 3H), 1.17 (d, *J* = 6.0 Hz, 3H), 1.15-1.10 (m, 1H), 1.06-1.02 (m, 1H), 0.99 (s, 3H), 0.97 -0.94 (m, 1H), 0.88 (s, 9H), 0.72 (s, 3H), 0.05 (s, 6H); ^{13}C NMR (125 MHz, $CDCl_3$) δ 169.5, 167.6, 141.6, 121.2, 113.8, 102.6, 101.0, 99.8, 99.7, 96.7, 86.5, 85.6, 84.1, 83.2, 82.6, 82.1, 80.6,

80.1, 79.3, 78.7, 78.4, 77.1, 76.4, 75.5, 72.7, 70.8, 70.6, 70.2, 69.9, 69.1, 68.6, 68.1, 63.2, 58.3, 58.2, 57.9, 57.4, 56.9, 51.2, 49.8, 45.5, 43.0, 41.0, 38.5, 37.5, 37.0, 36.7, 36.5, 35.6, 35.1, 32.6, 32.2, 32.1, 32.0, 31.1, 26.1, 23.6, 22.8, 21.0, 20.7, 19.6, 18.5, 18.4, 18.4, 18.2, 17.7, 17.2, 16.7, 14.3, -4.4; HRMS (ESI) calcd for $C_{73}H_{121}ClO_{25}Na$ $[M+Na]^+$ 1483.7547, found 1483.7544.

Periploside A (1)

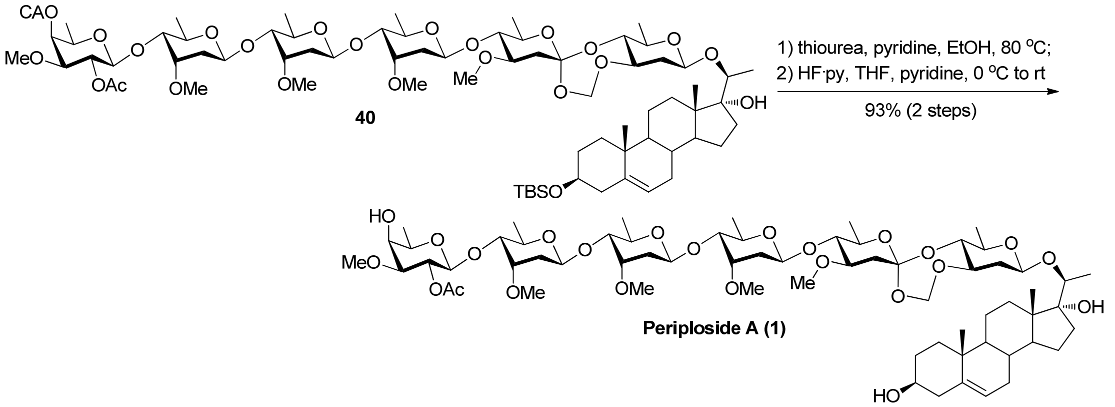

To a solution of **40** (4.7 mg, 3.2 μmol) in pyridine/EtOH (1.0 mL/1.0 mL) was added thiourea (10 mg, 0.13 mmol) at rt. After stirring for 2 h at 80 °C, the mixture was concentrated in vacuo to give a residue, which was purified by flash chromatography ($CHCl_3$/MeOH = 30:1) to afford a colorless syrup. The syrup was dissolved in THF/pyridine (1.5 mL/0.75 mL). HF·py (70% HF in pyridine, 0.10 mL) was added dropwise at 0 °C. After stirring for 40 h at rt, a saturated $NaHCO_3$ solution was added slowly to the mixture at rt. The resulting mixture was diluted with CH_2Cl_2, washed with saturated $NaHCO_3$ solution, and was then extracted with CH_2Cl_2 twice. The combined organic layer was washed with brine, dried over Na_2SO_4, and concentrated. The residue was purified by flash chromatography ($CHCl_3$/MeOH = 30:1) to afford periploside A (**1**) (3.8 mg, 93%) as a white foam: $[\alpha]^{25}_D$ = +12.6 (*c* = 0.12, MeOH); ^1H NMR (500 MHz, $CDCl_3$) δ 5.35 (brs, 1H), 5.13 (d, *J* = 7.5 Hz, 1H), 5.08 (dd, *J* = 9.7, 8.0 Hz, 1H), 4.93 (dd, *J* = 9.7, 1.7 Hz, 1H), 4.77-4.73 (m, 3H), 4.58 (dd, *J* = 9.8, 1.7 Hz, 1H), 4.38 (d, *J* = 8.0 Hz, 1H), 3.88-3.77 (m, 7H), 3.73 (dd, *J* = 12.6, 6.3 Hz, 1H), 3.65-3.48 (m, 5H), 3.44 (s, 3H), 3.44 (s, 3H), 3.43 (s, 3H), 3.42 (s, 3H), 3.41 (s, 3H), 3.38 (dd, *J* = 6.3, 3.0 Hz, 1H), 3.35-3.32 (m, 1H), 3.28-3.24 (m, 2H), 3.21-3.17 (m, 3H), 2.46 (dd, *J* = 12.7, 5.1 Hz, 1H), 2.31-2.18 (m, 4H), 2.13-2.09 (m, 2H), 2.07

(s, 3H), 2.00-1.89 (m, 3H), 1.86-1.62 (m, 8H), 1.55-1.39 (m, 8H), 1.37 (d, J = 6.4 Hz, 3H), 1.31 (d, J = 5.9 Hz, 3H), 1.29 (d, J = 6.4 Hz, 6H), 1.22 (d, J = 6.2 Hz, 3H), 1.19 (d, J = 6.2 Hz, 3H), 1.17 (d, J = 6.2 Hz, 3H), 1.15-1.12 (m, 1H), 1.08 (dd, J = 13.4, 3.5 Hz, 1H), 1.09-0.95 (m, 1H), 1.00 (s, 3H), 0.72 (s, 3H); ^{13}C NMR (125 MHz, $CDCl_3$) δ 169.4, 140.7, 121.6, 113.7, 102.5, 100.8, 99.7, 99.7, 98.5, 86.4, 85.4, 83.6, 83.1, 82.6, 82.4, 82.4, 81.5, 79.2, 78.2, 77.6, 77.2, 77.0, 76.5, 71.7, 70.9, 70.3, 69.9, 69.8, 68.8, 68.4, 68.1, 68.0, 58.6, 58.0, 57.9, 57.7, 57.4, 51.1, 49.6, 45.3, 42.3, 38.4, 37.2, 36.9, 36.7, 36.5, 35.9, 35.4, 35.2, 31.9, 31.8, 31.6, 30.9, 23.5, 21.0, 20.6, 19.4, 18.2, 18.2, 18.2, 18.0, 18.0, 17.0, 16.5, 14.1; HRMS (ESI) calcd for $C_{65}H_{106}O_{24}Na$ $[M+Na]^+$ 1293.6966, found 1293.6968.

Preparation of periploside analogs 41-43

C1'''-*epi*-periploside A (41)

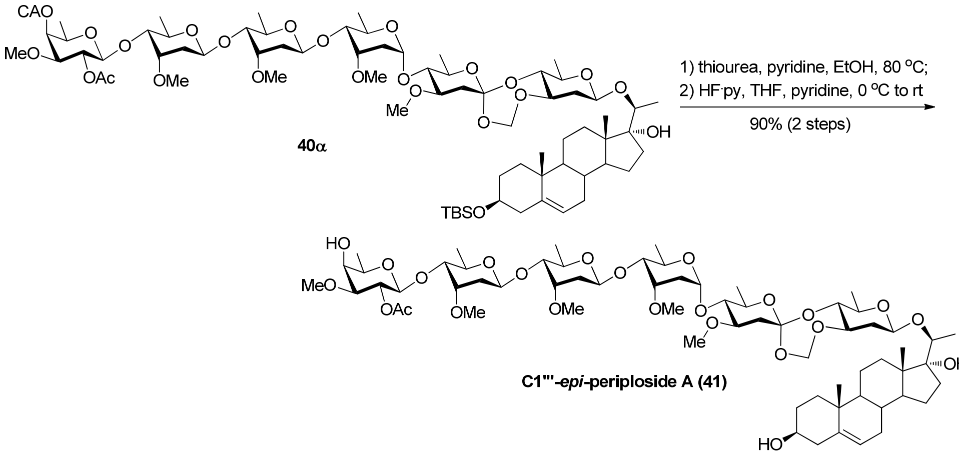

To a solution of **40α** (4.5 mg, 3.1 μmol) in pyridine/EtOH (1.0 mL/1.0 mL) was added thiourea (10 mg, 0.13 mmol) at rt. After stirring at 80 °C for 2 h, the mixture was concentrated in vacuo to give a residue, which was purified by flash chromatography ($CHCl_3$/MeOH = 30:1) to afford a colorless syrup. The syrup was dissolved in THF/pyridine (1.5 mL/0.75 mL). HF·py (70% HF in pyridine, 0.10 mL) was added dropwise at 0 °C. After stirring at rt for 40 h, a saturated $NaHCO_3$ solution was added slowly to the mixture. The resulting mixture was diluted with CH_2Cl_2, washed with saturated $NaHCO_3$ solution, and was then extracted with CH_2Cl_2 twice. The combined organic layer was washed with brine, dried over Na_2SO_4, and concentrated. The

residue was purified by flash chromatography ($CHCl_3$/MeOH = 30:1) to afford C1’’’-*epi*-periploside A (**41**) (3.5 mg, 90%) as a white foam: $[\alpha]^{25}_D$ = +35.1 (*c* = 0.14, $CHCl_3$); ^{1}H NMR (500 MHz, $CDCl_3$) δ 5.35 (brs, 1H), 5.25 (d, *J* = 4.7 Hz, 1H), 5.15 (d, *J* = 7.6 Hz, 1H), 5.08 (dd, *J* = 9.6, 8.1 Hz, 1H), 4.77-4.73 (m, 3H), 4.58 (d, *J* = 8.4 Hz, 1H), 4.38 (d, *J* = 7.9 Hz, 1H), 4.20 (dd, *J* = 9.6, 6.4 Hz, 1H), 3.92-3.71 (m, 7H), 3.59-3.51 (m, 5H), 3.44 (s, 3H), 3.43-3.38 (m, 1H), 3.42 (s, 3H), 3.41 (s, 3H), 3.41 (s, 3H), 3.35 (s, 3H), 3.35-3.30 (m, 1H), 3.29-3.26 (m, 2H), 3.19 (ddd, *J* = 14.0, 9.6, 2.7 Hz, 1H), 2.48 (dd, *J* = 12.3, 4.8 Hz, 1H), 2.37-2.18 (m, 5H), 2.16-2.08 (m, 2H), 2.07 (s, 3H), 2.01-1.89 (m, 3H), 1.88-1.60 (m, 11H), 1.51-1.40 (m, 4H), 1.37 (d, *J* = 6.4 Hz, 3H), 1.34 (d, *J* = 6.3 Hz, 3H), 1.32 (d, *J* = 5.9 Hz, 3H), 1.29 (d, *J* = 6.3 Hz, 3H), 1.19 (d, *J* = 6.5 Hz, 3H), 1.18 (d, *J* = 5.8 Hz, 3H), 1.17 (d, *J* = 4.3 Hz, 3H), 1.14-1.04 (m, 3H), 1.00 (s, 3H), 0.97 (dd, *J* = 11.8, 4.5 Hz, 1H), 0.73 (s, 3H); ^{13}C NMR (125 MHz, $CDCl_3$) δ 169.6, 140.8, 121.8, 113.8, 102.7, 101.0, 99.8, 99.7, 96.7, 86.5, 85.6, 83.7, 83.2, 82.5, 82.1, 81.7, 80.6, 79.3, 78.7, 78.4, 77.1, 76.7, 75.5, 71.9, 71.0, 70.5, 70.2, 69.9, 68.6, 68.2, 68.1, 63.3, 58.7, 58.0, 57.5, 57.4, 56.9, 51.2, 49.8, 45.5, 42.4, 38.5, 37.4, 37.0, 36.6, 36.5, 36.0, 35.2, 32.6, 32.1, 32.0, 31. 8, 31.1, 23.6, 21.1, 20.7, 19.5, 18.5, 18.4, 18.2, 18.1, 17.7, 17.2, 16.7, 14.3; HRMS (ESI) calcd for $C_{65}H_{106}O_{24}Na$ $[M+Na]^+$ 1293.6966, found 1293.6967.

Disaccharide 42

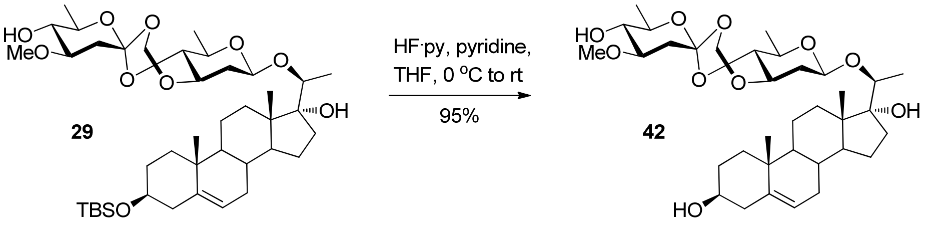

To a solution of **29** (7.0 mg, 9.32 μmol) in THF/pyridine (1.5 mL/0.75 mL) was added HF·py (70% HF in pyridine, 0.10 mL) dropwise at 0 °C. After stirring at rt for 40 h, a saturated $NaHCO_3$ solution was added slowly to the mixture. The resulting mixture was diluted with CH_2Cl_2, washed with saturated $NaHCO_3$ solution, and was then extracted with CH_2Cl_2 twice. The combined organic layer was washed with brine, dried over Na_2SO_4, and concentrated. The residue was purified by flash chromatography (petroleum ether/EtOAc = 1:1.5) to afford **42** (5.6 mg, 96%) as a

colorless syrup: $[\alpha]^{26}_{D}$ = −20.2 (c = 0.30, $CHCl_3$); ^1H NMR (500 MHz, $CDCl_3$) δ 5.35 (brs, 1H), 4.95 (d, J = 7.8 Hz, 1H), 4.84 (d, J = 7.9 Hz, 1H), 4.62 (dd, J = 9.8, 1.9 Hz, 1H), 3.77-3.69 (m, 3H), 3.56-3.49 (m, 1H), 3.41 (s, 3H), 3.40-3.34 (m, 2H), 3.33-3.27 (m, 1H), 3.23 (t, J = 9.1 Hz, 1H), 2.50-2.47 (m, 2H), 2.31-2.19 (m, 3H), 2.02 (s, 1H), 2.00-1.90 (m, 2H), 1.85-1.63 (m, 8H), 1.55-1.40 (m, 5H), 1.36 (d, J = 6.2 Hz, 3H), 1.34 (d, J = 6.2 Hz, 3H), 1.30 (d, J = 6.3 Hz, 3H), 1.14 (dd, J = 12.0, 5.7 Hz, 1H), 1.10-1.04 (m, 1H), 1.01 (s, 3H), 0.98 (dd, J = 11.8, 4.4 Hz, 1H), 0.73 (s, 3H); ^{13}C NMR (125 MHz, $CDCl_3$) δ 140.8, 121.8, 114.5, 101.0, 88.6, 85.5, 83.1, 79.3, 78.0, 77.3, 75.3, 71.9, 70.7, 70.3, 56.7, 51.2, 49.7, 45.5, 42.4, 38.5, 37.5, 37.4, 37.2, 36.6, 32.1, 32.0, 31.8, 31.1, 23.6, 20.7, 19.5, 19.2, 17.7, 17.1, 14.3; HRMS (ESI) calcd for $C_{35}H_{56}O_{10}Na$ $[M+Na]^+$ 659.3766, found 659.3785.

Disaccharide 43

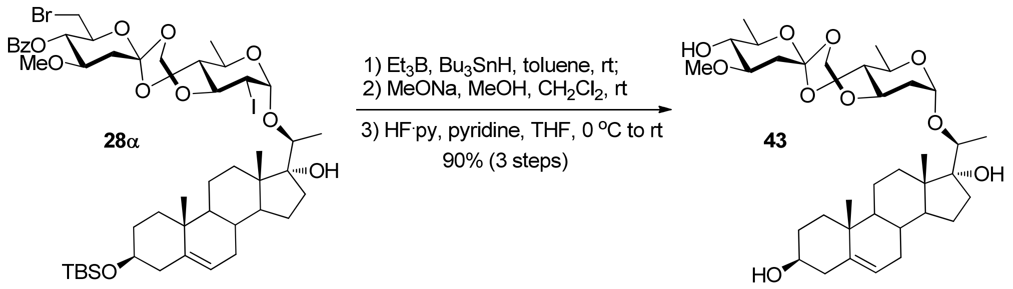

Compound **43** (5.1 mg, 90%, 3 steps) was prepared from **28α** (8.7 mg, 8.2 μmol) following a procedure similar to that for **28→42**. **43**: $[\alpha]^{24}_{D}$ = +32.0 (c = 0.11, $CHCl_3$); ^1H NMR (500 MHz, $CDCl_3$) δ 5.36 (brs, 1H), 5.00 (d, J = 3.8 Hz, 1H), 4.95 (d, J = 7.8 Hz, 1H), 4.87 (d, J = 7.9 Hz, 1H), 3.85-3.79 (m, 2H), 3.73 (dd, J = 9.3, 6.2 Hz, 1H), 3.68 (t, J = 8.9 Hz, 1H), 3.66-3.60 (m, 1H), 3.53 (tt, J = 10.4, 5.2 Hz, 1H), 3.43 (s, 3H), 3.33 (ddd, J = 11.6, 8.9, 4.7 Hz, 1H), 3.24 (t, J = 9.1 Hz, 1H), 2.52 (dd, J = 12.5, 4.7 Hz, 1H), 2.49 (brs, 1H), 2.30 (ddd, J = 12.9, 4.9, 1.7 Hz, 1H), 2.27-2.21 (m, 1H), 2.10 (dd, J = 12.9, 4.6 Hz, 1H), 2.03-1.93 (m, 2H), 1.87-1.75 (m, 7H), 1.67-1.59 (m, 2H), 1.55-1.42 (m, 5H), 1.35 (d, J = 6.2 Hz, 3H), 1.29 (d, J = 6.3 Hz, 3H), 1.23-1.19 (m, 1H), 1.15 (d, J = 6.1 Hz, 3H), 1.11-1.05 (m, 1H), 1.03-0.98 (m, 1H), 1.02 (s, 3H), 0.74 (s, 3H); ^{13}C NMR (125 MHz, $CDCl_3$) δ 140.9, 121.7, 114.5, 93.1, 88.6, 85.4, 79.3, 77.9, 75.6, 75.4, 71.9, 70.7, 66.5, 56.7, 51.3, 49.8, 45.8, 42.4, 39.2, 37.5, 37.4, 36.6, 36.2, 32.1, 32.0, 31.8, 31.2, 23.8, 20.7, 19.5, 18.9, 17.8, 14.2, 13.9;

HRMS (ESI) calcd for $C_{35}H_{56}O_{10}Na$ $[M+Na]^+$ 659.3766, found 659.3787.

Bioassay for the immunosuppressive activities[17]

Preparation of spleen cells from mice

BALB/C mice were sacrificed and spleens were removed aseptically. A single cell suspension was prepared after cell debris and clumps were removed. Erythrocytes were lysed using ammonium chloride buffer solution. The isolated lymphocytes were washed 3 times with PBS containing 2% FBS, and were re-suspended in RPMI 1640 medium at the indicated concentration.

Cytotoxicity assay

Fresh spleen cells (1 x 10^6) were cultured in 96-well flat plates with 200 μL of RPMI 1640 media containing 10% FBS, 100 U/mL penicillin and 100 μg/mL streptomycin in a humidified, 37 ^oC, 5% CO_2-containing incubator for 48 h, in the presence or absence of various concentrations of the compounds. 18 μL of MTT (5 mg/mL) was added to each well at the final 5 h culture. Then 90 μL of lysis buffer (10% SDS, 50% DMF, pH 7.2) was added to each well for 6–7 h and the absorbance values at 570 nm were read by microplate reader (Bio-Rad, Model 550).

T cell function assay

1 x 10^6 of fresh spleen cells were cultured for 48 h at the same conditions as mentioned above. The cultures were stimulated with 5 μg/mL of concanavalin A (ConA) to induce T cells proliferative responses. The compounds were added to cultures with indicated concentrations to test their bioactivities. Proliferation was assessed in terms of uptake of [^{3}H]-thymidine during last 8 h culture pulsing with 25 μCi of [^{3}H]-thymidine for each well, and then cells were harvested onto glass fiber filters by a Basic 96 harvester. The incorporated radioactivity was counted by a liquid scintillation counter (1540 MicroBeta Trilux, Perkin–Elmer Life Sciences).

Supplementary References

1. Schmidt, R. R. & Toepfer, A. Glycosylation with highly reactive glycosyl donors: efficiency of the inverse procedure. *Tetrahedron Lett.* **32**, 3353–3356 (1991).
2. Takatani, M., Nakano, J., Arai, M. A., Ishiwata, A., Ohta, H. & Ito, Y. Accelerated glycosylation under frozen conditions. *Tetrahedron Lett.* **45**, 3929–3932 (2004).
3. Oshima, Y., Hirota, T. & Hikino, H. Periplosides A, B and C, steroidal glycosides of *Periploca sepium* root barks. *Heterocycles* **26**, 2093–2098 (1987).
4. Werz, D. B. & Seeberger, P. H. Total synthesis of antigen *Bacillus anthracis* tetrasaccharide—creation of an anthrax vaccine candidate. *Angew. Chem. Int. Ed.* **44**, 6315–6318 (2005).
5. Li, Y., Yang, W., Ma, Y., Sun, J., Shan, L., Zhang, W.-D. & Yu, B. Synthesis of kaempferol 3-*O*-[2”,3”- and 2”,4”-di-*O*-(*E*)-*p*-coumaroyl] -α-L-rhamnopyranosides. *Synlett* 914–918 (2011).
6. Ma, Y., Li, Z., Shi, H., Zhang, J. & Yu, B. Assembly of digitoxin by gold(I)-catalyzed glycosidation of glycosyl *o*-alkynylbenzoates. *J. Org. Chem.* **76**, 9748–9756 (2011).
7. Noshita, T., Sugiyama, T., Kitazumi, Y. & Oritani, T. Phenolic Ferrier reaction and its application to the natural product synthesis. *Tetrahedron Lett.* **35**, 8259–6262 (1994).
8. Durham, T. B. & Roush, W. R. Stereoselective synthesis of functionalized precursors of the CDEF and CDE 2,6-dideoxy-tetra- and trisaccharide units of durhamycins A and B. *Org. Lett.* **5**, 1875–1878 (2003).
9. Yang, X., Fu, B. & Yu, B. Total synthesis of landomycin A, a potent antitumor angucycline antibiotic. *J. Am. Chem. Soc.* **133**, 12433–12435 (2011).
10. Wehlan, H. *et al.* Apoptolidin A: total synthesis and partially glycosylated analogues. *Chem. Eur. J.* **12**, 7378–7397 (2006).
11. Gundersen, L. L. & Benneche, T. Evidence for formation of chloromethoxy(trimethyl)silane. *Acta Chem. Scand.* **45**, 975–977 (1991).
12. Sharpless, K. B. *et al.* The osmium-catalyzed asymmetric dihydroxylation: a new

ligand class and a process improvement. *J. Org. Chem*. **57**, 2768–2771 (1992).

13. Yu, W. & Jin, Z. A new strategy for the stereoselective introduction of steroid side chain via α-alkoxy vinyl cuprates: total synthesis of a highly potent antitumor natural product OSW-1. *J. Am. Chem. Soc.* **123**, 3369–3370 (2001).
14. Yu, B. & Tao, H. Glycosyl trifluoroacetimidates. Part 1: preparation and application as new glycosyl donors. *Tetrahedron Lett.* **42**, 2405–2407 (2001).
15. Bussolo, V. D. *et al*. Synthesis of diastereoisomeric 6-deoxy-D-allal- and 6-deoxy-D-galactalderived allyl epoxides and examination of the regio- and stereoselectivity in nucleophilic addition reactions. Comparison with the corresponding 6-*O*-functionalized allyl epoxides. *Tetrahedron* **64**, 8188–8201 (2008).
16. Benneche, T., Gundersen, L.-L. & Undheim, K. (*tert*-Butyldimethylsilyloxy)methyl chloride: synthesis and use as *N*-protecting group in pyrimidinones. *Acta Chem. Scand., Ser. B* **42**, 384–389 (1988).
17. Feng, J. Q. *et al*. Immunosuppressive pregnane glycosides from *Periploca sepium* and *Periploca forrestii*. *Phytochemistry* **69**, 2716-2723 (2008).
